# Lower vs Higher Fluid Volumes in Adult Patients With Sepsis: An Updated Systematic Review With Meta-Analysis and Trial Sequential Analysis

**DOI:** 10.1016/j.chest.2023.04.036

**Published:** 2023-05-02

**Authors:** Praleene Sivapalan, Karen L. Ellekjaer, Marie K. Jessen, Tine S. Meyhoff, Maria Cronhjort, Peter B. Hjortrup, Jørn Wetterslev, Anders Granholm, Morten H. Møller, Anders Perner

**Affiliations:** a Department of Intensive Care, Rigshospitalet, Copenhagen University Hospital, Copenhagen, Denmark; b Collaboration for Research in Intensive Care (CRIC), Copenhagen, Denmark; c Research Center for Emergency Medicine, Aarhus University and University Hospital, Aarhus N, Denmark; d Department of Anesthesiology and Intensive Care, Aarhus University Hospital, Aarhus, Denmark; e Karolinska Institutet, Department of Clinical Science and Education, Södersjukhuset, Section of Anaesthesia and Intensive Care, Stockholm, Sweden; f Department of Cardiothoracic Anaesthesia and Intensive Care, The Heart Center, Rigshospitalet, University of Copenhagen, Copenhagen, Denmark; g Private Office, Tuborg Sundpark, Hellerup, Denmark

**Keywords:** fluid therapy, intensive care, sepsis, septic shock

## Abstract

**Background:**

IV fluids are recommended for adults with sepsis. However, the optimal strategy for IV fluid management in sepsis is unknown, and clinical equipoise exists.

**Research Question:**

Do lower vs higher fluid volumes improve patient-important outcomes in adult patients with sepsis?

**Study Design and Methods:**

We updated a systematic review with meta-analysis and trial sequential analysis of randomized clinical trials assessing lower vs higher IV fluid volumes in adult patients with sepsis. The coprimary outcomes were all-cause mortality, serious adverse events, and health-related quality of life. We followed the recommendations from the Cochrane Handbook and used the Grading of Recommendations Assessment, Development and Evaluation approach. Primary conclusions were based on trials with low risk of bias if available.

**Results:**

We included 13 trials (N = 4,006) with four trials (n = 3,385) added to this update. The meta-analysis of all-cause mortality in eight trials with low risk of bias showed a relative risk of 0.99 (97% CI, 0.89-1.10; moderate certainty evidence). Six trials with predefined definitions of serious adverse events showed a relative risk of 0.95 (97% CI, 0.83-1.07; low certainty evidence). Health-related quality of life was not reported.

**Interpretation:**

Among adult patients with sepsis, lower IV fluid volumes probably result in little to no difference in all-cause mortality compared with higher IV fluid volumes, but the interpretation is limited by imprecision in the estimate, which does not exclude potential benefit or harm. Similarly, the evidence suggests lower IV fluid volumes result in little to no difference in serious adverse events. No trials reported on health-related quality of life.

**Trial Registration:**

PROSPERO; No.: CRD42022312572; URL: https://www.crd.york.ac.uk/prospero/

## Graphical Abstract

**Figure:**
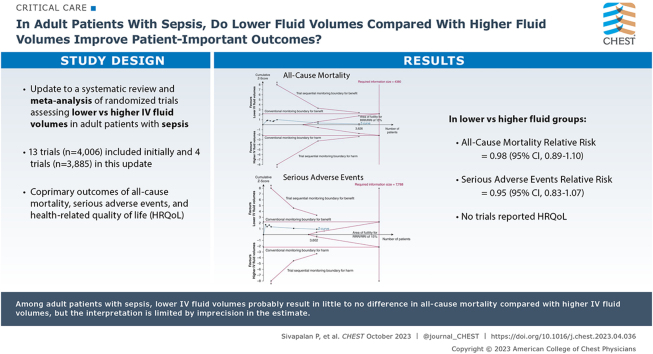

FOR EDITORIAL COMMENT, SEE PAGE 812

**Take-home Points**

**Study Question:** In adult patients with sepsis, do lower fluid volumes compared with higher fluid volumes improve patient-important outcomes?

**Results:** The relative risk of the coprimary outcomes was 0.98 (99% CI, 0.89-1.10) for all-cause mortality; for serious adverse events it was 0.95 (99% CI, 0.83-1.07) in the lower vs higher fluid groups; and no trials reported on health-related quality of life.

**Interpretation:** Among adult patients with sepsis, lower IV fluid volumes probably result in little to no difference in all-cause mortality compared with higher fluid volumes, but the interpretation is limited by imprecision in effect estimates, which does not exclude potential benefit or harm. Similarly, the evidence suggests that lower IV fluid volumes result in little to no difference in serious adverse events.

IV fluids are considered essential in the management of sepsis, but because current guidelines are supported by very low certainty evidence,^1^ additional research is warranted. Observational studies and randomized clinical trials (RCTs) report conflicting results on the effects of lower vs higher IV fluid volumes on patient-important outcomes.^2^, ^3^, ^4^, ^5^, ^6^, ^7^, ^8^ A previous version of this systematic review with meta-analysis of lower vs higher IV fluid volumes in adults with sepsis reported very low quantity and certainty of evidence.^9^ Hence, the strategies for IV fluid management in sepsis have been dominated by clinical equipoise. Several RCTs of fluid therapy in sepsis have been published,^10^, ^11^, ^12^, ^13^ which substantially increases the quantity of evidence. Therefore, we conducted an updated systematic review with meta-analysis to provide a summary of the available evidence on patient-important outcomes of lower vs higher IV fluid volumes in adult patients with sepsis.

## Study Design and Methods

We updated the previously published systematic review according to a prespecified, updated protocol registered in the International Prospective Register of Systematic Reviews, PROSPERO.^14^ Details on protocol specifications and deviations are provided in e-Appendix 1. We followed the recommendations by the Cochrane Handbook^15^; used the Grading of Recommendations Assessment, Development and Evaluation (GRADE) approach^16^; and reported the paper according to the Preferred Reporting Items for Systematic Reviews and Meta-Analyses statement (checklist in e-Appendix 2).^17^

### Types of Studies

We included RCTs comparing different strategies intended to obtain a separation in IV fluid volumes in hospitalized adults with sepsis. We imposed no restrictions regarding language, publication source, or status. We excluded quasi-randomized trials because of selection bias and crossover trials because of the nature of the design.^14^

### Types of Participants

Participants included adult patients with sepsis (as defined in the original trials) independent of hospital setting. Trials restricted to children were excluded.

### Types of Interventions

We included RCTs with a preplanned strategy for separation of IV fluid volumes or balances regardless of whether a separation was obtained.^14^ Trials using hemodynamic parameters as triggers for IV fluid administration were included, whereas trials with hemodynamic parameters as targets were excluded. We also excluded trials comparing different types of fluids and trials of resuscitation of severe blood loss and burns.

### Type of Outcome Measures

We assessed the following preplanned outcomes at the time closest to 90 days.

#### Primary Outcomes

We had the following three coprimary outcomes: (1) all-cause mortality, (2) proportion of patients with one or more serious adverse events (SAEs) (as defined in the original trials or any untoward medical occurrence that fulfills the International Council on Harmonization Guideline for Good Clinical Practice [ICH-GCP] definition^18^), and (3) health-related quality of life.

#### Secondary outcomes

Secondary outcomes included duration of mechanical ventilation, ventilator-free days, duration of vasopressor or inotropes, vasopressor-free days, use of renal replacement therapy (RRT), duration of RRT, RRT-free days, and incidence of acute kidney injury (AKI).

#### Exploratory outcomes

Exploratory outcomes included transfusion with any form of blood products, ICU length of stay, and hospital length of stay.

### Search Methods for Identification of Studies

We systematically searched the Cochrane Library (2022, Issue 9), MEDLINE (1946 onward), Embase (1974 onward), Science Citation Index Expanded and Conference Proceedings Citation Index (1990 onward), BIOSIS Previews (1969 onward), and Epistemonikos (no year-based restriction). The last search was performed September 6, 2022 (last search in the previous review was conducted on April 29, 2019). In addition, we searched for ongoing trials in clinical trial registers (ie, ClinicalTrials.gov, EU Clinical Trials Register, World Health Organization International Clinical Trials Registry Platform search portal). The full search strategy is available in e-Appendix 3.

### Data Collection

#### Study Selection

Two of three investigators (P. S., K. L. E., or M. K. J.) screened articles independently and in duplicate for inclusion based on titles and abstracts. Potentially eligible articles were independently and in duplicate evaluated in full text by two investigators (P. S., K. L. E., or M. K. J.). Disagreements were resolved by discussion with a third senior author (M. H. M. or A. P.).

#### Data Extraction and Management

Two authors independently and in duplicate extracted data using a standardized data extraction form (P. S., K. L. E., M. K. J., T. S. M., M. C.) (e-Appendix 4). Extracted data items included trial characteristics (year of publication, country, and inclusion period), characteristics of trial population (inclusion and exclusion criteria), intervention and control (types of fluids used, criteria for administering fluids, volumes of resuscitation fluids, total fluid input, use of diuretics or fluid removal by RRT, and fluid balance over the study period), and data on the predefined outcomes. If prespecified data were not available, corresponding authors were contacted for further data at least twice. Additional data and clarifications were provided by the authors of 12 trials.^10^, ^11^, ^12^, ^13^^,^^19^, ^20^, ^21^, ^22^, ^23^, ^24^, ^25^, ^26^

#### Risk of Bias

Two authors (P. S., K. L. E., or M. K. J.) independently assessed the risk of bias for all outcomes of the included trials using the revised Risk of Bias 2 tool by the Cochrane Collaboration.^15^^,^^27^ Risk of bias was assessed by P. S. and K. L. E. in one trial in which M. K. J. was first author,^13^ and equally K. L. E. and M. K. J. assessed risk of bias for the trial in which P. S. was involved.^12^ Disagreements were resolved by discussion with a third author (A. G. or M. H. M.). We assessed the following five bias domains for each outcome in the included trials: (1) bias arising from the randomization process, (2) bias because of deviations from intended interventions, (3) bias because of missing outcome data, (4) bias in measurement of the outcome, and (5) bias in selection of the reported results. The overall risk of bias adjudication for each specific outcome was based on all five domains (ie, trial outcomes with low risk of bias in all five domains were judged as having overall low risk of bias; trials with one or more domains with some concerns were adjudicated as having overall some concerns or high risk of bias; and if one or more domains were judged as having high risk of bias, we classified it as having overall high risk of bias).

Our primary conclusions were based on trials with low risk of bias with estimates for all trials also presented. We used the pooled estimate of trials with some concern for SAEs because we found no trials with low risk of bias.

### Statistical Analysis

#### Data Synthesis

We used R version 4.1.2 (R Core Team, R Foundation for Statistical Computing) to conduct the conventional meta-analyses using the meta R package, whereas Bayesian analyses were conducted using R version 4.1.3 and Stan version 2.29.2^28^ through the brms R package.^29^ We used Trial Sequential Analysis version 0.9.5.10 beta (Copenhagen Trial Unit, Centre for Clinical Intervention Research, Rigshospitalet; available from http://www.ctu.dk/tsa) to conduct the trial sequential analyses (TSAs).

#### Meta-analysis

Intention-to-treat (ITT) populations were used if available; otherwise, we used data from the modified ITT populations, as defined in the original trials.

Dichotomous outcomes were analyzed as relative risks (RRs), whereas continuous outcomes were analyzed as mean differences, both with 97% CIs for primary and exploratory outcomes and 99% CIs for secondary outcomes, respectively. Analyses were conducted using the raw numbers (dichotomous outcomes) and raw means and SDs (continuous outcomes) in each group.

We used both random effects models (assuming the true intervention effects are not identical in the included trials, but follow a normal distribution) and fixed effect models (assuming the true intervention effect is fixed in both direction and magnitude in the included trials), and reported the most conservative estimate according to highest *P* value.^14^^,^^30^ We considered *P* < .033, *P* < .0125, and *P* < .025 statistically significant for primary, secondary, and exploratory outcomes, respectively, because of protocolized adjustment for multiplicity based on the number of outcomes (e-Appendix 5).^14^

We estimated the number of patients with one or more SAEs in three different analyses. Highest proportion of SAEs or serious adverse reactions (as defined in the original trial) was available in six out of 13 trials. These data were used in the primary analysis of SAEs and in the subgroup and sensitivity analyses for missing data. Further two analyses were conducted in line with the analyses of SAEs in the previous review with the highest proportion of SAEs, including mortality based on ICH-GCP definition and the cumulated number of reported SAEs^9^ (e-Appendix 6, e-Tables 1-4).

#### Assessment of Heterogeneity

Forest plots were assessed for overlap of CIs between the trials. Moreover, heterogeneity was assessed with the inconsistency (*I*^2^) and diversity (*D*^2^) statistics.^15^^,^^31^ Potential heterogeneity was addressed in the planned subgroup analyses.

#### Assessment of Publication Bias

We assessed publication bias for outcomes with at least 10 trials included using the Harbord test for a funnel plot asymmetry considering *P* < .05 as statistically significant.^32^

#### Assessment of Risk of Random Errors

We used TSA to assess the risk of random errors for each outcome because of repetitive testing. TSA estimates the required information size (RIS) needed to detect or reject an a priori intervention effect in a meta-analysis and widens the CIs (TSA-adjusted CIs) in analyses when the RIS has not been reached.^31^ We applied trial sequential monitoring boundaries based on previous findings,^33^ and our definitions on clinically important effects were protocolized^14^ according to an a priori 15% RR reduction for dichotomous outcomes and a mean difference of 1 day, 24 h, or 1 unit of RBCs for the secondary or exploratory outcomes.

To match a family-wise error rate of 5%, we used an α of 3.3%, 1.25%, and 2.5% for primary, secondary, and exploratory outcomes, respectively (e-Appendix 5). A β of 10% (90% power) was used, and for binary outcomes we set the unweighted control event proportions as per the included trials, and used the empirical variances for continuous outcomes. For the random effects models heterogeneity adjustment was based on the increase in model variances changing from a fixed effect model to a random effects model (*D*^2^). This was set to 0% in the fixed effect models.^14^

#### Missing Data

We conducted sensitivity analyses for patients who were lost to follow-up or excluded in the modified ITT populations with best-worst case scenarios and worst-best case scenarios.^14^

#### Subgroup Analyses

We conducted the following six preplanned subgroup analyses: (1) overall low vs some concern or high risk of bias, (2) successful vs unsuccessful separation in fluid volumes as author-defined in each study, (3) patients with sepsis vs septic shock, (4) fluid-only interventions vs complex hemodynamic protocols, (5) earlier vs later resuscitation phase of sepsis, and (6) medical vs surgical treatment.^14^ Definitions of subgroups including the hypothesized direction of effect are available in the protocol.^14^ We used the χ^2^ test to assess the statistical heterogeneity across subgroups considering *P* = .10 as significant.

#### Bayesian Analysis

We conducted a secondary Bayesian analysis of all trials reporting the coprimary outcome, all-cause mortality, which was prespecified in the updated systematic review PROSPERO registration, but not included in the previously published protocol for the original version of this systematic review^14^ (e-Appendix 1). Bayesian fixed effect and random effects meta-analyses of all-cause mortality were conducted by analyzing crude RRs (with their SEs, both on the log RR scale) from the included trials. The primary analyses used weakly informative priors for the treatment effects. Details on priors and model diagnostics are provided in e-Appendix 6. Results are summarized using the median RRs from the posterior distributions as point estimates with 95% percentile-based credible intervals.

#### Assessment of the Overall Certainty of Evidence

Two authors (P. S. and K. L. E.) independently assessed the certainty of evidence using the GRADE methodology.^34^ The overall certainty of evidence was rated high, moderate, low, or very low based on evaluation of identified risks of bias, inconsistency, indirectness, imprecision, and publication bias.

## Results

**Figure 1:**
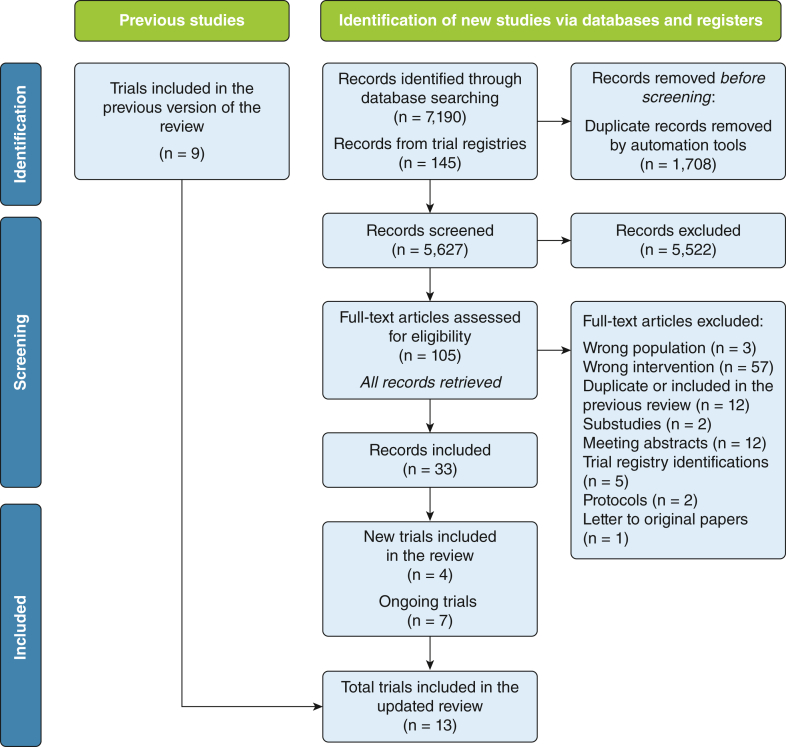
Trial flowchart.

In this updated review, we screened 5,627 new records, assessed 105 new articles in full text, and included four trials^10^, ^11^, ^12^, ^13^ (n = 3,385) in addition to the nine trials^19^, ^20^, ^21^, ^22^, ^23^, ^24^, ^25^, ^26^^,^^35^ (n = 621) included in the previous review. Eight eligible trials are still ongoing, but not included because the results are not yet available.^36^, ^37^, ^38^, ^39^, ^40^, ^41^, ^42^, ^46^ A total of 4,006 patients were randomized in the 13 included RCTs (Fig 1).^11^, ^12^, ^13^^,^^19^, ^20^, ^21^, ^22^, ^23^, ^24^, ^25^, ^26^^,^^35^

### Characteristics of Trials

The trials were published from 2015 to 2023. Six trials were single-center trials,^19^^,^^22^^,^^24^, ^25^, ^26^^,^^35^ and seven trials were multicenter trials,^10^, ^11^, ^12^, ^13^^,^^20^^,^^21^^,^^23^ of which the two largest trials included 1,563 and 1,549 patients, respectively.^10^^,^^12^ Ten trials included patients with septic shock in ICU settings,^11^^,^^12^^,^^19^, ^20^, ^21^, ^22^^,^^24^, ^25^, ^26^^,^^35^ and three trials included patients with sepsis in EDs.^10^^,^^13^^,^^23^ Patients had received IV fluid before randomization in nine trials.^10^, ^11^, ^12^, ^13^^,^^19^, ^20^, ^21^, ^22^^,^^26^ Eight trials had successful separation in fluid volumes,^10^, ^11^, ^12^, ^13^^,^^20^^,^^21^^,^^23^^,^^26^ whereas five trials were unsuccessful.^19^^,^^22^^,^^24^^,^^25^^,^^35^ Trial characteristics are presented in Table 1^43^, ^44^, ^45^ and further detailed in e-Table 5.

**Table 1:** Characteristics of the Included Trials

Study	Country	Inclusion Period	No. of Patients^a^; No. of Trial Sites	Clinical Setting	Population	Timing/Type of Fluids	Intervention	Comparator	Outcomes
Chen and Kollef^19^	United States	January 2014 to December 2014	82; 1	Medical ICU	≥ 18 y of age, septic shock,^b^ vasopressors ≥ 12 h, received 30 mL/kg IV fluid Exclusion: RRT before admission, pregnancy, comfort-only goals	≥ 12 h after initial resuscitation Saline or ringer lactate in restrictive group Fluids at physician’s discretion in liberal group	PLR test or fluid bolus → Assess PPV, IVC distention index, and SV → Additional fluid bolus of 500 mL if responsive If not responsive minimization therapy: discontinue IV fluids, diuretics, encourage RRT	Standard care PPV, IVC distention index, and SV available for physicians	In-hospital mortality Use of RRT Duration of MV VFDs Duration of vasopressor Vasopressor-free days
Cronhjort et al^22^	Sweden	February 2014 to January 2016	34; 1	Surgical ICU	Adult, septic shock, vasopressors, received 30 mL/kg IV crystalloids Exclusion: > 12 h septic shock,contraindication to femoral line or PLR, elevated ICP, imminent death	After initial resuscitation Fluids at physician’s discretion in both groups	Fluid bolus → PLR Further bolus based on SVI (measured by PiCCO)	Standard care PLR not allowed	30-d mortality SAEs ICU LOS
Hjortrup et al^20^	Denmark, Finland	September 2014 to August 2015	151; 9	General ICU	≥ 18 y of age, ICU, septic shock, SBP < 90 mm Hg, heart rate > 140 beats/min, lactate ≥ 4 mM, vasopressors < 12 h, received 30 mL/kg IV fluid, ongoing vasopressor Exclusion: RRT, K^+^ > 6mM, creatinine > 350 μmol/L, Fio_2_ > 0.8 and PEEP < 10 cm H_2_O, life-threatening bleeding, burns, comfort-only goals, consent not obtainable	After initial resuscitation Only crystalloids in both groups Colloids regarded as protocol violation	Resuscitation fluid restriction: 250- to 500-mL bolus if lactate ≥ 4 mM, MAP < 50 mm Hg, mottling score > 2, urine ≤ 0.1 mL/kg/h (first 2 h)	Standard care: bolus as long as hemodynamic improvement Variables at physician’s choice	90-d mortality SARs Use of RRT RRT-free days Duration of MV VFDs Blood transfusions ICU LOS
Richard et al^26^	France	July 2007 to July 2013	60; 1	Medical ICU	≥ 18 y of age, septic shock received ≥ 25 mL/kg IV fluid hypotension ≤ 12 h Exclusion: pregnancy, acute cerebral, cardiac, or pulmonary events, cannulation contraindicated, uncontrolled bleeding, burns, trauma/acute surgery, inclusion in another RCT, treatment withdrawal, consent not obtainable, no affiliation to French social security	After initial resuscitation Type of fluid at physician’s discretion in both groups	Preload dependence: PLR → Fluid bolus 500 mL based on PPV or SV	Fluid bolus 500 mL if CVP < 8 mm Hg	28-d mortality VFDs Duration of vasopressor Blood transfusions ICU LOS
van Genderen et al^35^	The Netherlands	October 2011 to November 2013	30; 1	ICU	≥ 18 y of age, ICU, severe sepsis/septic shock (vasopressor requirement or lactate ≥ 3 mEq/L) Exclusion: hypothermia, Raynaud or peripheral vascular disease, acute coronary syndrome or pulmonary edema, burn or trauma, liver failure, cannulation contraindicated, aerobic cause of hyperlactatemia, neurologic insult, do not resuscitate, pregnancy, recent participation in another study, inability to start study ≥ 4 h	Within 4 h of ICU admission Fluid boluses of (ie, HES 6% 130/0.4) in both groups	Fluid challenge 250 mL HES → If sufficient peripheral perfusion, discontinue fluids	Fluid challenge 250 mL HES → Hemodynamic goals based on 2012 SSC	ICU mortality VFDs ICU LOS Hospital LOS
Macdonald et al^23^	Australia	October 2016 to March 2018	99; 8	ED	≥ 18 y of age, ED, sepsis (Sepsis-3^43^), SBP < 100 mm Hg despite minimum 1-L IV crystalloid within 1 h, possible to start study within 2 h Exclusion: nonsepsis hypotension requirement for fluid replacement transferals > 2 L IV fluids, acute surgery, < 18 y of age, pregnancy, imminent death, patient wishes, fluids or vasopressors contraindicated	After at least 1 L fluid Crystalloids in both groups Hypotonic fluids, colloids, and 0.9% saline avoided Blood products and albumin at physician’s discretion	Vasopressor to MAP ≥ 65 mm Hg If altered perfusion: crystalloid bolus 250 mL allowed each hour Up to 1 L additional IV fluid allowed as safety measure Maintenance fluid of max 2 mL/kg/h if required	Fluid bolus 1 L → If SBP < 90 mm Hg, MAP < 65 mm Hg further 500-mL bolus every 30 min If persistent hypotension: NE to maintain MAP 65-70 mm Hg Maintenance fluid if required	90-d mortality SAEs Use of RRT RRT-free days Duration of MV VFDs Duration of vasopressor VFDs ICU LOS Hospital LOS
Lanspa et al^24^	United States	January 2015 to May 2017	30; 1	ED, ICU	≥ 18 y of age, septic shock,^44^ CVC, and arterial catheter Exclusion: inclusion criteria > 6 h, moribund patient, pregnancy, incarceration, acute surgery, chest/abdominal pathology, contraindicating TTE, protocol not possible because of physician’s or patient’s directives	As soon as possible after arrival to ICU Crystalloids in both groups Other fluid types not reported	Both groups: hourly assessment for 6 h – if intervention then assessment after 30 min ECHO group: If MAP < 65 mm Hg and lactate clearance < 10% → ECHO → 1 L IV fluid if IVC collapsing, if IVC not collapsing increase NE, if myocardial dysfunction and MAP < 70 mm Hg add/increase dobutamine	EGDT group: If CVP < 8 mm Hg → 1 L IV fluid If MAP < 65 mm Hg → Add/increase NE If Scvo_2_ < 70% → Add/increase dobutamine	28-d mortality VFDs ICU LOS
Semler et al^25^	United States	August 2014 to February 2016	30; 1	Medical ICU	≥ 18 y of age, ≥ 2 SIRS criteria, receiving antibiotics, shock (MAP < 60 mm Hg) or respiratory failure Exclusion: > 48 h since meeting inclusion criteria, consent unobtainable, known allergy to diuretics, medical diagnoses and comorbidities contraindicating the intervention, expected survival < 24 h, withdrawal of life support, choice of physician or study investigator	Within 48 h of sepsis and cardiopulmonary dysfunction IV crystalloids at physician’s discretion	No maintenance fluids, concentrate fluids with medication Shock: only IV fluids if oliguria or increasing vasopressor Without shock: IV fluids only if oliguria and continuous furosemide titrated to fluid output > fluid input	Usual care	In hospital- mortality Use of RRT RRT-free days VFDs Blood transfusions Vasopressor-free days
Corl et al^21^	United States	November 2016 to February 2018	109; 2	ED, medical ICU	≥ 18 y of age, admitted to ICU from ED, sepsis (Sepsis-2^44^ or deemed sepsis by attending physician), 1 L IV fluid, MAP < 65 mm Hg or lactate ≥ 4 mM Exclusion: primary diagnosis other than sepsis, fluid wasting condition, diagnosis requiring high-volume IV resuscitation, acute surgery or ECMO, pregnancy, incarcerated, received > 60 mL/kg IV fluid before randomization	After vital signs collected in ED triage Restrictive group: all saline, Ringer’s lactate, and sodium bicarbonate (resuscitative boluses and maintenance fluids)	72-h protocol: maximum 60 mL/kg resuscitative IV fluids If weight > 100 kg max 6,000 mL allowed IV fluids received before randomization included	Usual care	30-d mortality 60-d mortality SAEs Duration of vasopressor VFDs Duration of mechanical ventilation Vasopressor-free days Incidence of AKI Use of RRT Blood transfusions ICU LOS Hospital LOS
Douglas et al^11^	United States, United Kingdom	October 2016 to February 2019	ITT 150;13 mITT 124^c^	ED, ICU	18 y of age, anticipated ICU admission, sepsis or septic shock (defined as ≥ 2 SIRS criteria and a suspected or documented infection), MAP ≤ 65 mm Hg after ≥ 1 L IV fluid and < 3 L, enrollment within < 24 h of hospital arrival Exclusion: > 3 L IV fluid, do-not-resuscitate order, hemodynamic instability because of active hemorrhage, acute cerebral vascular event, acute coronary syndrome, acute pulmonary edema, status asthmaticus, major cardiac arrhythmia, drug overdose, burn or trauma, status epilepticus, indication for immediate surgery, PLR contraindication, pregnancy, incarceration, transferred from another hospital	After initial treatment in ED Type of fluid at physician’s discretion in both groups	PLR before any treatment of hypoperfusion → If SV > 10% → 500-mL IV fluid bolus → Reassess MAP/SBP If SV < 10% → Titrate pressors to MAP ≥ 65 mm Hg, repeat PLR after significant escalation	Standard care at physician’s discretion The use of dynamic fluid assessment to determine fluid responsiveness was prohibited	30-d mortality SAEs Use of RRT Duration of mechanical ventilation Duration of vasopressor ICU LOS Hospital LOS
Jessen et al^13^	Denmark	November 2021 to December 2021	123; 3	ED	≥ 18 y of age, unplanned ED admission, expected hospital stay > 24 h, sepsis (defined as infection suspected by physician, blood cultures, IV antibiotics administered or planned, and infection-related increase in SOFA score > 2) Exclusion: received ≥ 500-mL IV fluids, vasopressor or invasive ventilation started before screening, severe bleeding, prior enrollment in the trial, pregnancy, survival expectancy < 24 h	Initial resuscitation IV crystalloids in both groups	250-mL bolus could be given if severe hypoperfusion or circulatory with lactate ≥ 4 mM, SBP < 90 mm Hg, urine < 0.1 mL/kg/h (first 4 h) or mottling score > 2 Correction of overt fluid losses or if oral/enteral fluid was contraindicated to correct dehydration or electrolyte imbalances or ensure total fluid input of 1 L/d The protocol was paused if the patient underwent surgery during the first 24 h	Standard care at physician’s discretion	90-d mortality SAEs Use of RRT Incidence of AKI Duration of RRT Blood transfusions ICU LOS Hospital LOS
Meyhoff et al^12^	Denmark, Norway, Sweden, Switzerland, Italy, Czech Republic, United Kingdom, Belgium	November 2018 to November 2022	1,549; 31	General ICU	≥ 18 y of age, admitted to ICU, septic shock according to Sepsis-3 criteria^43^: suspected or confirmed infection, lactate ≥ 2 mM, ongoing vasopressor and received at least 1 L of IV fluid Exclusion: septic shock > 12 h, life-threatening bleeding, acute burn injury (> 10% BSA), pregnancy, consent not obtainable	After initial resuscitation Isotonic IV crystalloids in both groups for circulatory impairment and losses, albumin only if large amounts of ascites were removed	250- to 500-mL bolus could be given if severe hypoperfusion or circulatory with lactate ≥ 4 mM, MAP < 50 mm Hg, urine < 0.1 mL/kg/h (first 2 h) or mottling score > 2 Correction of overt fluid losses or if oral/enteral fluid was contraindicated to correct dehydration or electrolyte imbalances or ensure total fluid input of 1 L/d Other reasons for IV fluid were regarded as protocol violations	Standard care: bolus as long as hemodynamic improvement No fluid was regarded as protocol violation	90-d mortality SAEs Duration of mechanical ventilation VFDs Duration of vasopressor Vasopressor-free days Use of RRT Incidence of AKI Duration of RRT RRT-free days Blood transfusion ICU LOS Hospital LOS
National Heart, Lung, and Blood Institute Prevention and Early Treatment of Acute Lung Injury Clinical Trials Network et al^10^	United States	March 2018 to January 2022	1,563; 60	ED, ICU	≥ 18 y of age, suspected or confirmed infection, sepsis-induced hypotension (SBP < 100 mm Hg or MAP < 65 mm Hg after at least 1 L of fluid Exclusion: inclusion criteria > 4 h or hospital admission > 24 h, received > 3 L of IV fluids, nonsepsis hypotension, nonsepsis severe volume depletion, pulmonary edema or fluid overload, withdrawal of life support, protocol not possible because of physician’s directives or immediate surgery, pregnancy, consent not obtainable	Initial resuscitation Isotonic IV crystalloids in both groups	Vasopressor as primary treatment for sepsis-induced hypotension and halt all bolus and maintenance fluid; up to 2 L of total fluid at discretion of physicians Afterward, rescue fluids (500-mL boluses) permitted for prespecified indications suggesting severe intravascular volume depletion	Liberal protocol: halt maintenance Give initial 2 L followed by 500-mL boluses based on clinical triggers (eg, tachycardia) with rescue vasopressors October 2019 amended to initial 1 L if heart rate and BP stabilized and clinically volume repleted	90-d mortality SAEs Duration of mechanical ventilation VFDs Duration of vasopressor Use of RRT RRT-free days Blood transfusion Vasopressor-free days

Unless otherwise stated the standard definitions for sepsis and septic shock are used.^45^ AKI = acute kidney injury; BSA = body surface area; CVC = central venous catheter; CVP = central venous pressure; ECHO = echocardiogram; ECMO = extracorporeal membrane oxygenation; EGDT = early goal directed therapy; HES = hydroxy-ethyl starch; ICP = intracranial pressure; ITT = intention-to-treat; IVC = inferior vena cava; K+ = potassium ion; LOS = length of stay; MAP = mean arterial pressure; mITT = modified intention-to-treat; MV = mechanical ventilation; NE = norepinephrine; PEEP = positive-end expiratory pressure; PiCCO = pulse index continuous cardiac output; PLR = passive leg raise; PPV = pulse pressure variability; RCT = randomized clinical trial; RRT = renal replacement therapy; SAE = serious adverse event; SAR = serious adverse reaction; SBP = systolic BP; Scvo_2_ = central venous oxygen saturation; SIRS = systemic inflammatory response syndrome; SOFA = Sequential Organ Failure Asessment, SSC = Surviving Sepsis Campaign; SV = stroke volume; SVI = stroke volume index; TTE = transthoracic echocardiogram, VFD = ventilator-free day.

a No. of patients reported in ITT analysis.

b Criteria not defined.

c mIIT was defined as all patients who signed consent, met study eligibility criteria, were assigned randomly, and received monitoring for 72 h or ICU discharge if earlier.

### Description of the Intervention

The interventions varied between the included trials. Six trials reduced fluid volumes by assessing fluid responsiveness in combination with passive leg raise maneuvers,^11^^,^^19^^,^^22^^,^^26^ echocardiography,^24^ and using fluid bolus tests.^35^ Fixed hemodynamic triggers were used in six trials,^10^^,^^12^^,^^13^^,^^20^^,^^21^^,^^25^ among which four allowed additional hemodynamic assessment.^10^^,^^12^^,^^13^^,^^20^ In two trials, fluid volumes were reduced by using early intervention including vasopressors.^10^^,^^23^ The control group represented standard care in most trials,^12^^,^^13^^,^^19^, ^20^, ^21^^,^^23^^,^^25^^,^^35^ and most trials recommended crystalloids only.^10^^,^^12^^,^^13^^,^^20^^,^^23^, ^24^, ^25^

### Risk of Bias

An overview of risk of bias for all outcomes including adjudications is provided in e-Table 6. For all-cause mortality, one trial was judged as having overall high risk of bias, four trials were judged as having some concern for risk of bias, and the remaining eight trials were judged as having overall low risk of bias. Some concern/high risk of bias was because of the domains: bias in selection of the reported results and bias because of deviations from intended intervention. All six trials reporting SAEs were judged as having overall some concerns, mainly because of risk of bias in measurement of the outcome. For the secondary and exploratory outcomes, trials classified as having some concerns were because of bias in selection of the reported results and bias in measurement of the outcome.

### Primary Outcomes

#### Mortality

**Figure 2:**
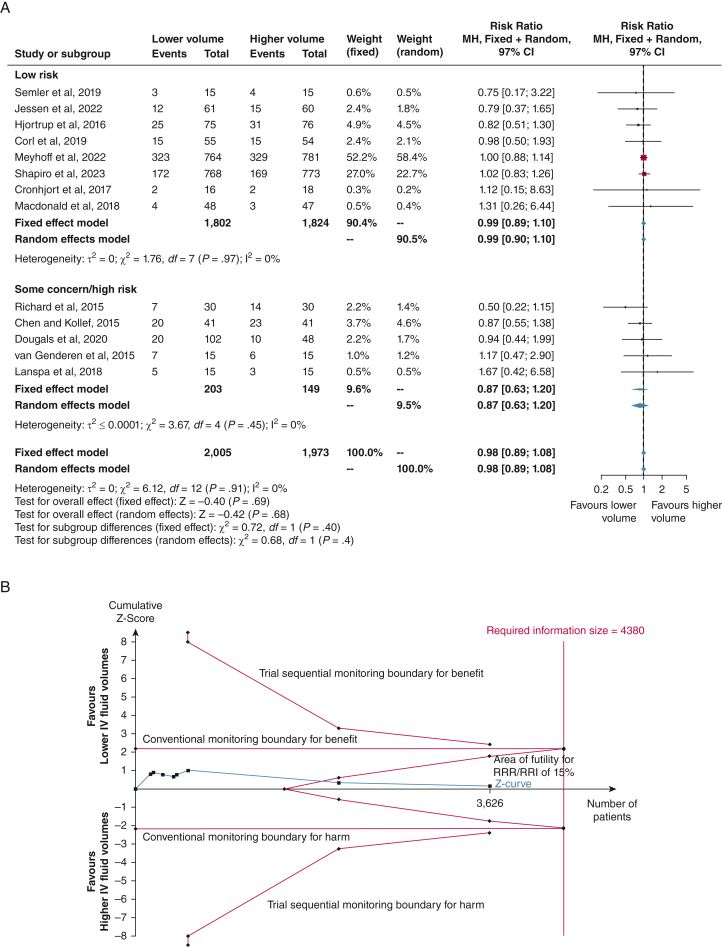
A-B, All-cause mortality. A, Meta-analysis of all-cause mortality in eight trials with low risk of bias (RoB) and five some concern/high concern RoB trials. The conventional meta-analysis of low RoB trials showed no statistically significant difference on mortality with lower vs higher fluid volumes (fixed effect model; relative risk, 0.99; 97% CI, 0.89-1.10; *P* = .69; *I*^2^ = 0%). B, Trial sequential analysis (TSA) for all-cause mortality in eight low RoB trials. We used a control event proportion of 31.1%, α of 3.3% (two-sided), β of 10% (power 90%), and an a priori relative risk reduction of 15% in the analysis. The TSA-adjusted CI in the fixed effect model was 0.89 to 1.11 with a diversity D^2^ of 0%. The blue cumulative z curve crossed the area of futility. Therefore, the TSA is conclusive, and a relative risk reduction of 15% is unlikely. A total of 83% (n = 3,626) of the required information size of 4,380 patients was accrued. MH = Mantel-Haaenszel; RRI = relative risk increase; RRR = relative risk reduction.

**Figure 3:**
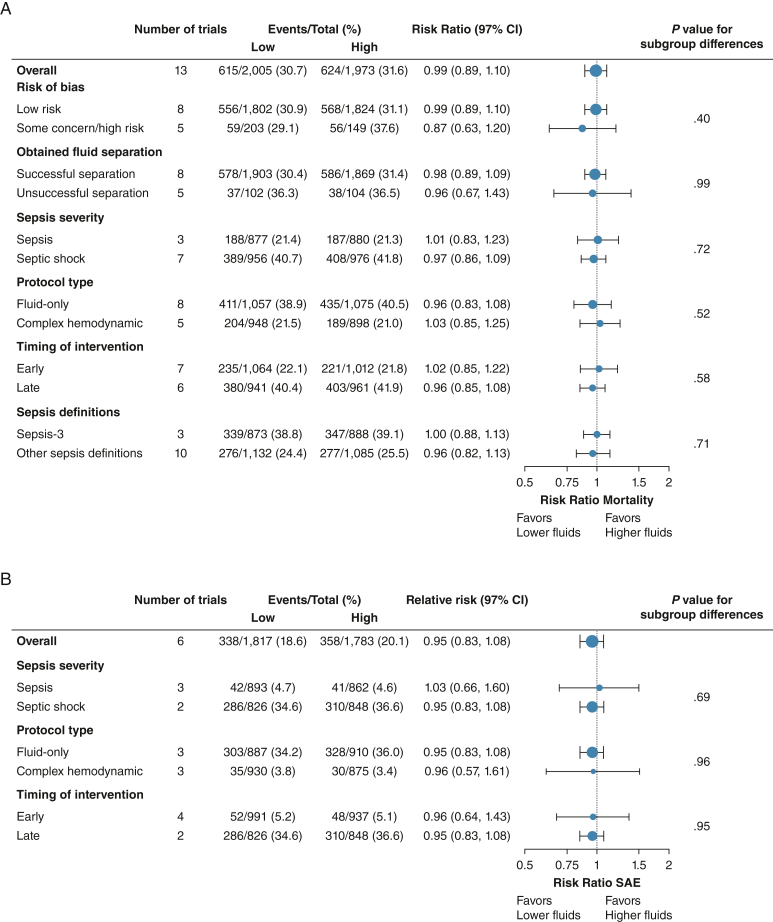
A, B, Subgroup analyses of coprimary outcomes. A, Relative risks with 97% CIs are shown for the all-cause mortality with lower vs higher IV fluid volumes among all the patients and the five predefined subgroups and one post hoc subgroup on the Sepsis-3 definition vs other definitions. B, Relative risks with 97% CIs for SAEs with lower vs higher IV fluid volumes among all the patients and three predefined subgroups. SAE = serious adverse event.

**Figure 4:**
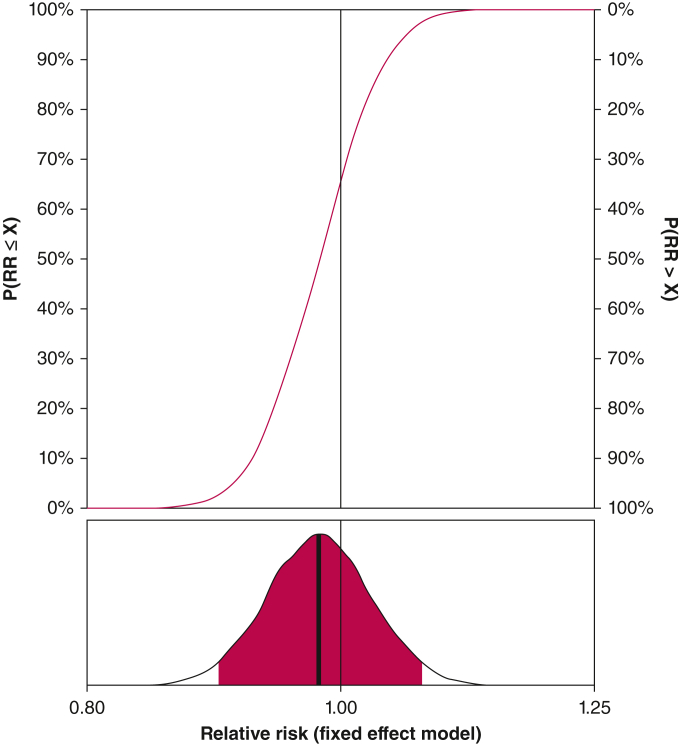
Bayesian analysis of all-cause mortality. Full posterior probability distribution for the treatment effect on all-cause mortality from the Bayesian analysis in a fixed effect model. We used a normally distributed weakly informative prior centered on no difference for the treatment effect (mean ± SD, 0 ± 1). Sensitivity analyses using different priors are reported in e-Appendix 6. The plot displays the relative difference (RR). An RR < 1 favors lower fluid volumes, whereas an RR > 1 favors higher fluid volumes. The upper subplot displays the cumulative posterior distribution, and therefore displays the probabilities (vertical axes) of various effect sizes (horizontal axis). The lower subplots display the entire posterior distribution, with the bold, vertical line indicating the median value (used as the point estimate) and the area highlighted in red indicating the percentile-based 95% credible interval. The vertical black line represents exactly no difference. The probability of any benefit (ie, RR < 1.00) with lower IV fluid volumes was 66.1%, whereas the probability of effect sizes smaller than an RR reduction of 15% (or the opposite RR increase) with lower IV fluid volumes was > 99.9%. The probability of an RR reduction of at least 15% was 0.1%, whereas the probability of the corresponding RR increase (ie, RR ≥ 1.18) was < 0.1%. RR = risk ratio

All 13 trials (n = 3,978) reported data on mortality,^10^, ^11^, ^12^, ^13^^,^^19^, ^20^, ^21^, ^22^, ^23^, ^24^, ^25^, ^26^^,^^35^ of which eight had low risk of bias (n = 3,626).^10^^,^^12^^,^^13^^,^^20^, ^21^, ^22^, ^23^^,^^25^ Meta-analysis of the eight trials showed an RR of 0.99 (fixed effect model; 97% CI, 0.89-1.10; *P* = .89) (Fig 2A) for the difference in all-cause mortality between lower vs higher fluid volumes. The TSA highlighted that 83% of the RIS of 4,380 patients was accrued (TSA-adjusted CI, 0.89-1.11) (Fig 2B), with the cumulative *z* curve reaching the area of futility. Therefore, an RR reduction of 15% is unlikely. This was consistent in the meta-analysis of all trials (n = 3,978) (fixed effect model RR, 0.98; 97% CI, 0.89-1.08; *P* = .69; TSA-adjusted CI, 0.89-1.08) (e-Appendix 6, Fig 2A). The certainty of evidence was moderate (Table 2). The subgroup analyses and sensitivity analyses for missing data were consistent with the primary estimates (e-Tables 7-9, Fig 3). The full posterior probability distribution for the treatment effect for all trials is presented in Figure 4 (details in e-Appendix 6 and e-Figs 1-5). In the primary Bayesian analysis of mortality for all trials, the RR was 0.98 (fixed effect model; 95% percentile-based credible interval, 0.90-1.07). The probability of any benefit (ie, RR < 1.00) with lower IV fluid volumes was 66.1%, whereas the probability of effect sizes smaller than an RR reduction of 15% (or the opposite RR increase) with lower IV fluid volumes was > 99.9%. The probability of an RR reduction of at least 15% was 0.1%, whereas the probability of the corresponding RR increase (ie, RR ≥ 1.18) was < 0.1%.

**Table 2:** GRADE Evaluation of the Certainty of Evidence (Low Risk of Bias Trials Only)

Certainty Assessment												
No. of Studies	Study Design	RoB	Certainty	Importance	Imprecision	Other Considerations	Lower Fluid Volumes	Higher Fluid Volumes	RR Effect (97% or 99% CI)	Absolute Effect (97% CI or 99% CI)		
All-cause mortality												
8	Randomized trials	Not serious	Not serious	Not serious	Serious^a^	None	556/1,802 (30.9)	568/1,824 (31.1)	0.99 (0.89-1.10)	3 fewer per 1.000 (from 34 fewer to 31 more)	Moderate	Critical
SAEs (highest proportion) - no low RoB trials												
6	Randomized trials	Serious^b^	Not serious	Not serious	Serious^c^	None	338/1,817 (18.6)	358/1,783 (20.1)	0.95 (0.83-1.07)	10 fewer per 1.000 (from 34 fewer to 14 more)	Low	Critical
Health-related quality of life												
0	Randomized trials	...	...	...	...	...	0/0 (0)	0/0 (0)	Not estimable	...	...	Critical
Duration of MV, d												
4	Randomized trials	Not serious	Not serious	Not serious	Not serious^d^	None	1,653	1,672	...	Mean difference**,** 0.11 days lower (0.5 lower to 0.28 higher)	High	Important
Ventilator-free days												
5	Randomized trials	Not serious	Not serious	Not serious	Serious^e^	None	1,667	1,687	...	Mean difference**,** 0.11 days lower (1.89 lower to 1.67 higher)	Moderate	Important
Duration of vasopressor or inotropes, h												
3	Randomized trials	Not serious	Not serious	Not serious	Not serious^f^	None	1,583	1,603	...	Mean difference**,** 0 h (0.33 lower to 0.33 higher)	High	Important
Vasopressor-free days												
3	Randomized trials	Not serious	Not serious	Not serious	Serious^g^	None	1,582	1,603	...	Mean difference**,** 0.43 d higher (0.68 lower to 1.53 higher)	Moderate	Important
Use of RRT												
6^h^	Randomized trials	Not serious	Not serious	Not serious	Serious^i^	None	203/1,662 (12.2)	206/1,681 (12.3)	1.01 (0.80-1.26)	1 more per 1.000 (from 25 fewer to 32 more)	Moderate	Important
Duration of RRT, d												
3^j^	Randomized trials	Not serious	Not serious	Not serious	Not serious^k^	None	866	887	...	Mean difference**,** 0.17 d lower (0.7 lower to 0.37 higher)	High	Important
RRT-free days												
5	Randomized trials	Not serious	Not serious	Not serious	Serious^l^	None	1,631	1,654	...	Mean difference**,** 0.46 d higher (0.68 lower to 1.61 higher)	Moderate	Important
Incidence of AKI												
2	Randomized trials	Not serious	Not serious	Not serious	Serious^m^	None	182/811 (22.4)	199/834 (23.9)	0.94 (0.75-1.19)	14 fewer per 1.000 (from 60 fewer to 45 more)	Moderate	Important
Use of blood products, units												
3	Randomized trials	Not serious	Not serious	Not serious	Not serious^n^	None	891	914	...	Mean difference, 0 units (0.1 lower to 0.1 higher)	High	Important
ICU length of stay, d												
6	Randomized trials	Not serious	Not serious	Not serious	Not serious^o^	None	965	982	...	Mean difference, 0.33 d lower (0.99 lower to 0.33 higher)	High	Important
Hospital length of stay, d												
3	Randomized trials	Not serious	Not serious	Not serious	Serious^p^	None	866	888	...	Mean difference, 0.78 d higher (0.73 lower to 2.28 higher)	Moderate	Important

Values are No. of patients, No. of patients (%), or as otherwise indicated. AKI = acute kidney injury; MV = mechanical ventilation; RIS = required information size; RoB = risk of bias; RR = risk ratio; RRT = renal replacement therapy; SAE = serious adverse event; TSA = trial sequential analysis.

a TSA highlighted that 83% of RIS was reached. The area of futility to detect a predefined relative risk reduction of 15% was reached; however, the CI overlaps no effect, and we cannot exclude important benefit or harm. Adjusted CI was 0.89 to 1.11 (from 34 fewer to 34 more).

b ROBs were adjudicated as some concerns for all six trials based measurement of the outcome, deviations from intended intervention, or selection of the reported result.

c TSA highlighted that 46% of RIS was reached. Adjusted CI was 0.78 to 1.15 (from 44 fewer to 30 more).

d TSA highlighted that the boundary for futility crossed and RIS was reached, thus the adjusted CI is identical to the unadjusted. Thus, we can exclude a predefined mean difference of 1 day.

e TSA highlighted that 34% of RIS was reached. Adjusted CI was −1.86 to 2.27.

f TSA highlighted that the boundary for futility crossed and RIS was reached; therefore, the adjusted CI is identical to the unadjusted. Therefore, we can exclude a predefined mean difference of 24 h.

g TSA highlighted that 38% of RIS was reached. Adjusted CI was −1.41 to 2.27.

h Jessen et al had zero events and was not included in meta-analysis or TSA.

i TSA highlighted that 19% of RIS was reached. Adjusted CI was 0.49 to 2.04 (from 62 fewer to 127 more).

j Jessen et al had zero events and was not included in meta-analysis or TSA. The remaining three trials are low RoB trials.

k TSA highlighted that the boundary for futility was crossed and RIS was reached; therefore, the adjusted CI is identical to the unadjusted. Therefore, we can exclude a predefined mean difference of 1 d.

l TSA highlighted that 35% of RIS was reached. Adjusted CI was −1.50 to 2.43.

m TSA highlighted that 22% of RIS was reached. Adjusted CI was 0.46 to 1.93.

n TSA highlighted that the boundary for futility was crossed and RIS was reached; therefore, the adjusted CI is identical to the unadjusted. Therefore, we can exclude a predefined mean difference of 1 unit of blood product.

o TSA highlighted 87% of RIS was reached, and the boundary for futility was crossed based on a predefined MD of 1 d. Adjusted CI was −1.07 to 0.40.

p TSA highlighted that 18% of RIS was reached. Adjusted CI was −4.60 to 6.15.

#### SAEs

**Figure 5:**
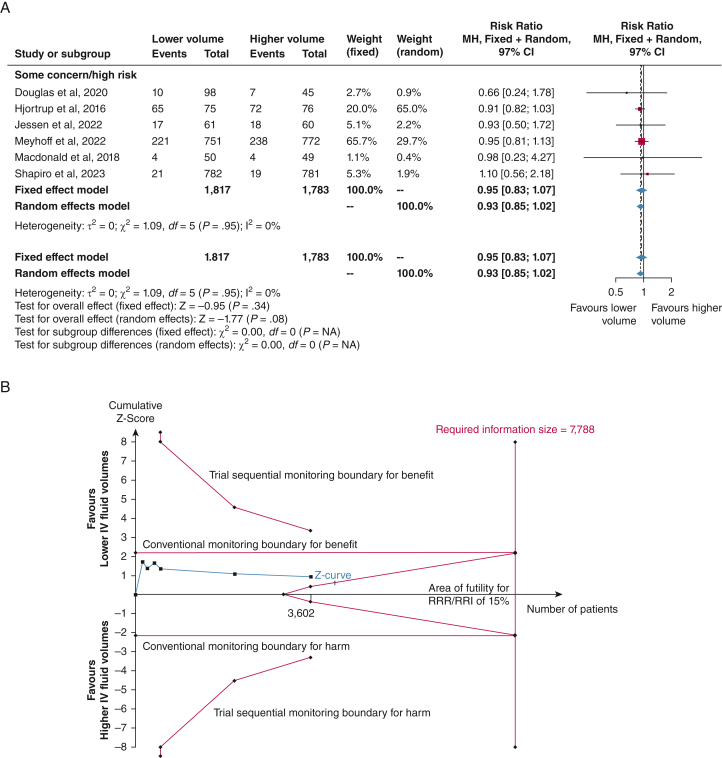
A, B, Serious adverse events. A, Meta-analysis of the highest proportion of serious adverse events (as defined in the original trial) in six trials, all with some concerns in the risk of bias adjudication. The conventional meta-analysis demonstrated no statistically significant difference in serious adverse events with lower vs higher fluid volumes (fixed effect model; relative risk, 0.95; 95% CI, 0.83-1.07; *I*^2^ = 0%). B, Trial sequential analysis (TSA) of the highest proportion of serious adverse events in six trials. We used a control event proportion of 20.1%, α of 3.3% (two-sided), β of 10% (power 90%), and an a priori relative risk reduction of 15% in the analysis. The TSA-adjusted CI in the fixed effect model was 0.78 to 1.15, with a diversity D^2^ of 0%. The blue cumulative z curve did not cross the conventional monitoring boundaries for benefit, harm, or futility; the TSA is therefore inconclusive. A total of 46% (n = 3,602) of the required information size of 7,788 patients was accrued. MH = Mantel-Haaenszel; RRI = relative risk increase; RRR = relative risk reduction

Six trials had defined and reported SAEs (n = 3,600)^10^, ^11^, ^12^, ^13^^,^^20^^,^^23^ and showed an RR of 0.95 (fixed effect model; 97% CI, 0.83-1.07; *P* = .28) (Fig 5A) for the difference in SAEs between lower vs higher fluid volumes. The TSA showed that 46% of the RIS of 7,788 patients was accrued (TSA-adjusted CI, 0.78-1.15) (Fig 5B). The certainty of evidence was low because of imprecision and risk of bias because all trials were adjudicated as having some concerns (Table 2). The subgroup analyses and sensitivity analyses for missing data were consistent with the primary estimates (e-Tables 7-9). Analyses of the highest proportion of SAEs including mortality based on ICH-GCP and cumulated SAEs are provided in e-Appendix 6.

#### Health-Related Quality of Life

No trials reported data on health-related quality of life.

### Secondary Outcomes

#### Mechanical Ventilation

Seven trials reported duration of mechanical ventilation, and six trials allowed meta-analysis (n = 3,481).^10^, ^11^, ^12^^,^^19^, ^20^, ^21^^,^^23^ Meta-analysis of the four trials with low risk of bias (n = 3,325) showed no statistically significant difference between lower vs higher fluid volumes (fixed effect model; mean difference, −0.11 days; 99% CI, −0.50 to 0.28; *P* = .46).^10^^,^^12^^,^^20^^,^^23^ The TSA highlighted that more than the RIS of 1,329 patients was accrued with the cumulative *z* curve reaching the area of futility. Therefore, we can exclude a predefined MD of 1 day, high certainty of evidence. Meta-analysis of all trials detected a significant heterogeneity (*I*^2^ = 61%; *D*^2^ = 93%), partially explained by risk of bias. However, results were still consistent with those of the primary analysis. Further details are provided in e-Appendixes 7 and 7.1.

Ten trials reported ventilator-free days, of which data from eight trials could be included in the meta-analysis (n = 3,476).^10^^,^^12^^,^^19^, ^20^, ^21^^,^^23^, ^24^, ^25^, ^26^^,^^35^ Meta-analysis of five trials with low risk of bias (n = 3,354) showed no statistically significant difference between lower vs higher fluid volumes (random effects model; mean difference, −0.11 days; 99% CI, −1.89 to 1.67; *P* = .87). Thirty-four percent of the RIS of 9,916 patients was accrued (TSA-adjusted CI, −1.86 to 2.27) (e-Appendixes 7, 7.2). The certainty of evidence was moderate because of imprecision.

#### Circulatory Support

Seven trials reported duration of vasopressor or inotropes, and six of those trials allowed meta-analysis (n = 3,460).^10^, ^11^, ^12^^,^^19^^,^^21^^,^^23^^,^^26^ No statistically significant difference between lower vs higher fluid volumes was observed in the meta-analysis of three trials with low risk of bias (fixed effect model; mean difference, 0.00 h; 99% CI, −0.33 to 0.33; *P* > .99). TSA highlighted that more than the RIS had been accrued, and the cumulative *z* curve crossed the boundary of futility (e-Appendixes 7, 7.3). Therefore, a predefined MD of 24 h is unlikely with high certainty of evidence.

Vasopressor-free days were reported in six trials, of which data from five trials allowed for meta-analysis (n = 3,324).^10^^,^^12^^,^^19^^,^^21^^,^^23^^,^^25^ Meta-analysis of three trials with low risk of bias showed no statistically significant difference between lower vs higher fluid volumes (fixed effect model; mean difference, 0.43 days; 99% CI, −0.68 to 1.53). In total, 38% of the RIS of 8,378 patients was accrued (TSA adjusted CI, −1.41 to 2.27) (e-Appendixes 7, 7.4). The certainty of evidence was moderate because of imprecision.

#### RRT

In nine trials reporting the use of RRT (n = 3,645),^10^, ^11^, ^12^, ^13^^,^^19^, ^20^, ^21^^,^^23^^,^^25^ six trials with low risk of bias (n = 3,343) showed an RR of 1.01 (fixed effect model; 99% CI, 0.80-1.26) for the difference between lower vs higher fluid volumes. TSA displayed that 19% of the RIS of 17,019 patients was accrued (TSA adjusted CI, 0.49-2.04) (e-Appendixes 7, 7.5). The certainty of evidence was moderate because of imprecision.

Duration of RRT was reported in three trials (n = 1,753),^12^^,^^13^^,^^23^ which were all at low risk of bias. The meta-analysis showed no statistically significant difference between lower vs higher fluid volumes (fixed effect model; mean difference, −0.17 days; 99% CI, −0.70 to 0.37). The TSA highlighted that the RIS of 1,013 patients was accrued, and the cumulative *z* curve crossed the boundary of futility. Therefore, a predefined MD of 1 day is unlikely (e-Appendixes 7, 7.6). The certainty of evidence was high.

Five trials reported RRT-free days (n = 3,285),^10^^,^^12^^,^^20^^,^^23^^,^^25^ and all were classified as trials with low risk of bias. We did not observe a statistically significant difference between lower vs higher fluid volumes (fixed effect model; mean difference, 0.46; 99% CI, −0.68 to 1.61). The TSA highlighted that 35% of the RIS of 9,283 patients was accrued (TSA adjusted CI, −1.50 to 2.43). The certainty of evidence was moderate because of imprecision. (e-Appendixes 7, 7.7)

#### AKI

Three trials reported the incidence of AKI as part of SAEs (n = 1,754).^12^^,^^13^^,^^21^ Meta-analysis of two trials with low risk of bias showed an RR of 0.94 (fixed effect model; 99% CI, 0.75-1.19) for the difference between lower vs higher fluid volumes. The TSA displayed that 22% of the RIS of 7,633 patients was accrued (TSA-adjusted CI, 0.46-1.93). The certainty of evidence was judged to be moderate because of imprecision.

Three trials either reported the Acute Kidney Injury Network score or Kidney Disease Improving Global Outcomes score.^20^^,^^23^^,^^25^ However, these were not included in the meta-analysis because of the reported worsening, and not incidence, of AKI.

### Exploratory Outcomes

Meta-analysis of all three exploratory outcomes showed no statistically significant difference between lower vs higher fluid volumes. Further details are provided in e-Appendix 8. The TSA of three trials with low risk of bias reporting the use of blood products showed that the RIS (n = 47) was reached within the first trial, and the cumulative *z* curve crossed the boundary of futility. Therefore, a mean difference of 1 unit of blood product is unlikely with high certainty of evidence (e-Appendixes 8, 8.1). The TSA of six trials with low risk of bias reporting ICU length of stay showed the cumulative *z* curve crossed the boundary of futility (TSA adjusted CI, −1.07 to 0.40), and 87% of the RIS was accrued. Therefore, a mean difference of 1 day is unlikely with moderate certainty of evidence (e-Appendixes 8, 8.2). Eighteen percent of the RIS was accrued for hospital length of stay (TSA adjusted CI, −4.60 to 6.15) (e-Appendixes 8, 8.3). The blue cumulative *z* curve did not cross the conventional monitoring boundaries for benefit, harm, or futility; therefore, the TSA is inconclusive. The certainty of evidence was moderate.

### Subgroup Analyses and Sensitivity Analyses

The subgroup analyses and sensitivity analyses for missing data for the secondary and exploratory outcomes were consistent with the primary analyses (e-Appendix 9, e-Tables 7-9). The subgroup analysis on medical vs surgical treatment was not conducted because these details could not be extracted separately.

## Discussion

In this systematic review of lower vs higher IV fluid volumes in adults with sepsis, we found that an RR reduction ≥ 15% on mortality is unlikely. Furthermore, the probability of effect sizes smaller than an RR reduction of 15% (or the opposite RR increase) with lower IV fluid volumes was > 99.9%. The occurrence of SAEs was predefined and reported in only six of the 13 trials, and the definitions varied across trials. We observed no statistically significant difference in SAEs with low certainty of evidence. No trials reported health-related quality of life. We could exclude a difference of 1 day in duration of mechanical, vasopressor, and RRT.

### Perspectives

IV fluids are used in daily clinical practice in patients with sepsis. We can with moderate certainty rule out substantial effects on mortality with lower vs higher IV fluids among adults with sepsis. This could partially be caused by clinical practice having changed over the last 10 years leading to use of lower volumes of fluids. Furthermore, the results might be blurred because most patients received IV fluids before inclusion in most trials and only three trials included patients in the ED. Of note, the effects of the initial resuscitation fluid is assessed in ongoing trials.^39^^,^^46^ Importantly, our finding does not exclude small but important effects on mortality, which would be clinically relevant considering the global burden of sepsis. Lower IV fluid volumes may result in little to no difference in SAEs compared with higher IV fluid volumes, but the interpretation is limited by imprecision in effect estimates, which do not exclude potential benefit or harm. All trials reporting on SAEs were adjudicated with some concern of risk of bias. Importantly, SAEs are likely to be underreported and definitions across trials differed. Therefore, our knowledge is still limited. We could exclude a mean difference of 1 day in duration of mechanical ventilation, vasopressor, or RRT. Therefore, IV fluid strategies as tested in the included trials may be safely used.

Future objectives within this research field would be to assess a smaller, but still clinically relevant, difference on mortality and in other patient-important outcomes, including health-related quality of life and cognitive function. Furthermore, a more systematic and pragmatic approach to the reporting of SAEs is highly warranted.

### Strengths and Limitations

We updated the previously conducted review because inclusion of the latest four trials enrolling 3,385 patients substantially increased the quantity and certainty of evidence. In addition to the meta-analyses, we conducted extensive analyses including TSA to estimate the RISs and conclusiveness of the evidence according to predefined effect sizes for each outcome. We also conducted Bayesian analyses of all-cause mortality to nuance the results. Additionally, we adhered to the published protocol and preplanned statistical analysis plan^14^ and the recommendations by the Cochrane Collaboration and GRADE.^15^^,^^16^ Finally, we reported the paper according to the Preferred Reporting Items for Systematic Reviews and Meta-Analyses statement.^15^, ^16^, ^17^

Our review also has limitations. First, the separation of fluid volumes between the groups was unsuccessful in five trials, which may have contributed to the finding of no difference in the outcomes. However, the subgroup analyses of trials with separation of fluid volumes were in line with the primary analysis. Second, the definitions of sepsis and septic shock were not consistent across the trials, which may influence mortality because this varies according to the definitions used.^47^ However, the post hoc meta-analysis of trials using the Sepsis-3 definition^43^ vs earlier sepsis definitions was consistent with the primary analysis of all-cause mortality. Third, heterogeneity in the patient population and interventions may have been present because seven trials included patients in the ICU, three trials included patients from the ED, and three trials included patients from both settings. Furthermore, some fluid trials used more complex protocols including a bundle of interventions. We allowed all types of IV fluid, and one trial used hydroxyethyl starch with well-known harms in patients with sepsis,^48^ but neither the treatment effects nor the potentially important dose dependencies were accounted for. Taken together, heterogeneity in patient populations and interventions is likely, which might have blurred any effects. The subgroup analyses assessing sepsis vs septic shock and simple vs complex protocols were consistent with the primary analyses. Fourth, SAE is a challenging outcome because definitions vary across trials and SAEs are likely underreported. Therefore, we analyzed data from all trials by including mortality and cumulated SAEs, as done in the first version of the review.^14^ These were consistent with the primary analysis of SAEs. Fifth, the TSAs are conducted based on a predefined RR reduction or mean difference; therefore, smaller reductions or difference cannot be excluded. Finally, in the individual days without life support outcomes and exploratory outcomes, data were reported with wide SDs, which is likely caused by the typically nonnormal distributions of these outcomes. This could influence the models used for these outcomes because they assume that data are normally distributed. Hence, results for these outcomes should be interpreted with caution.

## Interpretation

In this systematic review of adult patients with sepsis, lower IV fluid volumes probably result in little to no difference in all-cause mortality compared with higher IV fluid volumes, but the interpretation is limited by imprecision in the effect estimate, which does not exclude potential benefit or harm. Similarly, the evidence suggests that lower IV fluid volumes result in little to no difference in SAEs. However, the certainty of evidence was downgraded because of imprecision and risk of bias because all trials reporting on SAEs were adjudicated as having some concerns. No trials reported on health-related quality of life.

## Funding/Support

P. S. receives funding from the Research Council of Rigshospitalet, Copenhagen, Denmark; and the Ehrenreich Foundation. The Department of Intensive Care, Rigshospitalet receives support for research projects from Fresenius Kabi, Pfizer (CP231465), and Sygeforsikringen ‘danmark’ (2020-0320) and conducts contract research for AM-Pharma.

## Financial/Nonfinancial Disclosures

The authors have reported to *CHEST* the following: The authors are involved in a multicenter international randomized clinical trial (The Conservative vs Liberal Approach to fluid therapy of Septic Shock in Intensive Care [CLASSIC]; Clinicaltrials.gov identifier NCT03668236) included in the review and funded by the 10.13039/501100009708Novo Nordisk Foundation (NNF17OC0028608) and by the Sofus Friis Foundation. P. B. H., M. H. M., J. W., and A. P. were involved in the planning and conducting of the CLASSIC pilot randomized clinical trial. M. K. J., T. S. M., M. C., and P. B. H. are the first authors in four of the trials included in the present review.

## Supplementary Data

e-Online Data

Audio
