# Supplementary material for: Lower vs Higher Fluid Volumes in Adult Patients With Sepsis: An Updated Systematic Review With Meta-Analysis and Trial Sequential Analysis
Source: Chest. 2023 May 2;164(4):892–912. doi: 10.1016/j.chest.2023.04.036 (PMC10567931; doi:10.1016/j.chest.2023.04.036)
Supplement: e-Online Data [file mmc1.docx]

**Electronic Supplementary Material**

Lower vs Higher Fluid Volumes in Adult Patients with Sepsis -

An Updated Systematic Review with Meta-Analysis and Trial Sequential Analysis

*Praleene Sivapalan, Karen L. Ellekjaer, Marie K. Jessen, Tine S. Meyhoff, Maria Cronhjort, Peter B. Hjortrup, Jørn Wetterslev, Anders Granholm, Morten H. Møller, and Anders Perner.*

*Document Outline*

[1. e-Appendix 1. Protocol specifications and deviations 8](#_Toc133406446)

[2. e-Appendix 2. PRISMA checklist 9](#_Toc133406447)

[3. e-Appendix 3. Full search strategy 12](#_Toc133406448)

[4. e-Appendix 4. Data extraction template 16](#_Toc133406449)

[5. e-Appendix 5 Assessment of risk of random errors in meta-analyses 18](#_Toc133406450)

[6. e-Appendix 6 Analyses of primary outcomes 19](#_Toc133406451)

[7. e-Appendix 7 Analyses of secondary outcomes 35](#_Toc133406452)

[8. e-Appendix 8 Analyses of exploratory outcomes 47](#_Toc133406453)

[9. e-Appendix 9 Subgroup analyses 53](#_Toc133406454)

[10. e-Table 5 Fluid Data in the Included Trials 74](#_Toc133406455)

[11. e-Table 6 Overview of Risk of Bias (RoB2) adjudications for all outcomes 76](#_Toc133406456)

[12. e-Table 7 Overview of all primary meta-analyses 84](#_Toc133406457)

[13. e-Table 8 Overview of all subgroup analyses 85](#_Toc133406458)

[14. e-Table 9 Sensitivity analyses for missing data across all outcomes 90](#_Toc133406459)

[15. e-Table 10 Full GRADE Evaluation of the Certainty of Evidence 91](#_Toc133406460)

[15. References 97](#_Toc133406461)

**Table of contents**

[1. e-Appendix 1. Protocol specifications and deviations 7](#_Toc133406478)

[2. e-Appendix 2. PRISMA checklist 8](#_Toc133406479)

[3. e-Appendix 3. Full search strategy 11](#_Toc133406480)

[4. e-Appendix 4. Data extraction template 15](#_Toc133406481)

[5. e-Appendix 5 Assessment of risk of random errors in meta-analyses 17](#_Toc133406482)

[6. e-Appendix 6 Analyses of primary outcomes 18](#_Toc133406483)

[6.1 All-cause mortality - meta-analysis and TSA 18](#_Toc133406484)

[6.1.1 Meta-analysis and forest plot of all-cause mortality 18](#_Toc133406485)

[6.1.2 TSA of all-cause mortality (low RoB trials only) 18](#_Toc133406486)

[6.1.3 TSA of all-cause mortality (all trials) 19](#_Toc133406487)

[6.1.4 Funnel plot of all-cause mortality (all trials) 19](#_Toc133406488)

[6.1.5 Bayesian secondary analyses of all-cause mortality (all trials) 20](#_Toc133406489)

[6.1.6 Bayesian sensitivity analyses of all-cause mortality 21](#_Toc133406490)

[e-Figure 1 Bayesian analysis of all-cause mortality (random effects model) 21](#_Toc133406491)

[e-Figure 2 Bayesian sensitivity analysis of all-cause mortality (sceptic prior, fixed effect model) 22](#_Toc133406492)

[e-Figure 3 Bayesian sensitivity analysis of all-cause mortality (sceptic prior, random effects model) 23](#_Toc133406493)

[e-Figure 4 Bayesian sensitivity analysis of all-cause mortality (more sceptic prior, fixed effects model) 24](#_Toc133406494)

[e-Figure 5 Bayesian sensitivity analysis of all-cause mortality (more sceptic prior, random effects model) 25](#_Toc133406495)

[6.2 Serious adverse events - meta-analysis and TSA 26](#_Toc133406496)

[6.2.1 e-Table 1 Highest proportion of SAEs (as defined in the original trial) 26](#_Toc133406497)

[6.2.1a Meta-analysis and forest plot of highest proportion of SAE/SAR 27](#_Toc133406498)

[6.2.1b TSA of highest proportion of SAE/SAR 27](#_Toc133406499)

[6.2.2 e-Table 2 Highest proportion of SAE including mortality (based on ICH-GCP categorization) 28](#_Toc133406500)

[6.2.2a Meta-analysis and forest plot of highest proportion of SAE including mortality 29](#_Toc133406501)

[6.2.2b TSA of highest proportion of SAE including mortality 29](#_Toc133406502)

[6.2.c Funnel plot of highest proportion of SAE including mortality 30](#_Toc133406503)

[6.2.3 e-Table 3 Serious adverse events (cumulated) 30](#_Toc133406504)

[6.2.3 e-Table 4 Serious adverse events (cumulated) - specified 31](#_Toc133406505)

[6.2.3 a Meta-analysis and forest plot of serious adverse events (cumulated) 32](#_Toc133406506)

[6.2.3 b TSA of serious adverse events (cumulated) 33](#_Toc133406507)

[6.2.3 c Funnel plot of serious adverse events (cumulated) 33](#_Toc133406508)

[7. e-Appendix 7 Analyses of secondary outcomes 34](#_Toc133406509)

[7.1 Duration of mechanical ventilation - meta-analysis and TSA 34](#_Toc133406510)

[7.1.1 Meta-analysis and forest plot of duration of mechanical ventilation 34](#_Toc133406511)

[7.1.2 TSA of duration of mechanical ventilation (low RoB trials only) 34](#_Toc133406512)

[7.1.3 TSA of duration of mechanical ventilation (all trials) 35](#_Toc133406513)

[7.2 Ventilator-free days - meta-analysis and TSA 35](#_Toc133406514)

[7.2.1 Meta-analysis and forest plot of ventilator-free days 35](#_Toc133406515)

[7.2.1 TSA of ventilator-free days (low RoB trials only) 36](#_Toc133406516)

[7.2.2 TSA of ventilator-free days (all trials) 36](#_Toc133406517)

[7.3 Duration of vasopressor or inotropes-meta-analysis and TSA 37](#_Toc133406518)

[7.3.1 Meta-analysis and forest plot of duration of vasopressor or inotropes 37](#_Toc133406519)

[7.3.2 TSA of duration of vasopressor or inotropes (low RoB trials only) 37](#_Toc133406520)

[7.3.3 TSA of duration of vasopressor or inotropes (all trials) 38](#_Toc133406521)

[7.4 Vasopressor-free days - meta-analysis and TSA 38](#_Toc133406522)

[7.4.1 Meta-analysis and forest plot of vasopressor-free days 38](#_Toc133406523)

[7.4.2 TSA of vasopressor-free days (low RoB trials only) 39](#_Toc133406524)

[7.4.3 TSA of vasopressor-free days (all trials) 39](#_Toc133406525)

[7.5 Use of renal replacement therapy - meta-analysis and TSA 40](#_Toc133406526)

[7.5.1 Meta-analysis and forest plot of use of renal replacement therapy 40](#_Toc133406527)

[7.5.2 TSA of use of renal replacement therapy (low RoB trials only) 40](#_Toc133406528)

[7.5.3 TSA of use of renal replacement therapy (all trials) 41](#_Toc133406529)

[7.6 Duration of renal replacement therapy – meta-analysis and TSA 41](#_Toc133406530)

[7.6.1 Meta-analysis and forest plot of duration of renal replacement therapy 41](#_Toc133406531)

[7.6.2 TSA of duration of renal replacement therapy (two low RoB trials) 42](#_Toc133406532)

[7.7 Renal replacement therapy-free days - meta-analysis and TSA 42](#_Toc133406533)

[7.7.1 Meta-analysis and forest plot of renal replacement therapy-free days (all trials - all low RoB) 42](#_Toc133406534)

[7.7.2 TSA of renal replacement therapy-free days (all trials - all low RoB) 43](#_Toc133406535)

[7.6 Incidence of acute kidney injury - meta-analysis and TSA 43](#_Toc133406536)

[7.6.1 Meta-analysis and forest plot of incidence of acute kidney injury 43](#_Toc133406537)

[7.6.2 TSA of incidence of acute kidney injury (low RoB trials only) 44](#_Toc133406538)

[7.6.3 TSA of incidence of acute kidney injury (all trials) 44](#_Toc133406539)

[8. e-Appendix 8 Analyses of exploratory outcomes 46](#_Toc133406540)

[8.1 Use of blood products - meta-analysis and TSA 46](#_Toc133406541)

[8.1.1 Meta-analysis and forest plot of the use of blood products 46](#_Toc133406542)

[8.1.1 TSA of the use of blood products (low RoB trials only) 46](#_Toc133406543)

[8.1.2 TSA of the use of blood products (all trials) 47](#_Toc133406544)

[8.2 ICU length of stay - meta-analysis and TSA 47](#_Toc133406545)

[8.2.1 Meta-analysis and forest plot of ICU length of stay 47](#_Toc133406546)

[8.2.2 TSA of ICU length of stay (low RoB trials only) 48](#_Toc133406547)

[8.2.3 TSA of ICU length of stay (all trials) 48](#_Toc133406548)

[8.3 Hospital length of stay - meta-analysis and TSA 50](#_Toc133406549)

[8.3.1 Meta-analysis and forest plot of hospital length of stay 50](#_Toc133406550)

[8.3.2 TSA of hospital length of stay (low RoB trials only) 50](#_Toc133406551)

[8.3.3 TSA of hospital length of stay (all trials) 51](#_Toc133406552)

[9. e-Appendix 9 Subgroup analyses 52](#_Toc133406553)

[9.1 Pre-planned subgroups and hypothesised direction of effect^2^ 52](#_Toc133406554)

[9.3. Forest plots for all subgroup analyses 53](#_Toc133406555)

[9.3.1 Primary outcomes 53](#_Toc133406556)

[9.3.1 a) Subgroup analyses of all-cause mortality 53](#_Toc133406557)

[i. All-cause mortality: Overall low vs some concern or high risk of bias 53](#_Toc133406558)

[ii. All-cause mortality: Successful vs unsuccessful separation in fluid volumes 53](#_Toc133406559)

[iii. All-cause mortality: Patients with sepsis vs septic shock 54](#_Toc133406560)

[iv. All-cause mortality: Fluid-only interventions vs a complex hemodynamic protocol 54](#_Toc133406561)

[v. All-cause mortality: Sepsis-3 definition vs other sepsis definitions (post-hoc subgroup analysis) 55](#_Toc133406562)

[9.3.1 b) Subgroup analyses of serious adverse events 55](#_Toc133406563)

[i. Serious adverse events: Patients with sepsis vs septic shock 55](#_Toc133406564)

[vi. All-cause mortality: Early vs later resuscitation phase of sepsis 56](#_Toc133406565)

[ii. Serious adverse events: Fluid-only interventions vs a complex hemodynamic protocol 56](#_Toc133406566)

[iii. Serious adverse events: Early vs later resuscitation phase of sepsis 57](#_Toc133406567)

[9.3.2 Secondary outcomes 57](#_Toc133406568)

[9.3.2 a) Subgroup analyses of duration of mechanical ventilation 57](#_Toc133406569)

[i. Duration of mechanical ventilation: Overall low vs some concern or high risk of bias 57](#_Toc133406570)

[ii. Duration of mechanical ventilation: Patients with sepsis vs septic shock 57](#_Toc133406571)

[iii. Duration of mechanical ventilation: Fluid-only interventions vs a complex hemodynamic protocol 58](#_Toc133406572)

[iv. Duration of mechanical ventilation: Early vs later resuscitation phase of sepsis 58](#_Toc133406573)

[9.3.2 b) Subgroup analyses of ventilator-free days 58](#_Toc133406574)

[i. Ventilator-free days: Overall low vs some concern or high risk of bias 58](#_Toc133406575)

[ii. Ventilator-free days: Successful vs unsuccessful separation in fluid volumes 59](#_Toc133406576)

[iii. Ventilator-free days: Patients with sepsis vs septic shock 59](#_Toc133406577)

[iv. Ventilator-free days: Fluid-only interventions vs a complex hemodynamic protocol 59](#_Toc133406578)

[v. Ventilator-free days: Early vs later resuscitation phase of sepsis 60](#_Toc133406579)

[9.3.2 c) Subgroup analyses of duration of vasopressor or inotropes 60](#_Toc133406580)

[i. Duration of vasopressor or inotropes: Overall low vs some concern or high risk of bias 60](#_Toc133406581)

[ii. Duration of vasopressor or inotropes: Patients with sepsis vs septic shock 60](#_Toc133406582)

[iii. Duration of vasopressor or inotropes: Fluid-only interventions vs a complex hemodynamic protocol 61](#_Toc133406583)

[iv. Duration of vasopressor or inotropes: Early vs later resuscitation phase 61](#_Toc133406584)

[9.3.2 d) Subgroup analyses of vasopressor-free days 61](#_Toc133406585)

[i. Vasopressor-free days: Overall low vs some concern or high risk of bias 61](#_Toc133406586)

[ii. Vasopressor-free days: Successful vs unsuccessful separation in fluid volumes 62](#_Toc133406587)

[iii. Vasopressor-free days: Patients with sepsis vs septic shock 62](#_Toc133406588)

[iv. Vasopressor-free days: Fluid-only interventions vs a complex hemodynamic protocol 62](#_Toc133406589)

[v. Vasopressor-free days: Early vs later resuscitation phase of sepsis 63](#_Toc133406590)

[9.3.2 e) Subgroup analyses of the use of renal replacement therapy 63](#_Toc133406591)

[i. Use of renal replacement therapy: Overall low vs some concern or high risk of bias 63](#_Toc133406592)

[ii. Use of renal replacement therapy: Successful vs unsuccessful separation in fluid volumes 63](#_Toc133406593)

[iii. Use of renal replacement therapy: Patients with sepsis vs septic shock 64](#_Toc133406594)

[iv. Use of renal replacement therapy: Fluid-only interventions vs a complex hemodynamic protocol 64](#_Toc133406595)

[v. Use of renal replacement therapy: Early vs later resuscitation phase of sepsis 64](#_Toc133406596)

[9.3.2 f) Subgroup analyses of the duration of renal replacement therapy (all trials low risk of bias trials) 65](#_Toc133406597)

[i. Duration of renal replacement therapy: Patients with sepsis vs septic shock 65](#_Toc133406598)

[ii. Duration of renal replacement therapy: Fluid-only interventions vs a complex hemodynamic protocol 65](#_Toc133406599)

[iii. Duration of renal replacement therapy: Early vs later resuscitation phase of sepsis 65](#_Toc133406600)

[9.3.2 g) Subgroup analyses of renal replacement therapy free days (all trials low risk of bias trials) 65](#_Toc133406601)

[i. Renal replacement therapy free days: Successful vs unsuccessful separation in fluid volumes 65](#_Toc133406602)

[ii. Renal replacement therapy free days: Patients with sepsis vs septic shock 66](#_Toc133406603)

[iii. Renal replacement therapy free days: Fluid-only interventions vs a complex hemodynamic protocol 66](#_Toc133406604)

[iv. Renal replacement therapy free days: Early vs later resuscitation phase of sepsis 66](#_Toc133406605)

[9.3.2 h) Subgroup analyses of incidence of acute kidney injury 67](#_Toc133406606)

[i. Incidence of acute kidney injury: Overall low vs some concern or high risk of bias 67](#_Toc133406607)

[ii. Incidence of acute kidney injury: Patients with sepsis vs septic shock 67](#_Toc133406608)

[iii. Incidence of acute kidney injury: early vs later resuscitation phase of sepsis 67](#_Toc133406609)

[9.3.3 Exploratory outcomes 68](#_Toc133406610)

[9.3.3 a) Subgroup analyses of the use of blood products 68](#_Toc133406611)

[i. Use of blood products: Overall low vs some concern or high risk of bias 68](#_Toc133406612)

[ii. Use of blood products: Successful vs unsuccessful separation in fluid volumes 68](#_Toc133406613)

[iii. Use of blood products: Patients with sepsis vs septic shock 68](#_Toc133406614)

[iv. Use of blood products: Fluid-only interventions vs a complex hemodynamic protocol 69](#_Toc133406615)

[v. Use of blood products: Early vs later resuscitation phase of sepsis 69](#_Toc133406616)

[9.3.3 b) Subgroup analyses of ICU length of stay 69](#_Toc133406617)

[i. ICU length of stay: Overall low vs some concern or high risk of bias 69](#_Toc133406618)

[ii. ICU length of stay: Successful vs unsuccessful separation in fluid volumes 70](#_Toc133406619)

[iii. ICU length of stay: Patients with sepsis vs septic shock 70](#_Toc133406620)

[iv. ICU length of stay: Fluid-only interventions vs a complex hemodynamic protocol 70](#_Toc133406621)

[v. ICU length of stay: Early vs later resuscitation phase of sepsis 71](#_Toc133406622)

[9.3.3 c) Subgroup analyses of hospital length of stay 71](#_Toc133406623)

[i. Hospital length of stay: Overall low vs some concern or high risk of bias 71](#_Toc133406624)

[ii. Hospital length of stay: Patients with sepsis vs septic shock 71](#_Toc133406625)

[iii. Hospital length of stay: Fluid-only interventions vs a complex hemodynamic protocol 72](#_Toc133406626)

[iv. Hospital length of stay: Early vs later resuscitation phase of sepsis 72](#_Toc133406627)

[10. e-Table 5 Fluid Data in the Included Trials 73](#_Toc133406628)

[11. e-Table 6 Overview of Risk of Bias (RoB2) adjudications for all outcomes 75](#_Toc133406629)

[e-Table 6.1 RoB2 adjudications for all-cause mortality 75](#_Toc133406630)

[e-Table 6.2 RoB2 adjudications for serious adverse events 76](#_Toc133406631)

[e-Table 6.3 RoB2 adjudications for duration of mechanical ventilation 77](#_Toc133406632)

[e-Table 6.4 RoB2 adjudications for ventilator-free days 77](#_Toc133406633)

[e-Table 6.5 RoB2 adjudications for duration of vasopressor or inotropes 78](#_Toc133406634)

[e-Table 6.6 RoB2 adjudications for vasopressor-free days 78](#_Toc133406635)

[e-Table 6.7 RoB2 adjudications for use of renal replacement therapy (RRT) 78](#_Toc133406636)

[e-Table 6.8 RoB2 adjudications for duration of RRT 79](#_Toc133406637)

[e-Table 6.9 RoB2 adjudications for renal replacement therapy-free days 79](#_Toc133406638)

[e-Table 6.10 RoB2 adjudications for incidence of acute kidney injury 80](#_Toc133406639)

[e-Table 6.11 RoB2 adjudications for blood products 80](#_Toc133406640)

[e-Table 6.12 RoB2 adjudications for length of ICU stay 80](#_Toc133406641)

[e-Table 6.13 RoB2 adjudications for length of hospital stay 81](#_Toc133406642)

[12. e-Table 7 Overview of all primary meta-analyses 83](#_Toc133406643)

[13. e-Table 8 Overview of all subgroup analyses 84](#_Toc133406644)

[14. e-Table 9 Sensitivity analyses for missing data across all outcomes 89](#_Toc133406645)

[15. e-Table 10 Full GRADE Evaluation of the Certainty of Evidence 90](#_Toc133406646)

[16. References 96](#_Toc133406647)

# 1. e-Appendix 1. Protocol specifications and deviations

| **Specification** | **Reason for deviation** |
| --- | --- |
| The co-primary outcome serious adverse events (SAEs) was defined as proportion of patients with one or more SAEs (as defined in the original trials or any untoward medical occurrence that fulfils the International Council on Harmonization Guideline for Good Clinical Practice’s definition) in the protocol. | We reported SAEs separately as five trials had predefined and reported SAEs and based our primary conclusions on these, including the sensitivity and subgroup analyses. However, as this might underestimate the results, we also reported highest proportion of SAEs including mortality and cumulated proportion of SAEs in line with analyses in the previous review^1^. |
| Risk of bias was protocolized with ROB 1 tool. | We used risk of bias (RoB) 2 tool as this is latest updated tool, which gives a more nuanced risk of bias assessment compared to the previously used RoB 1 tool. |
| The secondary Bayesian analysis of the co-primary outcome, all-cause mortality, was not part of the original protocol, but was added to the updated PROSPERO registry (CRD42022312572)^2^. | We preplanned a Bayesian analysis of all-cause mortality in the updated protocol to nuance the co-primary result of all-cause mortality. |

# 2. e-Appendix 2****. PRISMA checklist****

The Preferred Reporting Items for Systematic Reviews and Meta-Analyses (PRISMA) 2020^3^

| **Section and topic** | **Item#** | **Checklist item** | **Pages** |
| --- | --- | --- | --- |
| **TITLE** | | | |
|  | 1 | Identify the report as a systematic review | P 1 |
| **ABSTRACT** | | | |
|  | 2 | See the PRISMA 2020 for Abstracts checklist. | P 1 |
| **INTRODUCTION** | | | |
| Rationale | 3 | Describe the rationale for the review in the context of existing knowledge. | P 2 |
| Objectives | 4 | Provide an explicit statement of the objective(s) or question(s) the review addresses. | P 2 |
| **METHODS** | | | |
| Eligibility criteria | 5 | Specify the inclusion and exclusion criteria for the review and how studies were grouped for the syntheses. | P 2 |
| Information sources | 6 | Specify all databases, registers, websites, organisations, reference lists and other sources searched or consulted to identify studies. Specify the date when each source was last searched or consulted. | P 3 |
| Search strategy | 7 | Present the full search strategies for all databases, registers and websites, including any filters and limits used. | P 3, e-Appendix 3 |
| Selection process | 8 | Specify the methods used to decide whether a study met the inclusion criteria of the review, including how many reviewers screened each record and each report retrieved, whether they worked independently, and if applicable, details of automation tools used in the process. | P 3-4 |
| Data collection process | 9 | Specify the methods used to collect data from reports, including how many reviewers collected data from each report, whether they worked independently, any processes for obtaining or confirming data from study investigators, and if applicable, details of automation tools used in the process. | P 4 |
| Data items | 10a | List and define all outcomes for which data were sought. Specify whether all results that were compatible with each outcome domain in each study were sought (e.g. for all measures, time points, analyses), and if not, the methods used to decide which results to collect. | P 4, e-Appendix 4 |
|  | 10b | List and define all other variables for which data were sought (e.g. participant and intervention characteristics, funding sources). Describe any assumptions made about any missing or unclear information. | P 2-5, Tabel 1, e-Table 5 |
| Study risk of bias assessment | 11 | Specify the methods used to assess risk of bias in the included studies, including details of the tool(s) used, how many reviewers assessed each study and whether they worked independently, and if applicable, details of automation tools used in the process. | P 4-5, e-Table 6.1-13 |
| Effect measures | 12 | Specify for each outcome the effect measure(s) (e.g. risk ratio, mean difference) used in the synthesis or presentation of results. | P 3 |
| Synthesis methods | 13a | Describe the processes used to decide which studies were eligible for each synthesis (e.g. tabulating the study intervention characteristics and comparing against the planned groups for each synthesis (item #5)). | P 2 |
|  | 13b | Describe any methods required to prepare the data for presentation or synthesis, such as handling of missing summary statistics, or data conversions. | P 6 |
|  | 13c | Describe any methods used to tabulate or visually display results of individual studies and syntheses. | P 4-5 |
|  | 13d | Describe any methods used to synthesize results and provide a rationale for the choice(s). If meta-analysis was performed, describe the model(s), method(s) to identify the presence and extent of statistical heterogeneity, and software package(s) used. | P 4-5 |
|  | 13e | Describe any methods used to explore possible causes of heterogeneity among study results (e.g. subgroup analysis, meta-regression). | P 5-6 |
|  | 13f | Describe any sensitivity analyses conducted to assess robustness of the synthesized results. | p 5-6 |
| Reporting bias assessment | 14 | Describe any methods used to assess risk of bias due to missing results in a synthesis (arising from reporting biases). | P 4 |
| Certainty assessment | 15 | Describe any methods used to assess certainty (or confidence) in the body of evidence for an outcome. | P 6-7 |
| **RESULTS** | | | |
| Study selection | 16a | Describe the results of the search and selection process, from the number of records identified in the search to the number of studies included in the review, ideally using a flow diagram. | P 7, Figure 1 |
|  | 16b | Cite studies that might appear to meet the inclusion criteria, but which were excluded, and explain why they were excluded. | P 7, Figure 1 |
| Study characteristics | 17 | Cite each included study and present its characteristics. | P 7, Figire 1, e-Table 5 |
| Risk of bias in studies | 18 | Present assessments of risk of bias for each included study. | P 7-8, eTable 6.1-13 |
| Results of individual studies | 19 | For all outcomes, present, for each study: (a) summary statistics for each group (where appropriate) and (b) an effect estimate and its precision (e.g. confidence/credible interval), ideally using structured tables or plots. | P 7-11, appendix 6-8 |
| Results of syntheses | 20a | For each synthesis, briefly summarise the characteristics and risk of bias among contributing studies. | P 7, e-Table 6.1-13 |
|  | 20b | Present results of all statistical syntheses conducted. If meta-analysis was done, present for each the summary estimate and its precision (e.g. confidence/credible interval) and measures of statistical heterogeneity. If comparing groups, describe the direction of the effect. | P 7-10, appendix 6-8, e-Table 7-9 |
|  | 20c | Present results of all investigations of possible causes of heterogeneity among study results. | e-Table 8 |
|  | 20d | Present results of all sensitivity analyses conducted to assess the robustness of the synthesized results. | e-table 9 |
| Reporting biases | 21 | Present assessments of risk of bias due to missing results (arising from reporting biases) for each synthesis assessed. | Table 2 and e-table 10 |
| Certainty of evidence | 22 | Present assessments of certainty (or confidence) in the body of evidence for each outcome assessed. | Table 2 and e-table 10 |
| **DISCUSSION** | | | |
| Discussion | 23a | Provide a general interpretation of the results in the context of other evidence. | P 11 |
|  | 23b | Discuss any limitations of the evidence included in the review. | P 11-12 |
|  | 23c | Discuss any limitations of the review processes used. | P 12 |
|  | 23d | Discuss implications of the results for practice, policy, and future research. | P 12 |
| **OTHER INFORMATION** | | | |
| Registration and protocol | 24a | Provide registration information for the review, including register name and registration number, or state that the review was not registered. | P 1 |
|  | 24b | Indicate where the review protocol can be accessed, or state that a protocol was not prepared. | P 1 |
|  | 24c | Describe and explain any amendments to information provided at registration or in the protocol. | Appendix 1 |
| Support | 25 | Describe sources of financial or non-financial support for the review, and the role of the funders or sponsors in the review. | P 1 |
| Competing interests | 26 | Declare any competing interests of review authors. | P 1 |
| Availability of data, code and other materials | 27 | Report which of the following are publicly available and where they can be found; template data collection forms; data extracted from included studies; data used for all analyses; analytic code; any other materials used in the review. | Appendix 4 |

# 3. e-Appendix 3. Full search strategy

**Medline**

Previous review last updated April 29^th^, 2019

Updated search March 21^st^, 2022 Records identified: 576

Updated search September 6^th^, 2022 Records identified: 88

Search strategy:

Population

| #1 sepsis [MeSH Terms]  #2 sepsis OR septic OR “Systemic inflammatory response syndrome” OR SIRS OR bacteraemi* OR bacteremi* OR septicaemi* OR septicemi* OR endotoxemi* [All Fields] |  |  |
| --- | --- | --- |
| #3 “multiple organ failure” [MeSH Terms] |  |  |
| #4 “multiple organ failure"[All Fields] |  |  |
| #5 vasoplegia [MeSH Terms]  #6 "vasoplegia"[All Fields] |  |  |
|  |  |  |
| #7 (filter: from 29/04/2019) | #1 OR #2 OR #3 OR #4 OR #5 OR #6 | 40256 |

Intervention

| #8 “fluid therapy” [MeSH Terms]  #9 fluid* OR volume* [All Fields] |  |  |
| --- | --- | --- |
| #10 resuscitation [MeSH Terms]  #11 "resuscitation"[All Fields] |  |  |
| #12 (filter: from 29/04/2019) | #8 OR #9 OR #10 OR #11 | 229121 |

Methodological filter

| #13 random* OR blind* OR placebo OR meta-analys* [All Fields] | (filter: from 29/04/2019) | 354075 |
| --- | --- | --- |
|  |  |  |

#14 #7 AND #12 AND #13 576

**Epistemonikos**

Previous review last updated April 29^th^, 2019

Updated search March 21^st^, 2022 Records identified: 54

Updated search September 6^th^, 2022 Records identified: 34

Search strategy:

(sepsis OR septic OR "Systemic inflammatory response syndrome" OR SIRS OR bacteraemi* OR bacteremia* OR septicaemi* OR septicemi* OR endotoxemi* OR "multiple organ failure" OR "multiple organ failure" OR vasoplegia OR "vasoplegia") AND ("fluid therapy" OR fluid* OR volume* OR resuscitation OR "resuscitation") AND (random* OR blind* OR placebo OR meta-analys*)

Advanced search. Only supports English.

**Science Citation Index**

Previous review last updated May 2^nd^, 2019

Updated search March 21^st^, 2022 Records identified: 609

Updated search September 6^th^, 2022 Records identified: 69

Search strategy:

#1 TS=(sepsis OR septic* OR 'Systemic inflammatory response syndrome' OR SIRS OR bacteraemi OR bacteremi OR endotoxemi OR 'multiple organ failure' OR vasoplegia)

#2 TS=('fluid therapy' OR fluid OR volume OR resuscitation)

#3 #2 AND #1

#4 TS=(random* OR blind* OR placebo* OR meta-analys*)

#5 #4 AND #3 (Filter: Publication year from 2019 to 2022)

**BIOSIS**

Previous review last updated May 2^nd^, 2019

Updated search March 21^st^, 2022 Records identified: 143

Updated search September 6^th^, 2022 Records identified: 17

#1 TS=(sepsis OR septic* OR 'Systemic inflammatory response syndrome' OR SIRS OR bacteraemi OR bacteremi OR endotoxemi OR 'multiple organ failure' OR vasoplegia)

#2 TS=('fluid therapy' OR fluid OR volume OR resuscitation)

#3 #2 AND #1

#4 TS=(random* OR blind* OR placebo* OR meta-analys*)

#5 #4 AND #3 (From 02/05/2019 to 21/03/2022)

**Cochrane Central Register of Controlled Trials (CENTRAL; 2017, Issue 7) in the Cochrane Library**

Previous review last updated August 1^st^ 2017

Updated search March 21^st^ 2022 Records identified: 3178

Updated search September 6^th^ 2022 Records identified: 356

**Cochrane Database of Systematic Reviews in the Cochrane Library (2018, Issue 10)**

Previous review last updated May 2^nd^ 2019

Updated search March 21^st^ 2022 Records identified: 284

Updated search September 6^th^ 2022 Records identified: 33

**Cochrane Database of Cochrane protocols**

Updated search March 21^st^ 2022 Records identified: 57

Updated search September 6^th^ 2022 Records identified: 11

#1 MeSH descriptor: [Sepsis] explode all trees

#2 MeSH descriptor: [Multiple Organ Failure] explode all trees

#3 MeSH descriptor: [Vasoplegia] explode all trees

#4 (sepsis or septic* or 'Systemic inflammatory response syndrome' or SIRS or bacteraemi or bacteremi or endotoxemi or 'multiple organ failure' or vasoplegia)

#5 #1 or #2 or #3 or #4

#6 MeSH descriptor: [Fluid Therapy] explode all trees

#7 MeSH descriptor: [Resuscitation] explode all trees

#8 ('fluid therapy' or fluid or volume or resuscitation)

#9 #6 or #7 or #8

#10 #5 and #9 (Filter: Year first published 2019)

**EMBASE**

Previous review last updated May 2^nd^, 2019

Updated search March 21^st^, 2022 Records identified: 1538

Updated search September 6^th^ 2022 Records identified: 160

1. exp sepsis/

2. exp multiple organ failure/

3. exp vasoplegia/

4. (sepsis or septic* or 'Systemic inflammatory response syndrome' or SIRS or bacteraemi or bacteremi or endotoxemi or 'multiple organ failure' or vasoplegia).mp. [mp=title, abstract, heading word, drug trade name, original title, device manufacturer, drug manufacturer, device trade name, keyword, floating subheading word]

5. 1 or 2 or 3 or 4

6. exp fluid therapy/

7. exp resuscitation/

8. ('fluid therapy' or fluid or volume or resuscitation).mp. [mp=title, abstract, heading word, drug trade name, original title, device manufacturer, drug manufacturer, device trade name, keyword, floating subheading word]

9. 6 or 7 or 8

10. 5 and 9

11. (random* or blind* or placebo* or meta-analys*).mp. [mp=title, abstract, heading word, drug trade name, original title, device manufacturer, drug manufacturer, device trade name, keyword, floating subheading word]

12. 10 and 11 (Filter: from 02/05/2019 to 21/03/2022)

**Records identified through registries:**

We searched databases of ongoing trials as protocolized which included ClinicalTrials.gov, the EU Clinical Trials register (and the World Health Organization (WHO) International Clinical Trials Registry Platform Search Portal). The metaRegister of Controlled Trials was closed in 2014 and therefore not available.

Updated search September 6^th^ 2022 Records identified: 145

# 4. e-Appendix 4. Data extraction template

**Trial identification and characteristics**

| Author | Year | Country | No. of patients | Population  (inclusion & exclusion criteria) | Fluid types | Intervention | Control | Other interventions |
| --- | --- | --- | --- | --- | --- | --- | --- | --- |
|  |  |  |  |  |  |  |  |  |

**Primary outcomes**

| **All-cause mortality** | | | |
| --- | --- | --- | --- |
| **Intervention** | | **Control** | |
| Events | No. of patients | Events | No. of patients |
|  |  |  |  |

| **Serious adverse events** | | | |
| --- | --- | --- | --- |
| **Intervention** | | **Control** | |
| Events | No. of patients | Events | No. of patients |
|  |  |  |  |

| **Heallth-related quality-of-life** | | | |
| --- | --- | --- | --- |
| **Intervention** | | **Control** | |
| Events | No. of patients | Events | No. of patients |
|  |  |  |  |

**Secondary outcomes**

| **Duration of mechanical ventilation, days** | | | | | |
| --- | --- | --- | --- | --- | --- |
| **Intervention** | | | **Control** | | |
| Mean (SD) | Median (IQR) | No. of patients | Mean (SD) | Median (IQR) | No. of patients |
|  |  |  |  |  |  |

| **Ventilator-free days** | | | | | |
| --- | --- | --- | --- | --- | --- |
| **Intervention** | | | **Control** | | |
| Mean (SD) | Median (IQR) | No. of patients | Mean (SD) | Median (IQR) | No. of patients |
|  |  |  |  |  |  |

| **Duration of vasopressor or inotropes, hours** | | | | | |
| --- | --- | --- | --- | --- | --- |
| **Intervention** | | | **Control** | | |
| Mean (SD) | Median (IQR) | No. of patients | Mean (SD) | Median (IQR) | No. of patients |
|  |  |  |  |  |  |

| **Vasopressor -free days** | | | | | |
| --- | --- | --- | --- | --- | --- |
| **Intervention** | | | **Control** | | |
| Mean (SD) | Median (IQR) | No. of patients | Mean (SD) | Median (IQR) | No. of patients |
|  |  |  |  |  |  |

| **Use of renal replacement therapy** | | | |
| --- | --- | --- | --- |
| **Intervention** | | **Control** | |
| Events | No. of patients | Events | No. of patients |
|  |  |  |  |

| **Duration of renal replacement therapy, days** | | | | | |
| --- | --- | --- | --- | --- | --- |
| **Intervention** | | | **Control** | | |
| Mean (SD) | Median (IQR) | No. of patients | Mean (SD) | Median (IQR) | No. of patients |
|  |  |  |  |  |  |

| **Renal replacement therapy-free days** | | | | | |
| --- | --- | --- | --- | --- | --- |
| **Intervention** | | | **Control** | | |
| Mean (SD) | Median (IQR) | No. of patients | Mean (SD) | Median (IQR) | No. of patients |
|  |  |  |  |  |  |

| **Incidence of acute kidney injury** | | | |
| --- | --- | --- | --- |
| **Intervention** | | **Control** | |
| Events | No. of patients | Events | No. of patients |
|  |  |  |  |

**Exploratory outcomes**

| **Use of blood products, units** | | | |
| --- | --- | --- | --- |
| **Intervention** | | **Control** | |
| Events | No. of patients | Events | No. of patients |
|  |  |  |  |

| **ICU length of stay, days** | | | | | |
| --- | --- | --- | --- | --- | --- |
| **Intervention** | | | **Control** | | |
| Mean (SD) | Median (IQR) | No. of patients | Mean (SD) | Median (IQR) | No. of patients |
|  |  |  |  |  |  |

| **Hospital length of stay** | | | | | |
| --- | --- | --- | --- | --- | --- |
| **Intervention** | | | **Control** | | |
| Mean (SD) | Median (IQR) | No. of patients | Mean (SD) | Median (IQR) | No. of patients |
|  |  |  |  |  |  |

No.: number, SD: standard deviation, IQR: interquartile range, ICU: Intensive care uni

# 5. e-Appendix 5 Assessment of risk of random errors in meta-analyses

In line with our statistical analysis plan^2^, we applied a pragmatic approach as suggested by Jakobsen et al.^17,18^ instead of Bonferoni corrections for multiple comparison, which may be considered too conservative, and no adjustment, which may be too liberal. We divided the pre-specified P-value threshold with the value halfway between 1 (no adjustment) and the number of primary outcome comparisons (Bonferroni adjustment).

We had data available for two of three co-primary outcomes, thus the threshold for the P-value was calculated as follows: 0.05 / 1.5 = 0.033

We had data available for all eight secondary outcomes, thus the threshold for the P-value for these outcomes was calculated as follows: 0.05 / 4 = 0.0125

We had data available for three exploratory outcomes, thus the threshold for the P-value for these outcomes was calculated as follows: 0.05 / 2 = 0.025

We had prespecified to use Chi-squared test the statistical heterogeneity considering P=0.10 significant in all subgroup analyses.

# 6. e-Appendix 6 Analyses of primary outcomes

## 6.1 All-cause mortality - meta-analysis and TSA

Meta-analysis of 13 trials reporting all-cause mortality.

### 6.1.1 Meta-analysis and forest plot of all-cause mortality


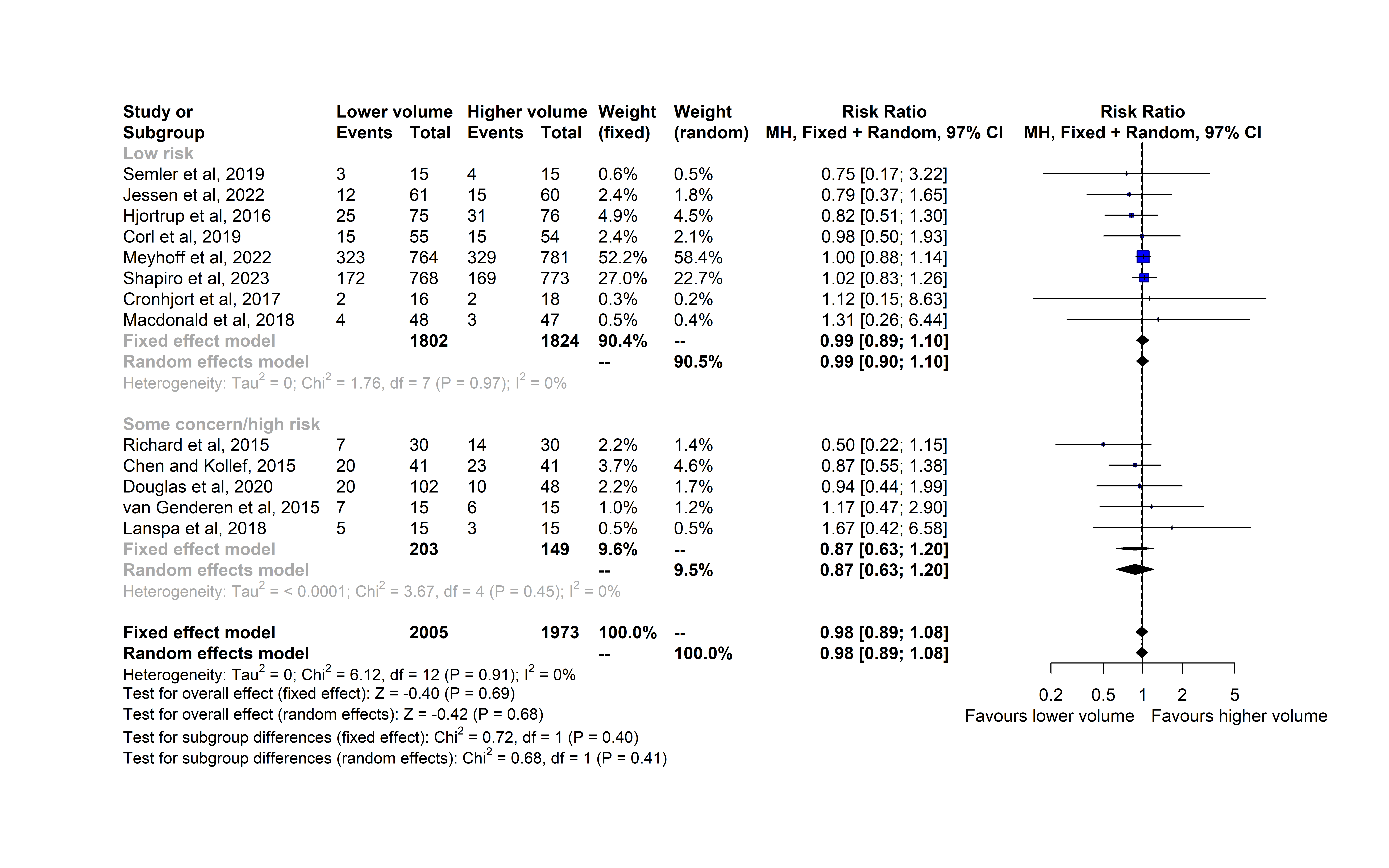


### 6.1.2 TSA of all-cause mortality (low RoB trials only)


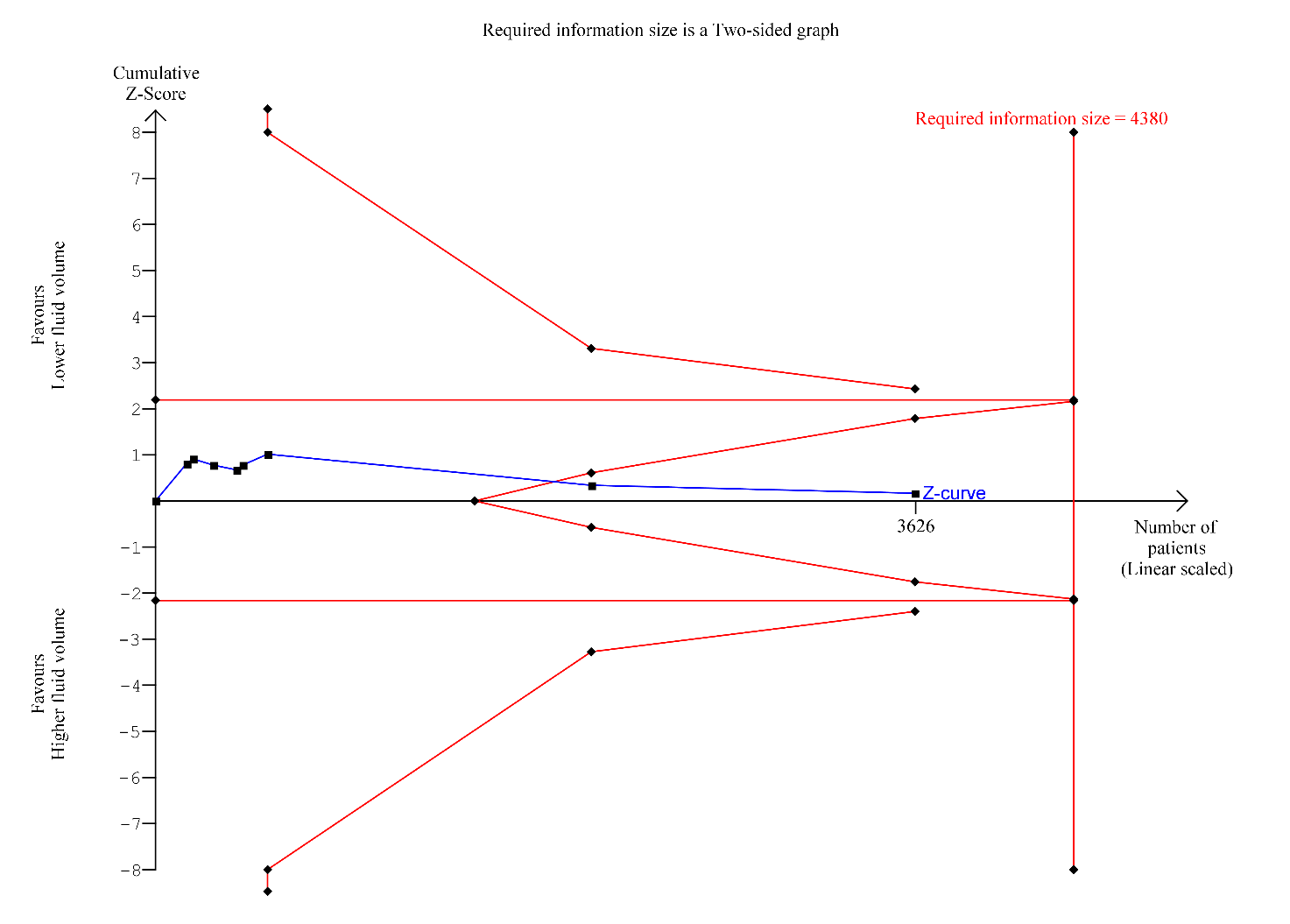


Conventional monitoring boundary for harm

Trial sequential monitoring boundary for harm

Trial sequential monitoring boundary for benefit

Area of futility

Favors

Higher fluid volumes

Favors

Lower fluid volumes

3626

No. of patients

Required information size = 4380

Required information size is a Two-sided graph

Conventional monitoring boundary for benefit

Trial sequential analysis (TSA) for all-cause mortality in 8 low risk of bias trials. We used a control event proportion of 31.1%, alpha 3.3% (two-sided), beta 10% (power 90%), model variance-based heterogeneity adjustment, and an a priori relative risk reduction of 15% in the analysis. The TSA adjusted CI in the fixed effects model was 0.89 to 1.11 with a diversity D^2^ of 0%. The blue cumulative Z-curve crossed the area of futility. Thus, the TSA is conclusive and a relative risk reduction of 15% is unlikely. 83% (3626 patients) of the required information size of 4380 patients was accrued.

### 6.1.3 TSA of all-cause mortality (all trials)

Conventional monitoring boundary for harm

Trial sequential monitoring boundary for harm

Trial sequential monitoring boundary for benefit

Area of futility

Favors

Higher fluid volumes

Favors

Lower fluid volumes

3978

No. of patients

Required information size = 4292

Required information size is a Two-sided graph


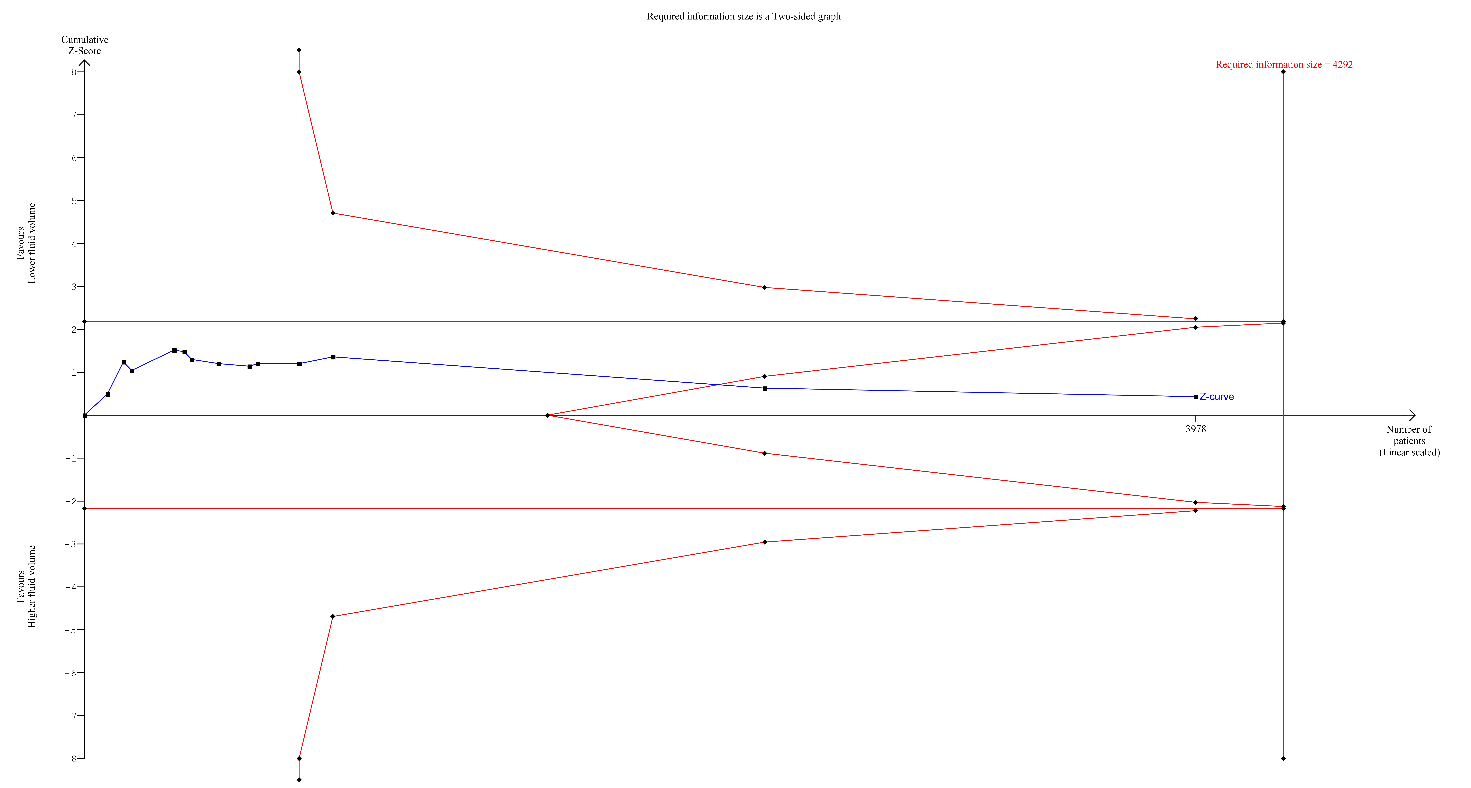


Conventional monitoring boundary for benefit

Trial sequential analysis (TSA) for all-cause mortality in 13 trials. We used a control event proportion of 31.6%, alpha 3.3% (two-sided), beta 10% (power 90%), model variance-based heterogeneity adjustment, and an a priori relative risk reduction of 15% in the analysis. The TSA adjusted CI in the fixed effects model was 0.89 to 1.08 with a diversity D^2^ of 0%. The blue cumulative Z-curve crossed the area of futility. Thus, the TSA is conclusive and a relative risk reduction of 15% is unlikely. 93% (3978 patients) of the required information size of 4292 patients was accrued.

### 6.1.4 Funnel plot of all-cause mortality (all trials)


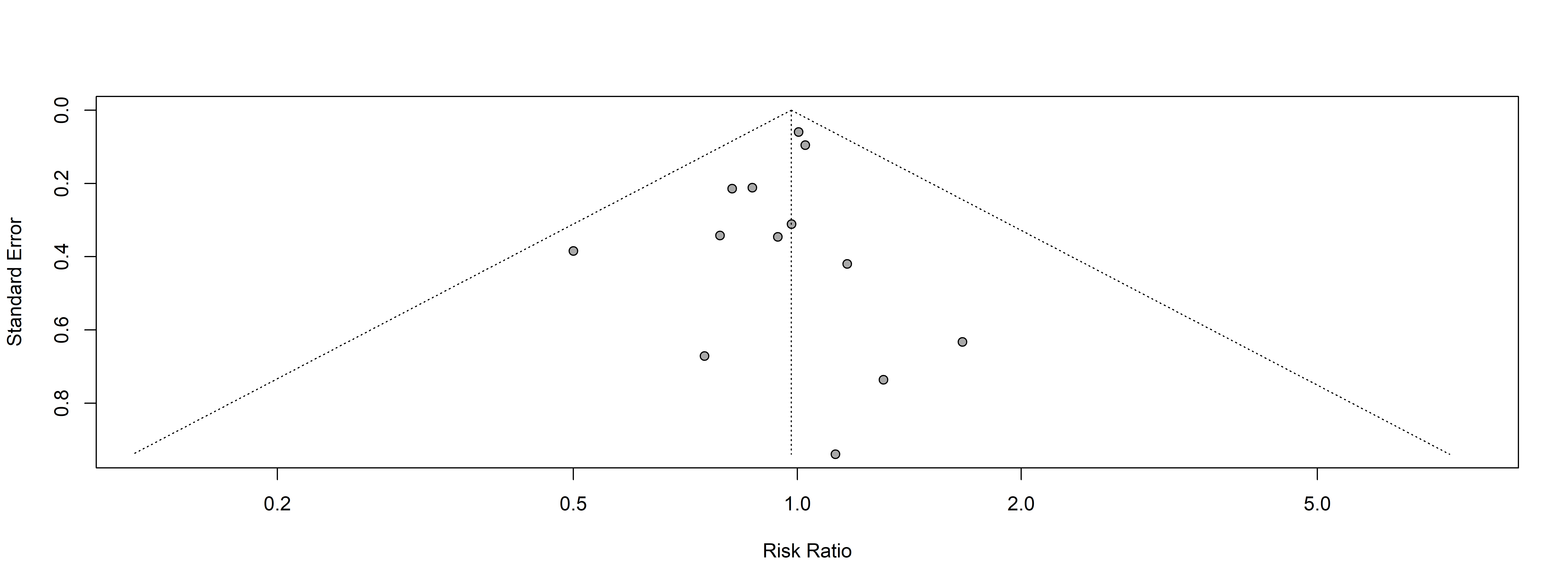


Asymmetry was tested with Harbord test (P=0.38).

### 6.1.5 Bayesian secondary analyses of all-cause mortality (all trials)

Priors for Bayesian analyses

The primary Bayesian analysis used weak priors (centered on no difference) as this allows the trial results to lead the model while the priors stabilize the model and allow realistic effect sizes only. Therefore, the priors create the framework around the model but practically have minimal influence on the results. Priors for heterogeneity are generally vague and primarily serve to stabilize the model. All choices of priors were based on previously used priors and recommendations^19–21^.

We used a normally distributed prior for the treatment effect (mean = 0, SD = 1) in both models corresponding to a probability distribution of odds ratios (OR) centered on 1.00 (no difference) with a 95% central probability mass between 0.14 and 7.10. We used a half-normal prior with SD = 1 for the between-trial SD (*Tau*) in the random effects meta-analysis corresponding to a 95% central probability mass between 0.03 and 2.25.

We conducted sensitivity analyses with more sceptical priors. First a normal(0, 0.35) prior for the treatment effect corresponding to a probability distribution of OR centered on 1.00 with a 95% central probability mass between 0.50 and 1.99, and a half-normal(0.35) prior for the between-trial SDs. Second, a normal(0,0.15) prior for the treatment effect corresponding to a prior probability distribution of OR centered on 1.00 with 95% central probability mass between 0.75 and 1.34. and a half-normal(0.15) prior for the between-trial SD corresponding to a 95% central probability mass between 0.00 and 0.34. The sceptical priors were chosen to shrink effect estimates towards no difference thereby being sceptical of large effects, as large effects were unexpected and are rarely observed in pragmatic critical care trials.

Complete posterior distributions were presented graphically and summarized using median values as point estimates and with percentile-based 95% credibility intervals (e-Figures 1S-6S).

Model diagnostics

We used the default dynamic Hamiltonian Monte Carlo sampler in Stan with four chains with 10,000 post-warm-up iterations each (i.e. 40000 in all) and at least 1000 bulk/tail effective sample sizes as previously described^22^. Model adequacy was assessed by using density and trace plots to assess convergency and the updated Rhat statistics, which we required to be <1.01^22^. Finally, we assessed model fit using graphical posterior predictive checks^23^ and Pareto-smoothed importance sampling leave-one-out cross-validation (primarily focused on the effective number of parameters compared to the actual number of parameters in each models)^24^. These criteria were fulfilled for all models.

### 6.1.6 Bayesian sensitivity analyses of all-cause mortality

### e-Figure 1 Bayesian analysis of all-**cause mortality** (random effects model)

Full posterior probability distribution for the treatment effect on all-cause mortality from the primary Bayesian analysis using weakly informative priors centered on no difference. The plot displays the relative difference (relative risk, RR) in a random effects model. An RR <1 favors lower fluid volumes while an RR >1 favors higher fluid volumes. The upper subplot displays the cumulative posterior distribution, and thus displays the probabilities (vertical axes) of various effect sizes (horizontal axis). The lower subplots display the entire posterior distribution, with the bold, vertical line indicating the median value (used as the point estimate) and the area highlighted in red indicating the percentile-based 95% credible interval. The vertical black line represents exactly no difference.

The probability of any benefit (i.e., an RR < 1.00) with lower IV fluid volumes was 71.6%, while the probability of effect sizes smaller than a relative risk reduction of 15% (or the opposite relative risk increase) with lower IV fluid volumes was 94.4%. The probability of a relative risk reduction of at least 15% was 5.1%, while the probability of the corresponding relative risk increase (i.e., an RR of ≥ 1.18) was only 0.4%.


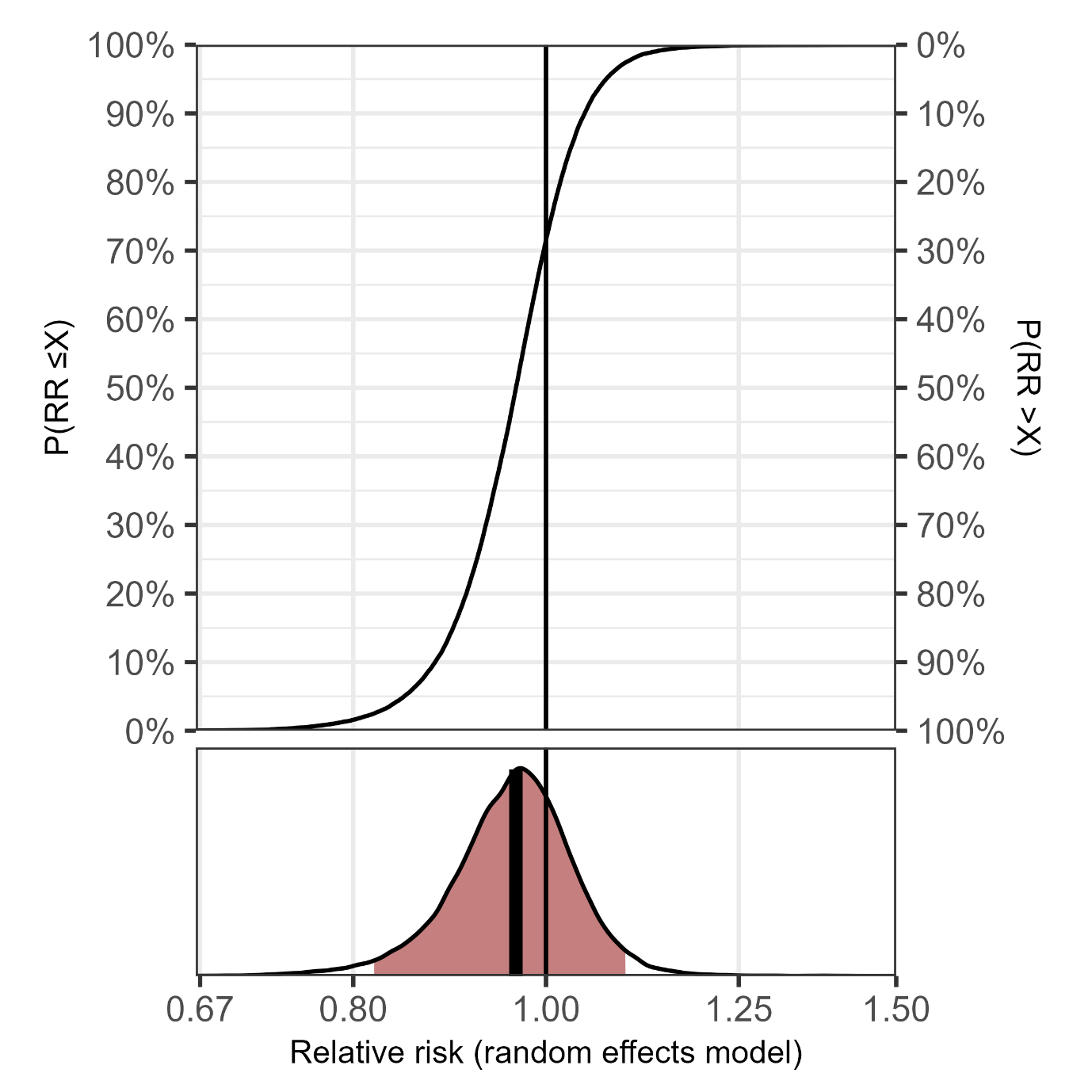


### e-Figure 2 Bayesian sensitivity analysis of all-**cause mortality** (sceptic prior, fixed effect model)

Full posterior probability distribution for the treatment effect on all-cause mortality from a Bayesian sensitivity analysis in a fixed effect model. We chose a sceptical prior using a normal(0, 0.35) prior for the treatment effect. The plot displays the relative difference (relative risk, RR). An RR <1 favours lower fluid volumes while an RR >1 favours higher fluid volumes. The upper subplot displays the cumulative posterior distribution, and thus displays the probabilities (vertical axes) of various effect sizes (horizontal axis). The lower subplots display the entire posterior distribution, with the bold, vertical line indicating the median value (used as the point estimate) and the area highlighted in red indicating the percentile-based 95% credible interval. The vertical black line represents exactly no difference.

The probability of any benefit (i.e., an RR < 1.00) with lower IV fluid volumes was 65.7%, while the probability of effect sizes smaller than a relative risk reduction of 15% (or the opposite relative risk increase) with lower IV fluid volumes was 99.9.%. The probability of a relative risk reduction of at least 15% was 0.1%, while the probability of the corresponding relative risk increase (i.e., an RR of ≥ 1.18) was <0.1%.


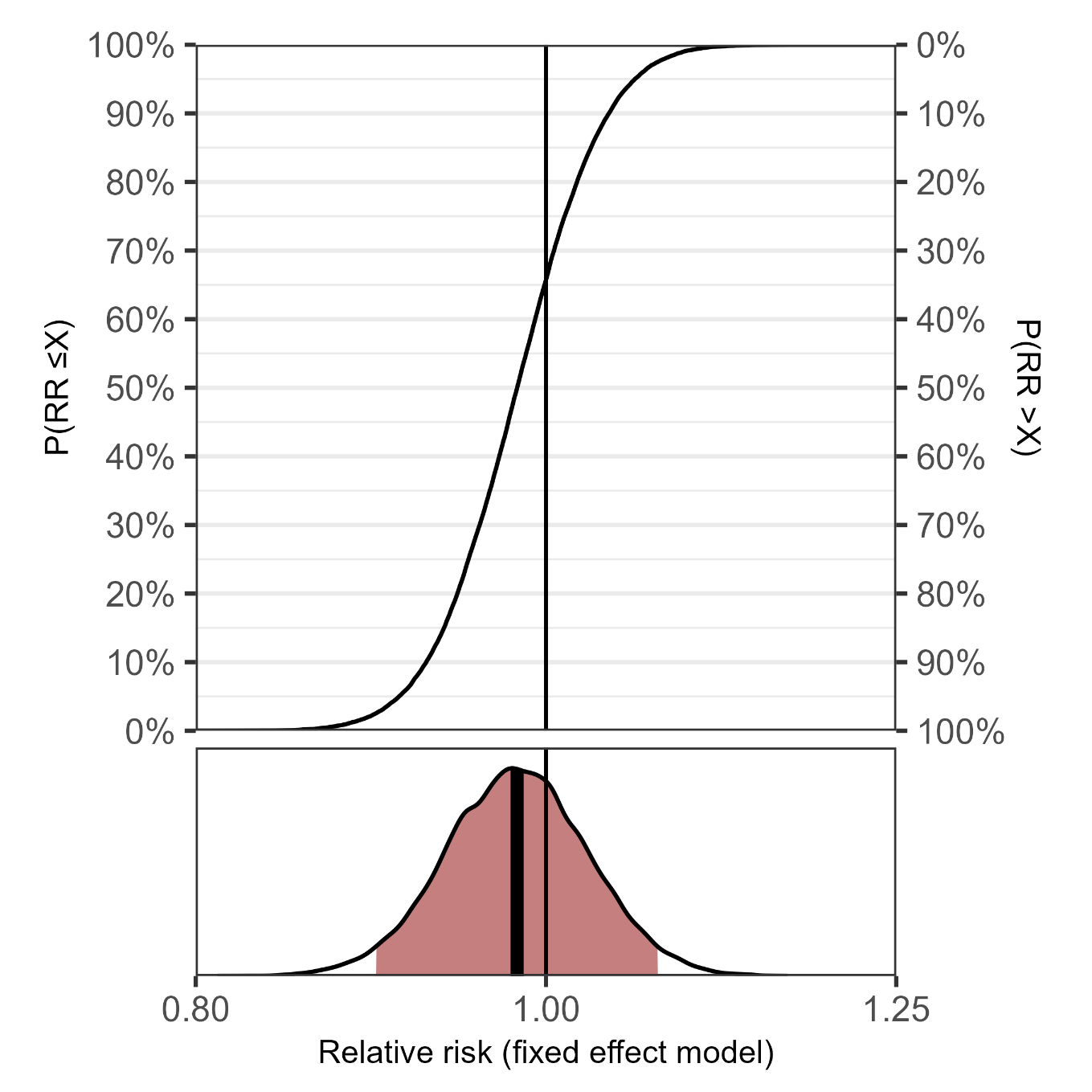


### e-Figure 3 Bayesian sensitivity analysis of all-**cause mortality** (sceptic prior, random effects model)

Full posterior probability distribution for the treatment effect on all-cause mortality from a Bayesian sensitivity analysis in a random effects model. We chose a sceptical prior using a normal(0, 0.35) prior for the treatment effect and a half-normal(0.35) prior for the between-trial SDs, The plot displays the relative difference (relative risk, RR). An RR <1 favours lower fluid volumes while an RR >1 favours higher fluid volumes. The upper subplot displays the cumulative posterior distribution, and thus displays the probabilities (vertical axes) of various effect sizes (horizontal axis). The lower subplots display the entire posterior distribution, with the bold, vertical line indicating the median value (used as the point estimate) and the area highlighted in red indicating the percentile-based 95% credible interval. The vertical black line represents exactly no difference.

The probability of any benefit (i.e., an RR < 1.00) with lower IV fluid volumes was 71.1%, while the probability of effect sizes smaller than a relative risk reduction of 15% (or the opposite relative risk increase) with lower IV fluid volumes was 95.8%. The probability of a relative risk reduction of at least 15% was 4.0%, while the probability of the corresponding relative risk increase (i.e., an RR of ≥ 1.18) was only 0.3%.


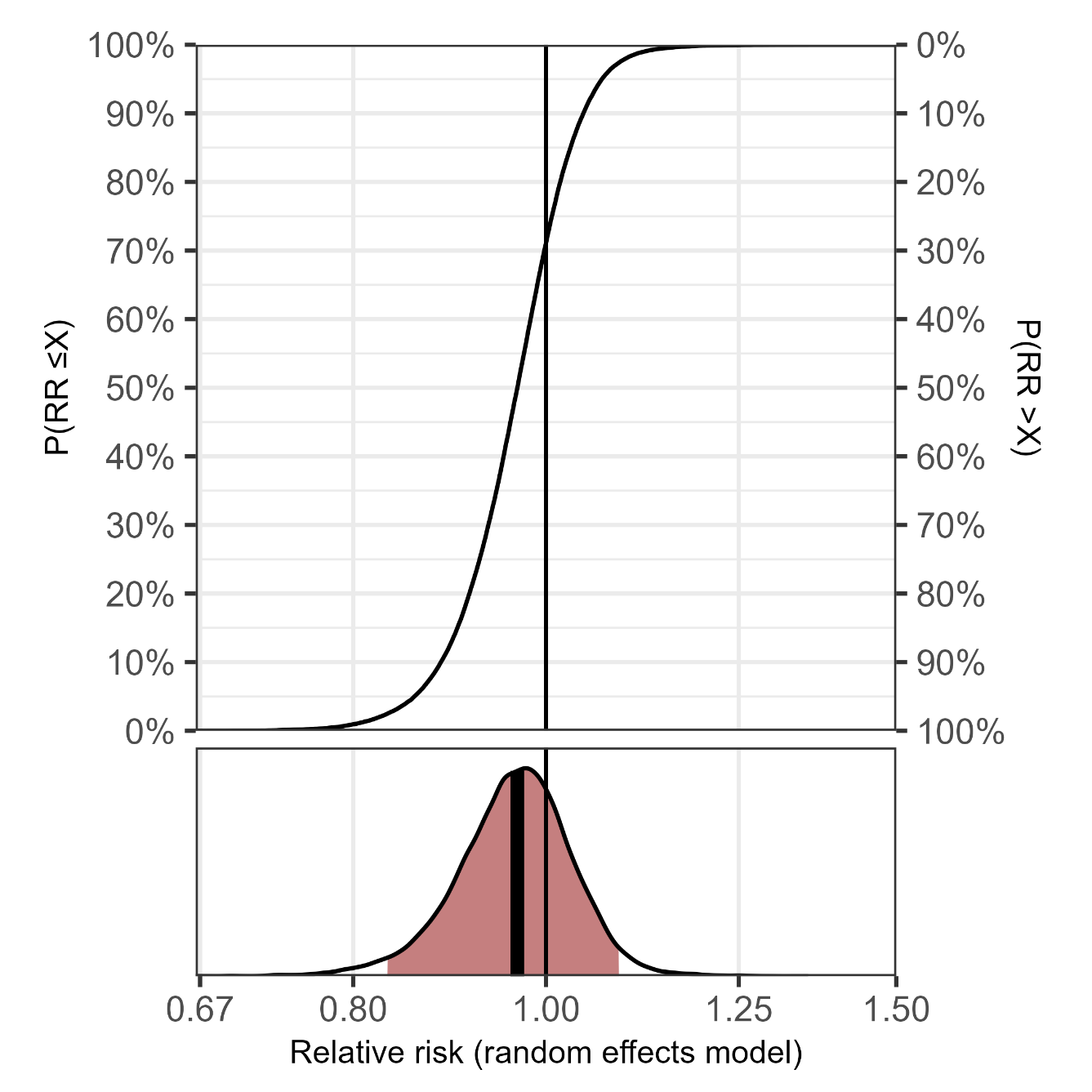


### e-Figure 4 Bayesian sensitivity analysis of all-**cause mortality** (more sceptic prior, fixed effects model)

Full posterior probability distribution for the treatment effect on all-cause mortality from a Bayesian sensitivity analysis in a fixed effect model. We used a more sceptical prior using a normal(0, 0.15) prior for the treatment. The plot displays the relative difference (relative risk, RR). An RR <1 favours lower fluid volumes while an RR >1 favours higher fluid volumes. The upper subplot displays the cumulative posterior distribution, and thus displays the probabilities (vertical axes) of various effect sizes (horizontal axis). The lower subplots display the entire posterior distribution, with the bold, vertical line indicating the median value (used as the point estimate) and the area highlighted in red indicating the percentile-based 95% credible interval. The vertical black line represents exactly no difference.

The probability of any benefit (i.e., an RR < 1.00) with lower IV fluid volumes was 65.2%, while the probability of effect sizes smaller than a relative risk reduction of 15% (or the opposite relative risk increase) with lower IV fluid volumes was >99.9.%. The probability of a relative risk reduction as well an increase of at least 15% were both <0.1%.


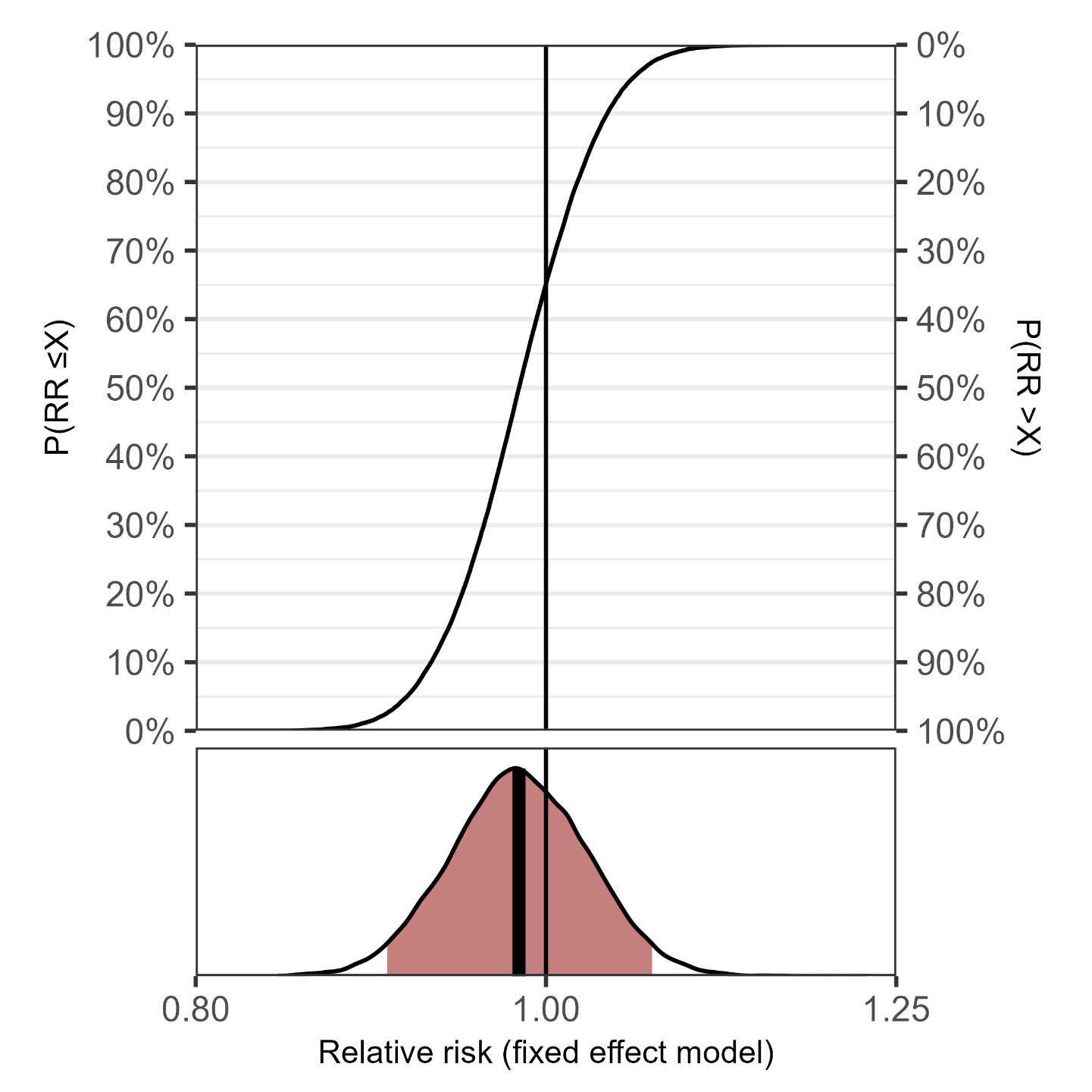


### e-Figure 5 Bayesian sensitivity analysis of all-**cause mortality** (more sceptic prior, random effects model)

Full posterior probability distribution for the treatment effect on all-cause mortality from a Bayesian sensitivity analysis in a random effects model. We used a more sceptical prior using a normal(0, 0.15) prior for the treatment effect and a half-normal(0.15) prior for the between-trial SDs. The plot displays the relative difference (relative risk, RR). An RR <1 favours lower fluid volumes while an RR >1 favours higher fluid volumes. The upper subplot displays the cumulative posterior distribution, and thus displays the probabilities (vertical axes) of various effect sizes (horizontal axis). The lower subplots display the entire posterior distribution, with the bold, vertical line indicating the median value (used as the point estimate) and the area highlighted in red indicating the percentile-based 95% credible interval. The vertical black line represents exactly no difference.

The probability of any benefit (i.e., an RR < 1.00) with lower IV fluid volumes was 69.2%, while the probability of effect sizes smaller than a relative risk reduction of 15% (or the opposite relative risk increase) with lower IV fluid volumes was 98.4.%. The probability of a relative risk reduction of at least 15% was 1.5%, while the probability of the corresponding relative risk increase (i.e., an RR of ≥ 1.18) was 0.1%.


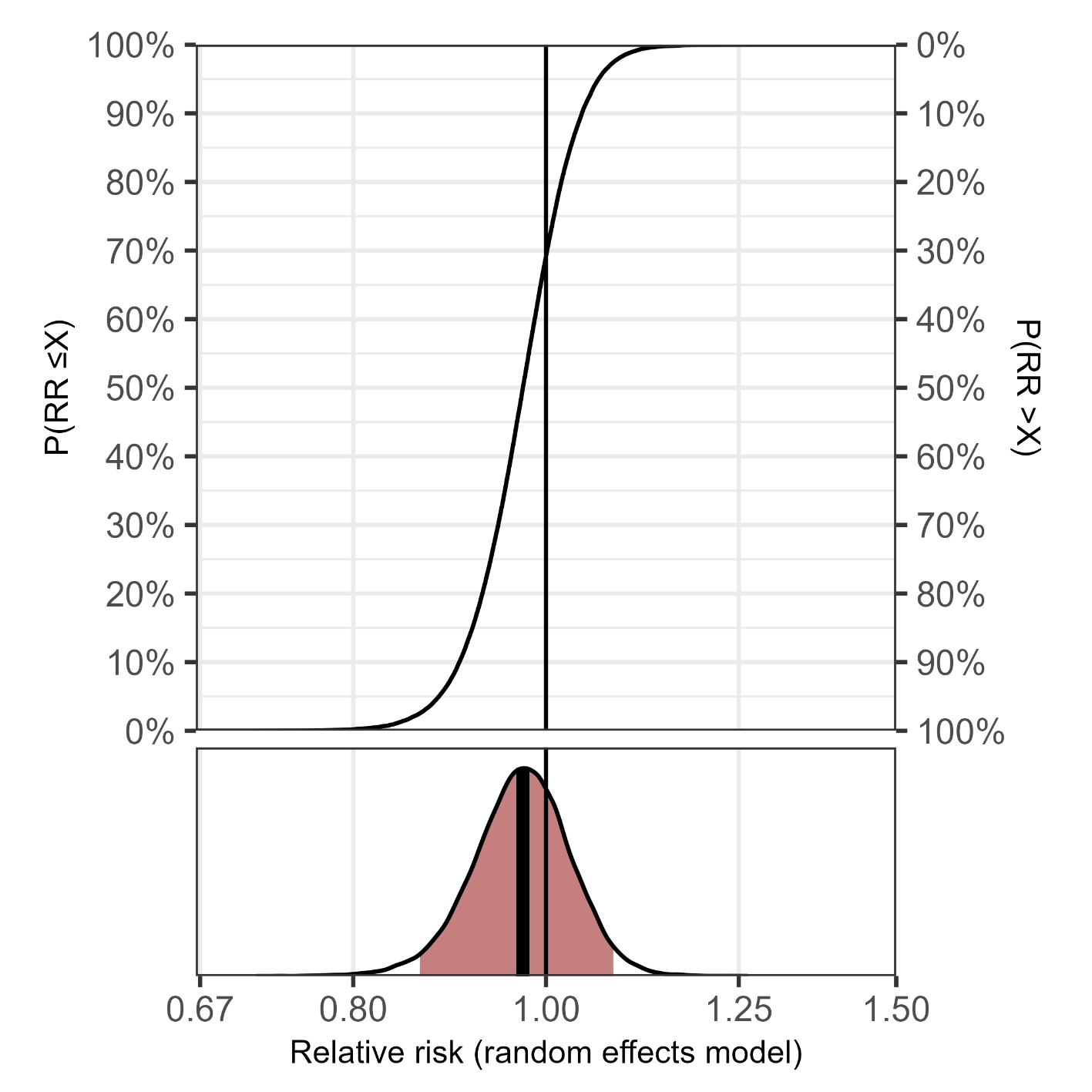


## 6.2 Serious adverse events - meta-analysis and TSA

### 6.2.1 e-Table 1 Highest proportion of SAEs (as defined in the original trial)

| ***** | **Prop. of pts with SAR as defined by the trial** | | | | **Prop. of pts with SAE as defined by the trial** | | | | **Highest proportion of events** | | | |
| --- | --- | --- | --- | --- | --- | --- | --- | --- | --- | --- | --- | --- |
| **Trial** | E: No. events | E: No.  at risk | C: No. events | C: No.  at risk | E: No. events | E: No.  at risk | C: No. events | C: No.  at risk | E: No. events | E: No.  at risk | C: No. events | C: No.  at risk |
| Chen and Kollef^4^ |  |  |  |  |  |  |  |  |  |  |  |  |
| Cronhjort et al.^5^ |  |  |  |  |  |  |  |  |  |  |  |  |
| Hjortrup et al.^6^ | 65 | 75 | 72 | 76 |  |  |  |  | **65** | **75** | **72** | **76** |
| Lanspa et al. ^10^ |  |  |  |  |  |  |  |  |  |  |  |  |
| Macdonald et al. ^9^ |  |  |  |  | 4 | 50 | 4 | 49 | **4** | **50** | **4** | **49** |
| Richard et al. ^7^ |  |  |  |  |  |  |  |  |  |  |  |  |
| Semler et al. ^11^ |  |  |  |  |  |  |  |  |  |  |  |  |
| Van Genderen et al.^8^ |  |  |  |  |  |  |  |  |  |  |  |  |
| Corl et al.^12^ |  |  |  |  |  |  |  |  |  |  |  |  |
| Douglas et al. ^13^ |  |  |  |  | 10 | 98 | 7 | 45 | **10** | **98** | **7** | **45** |
| Jessen et al.^14^ | 0 | 61 | 0 | 62 | 17 | 61 | 18 | 62 | **17** | **61** | **18** | **62** |
| Meyhoff et al.^15^ | 31 | 755 | 32 | 776 | 221 | 751 | 238 | 772 | **221** | **755** | **238** | **776** |
| Shapiro et al.^16^ |  |  |  |  | 21 | 782 | 19 | 781 | **21** | **782** | **19** | **781** |

Prop.: proportion; pts: patients, SAR: serious adverse reaction, SAE: serious adverse events, E: experimental; C: control; No.: number. Types of serious adverse reactions or events from each trial. The cells are left blank if SAE/SAR was not defined in the original trial. The highest proportion of events marked in bold are the numbers included in the following meta-analysis and trial sequential analysis.

* The proportion of patients are reported as the number of patients with one or more SARs (or SAEs) out of the total no of patients at risk receiving either lower IV fluid (E) vs higher IV fluid (C), respectively. One patient might be included more than once due to many SARs or SAEs.

### 6.2.1a Meta-analysis and forest plot of highest proportion of SAE/SAR


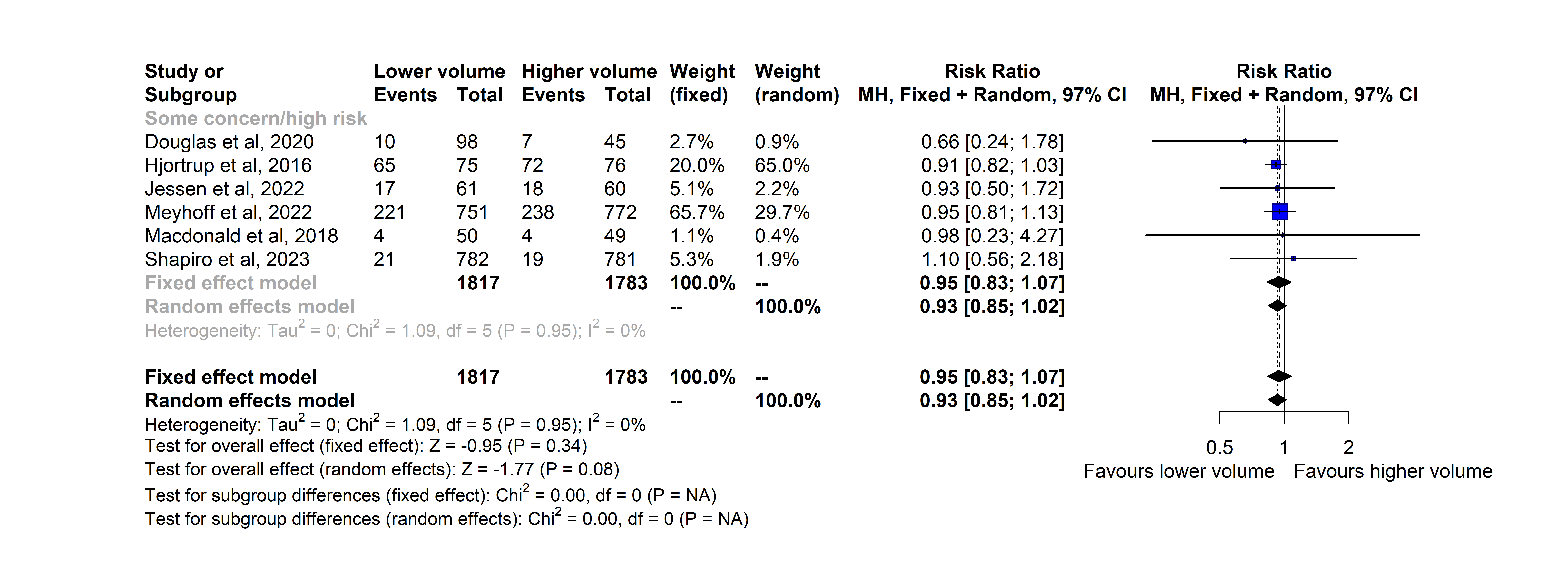


Meta-analysis of six trials reporting serious adverse reactions or events (as defined in original trial) cf. e-Table 4S**.**

### 6.2.1b TSA of highest proportion of SAE/SAR

Conventional monitoring boundary for harm

Trial sequential monitoring boundary for harm

Trial sequential monitoring boundary for benefit

Area of futility

Favors

Higher fluid volumes

Favors

Lower fluid volumes

3602

No. of patients

Required information size = 7788

Required information size is a Two-sided graph

Conventional monitoring boundary for benefit


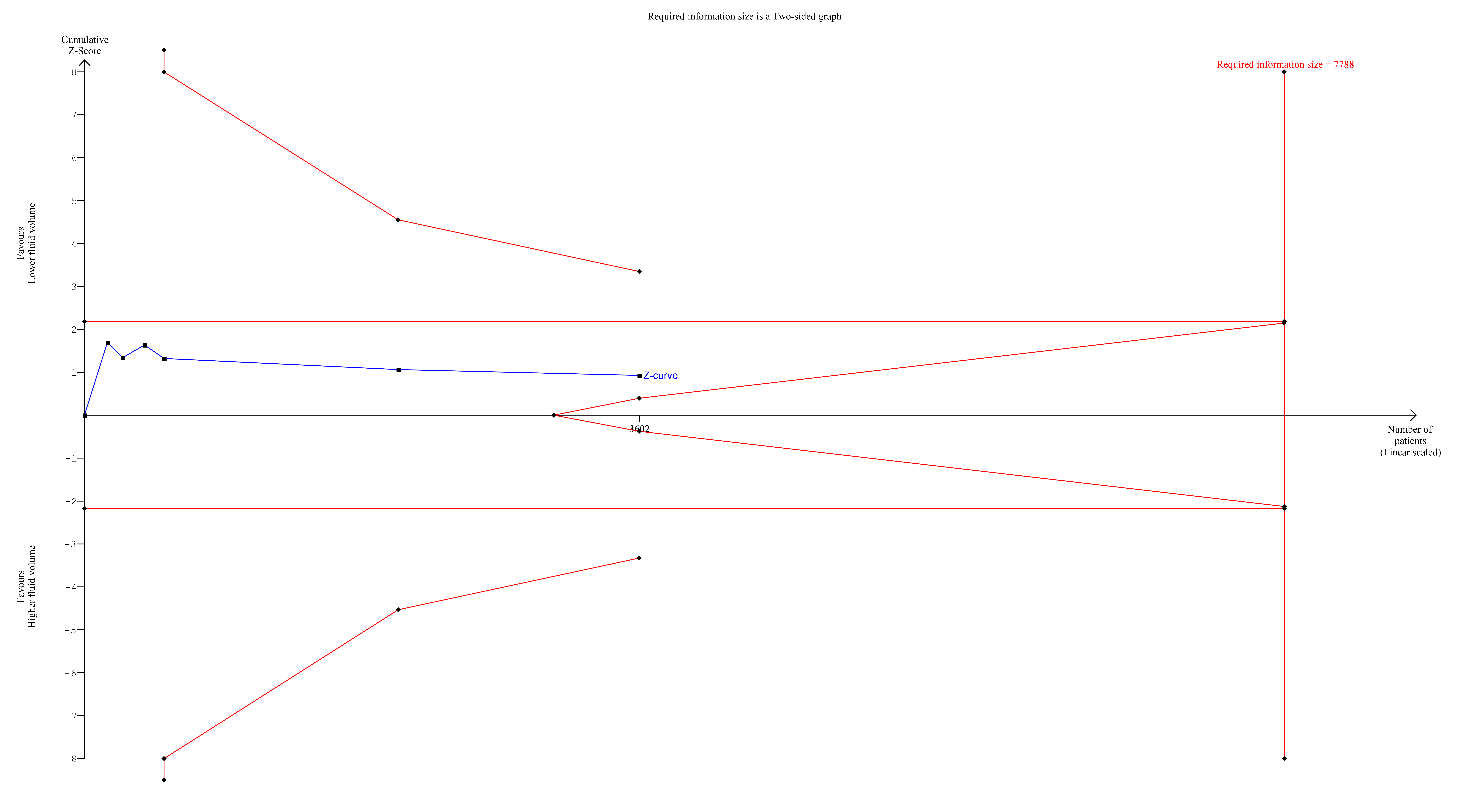


Trial sequential analysis (TSA) of the highest proportion of serious adverse events in six trials. We used a control event proportion of 20.1 %, alpha 3.3% (two-sided), beta 10% (power 90%), heterogeneity adjustment of 0%, and an a priori relative risk reduction of 15% in the analysis. The TSA adjusted confidence interval in the fixed effect model was 0.78 to 1.15 with a diversity D^2^ 0%. The blue cumulative Z-curve did not cross the conventional monitoring boundaries for benefit, harm or futility; thus, the TSA is inconclusive. 46% (3602 patients) of the required information size of 7788 patients was accrued.

### **6.2.2** e-Table 2 Highest proportion of SAE including mortality (based on ICH-GCP categorization)

|  | **All-cause mortality** | | | | ***Prop. of pts with SAR to IV crystalloids or vasopressors** | | | | **Prop. of pts with SAE as defined by the trial** | | | | **Highest proportion of events** | | | |
| --- | --- | --- | --- | --- | --- | --- | --- | --- | --- | --- | --- | --- | --- | --- | --- | --- |
| **Trial** | E: No. events | E: No.  at risk | C: No. events | C: No.  at risk | E: No. events | E: No.  at risk | C: No. events | C: No.  at risk | E: No. events | E: No.  at risk | C: No. events | C: No.  at risk | E: No. events | E: No.  at risk | C: No. events | C: No.  at risk |
| Chen and Kollef^4^ | 20 | 41 | 23 | 41 |  |  |  |  |  |  |  |  | **20** | **41** | **23** | **41** |
| Cronhjort et al.^5^ | 2 | 16 | 2 | 18 |  |  |  |  |  |  |  |  | **2** | **16** | **2** | **18** |
| Hjortrup et al.^6^ | 25 | 75 | 31 | 76 | 65 | 75 | 72 | 76 |  |  |  |  | **65** | **75** | **72** | **76** |
| Lanspa et al. ^10^ | 5 | 15 | 3 | 15 |  |  |  |  |  |  |  |  | **5** | **15** | **3** | **15** |
| Macdonald et al. ^9^ | 4 | 48 | 3 | 47 |  |  |  |  | 4 | 50 | 4 | 49 | **4** | **50** | **4** | **49** |
| Richard et al. ^7^ | 7 | 30 | 14 | 30 |  |  |  |  |  |  |  |  | **7** | **30** | **14** | **30** |
| Semler et al. ^11^ | 3 | 15 | 4 | 15 |  |  |  |  |  |  |  |  | **3** | **15** | **4** | **15** |
| Van Genderen et al.^8^ | 7 | 15 | 6 | 15 |  |  |  |  |  |  |  |  | **7** | **15** | **6** | **15** |
| Corl et al.^12^ | 15 | 55 | 15 | 54 |  |  |  |  |  |  |  |  | **15** | **55** | **15** | **54** |
| Douglas et al. ^13^ | 20 | 102 | 10 | 48 |  |  |  |  | 10 | 98 | 7 | 45 | **20** | **102** | **10** | **48** |
| Jessen et al.^14^ | 12 | 61 | 15 | 60 | 0 | 61 | 0 | 62 | 17 | 61 | 18 | 62 | **17** | **61** | **18** | **62** |
| Meyhoff et al.^15^ | 323 | 764 | 329 | 781 | 31 | 755 | 32 | 776 | 221 | 751 | 238 | 772 | **323** | **764** | **329** | **781** |
| Shapiro et al.^16^ | 172 | 768 | 169 | 773 |  |  |  |  | 21 | 782 | 19 | 781 | **193** | **782** | **188** | **781** |

Prop.: proportion; pts: patients, SAR: serious adverse reaction, SAE: serious adverse events, E: experimental; C: control; No.: number.

Types of serious adverse reactions or events from each trial. The cells are left blank if SAE/SAR was not defined in the original trial. The highest proportion of events marked in bold are the numbers included in the following meta-analysis and trial sequential analysis.

* The proportion of patients are reported as the number of patients with one or more SARs (or SAEs) out of the total no of patients at risk receiving either lower IV fluid (E) vs higher IV fluid (C), respectively. One patient might be included more than once due to many SARs or SAEs.

### **6.2.2a Meta-analysis and forest plot of** highest proportion of SAE including mortality


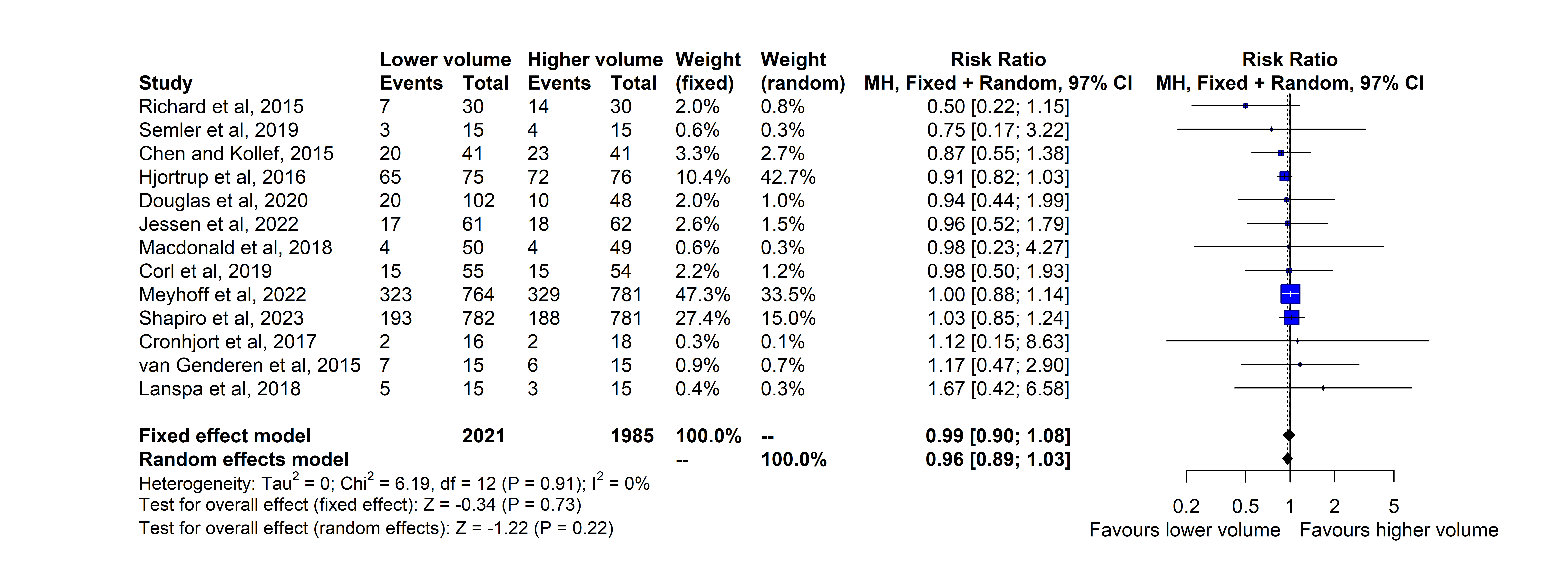


Meta-analysis of highest proportion of serious adverse reactions or events including mortality in 13 trials cf. e-Table 5S**.**

### 6.2.2b TSA **of** highest proportion of SAE including mortality

Conventional monitoring boundary for harm

Trial sequential monitoring boundary for harm

Trial sequential monitoring boundary for benefit

Area of futility

Favors

Higher fluid volumes

Favors

Lower fluid volumes

No. of patients

Required information size = 3758

Required information size is a Two-sided graph

Conventional monitoring boundary for benefit

**
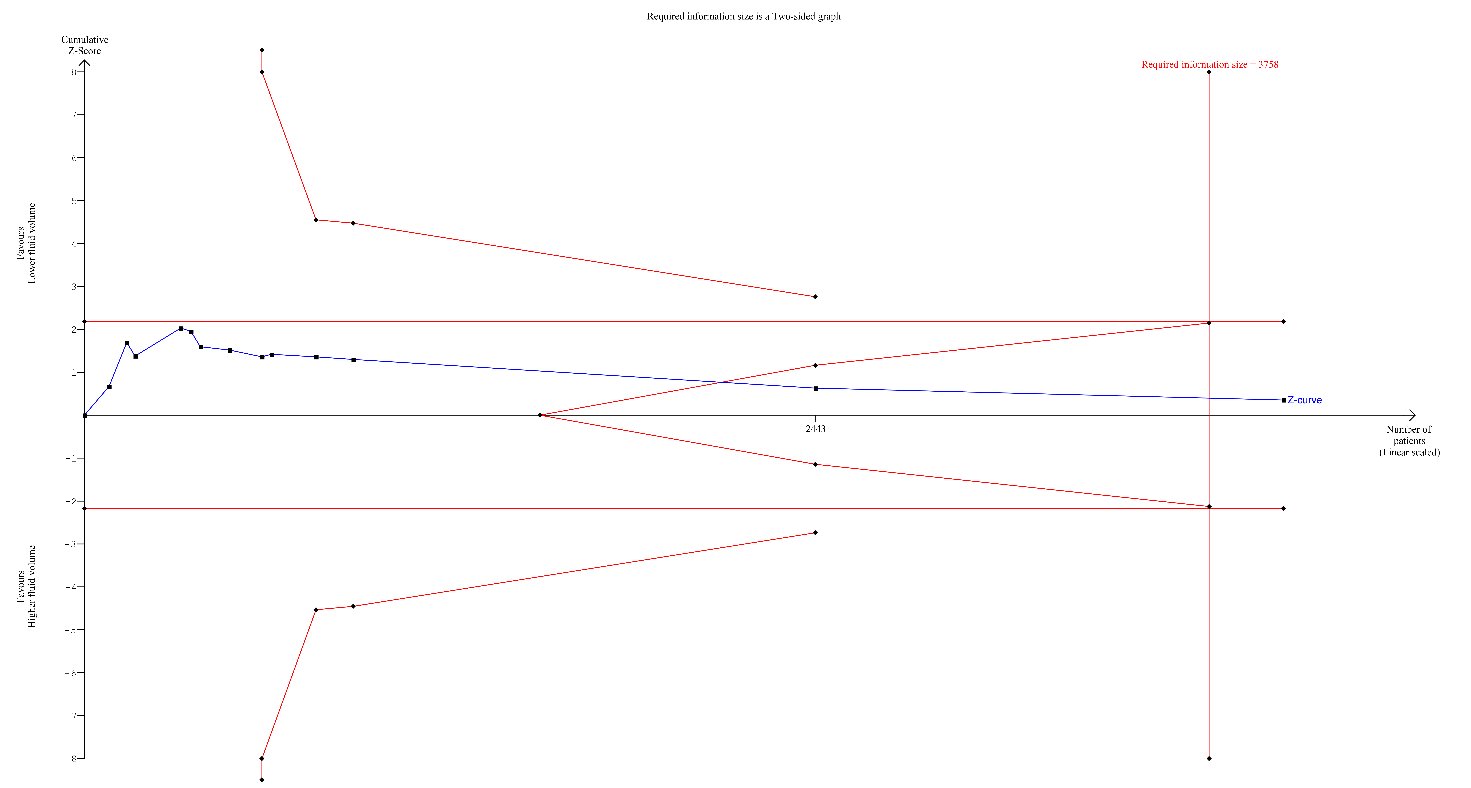
**

Trial sequential analysis (TSA) of the highest proportion of serious adverse events including mortality in 13 trials. We used a control event proportion of 34.7 %, alpha 3.3% (two-sided), beta 10% (power 90%), heterogeneity adjustment of 0%, and an a priori relative risk reduction of 15% in the analysis. The blue cumulative Z-curve crossed the area of futility and required information size was reached. Thus, the TSA is conclusive and a relative risk reduction of 15% is unlikely. The TSA-adjusted CI is identical to the unadjusted CI as accrued information size is larger than the required information size.

### 6.2.c Funnel plot **of h**ighest proportion of SAE including mortality

**
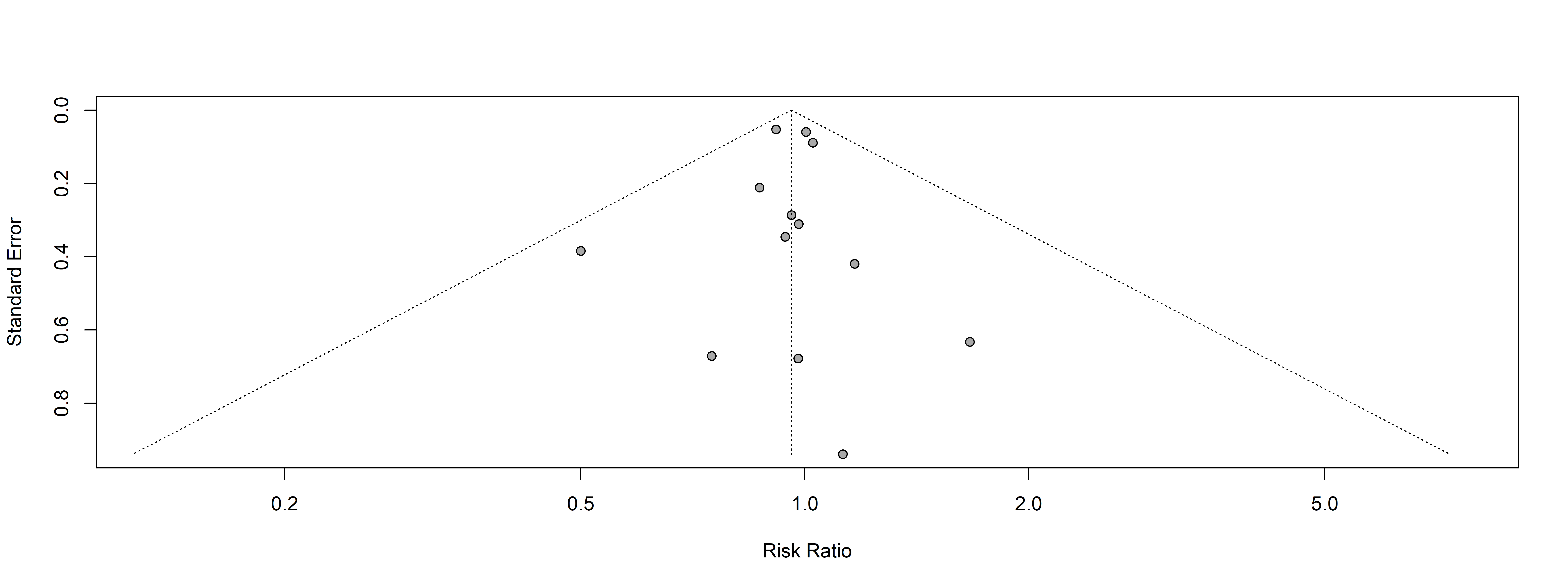
**

Asymmetry was tested with Harbord test (P=0.38).

### 6.2.3 e-**Table 3** Serious adverse events (cumulated)

|  | **Total SAE events** | | | |
| --- | --- | --- | --- | --- |
| **Trial** | E: No. events | E: No. at risk | C: No. events | C: No. at risk |
| Chen and Kollef^4^ | 20 | 41 | 23 | 41 |
| Cronhjort et al.^5^ | 4 | 16 | 2 | 18 |
| Hjortrup et al.^6^ | 93 | 75 | 112 | 76 |
| Lanspa et al. ^10^ | 5 | 15 | 3 | 15 |
| Macdonald et al. ^9^ | 8 | 50 | 6 | 49 |
| Richard et al. ^7^ | 7 | 30 | 14 | 30 |
| Semler et al. ^11^ | 3 | 15 | 4 | 15 |
| Van Genderen et al.^8^ | 7 | 15 | 6 | 15 |
| Corl et al.^12^ | 20 | 55 | 19 | 54 |
| Douglas et al. ^13^ | 27 | 102 | 15 | 48 |
| Jessen et al.^14^ | 26 | 61 | 32 | 62 |
| Meyhoff et al.^15^ | 608 | 764 | 628 | 781 |
| Shapiro et al.^16^ | 214 | 782 | 207 | 781 |

SAE: serious adverse events, E: experimental; C: control; No.: number.

The highest proportion of events are the numbers included in the following meta-analysis and trial sequential analysis. One patient might be included more than once due to many events.

### 6.2.3 e-Table 4 Serious adverse events (cumulated) - specified

|  | | **All-cause mortality** | | | | | | ***Prop. of pts with SAR to IV crystalloids or vasopressors** | | | | | | **Prop. of pts with catheter related complications** | | | | | | **Prop. of pts with complications to fluid overload** | | | |
| --- | --- | --- | --- | --- | --- | --- | --- | --- | --- | --- | --- | --- | --- | --- | --- | --- | --- | --- | --- | --- | --- | --- | --- |
| **Trial** | | | E: No. events | E: No.  at risk | C: No. events | C: No.  at risk | | | E: No. events | E: No.  at risk | C: No. events | C: No.  at risk | | | E: No. events | E: No.  at risk | C: No. events | | C: No.  at risk | E: No. events | E: No.  at risk | C: No. events | C: No.  at risk |
| Chen and Kollef^4^ | | | 20 | 41 | 23 | 41 | | |  |  |  |  | | |  |  |  | |  |  |  |  |  |
| Cronhjort et al.^5^ | | | 2 | 16 | 2 | 18 | | |  |  |  |  | | | 2 | 16 | 0 | | 18 |  |  |  |  |
| Hjortrup et al.^6^ | | | 25 | 75 | 31 | 76 | | | 65 | 75 | 72 | 76 | | |  |  |  | |  |  |  |  |  |
| Lanspa et al. ^10^ | | | 5 | 15 | 3 | 15 | | |  |  |  |  | | |  |  |  | |  |  |  |  |  |
| Macdonald et al. ^9^ | | | 4 | 48 | 3 | 47 | | |  |  |  |  | | | 2 | 50 | 1 | | 49 | 1 | 50 | 1 | 49 |
| Richard et al. ^7^ | | | 7 | 30 | 14 | 30 | | |  |  |  |  | | |  |  |  | |  |  |  |  |  |
| Semler et al. ^11^ | | | 3 | 15 | 4 | 15 | | |  |  |  |  | | |  |  |  | |  |  |  |  |  |
| Van Genderen et al.^8^ | | | 7 | 15 | 6 | 15 | | |  |  |  |  | | |  |  |  | |  |  |  |  |  |
| Corl et al.^12^ | | | 15 | 55 | 15 | 54 | | |  |  |  |  | | |  |  |  | |  |  |  |  |  |
| Douglas et al. ^13^ | | | 20 | 102 | 10 | 48 | | |  |  |  |  | | |  |  |  | |  |  |  |  |  |
| Jessen et al.^14^ | | | 12 | 61 | 15 | 60 | | | 0 | 61 | 0 | 62 | | |  |  |  | |  | 4 | 61 | 5 | 62 |
| Meyhoff et al.^15^ | | | 323 | 764 | 329 | 781 | | | 31 | 755 | 32 | 776 | | |  |  |  | |  |  |  |  |  |
| Shapiro et al.^16^ | | | 172 | 768 | 169 | 773 | | |  |  |  |  | | | 18 | 208 | 12 | | 155 | 0^a^ | 782 | 11 | 781 |
| **Continued** | **Prop. of pts with ischemic events** | | | | | | **Acute kidney injury** | | | | | | **Respiratory adverse events** | | | | | **Other adverse events .** | | | | | |
| Chen and Kollef^4^ | | |  |  |  |  | | |  |  |  |  | | |  |  |  | |  |  |  |  |  |
| Cronhjort et al.^5^ | | |  |  |  |  | | |  |  |  |  | | |  |  |  | |  |  |  |  |  |
| Hjortrup et al.^6^ | | | 3 | 75 | 9 | 76 | | |  |  |  |  | | |  |  |  | |  |  |  |  |  |
| Lanspa et al. ^10^ | | |  |  |  |  | | |  |  |  |  | | |  |  |  | |  |  |  |  |  |
| Macdonald et al. ^9^ | | | 1 | 50 | 1 | 49 | | |  |  |  |  | | |  |  |  | |  |  |  |  |  |
| Richard et al. ^7^ | | |  |  |  |  | | |  |  |  |  | | |  |  |  | |  |  |  |  |  |
| Semler et al. ^11^ | | |  |  |  |  | | |  |  |  |  | | |  |  |  | |  |  |  |  |  |
| Van Genderen et al.^8^ | | |  |  |  |  | | |  |  |  |  | | |  |  |  | |  |  |  |  |  |
| Corl et al.^12^ | | | 3^b^ | 55 | 0 | 54 | | | 1 | 55 | 1 | 54 | | | 1^c^ | 55 | 3 | | 54 | 0^d^ | 55 | 0 | 54 |
| Douglas et al. ^13^ | | | 7^e^ | 98 | 5 | 45 | | |  |  |  |  | | |  |  |  | |  |  |  |  |  |
| Jessen et al.^14^ | | | 1 | 61 | 2 | 62 | | | 9 | 61 | 10 | 62 | | |  |  |  | |  |  |  |  |  |
| Meyhoff et al.^15^ | | | 81 | 755 | 78 | 776 | | | 173 | 750 | 189 | 772 | | |  |  |  | |  |  |  |  |  |
| Shapiro et al.^16^ | | | 4^f^ | 782 | 1 | 781 | | | 1 | 782 | 0 | 781 | | | 2^g^ | 782 | 4 | | 781 | 17^h^ | 782 | 10 | 781 |

Prop.: proportion; pts: patients, SAR: serious adverse reaction, E: experimental; C: control; No.: number.

Types of serious adverse reactions and events and adverse events from each trial. The cells are left blank if data was not available from the original trial. Cumulated number of events are reported in e-Table 6S.

* The proportion of patients are reported as the number of patients with one or more SARs out of the total no of patients at risk receiving either lower IV fluid (E) vs higher IV fluid (C), respectively. One patient might be included more than once due to many events.

^a^ Shapiro et al. reported adverse events due to fluid overload (E: 0 events, C: 6 events) incl. transfusion associated circulatory overload (E: 0 events, C: 1 events) and pulmonary edema (E: 0 events, C: 3 events).

^b^ Corl et al. reported myocardial infarction (E: 3 events, C: 0 events) and limb ischemia (0 events in each group), ^c^ requirement for reintubation (E: 1 event, C: 1 event) and ^d^disseminated intravascular coagulation (E: 0 events, C: 0 events).

^e^ Douglas et al. reported major adverse cardiac events, but this was not further specified. (E: 7 events, C: 5 events)

^f^ Shapiro et al. reported myocardial infarction (E: 0 events, C: 1 events), cerebral infarct (E: 1 events, C: 0 events) and peripheral ischemia (E: 3 events, C: 0 events); ^g^ hypoxia (E: 0 events, C: 1 events); or worsening of hypoxia(E: 0 events, C: 1 events), pneumothorax (E: 1 event, C: 0 events), respiratory failure (E: 0 events, C: 1 events), shortness of breath (E: 0 events, C: 1 events); and ^h^ other adverse events covers all other specific adverse events during hospitalization in the appendix table S13 such as blood and lymphatic disorders, cardiac disorders, gastrointerstinal and hepatobiliary disorders, infectious and metabolic disorders, musculosketal and nervous system disorders, renal disorders, skin and vascular disorders.

### 6.2.3 a Meta-analysis and forest plot of serious adverse events (cumulated)


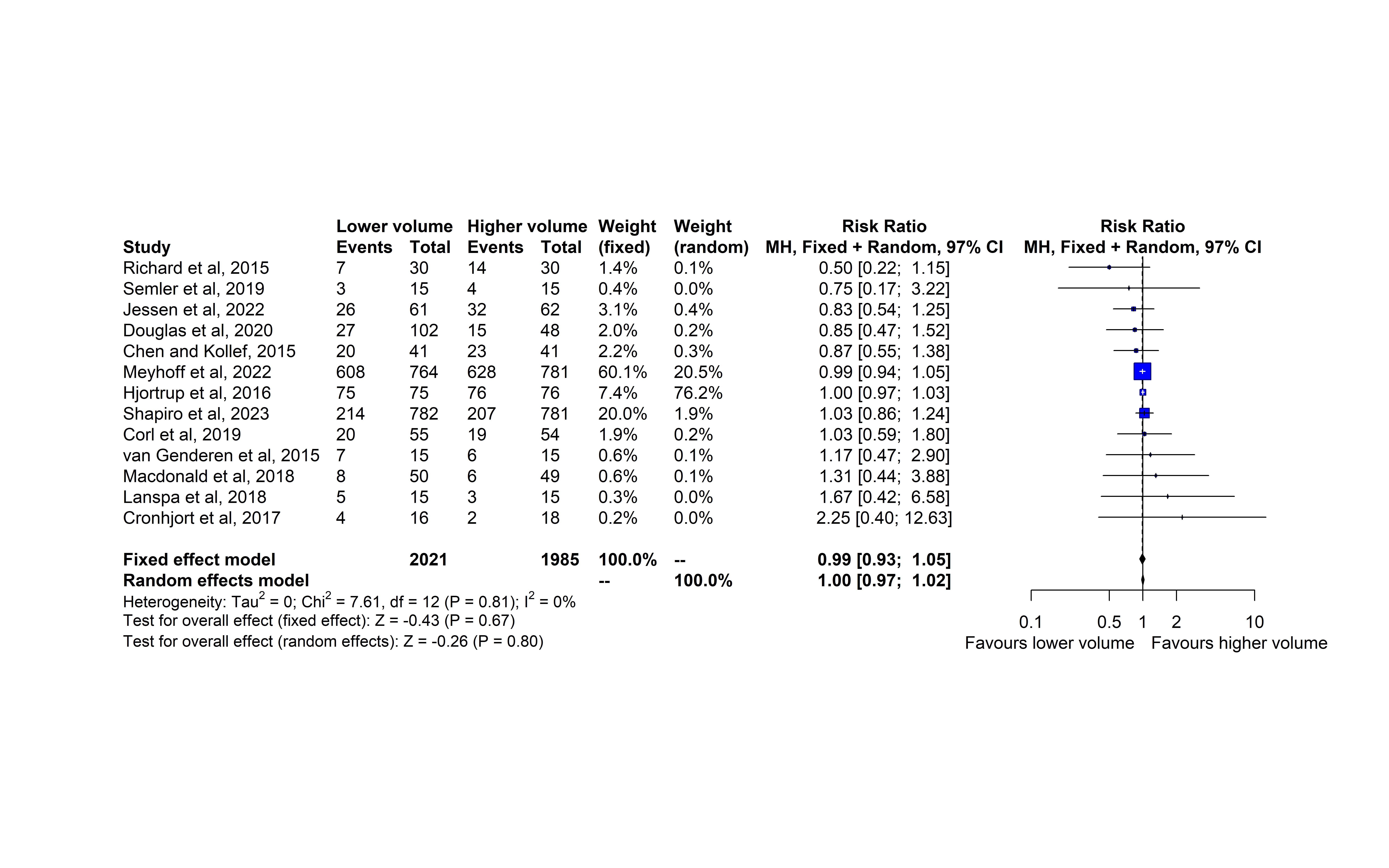


Meta-analysis of 13 trials reporting serious adverse reactions and events and adverse events cf. e-Table 6S**.**

### 6.2.3 b TSA of serious adverse events (cumulated)

Conventional monitoring boundary for harm

Trial sequential monitoring

boundary for harm

Trial sequential monitoring

boundary for benefit

Area of futility

Favors

Higher fluid volumes

Favors

Lower fluid volumes

No. of patients

Required information size = 1904

Required information size is a Two-sided graph

Conventional monitoring boundary for benefit


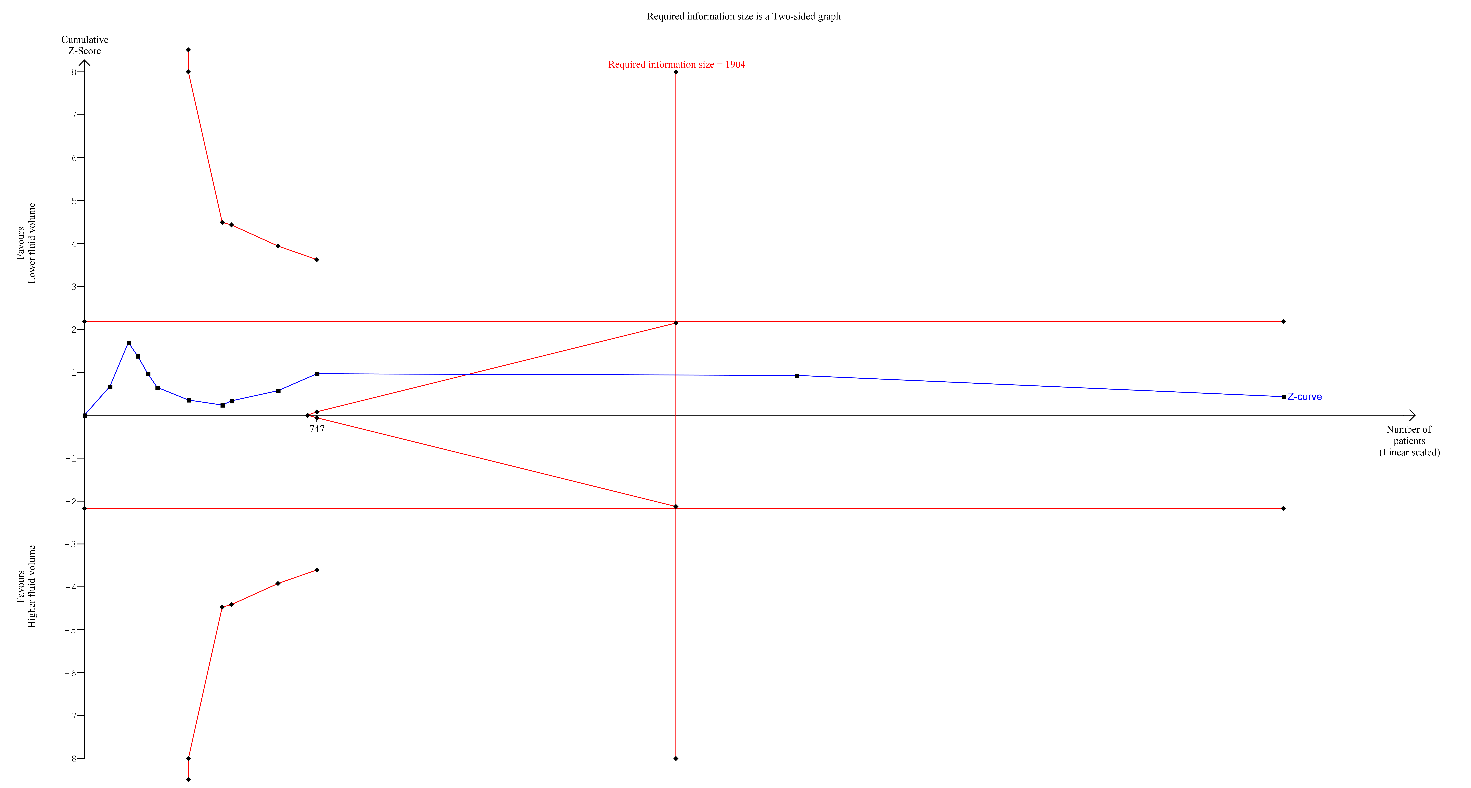


747

Trial sequential analysis (TSA) of the cumulated numbers of serious adverse reactions and events including mortality in 13 trials. We used a control event proportion of 52.1 %, alpha 3.3% (two-sided), beta 10% (power 90%), model variance-based heterogeneity adjustment, and an a priori relative risk reduction of 15% in the analysis. The blue cumulative Z-curve crossed the area of futility and required information size was reached. Thus, the TSA is conclusive and a relative risk reduction of 15% is unlikely. The TSA-adjusted CI is identical to the unadjusted CI as accrued information size is larger than the required information size.

### 6.2.3 c Funnel plot of serious adverse events (cumulated)


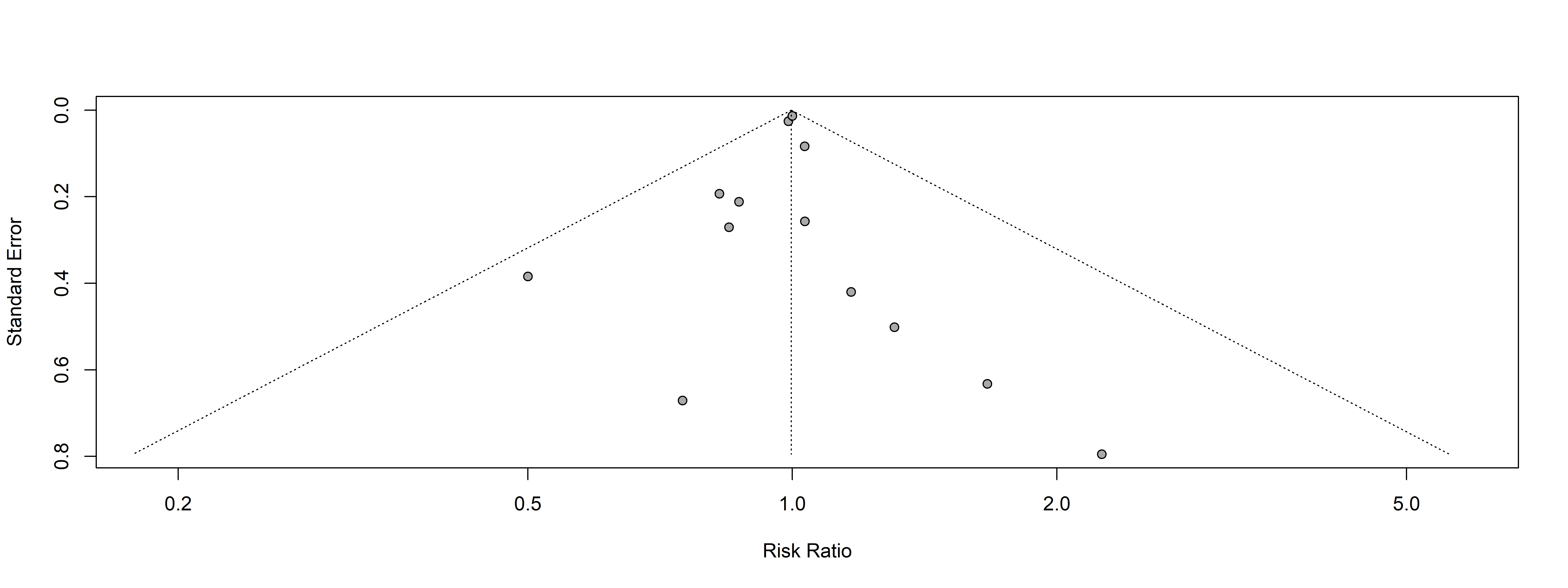


Asymmetry was tested with Harbord test (P=0.81).

# **7. e-Appendix 7 Analyses of secondary outcomes**

## 7.1 Duration of mechanical ventilation - meta-analysis and TSA

### 7.1.1 Meta-analysis and forest plot of duration of mechanical ventilation


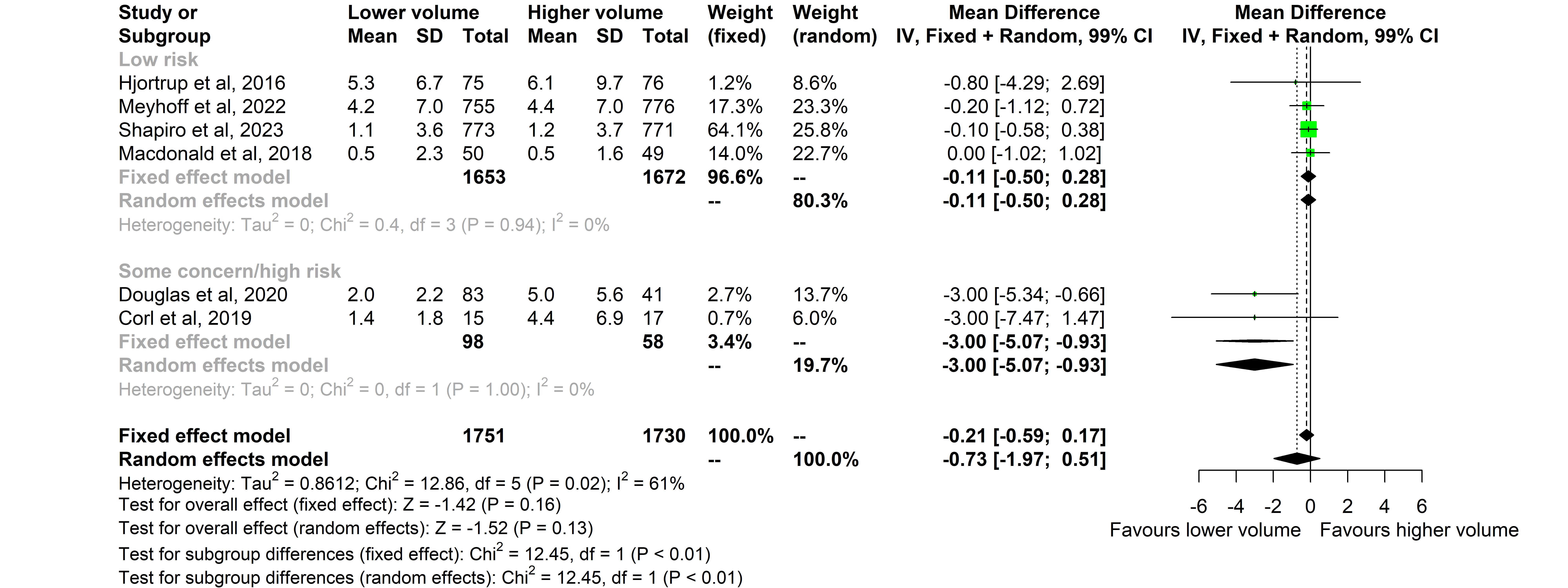


### 7.1.2 TSA of duration of mechanical ventilation (low RoB trials only)

Conventional monitoring boundary for harm

Trial sequential monitoring

boundary for harm

Trial sequential monitoring

boundary for benefit

Area of futility

Favors

Higher fluid volumes

Favors

Lower fluid volumes

250

No. of patients

Required information size = 1329

Required information size is a Two-sided graph

Conventional monitoring boundary for benefit


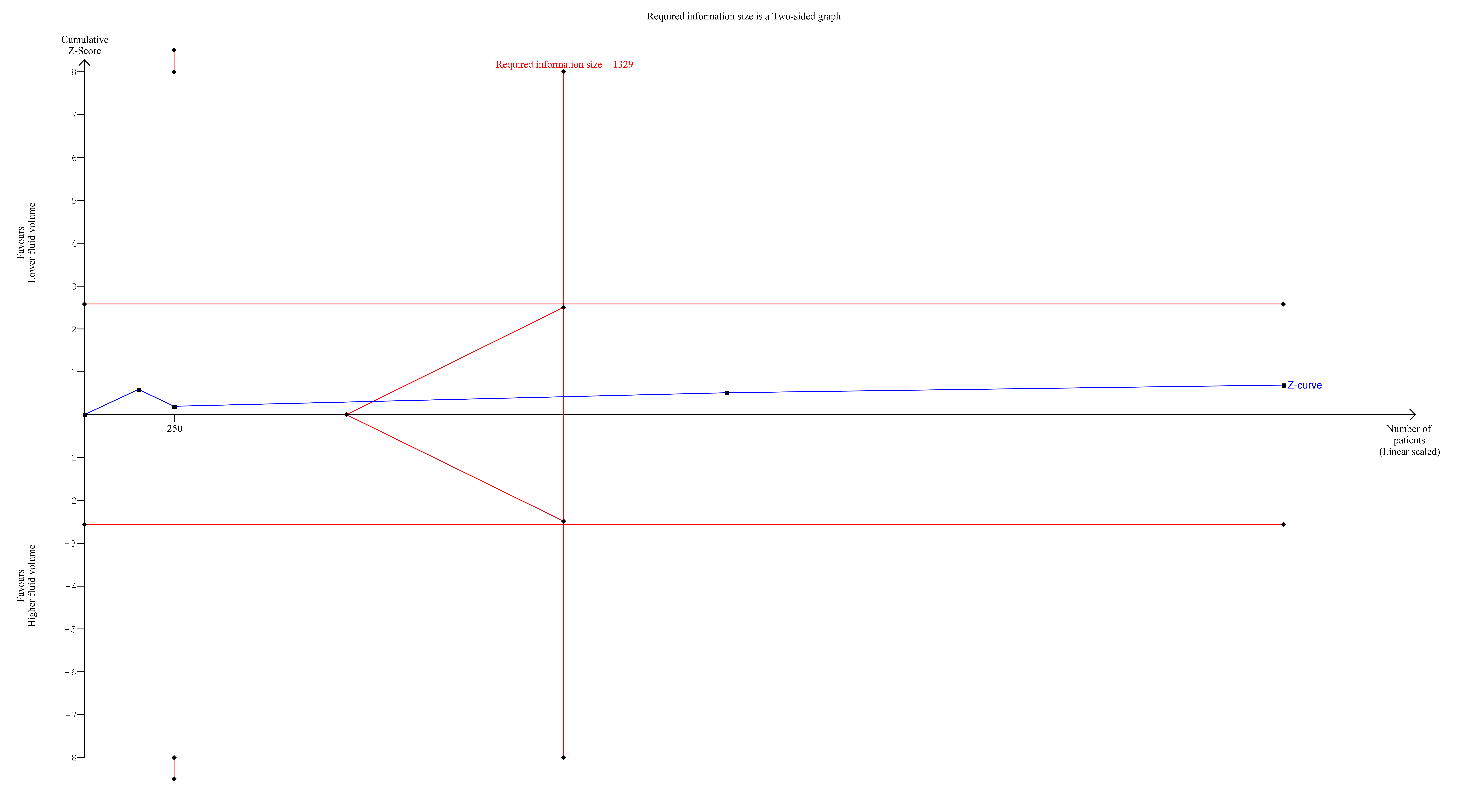


Trial sequential analysis (TSA) of duration of mechanical ventilation in three low risk of bias trials. We used an alpha of 1.25% (two-sided), beta of 10% (power 90%), model variance-based heterogeneity adjustment, and an a priori mean difference (MD) of one day in the analysis. The blue cumulative Z-curve crossed the area of futility and required information size was reached. Thus, the TSA is conclusive and a predefined MD of one day is unlikely. The TSA-adjusted CI is identical to the unadjusted CI as accrued information size is larger than the required information size. The diversity was D^2^=18%.

### 7.1.3 TSA of duration of mechanical ventilation (all trials)

Conventional monitoring boundary for harm

Trial sequential monitoring boundary for harm

Trial sequential monitoring boundary for benefit

Area of futility

Favors

Higher fluid volumes

Favors

Lower fluid volumes

3481

No. of patients

Required information size = 15579

Required information size is a Two-sided graph

Conventional monitoring boundary for benefit


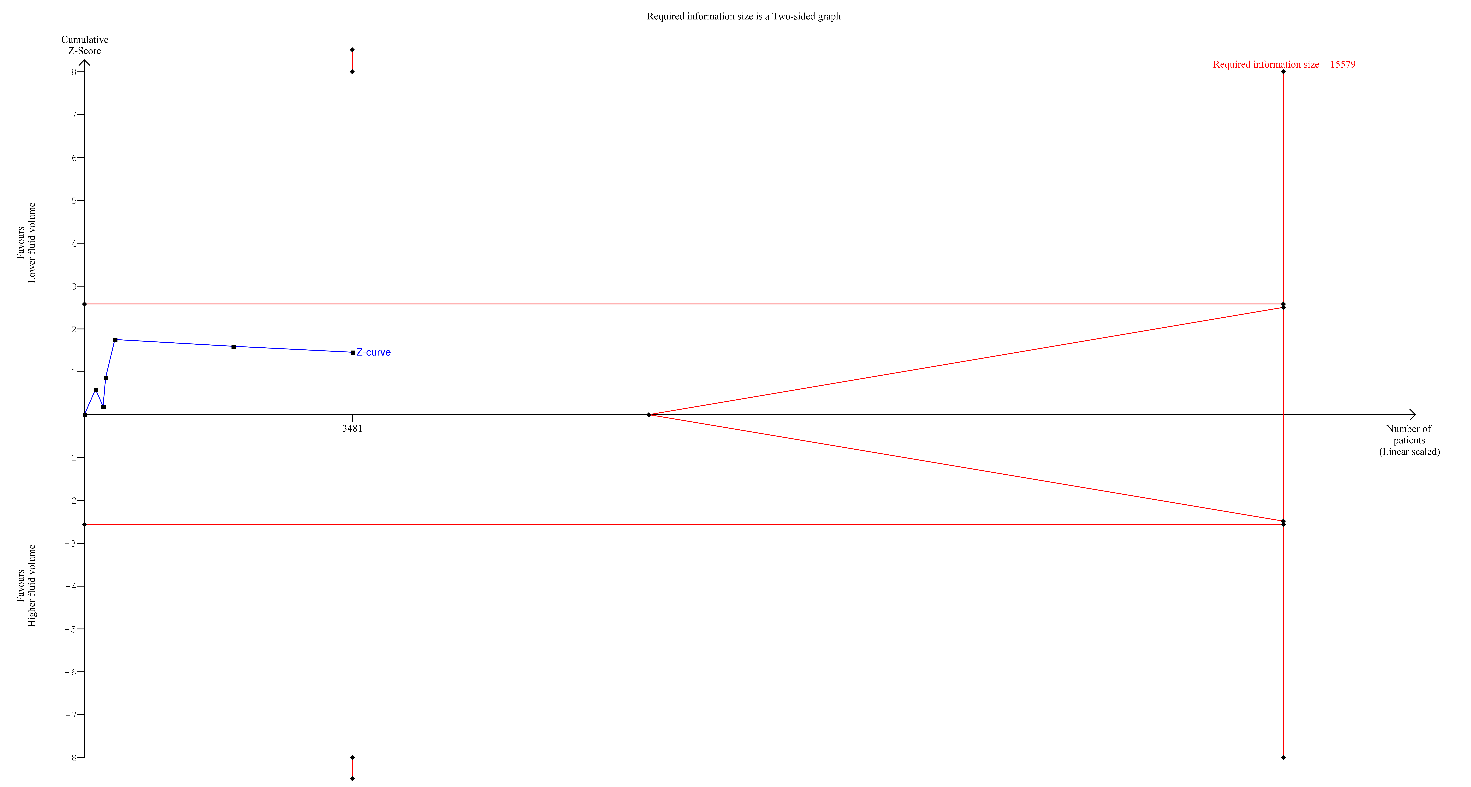


Trial sequential analysis (TSA) of duration of mechanical ventilation in five trials. We used an alpha of 1.25% (two-sided), beta of 10% (power 90%), model variance-based heterogeneity adjustment, and an a priori mean difference (MD) of one day in the analysis. The TSA adjusted confidence interval in the random effects model was -5.29 to 3.66 with a diversity D^2^=93%. The blue cumulative Z-curve did not cross the conventional monitoring boundaries for benefit, harm or futility; thus, the TSA is inconclusive. 22% (3481 patients) of the required information size of 15579 patients was accrued.

## 7.2 Ventilator-free days - meta-analysis and TSA

### 7.2.1 Meta-analysis and forest plot of ventilator-free days


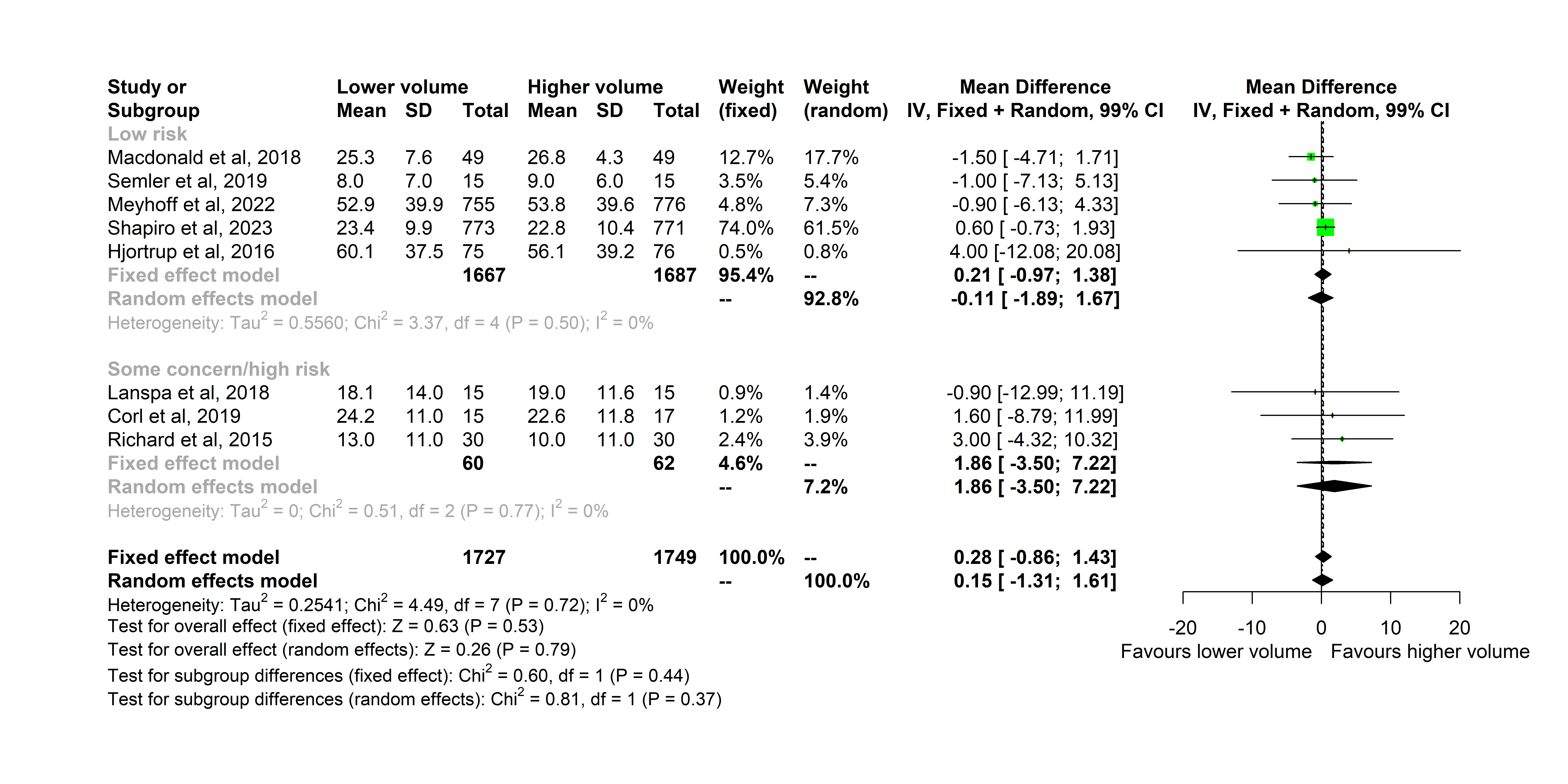


### 7.2.1 TSA of ventilator-free days (low RoB trials only)

Conventional monitoring boundary for harm

Trial sequential monitoring boundary for harm

Trial sequential monitoring boundary for benefit

Area of futility

Favors

Higher fluid volumes

Favors

Lower fluid volumes

3354

No. of patients

Required information size = 9916

Required information size is a Two-sided graph

Conventional monitoring boundary for benefit


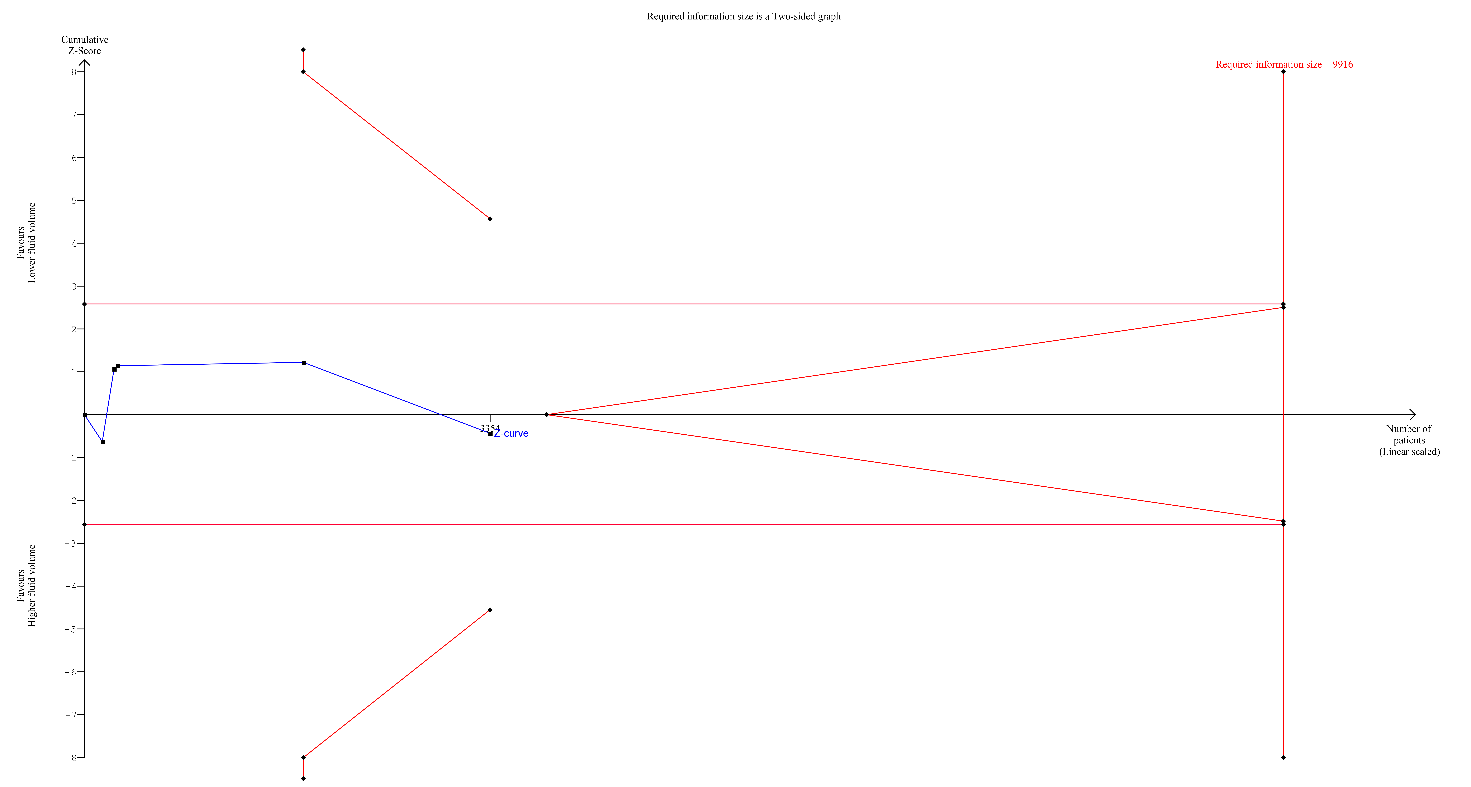


Trial sequential analysis (TSA) of ventilator-free days in five low risk of bias trials. We used an alpha of 1.25% (two-sided), beta of 10% (power 90%), heterogeneity adjustment of 0%, and an a priori mean difference (MD) of one day in the analysis. The TSA adjusted confidence interval in the fixed effect model was -1.86 to 2.27 with a diversity D^2^=0%. The blue cumulative Z-curve did not cross the conventional monitoring boundaries for benefit, harm or futility; thus, the TSA is inconclusive. 34% (3354 patients) of the required information size of 9916 patients was accrued.

### 7.2.2 TSA of ventilator-free days (all trials)

Conventional monitoring boundary for harm

Trial sequential monitoring boundary for harm

Trial sequential monitoring boundary for benefit

Area of futility

Favors

Higher fluid volumes

Favors

Lower fluid volumes

3476

No. of patients

Required information size = 9808

Required information size is a Two-sided graph

Conventional monitoring boundary for benefit


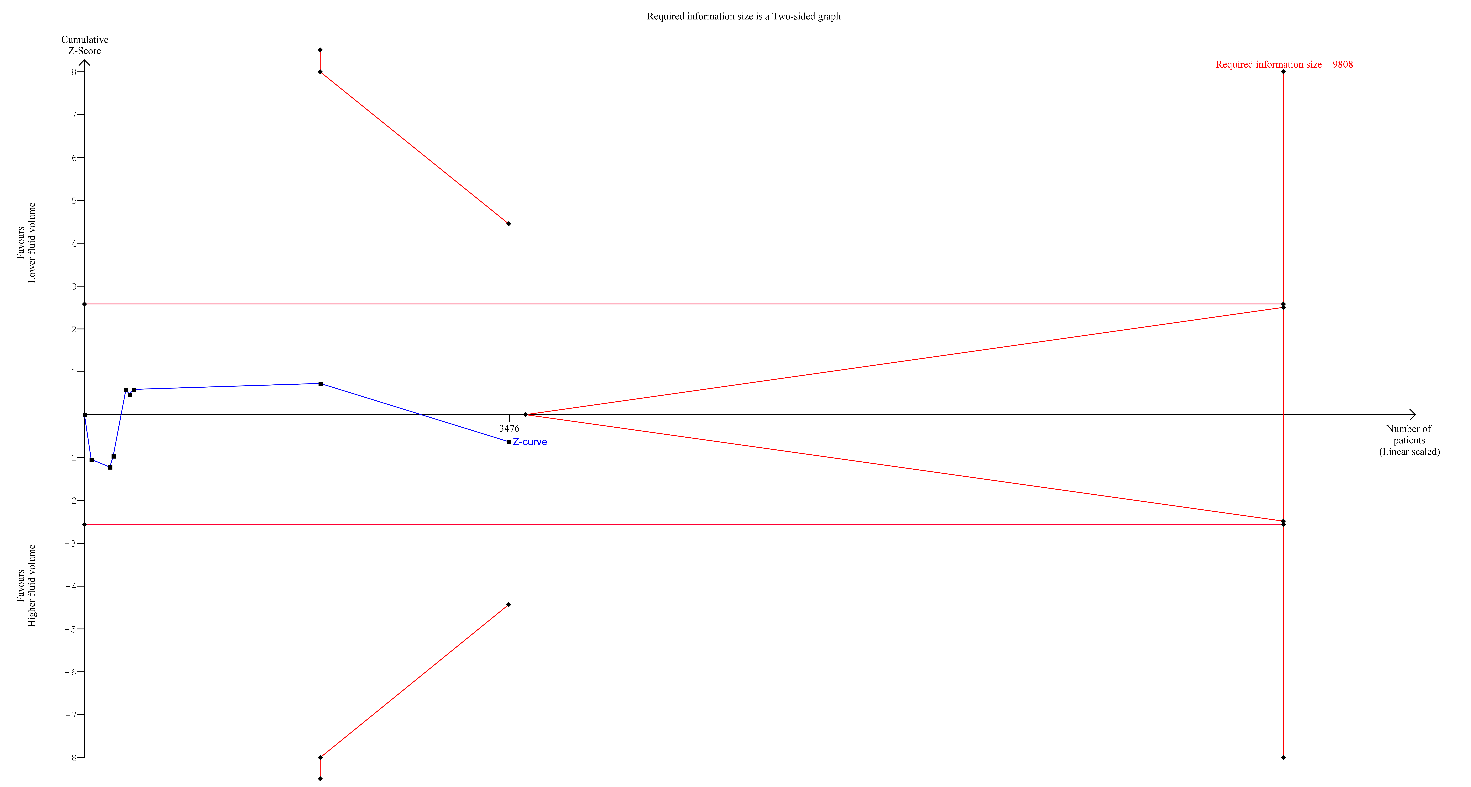


Trial sequential analysis (TSA) of ventilator-free days in seven trials. We used an alpha of 1.25% (two-sided), beta of 10% (power 90%), heterogeneity adjustment of 0%, and an a priori mean difference (MD) of one day in the analysis. The TSA adjusted confidence interval in the fixed effect model was -1.66 to 2.23 with a diversity D^2^=0%. The blue cumulative Z-curve did not cross the conventional monitoring boundaries for benefit, harm or futility; thus, the TSA is inconclusive. 35% (3476 patients) of the required information size of 9808 patients was accrued.

## 7.3 Duration of vasopressor or inotropes-meta-analysis and TSA

### 7.3.1 Meta-analysis and forest plot of duration of vasopressor or inotropes


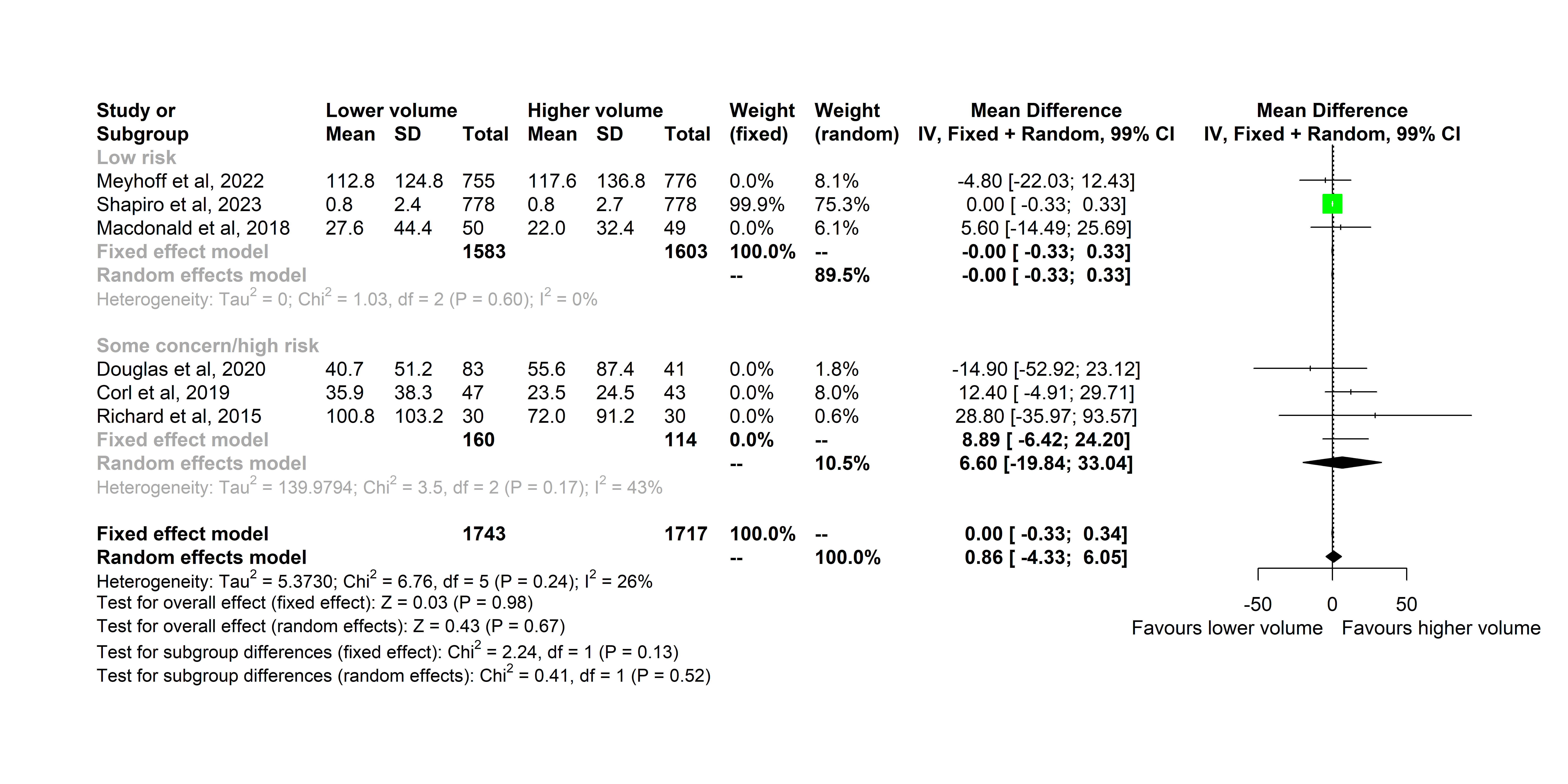


### 7.3.2 TSA of duration of vasopressor or inotropes (low RoB trials only)

Conventional monitoring boundary for harm

Trial sequential monitoring boundary for harm

Trial sequential monitoring boundary for benefit

Area of futility

Favors

Higher fluid volumes

Favors

Lower fluid volumes

No. of patients

Required information size is a Two-sided graph

Conventional monitoring boundary for benefit


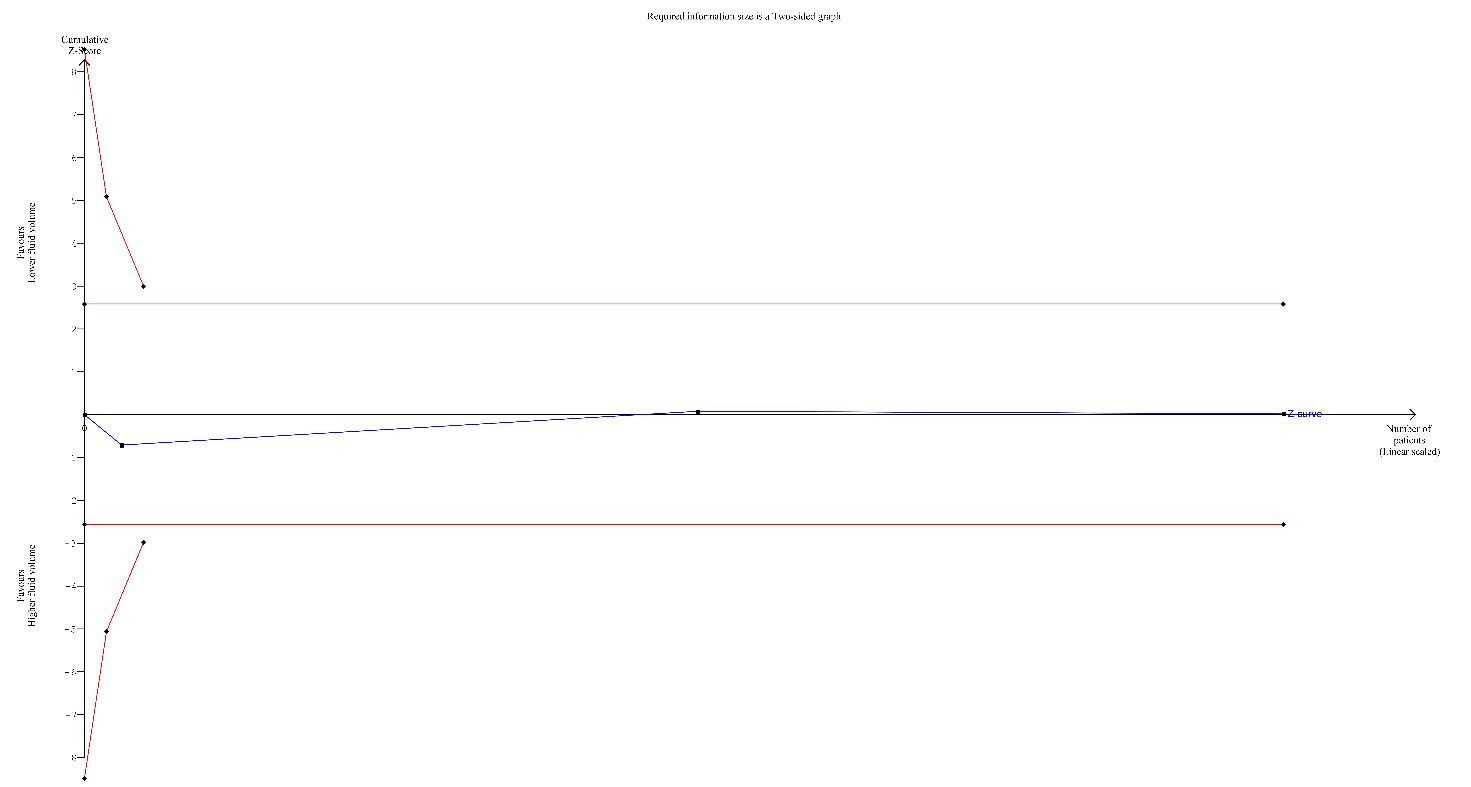


Trial sequential analysis (TSA) of duration of vasopressor in three low risk of bias trials. We used an alpha of 1.25% (two-sided), beta of 10% (power 90%), heterogeneity adjustment of 0%, and an a priori mean difference (MD) of 24 hours in the analysis. The blue cumulative Z-curve crossed the area of futility and required information size was reached. Thus, the TSA is conclusive and a predefined MD of one day is unlikely. The TSA-adjusted CI is identical to the unadjusted CI as accrued information size is larger than the required information size. The diversity was D^2^=0%.

### 7.3.3 TSA of duration of vasopressor or inotropes (all trials)

Conventional monitoring boundary for harm

Trial sequential monitoring boundary for harm

Trial sequential monitoring boundary for benefit

Area of futility

Favors

Higher fluid volumes

Favors

Lower fluid volumes

No. of patients

Required information size is a Two-sided graph

Conventional monitoring boundary for benefit


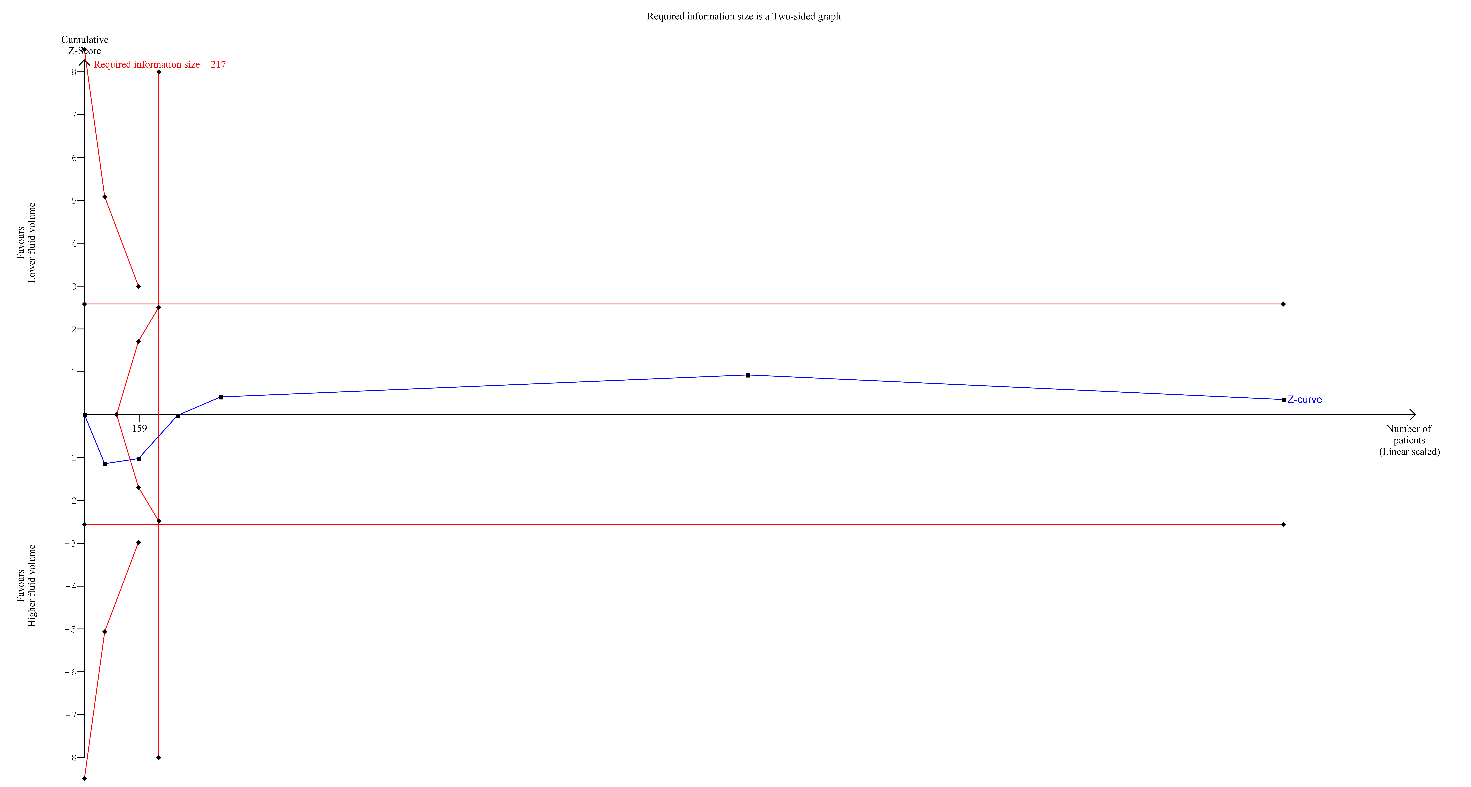


Required information size = 217

Trial sequential analysis (TSA) of duration of vasopressor in six trials. We used an alpha of 1.25% (two-sided), beta of 10% (power 90%), heterogeneity adjustment of 0%, and an a priori mean difference (MD) of one day in the analysis. The blue cumulative Z-curve crossed the area of futility and required information size was reached. Thus, the TSA is conclusive and a predefined MD of one day is unlikely. The TSA-adjusted CI is identical to the unadjusted CI as accrued information size is larger than the required information size. The diversity was D^2^=0%.

## 7.4 Vasopressor-free days - meta-analysis and TSA

### 7.4.1 Meta-analysis and forest plot of vasopressor-free days


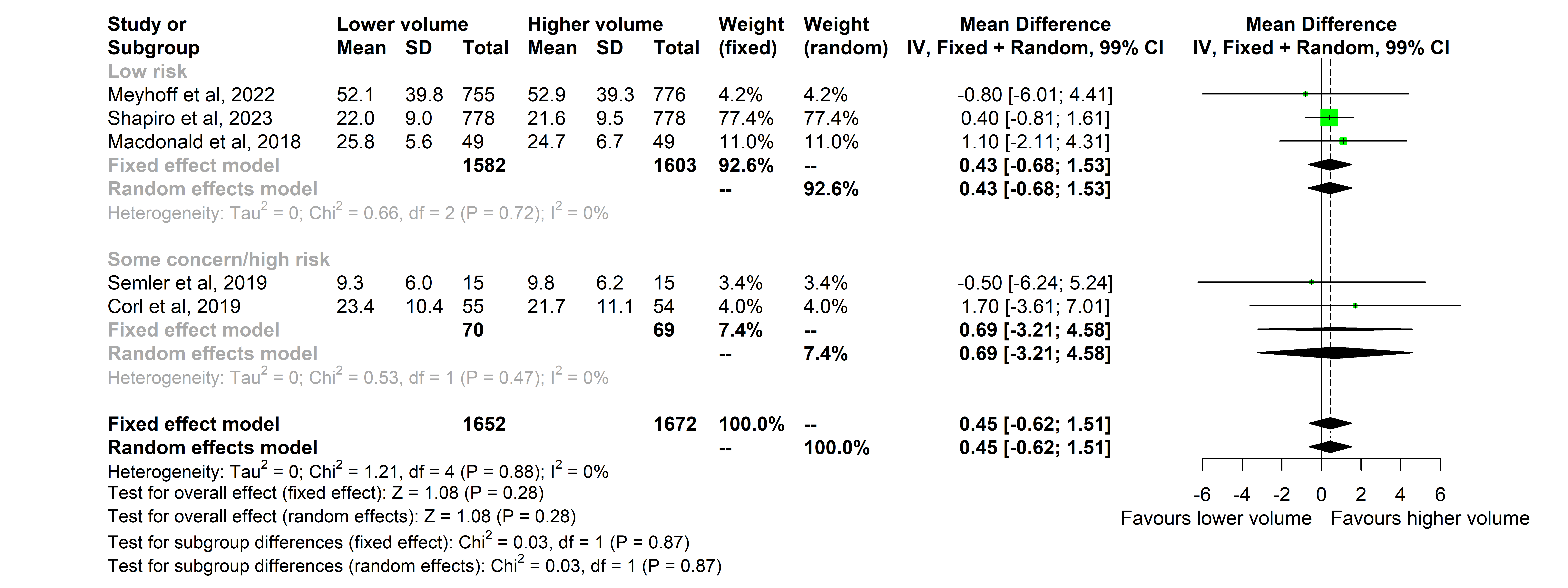


### 7.4.2 TSA of vasopressor-free days (low RoB trials only)

Conventional monitoring boundary for harm

Trial sequential monitoring boundary for harm

Trial sequential monitoring boundary for benefit

Area of futility

Favors

Higher fluid volumes

Favors

Lower fluid volumes

3185

No. of patients

Required information size = 8378

Required information size is a Two-sided graph

Conventional monitoring boundary for benefit


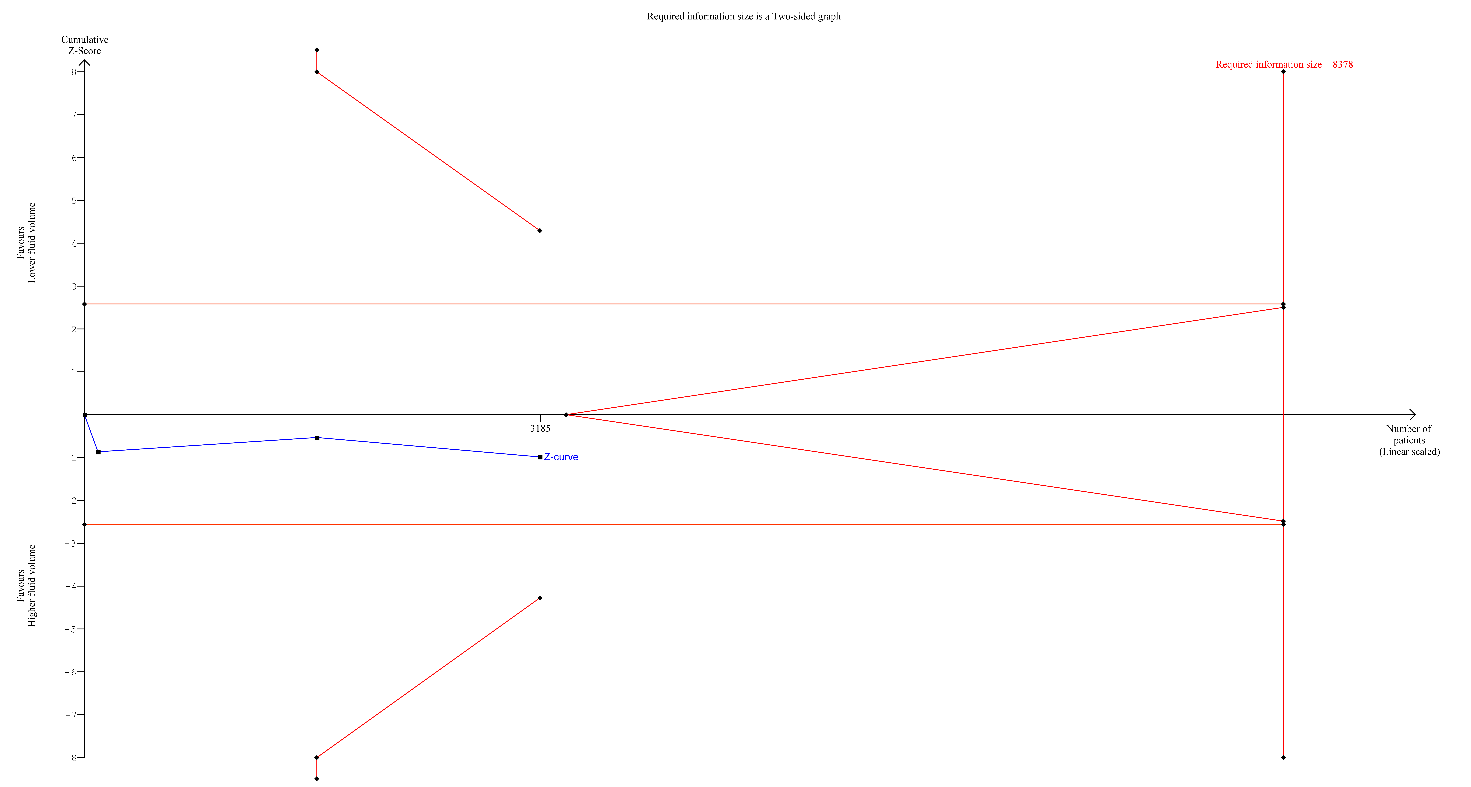


Trial sequential analysis (TSA) of vasopressor-free days in three low risk of bias trials. We used an alpha of 1.25% (two-sided), beta of 10% (power 90%), heterogeneity adjustment of 0%, and an a priori mean difference (MD) of one day in the analysis. The TSA adjusted confidence interval in the fixed effect model was -1.41 to 2.27 with a diversity D^2^=0%. The blue cumulative Z-curve did not cross the conventional monitoring boundaries for benefit, harm or futility; thus, the TSA is inconclusive. 38% (3185 patients) of the required information size of 8378 patients was accrued.

### 7.4.3 TSA of vasopressor-free days (all trials)

Conventional monitoring boundary for harm

Trial sequential monitoring boundary for harm

Trial sequential monitoring boundary for benefit

Area of futility

Favors

Higher fluid volumes

Favors

Lower fluid volumes

3324

No. of patients

Required information size = 8105

Required information size is a Two-sided graph

Conventional monitoring boundary for benefit


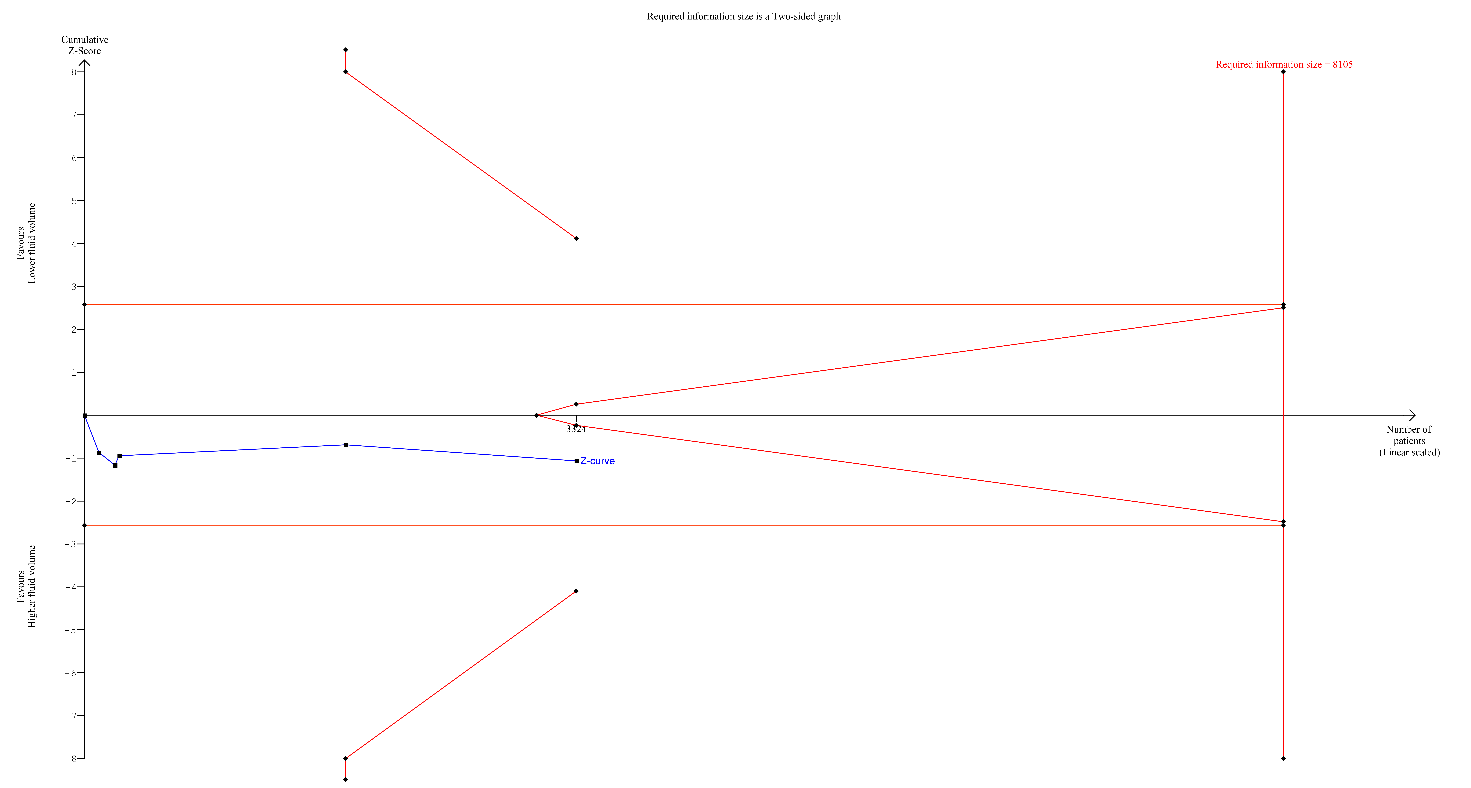


Trial sequential analysis (TSA) of vasopressor-free days in five trials. We used an alpha of 1.25% (two-sided), beta of 10% (power 90%), heterogeneity adjustment of 0%, and an a priori mean difference (MD) of one day in the analysis. The TSA adjusted confidence interval in the fixed effect model was -1.25 to 2.14 with a diversity D^2^=0%. The blue cumulative Z-curve did not cross the conventional monitoring boundaries for benefit, harm or futility; thus, the TSA is inconclusive. 41% (3324 patients) of the required information size of 8105 patients was accrued.

## 7.5 Use of renal replacement therapy - meta-analysis and TSA

### 7.5.1 Meta-analysis and forest plot of use of renal replacement therapy


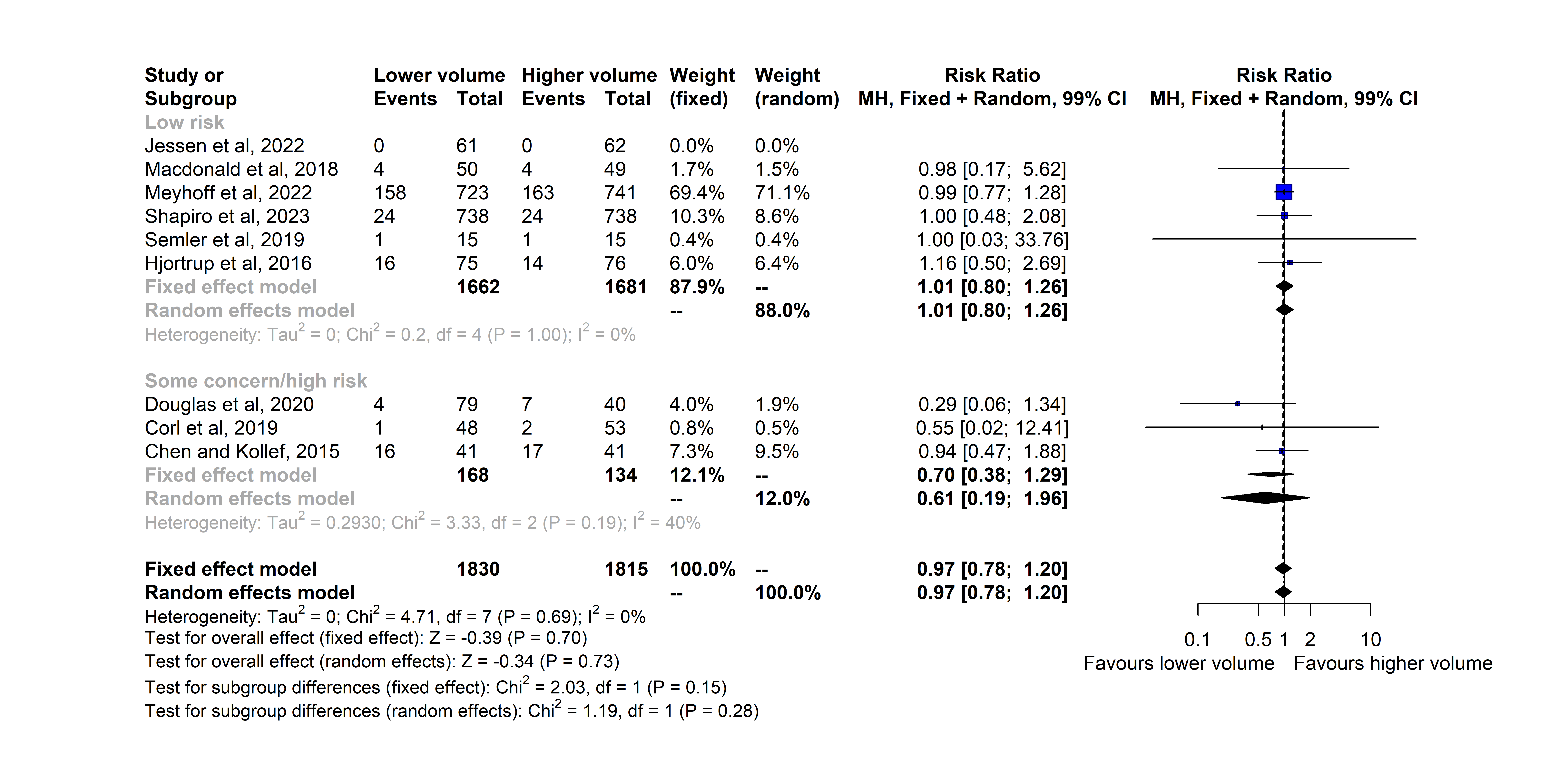


One zero-event trial not included in the meta-analysis^14^

### 7.5.2 TSA of use of renal replacement therapy (low RoB trials only)

Conventional monitoring boundary for harm

Trial sequential monitoring boundary for harm

Trial sequential monitoring boundary for benefit

Area of futility

Favors

Higher fluid volumes

Favors

Lower fluid volumes

3220

No. of patients

Required information size = 17019

Required information size is a Two-sided graph

Conventional monitoring boundary for benefit


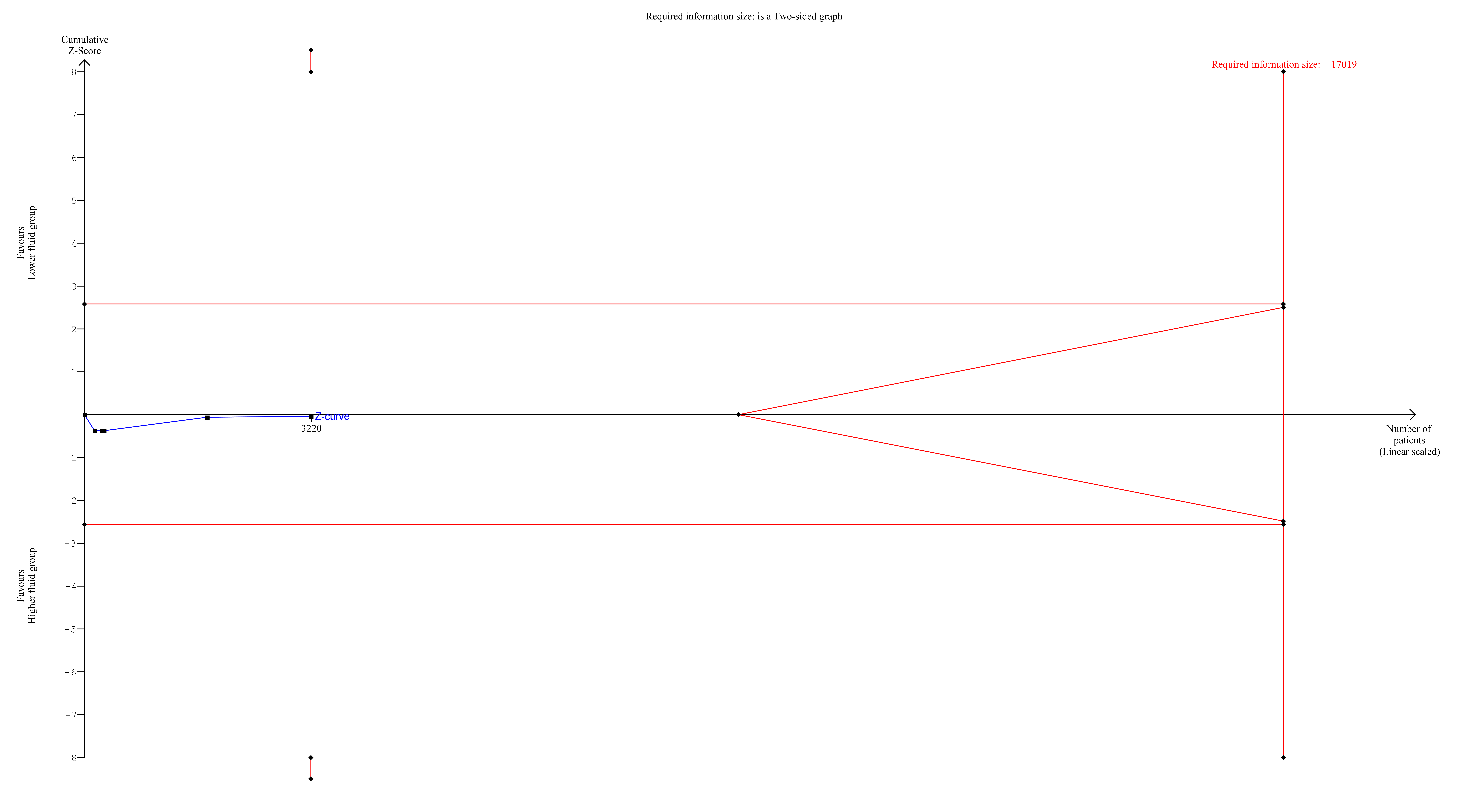


Trial sequential analysis (TSA) of the use of renal replacement therapy in six low risk of bias trials, but one zero-event trial not included in the TSA^14^. We used a control event proportion of 12.3%, alpha 1.25% (two-sided), beta 10% (power 90%), model-based variance heterogeneity adjustment, and an a priori relative risk reduction of 15% in the analysis. The TSA adjusted confidence interval in the random effects model was 0.49 to 2.04 with a diversity D^2^=0%. The blue cumulative Z-curve did not cross the conventional monitoring boundaries for benefit, harm or futility; thus, the TSA is inconclusive. 19% (3220 patients) of the required information size of 17019 patients was accrued.

### 7.5.3 TSA of use of renal replacement therapy (all trials)

Conventional monitoring boundary for harm

Trial sequential monitoring boundary for harm

Trial sequential monitoring boundary for benefit

Area of futility

Favors

Higher fluid volumes

Favors

Lower fluid volumes

3522

No. of patients

Required information size = 16177

Required information size is a Two-sided graph

Conventional monitoring boundary for benefit


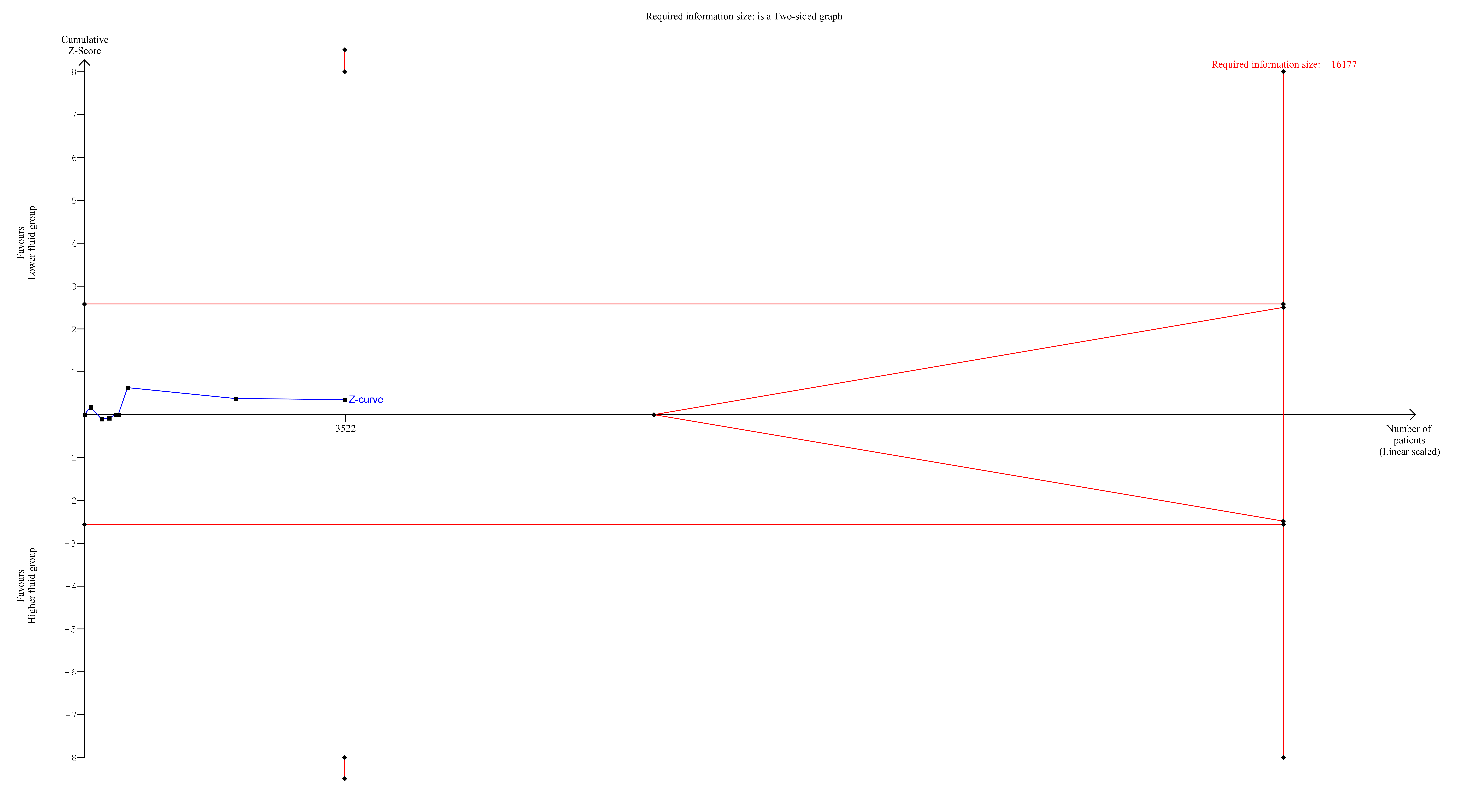


Trial sequential analysis (TSA) of the use of renal replacement therapy in eight trials. We used a control event proportion of 12.8%, alpha 1.25% (two-sided), beta 10% (power 90%), model-based variance heterogeneity adjustment and an a priori relative risk reduction of 15% in the analysis. The TSA adjusted confidence interval in the random effects model was 0.50 to 1.89 with a diversity D^2^=0%. The blue cumulative Z-curve did not cross the conventional monitoring boundaries for benefit, harm or futility; thus, the TSA is inconclusive. 22% (3522 patients) of the required information size of 16177 patients was accrued.

## 7.6 Duration of renal replacement therapy – meta-analysis and TSA

### 7.6.1 Meta-analysis and forest plot of duration of renal replacement therapy


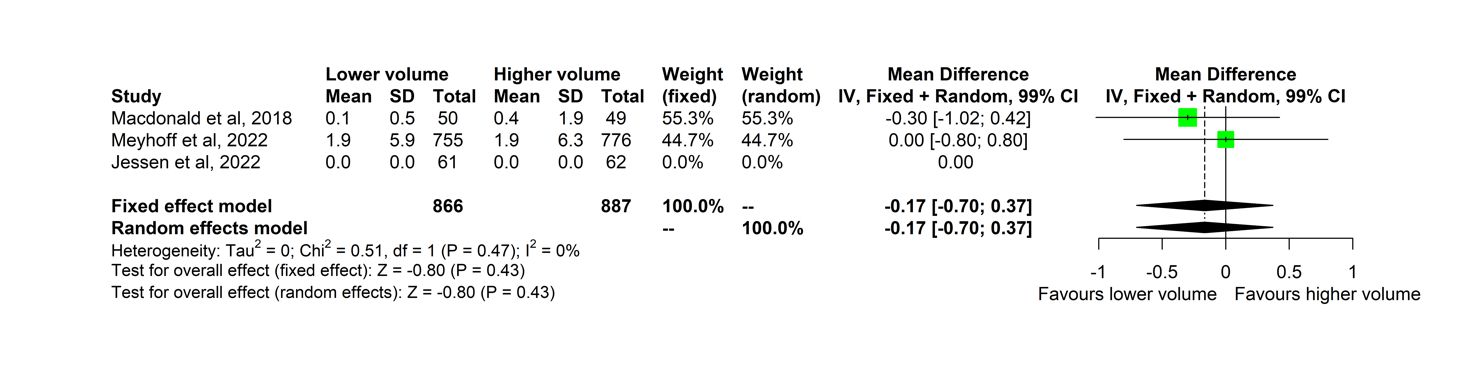


One zero-event trial not included in the meta-analysis^14^

### 7.6.2 TSA of duration of renal replacement therapy (two low RoB trials)

Conventional monitoring boundary for harm

Trial sequential monitoring boundary for harm

Trial sequential monitoring boundary for benefit

Area of futility

Favors

Higher fluid volumes

Favors

Lower fluid volumes

No. of patients

Required information size = 1013

Required information size is a Two-sided graph

Conventional monitoring boundary for benefit


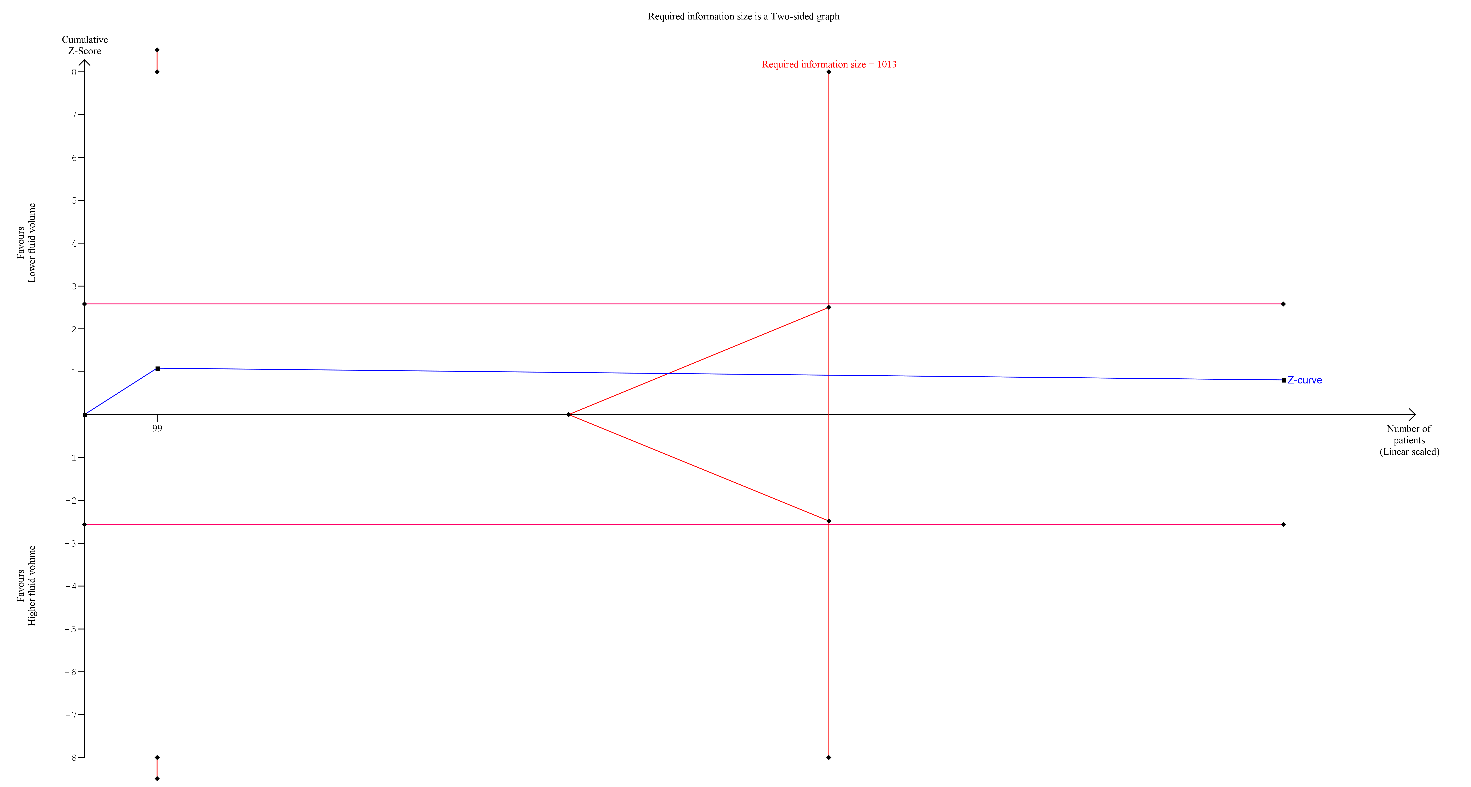


Trial sequential analysis (TSA) of duration of renal replacement therapy in three low risk of bias trials, but one zero-event trial not included in the TSA^14^. We used an alpha of 1.25% (two-sided), beta of 10% (power 90%), heterogeneity adjustment of 0%, and an a priori mean difference (MD) of one day in the analysis. The blue cumulative Z-curve crossed the area of futility and required information size was reached. Thus, the TSA is conclusive and a predefined MD of one day is unlikely.

## 7.7 Renal replacement therapy-free days - meta-analysis and TSA

### 7.7.1 Meta-analysis and forest plot of renal replacement therapy-free days (all trials - all low RoB)


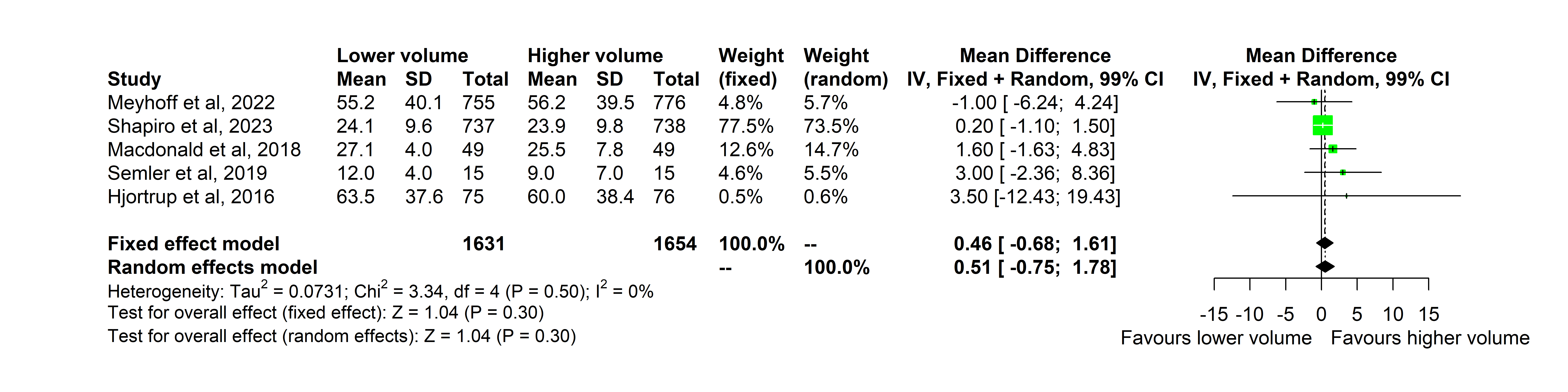


### 7.7.2 TSA of renal replacement therapy-free days (all trials - all low RoB)

Conventional monitoring boundary for harm

Trial sequential monitoring boundary for harm

Trial sequential monitoring boundary for benefit

Area of futility

Favors

Higher fluid volumes

Favors

Lower fluid volumes

3285

No. of patients

Required information size = 9283

Required information size is a Two-sided graph

Conventional monitoring boundary for benefit


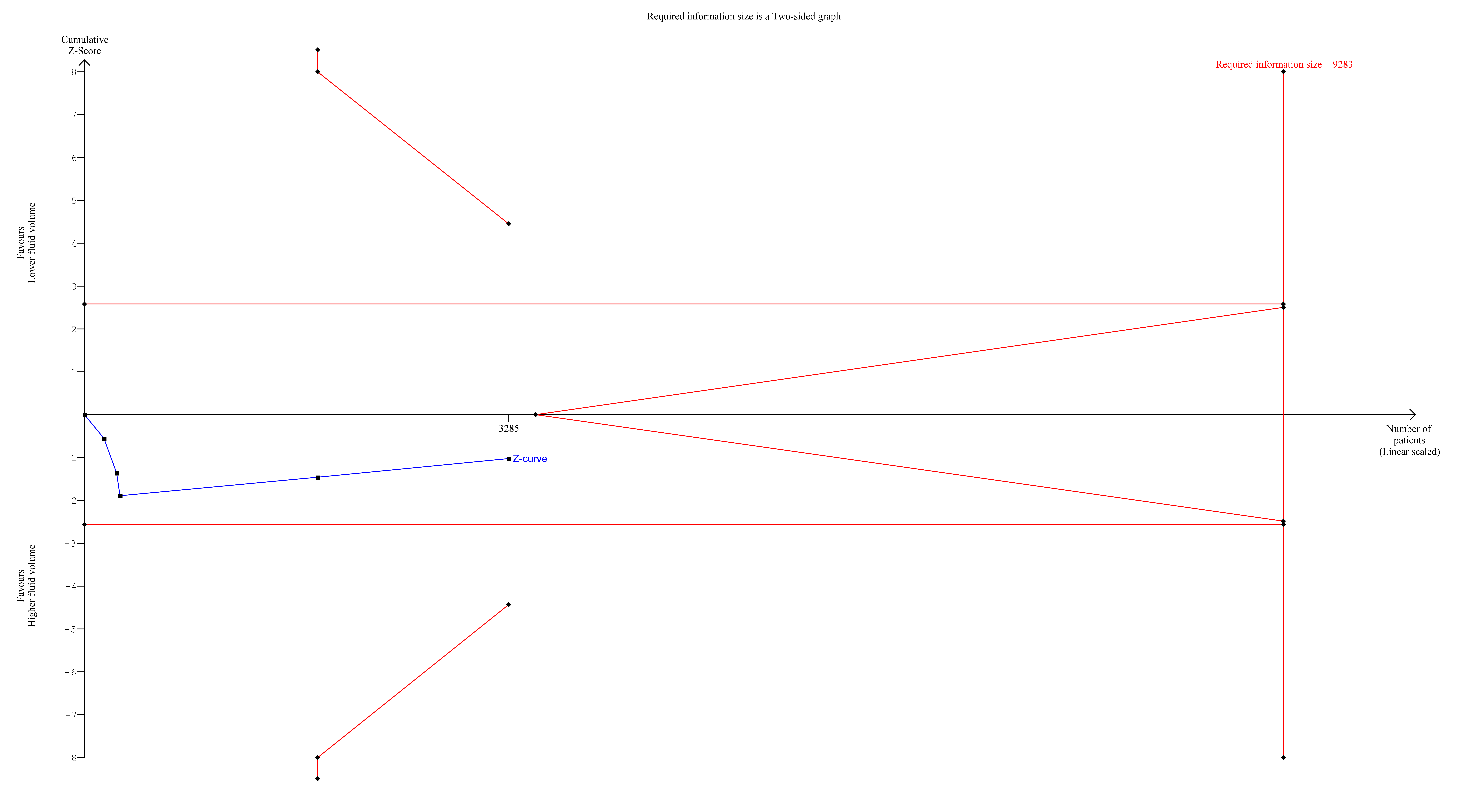


Trial sequential analysis (TSA) of renal replacement therapy free days in four low risk of bias trials. We used an alpha of 1.25% (two-sided), beta of 10% (power 90%), heterogeneity adjustment of 0%, and an a priori mean difference (MD) of one day in the analysis. The TSA adjusted confidence interval in the fixed effect model was -1.50 to 2.43 with a diversity D^2^=0%. The blue cumulative Z-curve did not cross the conventional monitoring boundaries for benefit, harm or futility; thus, the TSA is inconclusive. 35% (3285 patients) of the required information size of 9283 patients was accrued.

## 7.6 Incidence of acute kidney injury - meta-analysis and TSA

### 7.6.1 Meta-analysis and forest plot of incidence of acute kidney injury


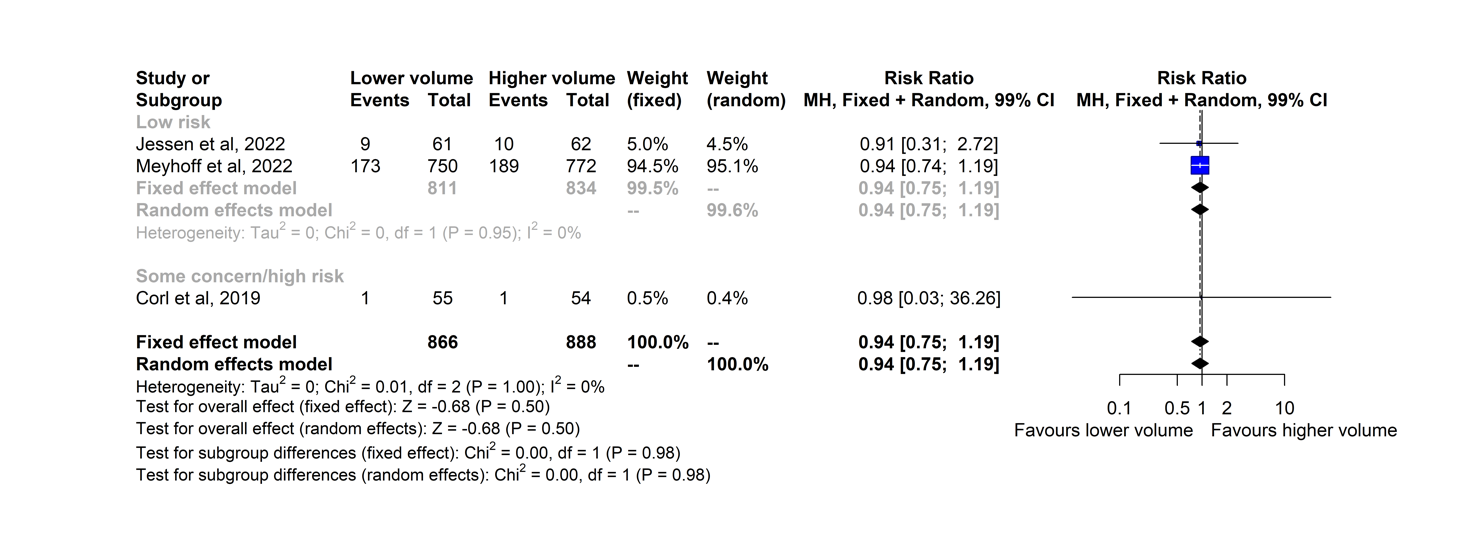


### 7.6.2 TSA of incidence of acute kidney injury (low RoB trials only)

Conventional monitoring boundary for harm

Trial sequential monitoring boundary for harm

Trial sequential monitoring boundary for benefit

Area of futility

Favors

Higher fluid volumes

Favors

Lower fluid volumes

1643

No. of patients

Required information size = 7633

Required information size is a Two-sided graph

Conventional monitoring boundary for benefit


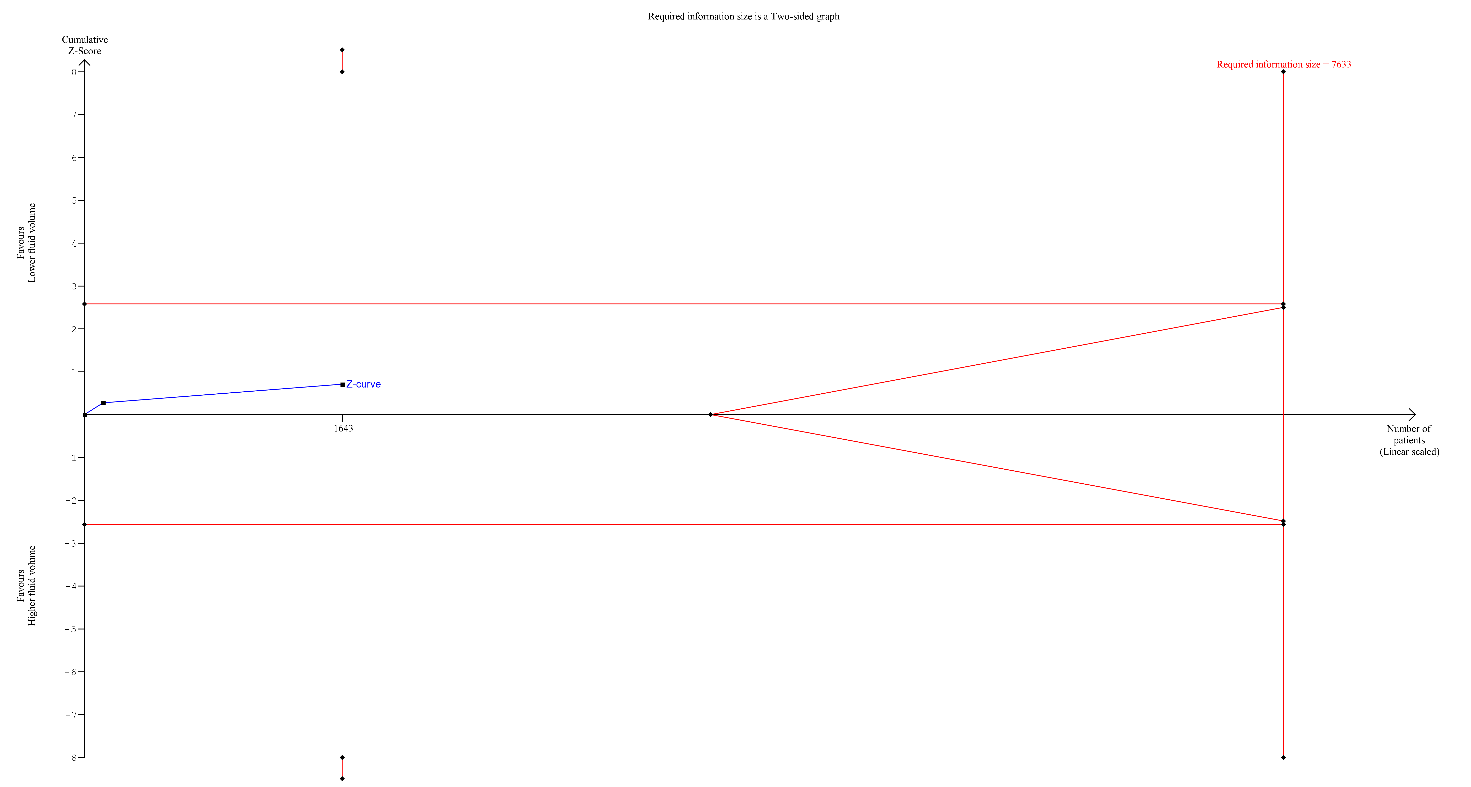


Trial sequential analysis (TSA) of the incidence of acute kidney injury in two trials. We used a control event proportion of 23.9%, alpha 1.25% (two-sided), beta 10% (power 90%), heterogeneity adjustment of 0%, and an a priori relative risk reduction of 15% in the analysis. The TSA adjusted confidence interval in the fixed effect model was 0.46 to 1.93 with a diversity D^2^=0%. The blue cumulative Z-curve did not cross the conventional monitoring boundaries for benefit, harm or futility; thus, the TSA is inconclusive. 22% (1643 patients) of the required information size of 7633 patients was accrued.

### 7.6.3 TSA of incidence of acute kidney injury (all trials)

Conventional monitoring boundary for harm

Trial sequential monitoring boundary for harm

Trial sequential monitoring boundary for benefit

Area of futility

Favors

Higher fluid volumes

Favors

Lower fluid volumes

1851

No. of patients

Required information size = 8242

Required information size is a Two-sided graph

Conventional monitoring boundary for benefit


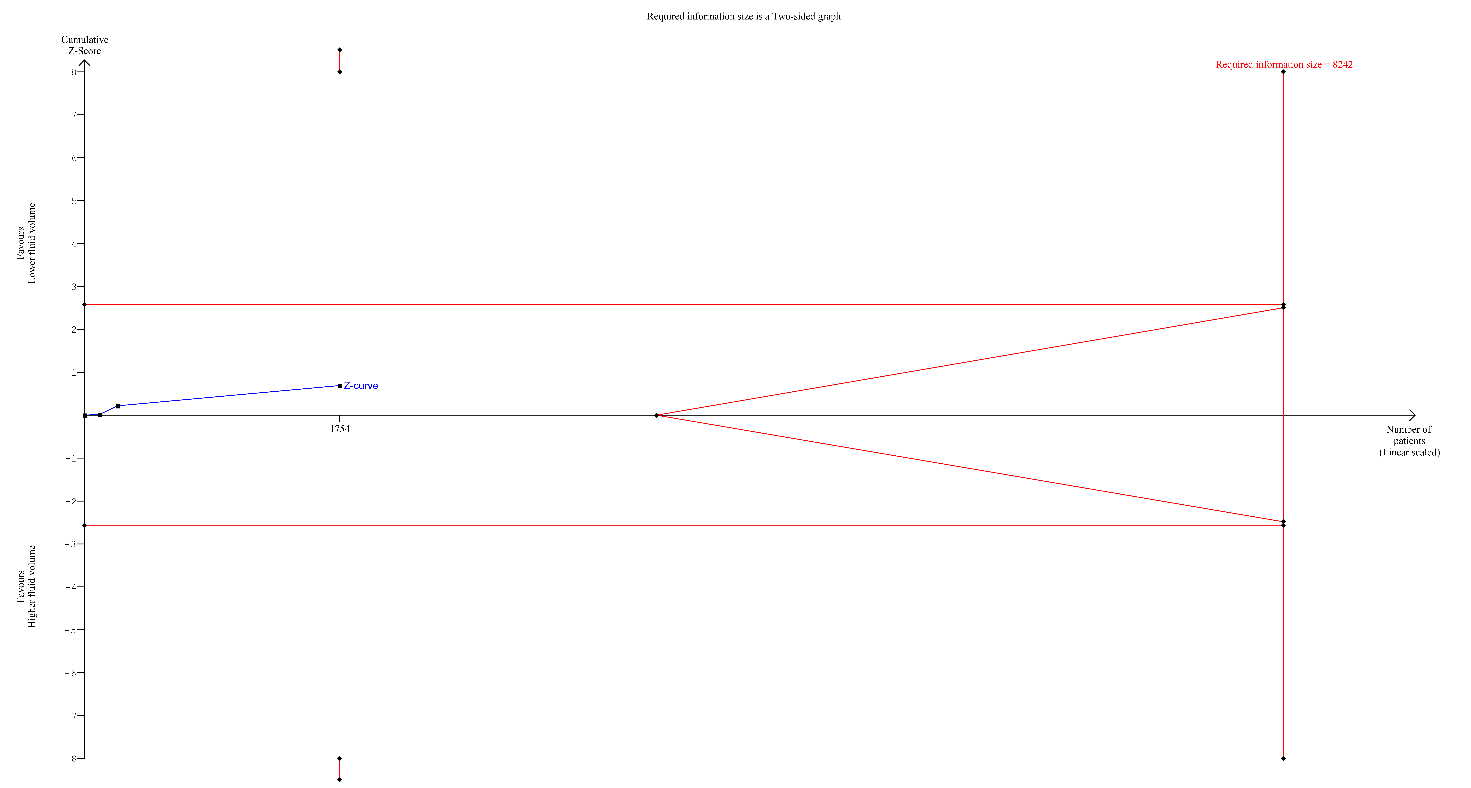


Trial sequential analysis (TSA) of the incidence of acute kidney injury in three trials. We used a control event proportion of 22.5 %, alpha 1.25% (two-sided), beta 10% (power 90%), heterogeneity adjustment of 0%, and an a priori relative risk reduction of 15% in the analysis. The TSA adjusted confidence interval in the fixed effect model was 0.46 to 1.89 with a diversity D^2^=0%. The blue cumulative Z-curve did not cross the conventional monitoring boundaries for benefit, harm or futility; thus, the TSA is inconclusive. 22% (1851 patients) of the required information size of 8482 patients was accrued.

# 8. e-Appendix 8 Analyses of exploratory outcomes

## 8.1 Use of blood products - meta-analysis and TSA

### 8.1.1 Meta-analysis and forest plot of the use of blood products


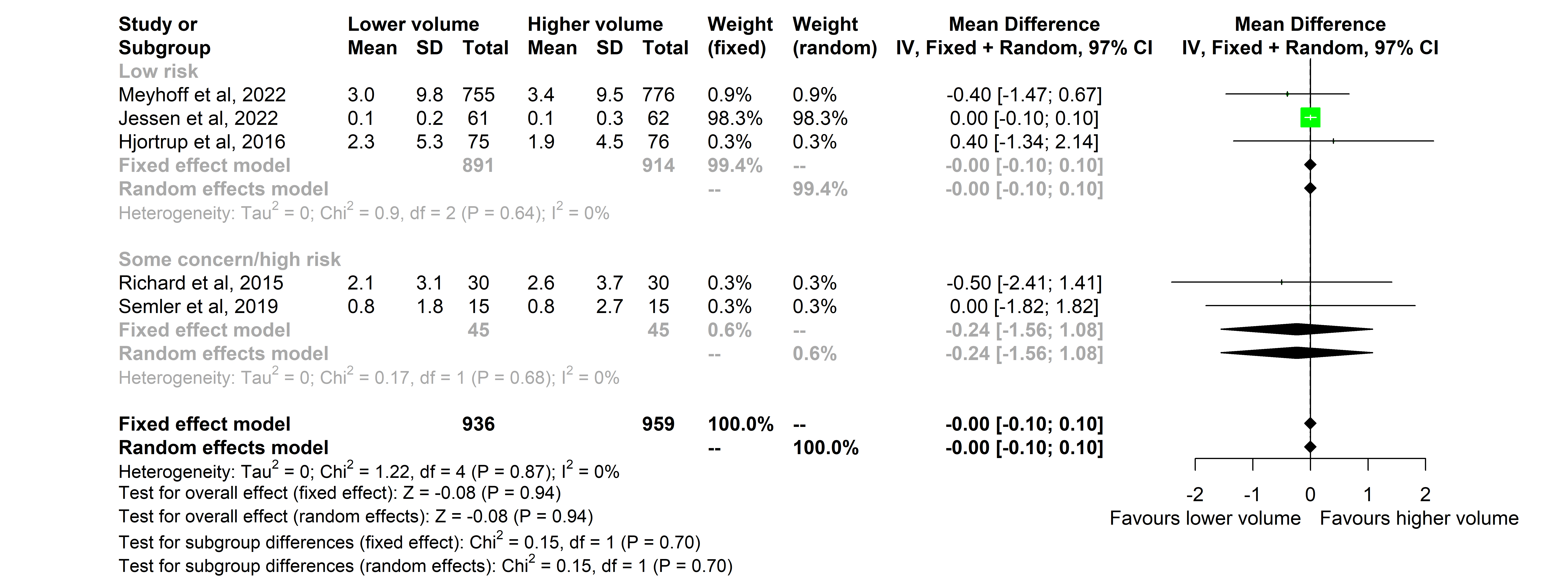


Six trials reported the use of blood products, and five trials allowed for meta-analysis (n=1895)^6,7,11,14–16^. In the meta-analysis of the three low risk of bias trials, we found no statistically significant difference between lower vs higher fluid volumes (fixed effect, MD 0.00 units, 97% CI -0.10 to 0.10). This was consistent with the analysis including all trials.

### 8.1.1 TSA of the use of blood products (low RoB trials only)


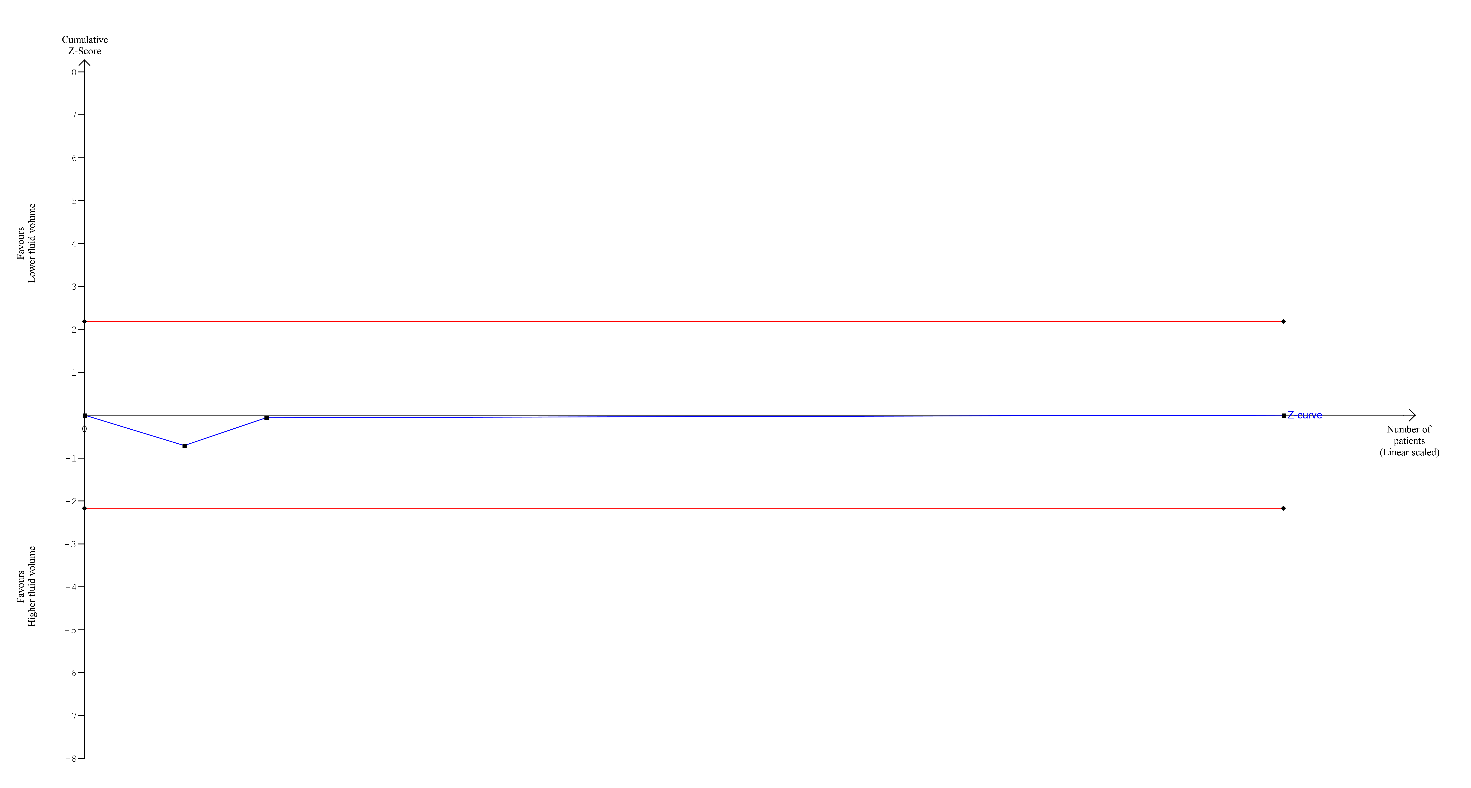


Conventional monitoring boundary for harm

Favors

Higher fluid volumes

Favors

Lower fluid volumes

No. of patients

Required information size is a Two-sided graph

Conventional monitoring boundary for benefit

Trial sequential analysis (TSA) of the use of blood products in three low risk of bias trials. We used an alpha of 2.5% (two-sided), beta of 10% (power 90%), heterogeneity adjustment of 0%, and an a priori mean difference (MD) of one unit in the analysis. First trial exceeds required information size of 47 patients. Thus, the TSA is conclusive and a predefined MD of one unit is unlikely; high certainty of evidence.

### 8.1.2 TSA of the use of blood products (all trials)

Conventional monitoring boundary for harm

Trial sequential monitoring boundary for harm

Trial sequential monitoring boundary for benefit

Favors

Higher fluid volumes

Favors

Lower fluid volumes

No. of patients

Required information size is a Two-sided graph

Conventional monitoring boundary for benefit


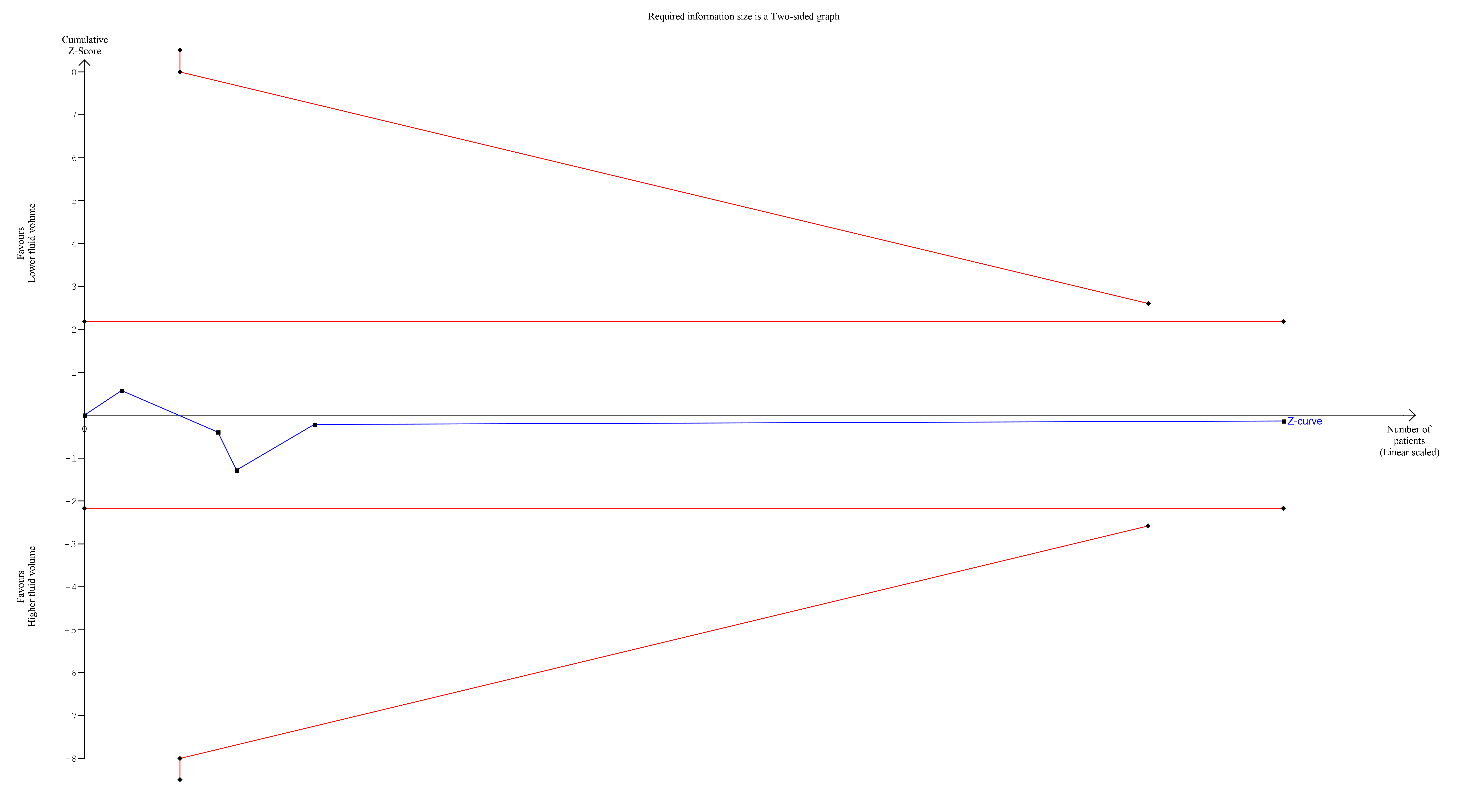


Trial sequential analysis (TSA) of the use of blood products in five trials. We used an alpha of 2.5% (two-sided), beta of 10% (power 90%), heterogeneity adjustment of 0%, and an a priori mean difference (MD) of one unit in the analysis. First trial exceeds required information size of 48 patients. Thus, the TSA is conclusive and a predefined MD of one unit is unlikely.

## 8.2 ICU length of stay - meta-analysis and TSA

### 8.2.1 Meta-analysis and forest plot of ICU length of stay


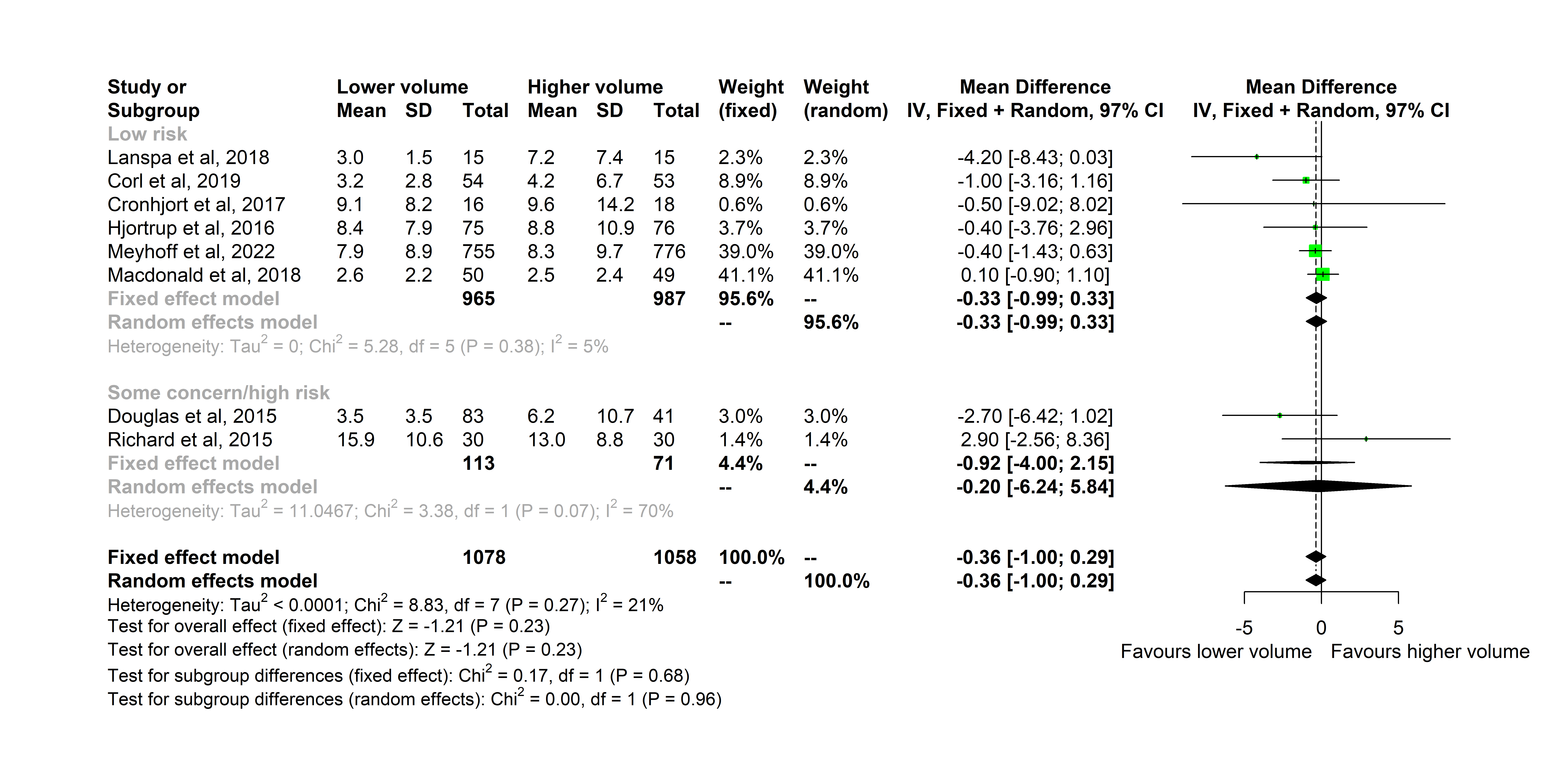


Nine trials reported ICU LOS and eight trials allowed meta-analysis (n=2136) ^5–10,12,13,15^. We found no statistically significant difference between lower vs higher fluid volumes in the meta-analysis of six low risk of bias trials (fixed effect, MD -0.33 days, 97% CI -0.99 to 0.33). This was consistent when including all trials in the analysis.

### 8.2.2 TSA of ICU length of stay (low RoB trials only)

Conventional monitoring boundary for harm

Trial sequential monitoring boundary for harm

Trial sequential monitoring boundary for benefit

Area of futility

Favors

Higher fluid volumes

Favors

Lower fluid volumes

1952

No. of patients

Required information size = 2236

Required information size is a Two-sided graph

Conventional monitoring boundary for benefit


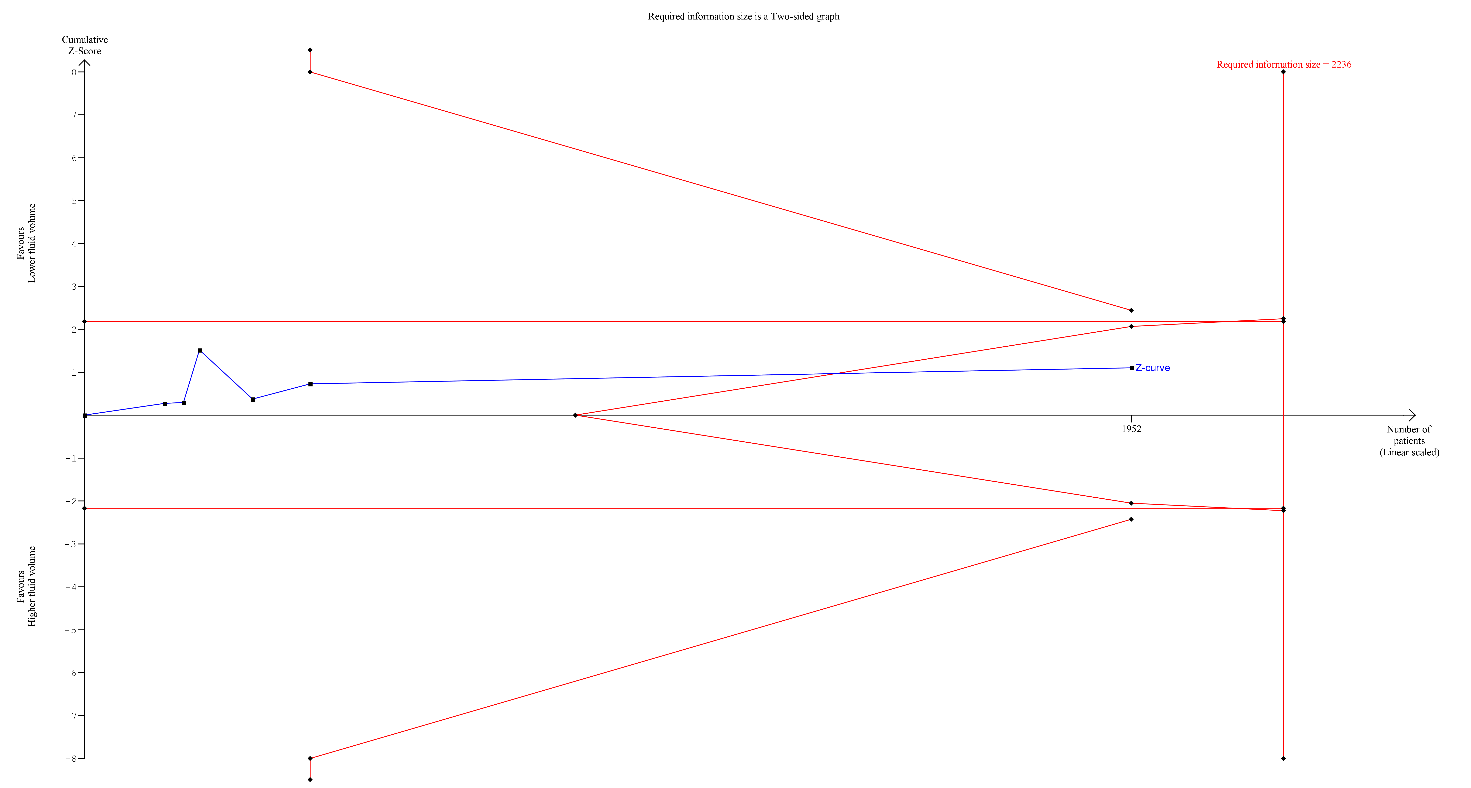


Trial sequential analysis (TSA) of length of stay in the intensive care unit in in six low risk of bias trials. We used an alpha of 2.5% (two-sided), beta of 10% (power 90%), heterogeneity adjustment of 0%, and an a priori mean difference (MD) of one day in the analysis. The TSA adjusted confidence interval in the fixed effect model was -1.07 to 0.40 with a diversity D^2^=14%. The blue cumulative Z-curve crossed the area of futility. Thus, the TSA is conclusive and a predefined mean difference of one day is unlikely. 87% (1952 patients) of the required information size of 2236 patients was accrued, and the certainty of evidence was moderate due to imprecision.

### 8.2.3 TSA of ICU length of stay (all trials)

Conventional monitoring boundary for harm

Trial sequential monitoring boundary for harm

Trial sequential monitoring boundary for benefit

Area of futility

Favors

Higher fluid volumes

Favors

Lower fluid volumes

2121

No. of patients

Required information size = 2332

Required information size is a Two-sided graph

Conventional monitoring boundary for benefit


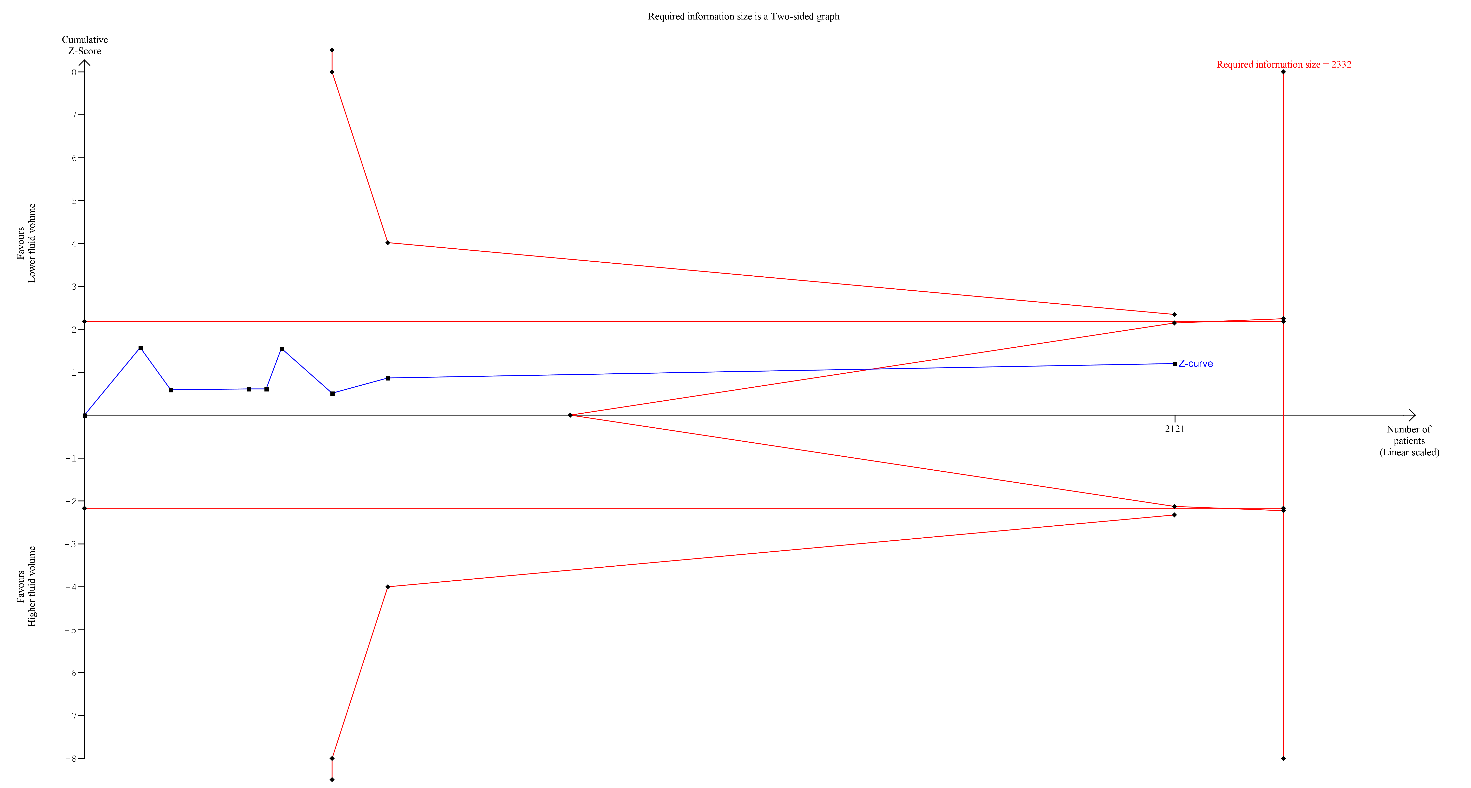


Trial sequential analysis (TSA) of length of stay in the intensive care unit in in eight trials. We used an alpha of 2.5% (two-sided), beta of 10% (power 90%), heterogeneity adjustment of 0%, and an a priori mean difference (MD) of one day in the analysis. The TSA adjusted confidence interval in the fixed effect model was -1.05 to 0.34 with a diversity D^2^=45%. The blue cumulative Z-curve crossed the area of futility. Thus, the TSA is conclusive and a predefined MD of one day is unlikely. 91% (2121 patients) of the required information size of 2332 patients was accrued.

## 8.3 Hospital length of stay - meta-analysis and TSA

### 8.3.1 Meta-analysis and forest plot of hospital length of stay


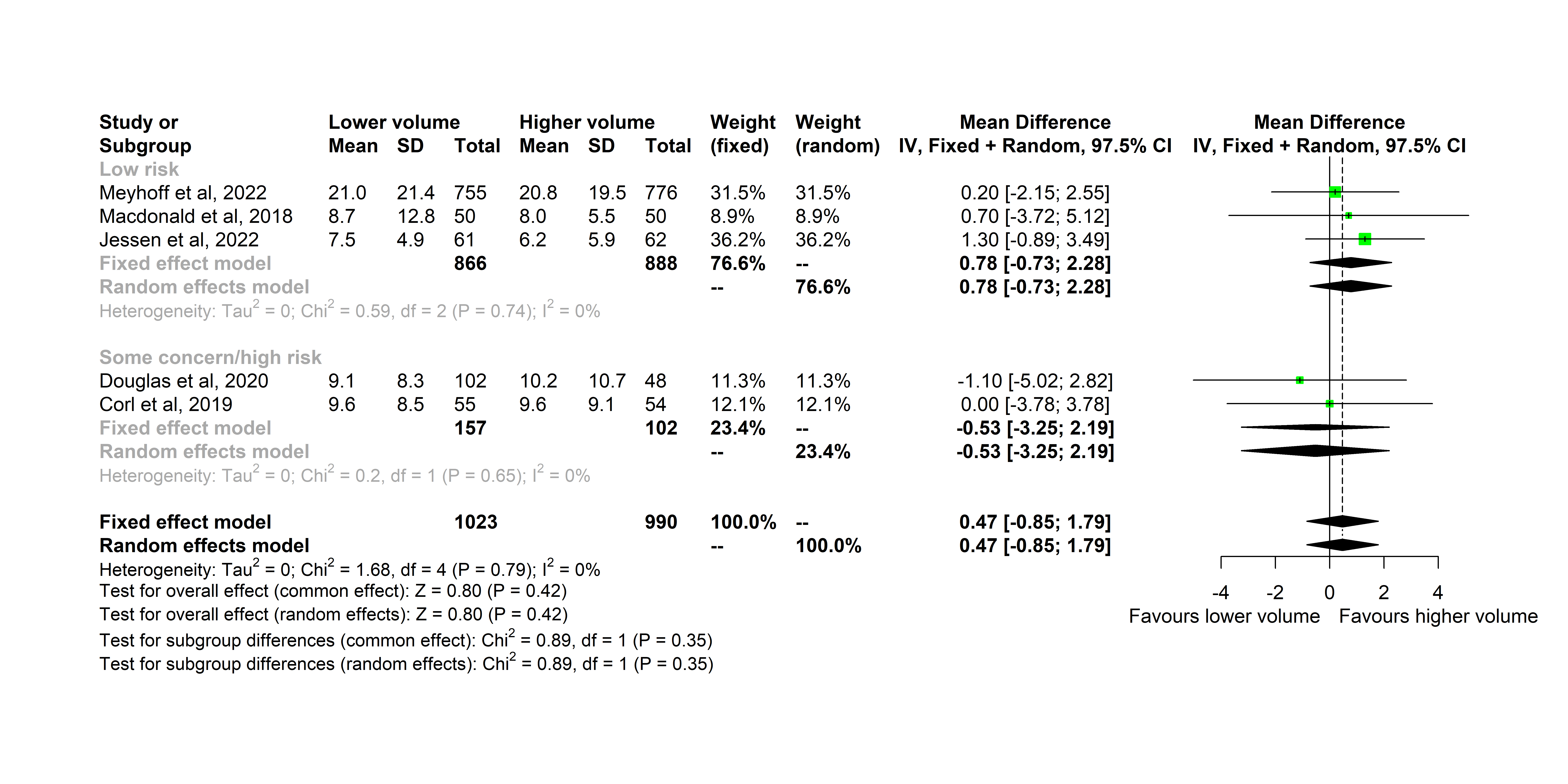


In six trials reporting hospital LOS, five trials allowed meta-analysis (n=2013) ^8,9,12–15^. Meta-analysis of three low risk of bias trials showed no statistically significant difference between lower vs higher fluid volumes (fixed effect MD -0.52 days, 97% CI -0.92 to 1.95). This was consistent with the analysis of all trials.

### 8.3.2 TSA of hospital length of stay (low RoB trials only)

Conventional monitoring boundary for harm

Trial sequential monitoring boundary for harm

Trial sequential monitoring boundary for benefit

Area of futility

Favors

Higher fluid volumes

Favors

Lower fluid volumes

1754

No. of patients

Required information size = 9822

Required information size is a Two-sided graph

Conventional monitoring boundary for benefit


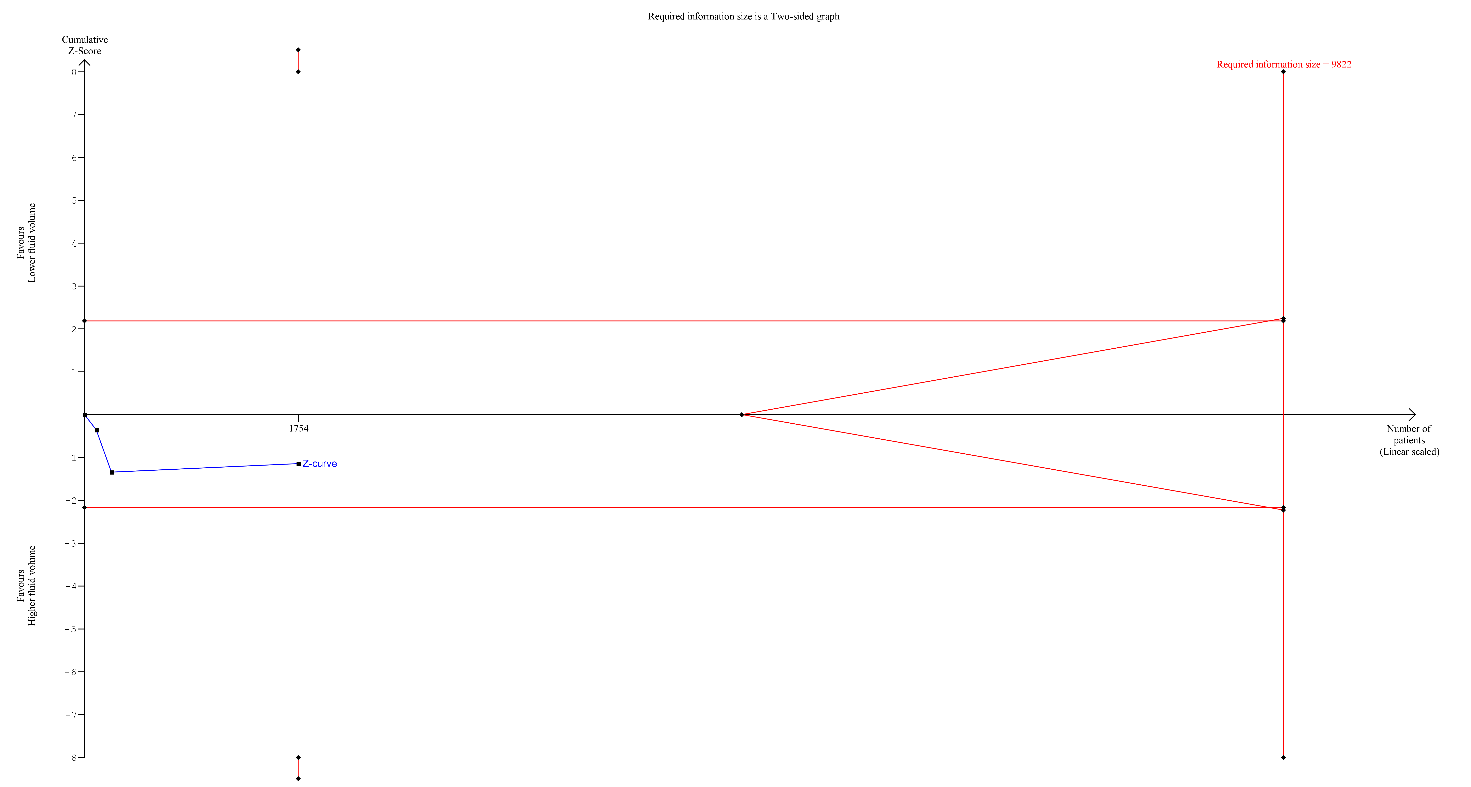


Trial sequential analysis (TSA) of hospital length of stay in three low risk of bias trials. We used an alpha of 2.5% (two-sided), beta of 10% (power 90%), heterogeneity adjustment of 0%, and an a priori mean difference (MD) of one day in the analysis. The TSA adjusted confidence interval in the fixed effect model was -4.60 to 6.15 with a diversity D^2^=0%. The blue cumulative Z-curve did not cross the conventional monitoring boundaries for benefit, harm or futility; thus, the TSA is inconclusive. 18% (1754 patients) of the required information size of 9822 patients was accrued. The certainty of evidence was moderate due to imprecision.

### 8.3.3 TSA of hospital length of stay (all trials)

Conventional monitoring boundary for harm

Trial sequential monitoring boundary for harm

Trial sequential monitoring boundary for benefit

Area of futility

Favors

Higher fluid volumes

Favors

Lower fluid volumes

2013

No. of patients

Required information size = 8632

Required information size is a Two-sided graph

Conventional monitoring boundary for benefit


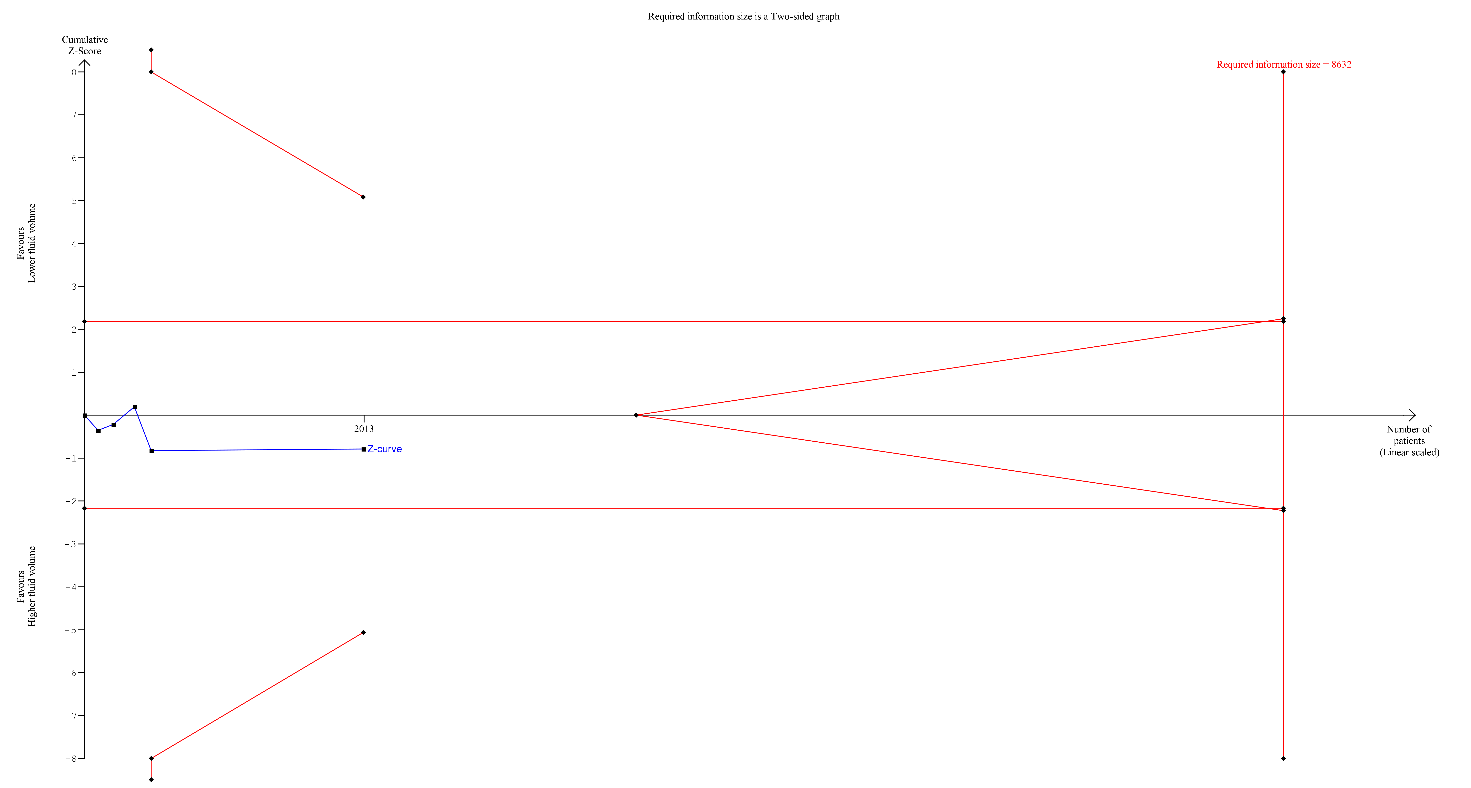


Trial sequential analysis (TSA) of hospital length of stay in three low risk of bias trials. We used an alpha of 2.5% (two-sided), beta of 10% (power 90%), heterogeneity adjustment of 0%, and an a priori mean difference (MD) of one day in the analysis. The TSA adjusted confidence interval in the fixed effect model was -2.52 to 3.46 with a diversity D^2^=0%. The blue cumulative Z-curve did not cross the conventional monitoring boundaries for benefit, harm or futility; thus, the TSA is inconclusive. 23% (2013 patients) of the required information size of 8632 patients was accrued.

# 9. e-Appendix 9 Subgroup analyses

## 9.1 Pre-planned subgroups and hypothesised direction of effect^2^

1. Trials in which the protocol significantly separated administered fluid volumes vs trials in which the protocol did not.

Hypothesised direction of subgroup effect: increased beneficial intervention effect in the trials with separation of fluid volumes.

1. Trials with patients with sepsis vs. trials with patients with septic shock.

Hypothesised direction of subgroup effect: increased beneficial intervention in patients with septic shock.

1. Trials with fluid resuscitation as the only intervention vs trials with fluids as a part of a complex intervention.

Hypothesised direction of subgroup effect: increased beneficial intervention effect in trials with fluid resuscitation as the only intervention.

1. Trials with earlier (≤ 6 h) start of protocolised resuscitation vs trials with later (> 6 h) start.

Hypothesised direction of subgroup effect: increased intervention effect in trials with earlier start.

1. Trials with overall low risk of bias vs some concerns and high risk of bias

Hypothesised direction of subgroup effect: increased beneficial intervention effect in the trials with overall high risk of bias.

1. Comparing estimates of the pooled intervention effect in medical vs surgical patients.

Hypothesised direction of subgroup effect: increased beneficial intervention effect in medical patients.

## 9.2. Forest plots for all subgroup analyses

### 9.2.1 Primary outcomes

### 9.2.1 a) Subgroup analyses of all-cause mortality

### All-cause mortality: Overall low vs some concern or high risk of bias


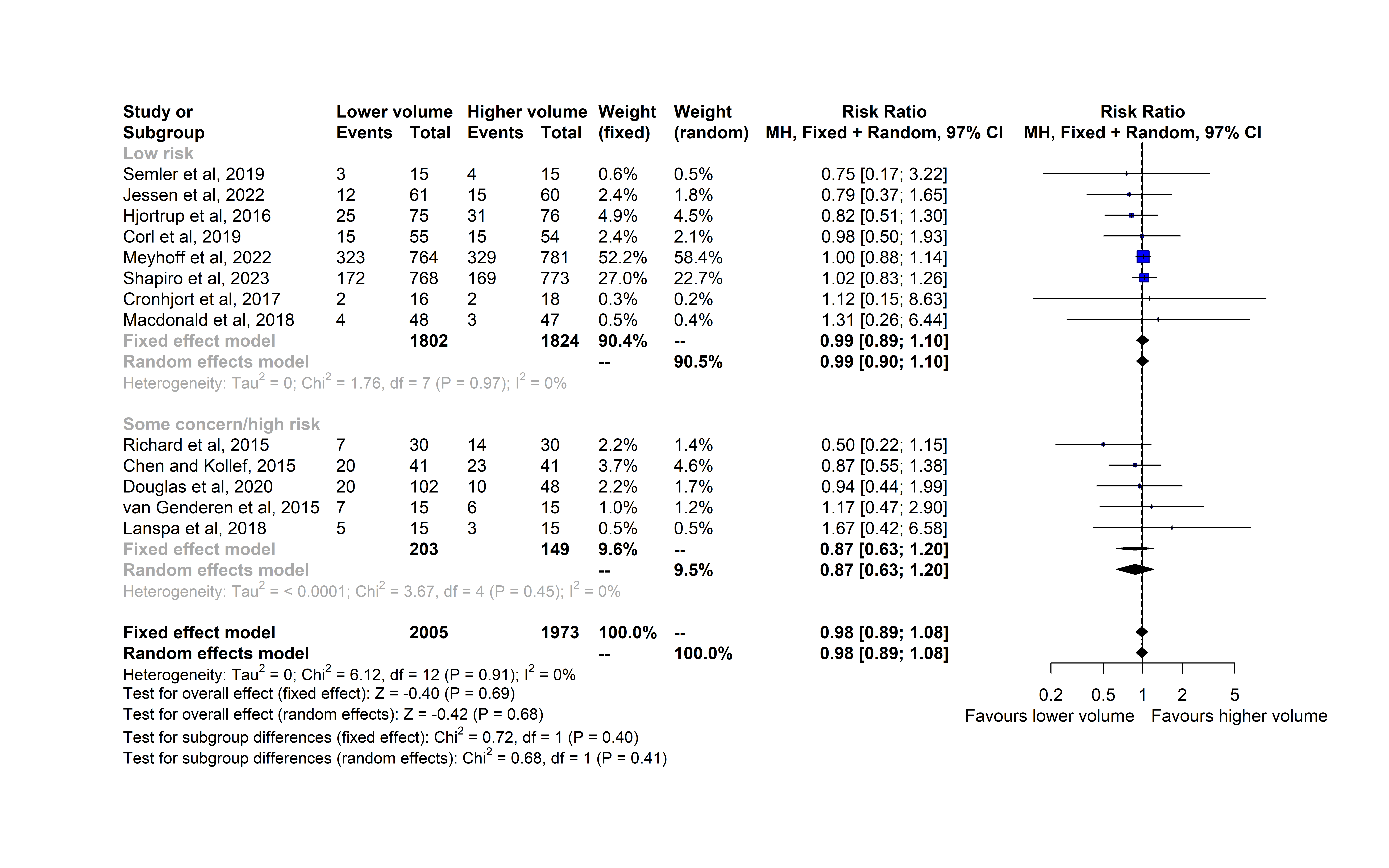


### All-cause mortality: Successful vs unsuccessful separation in fluid volumes


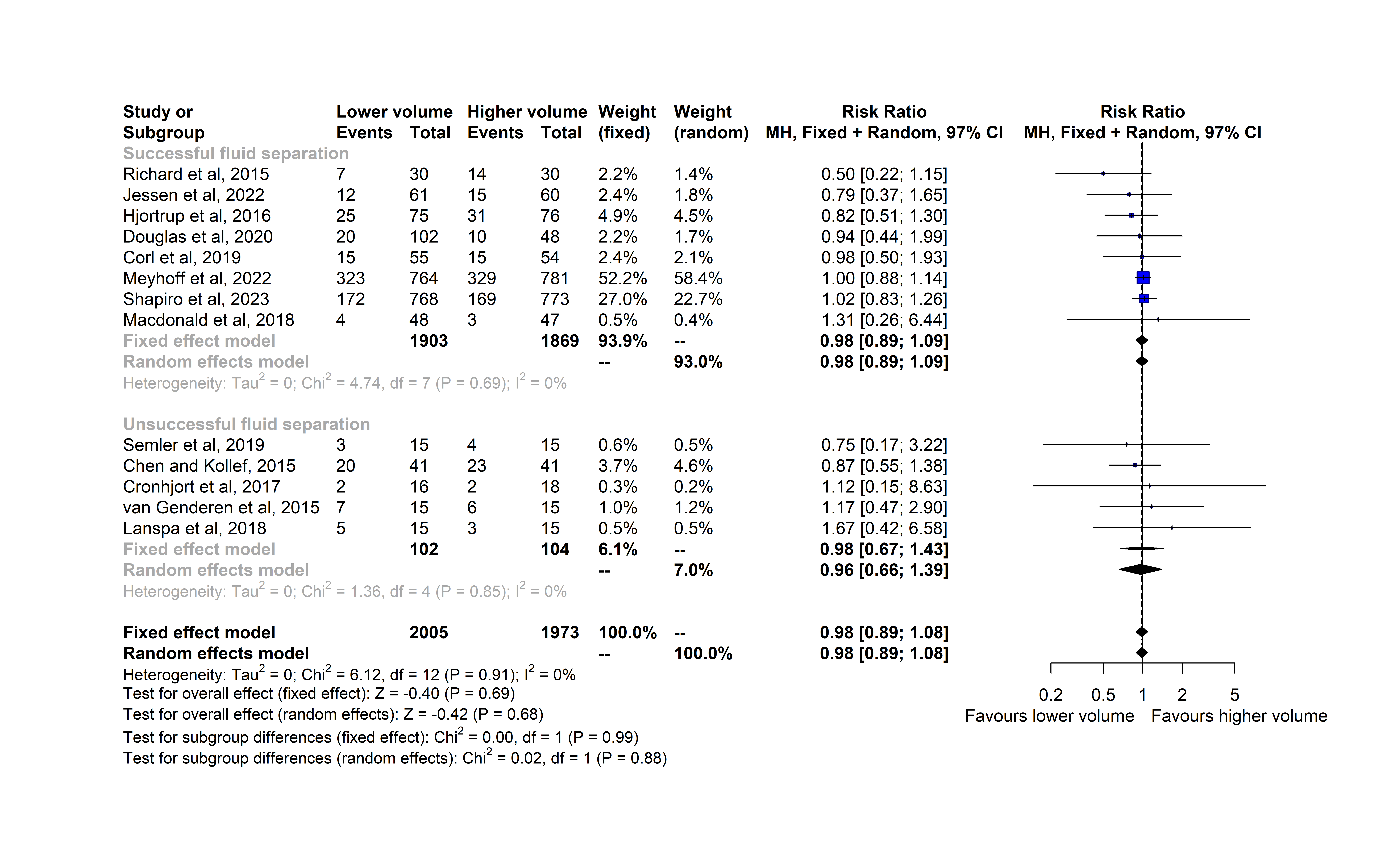


### All-cause mortality: Patients with sepsis vs septic shock


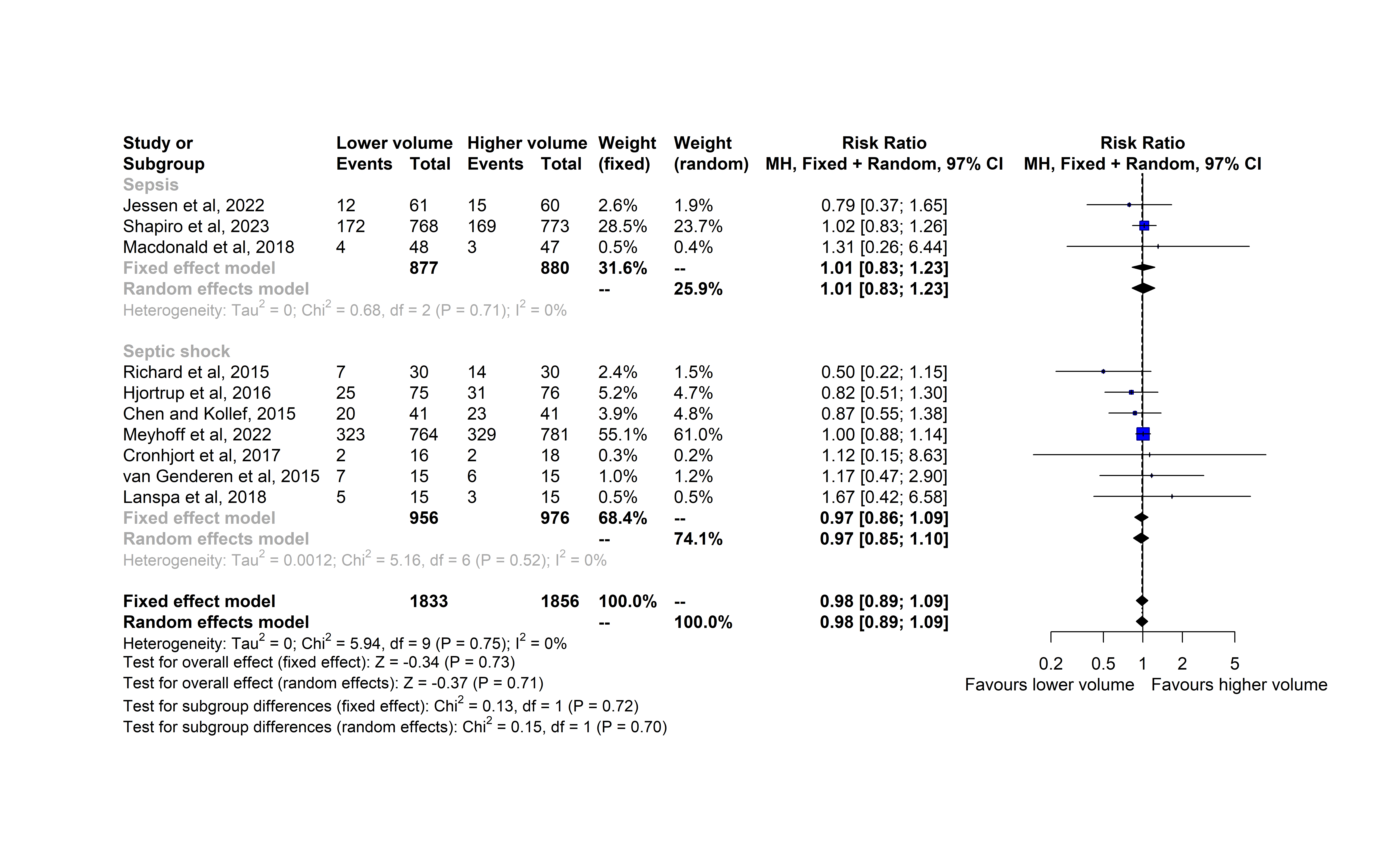


### All-cause mortality: Fluid-only interventions vs a complex hemodynamic protocol


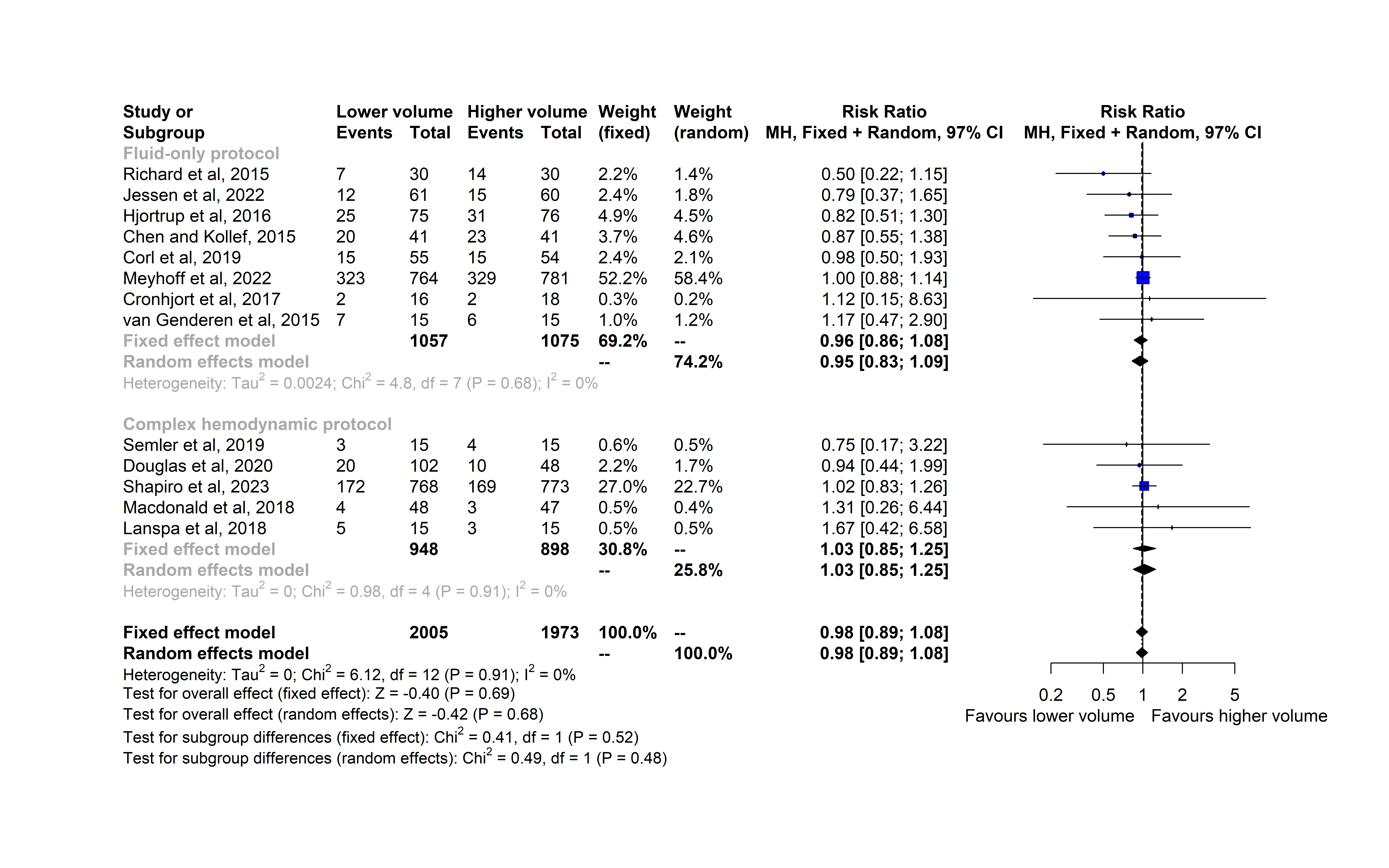


### All-cause mortality: Sepsis-3 definition vs other sepsis definitions (post-hoc subgroup analysis)


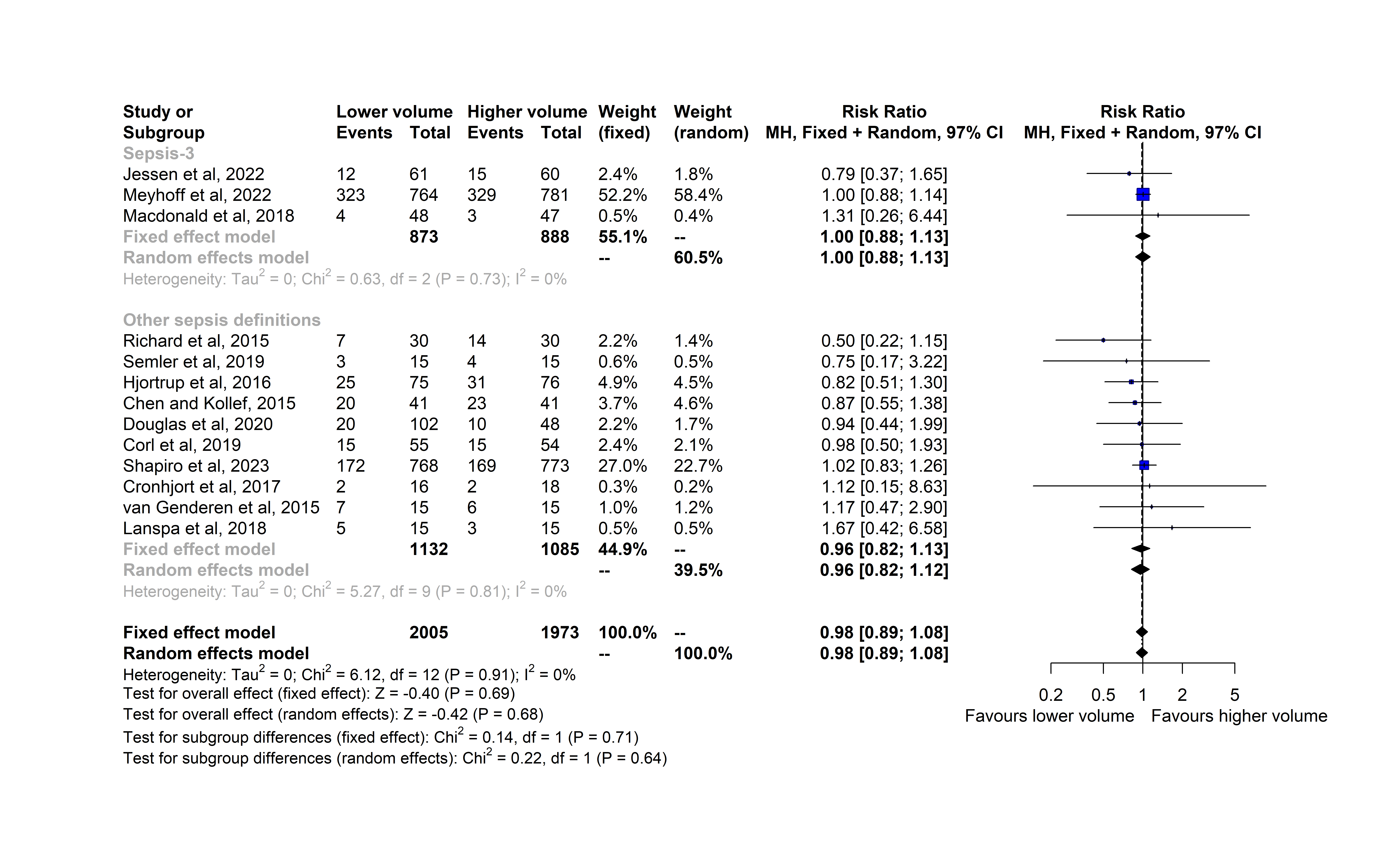


### 9.2.1 b) Subgroup analyses of serious adverse events

### Serious adverse events: Patients with sepsis vs septic shock


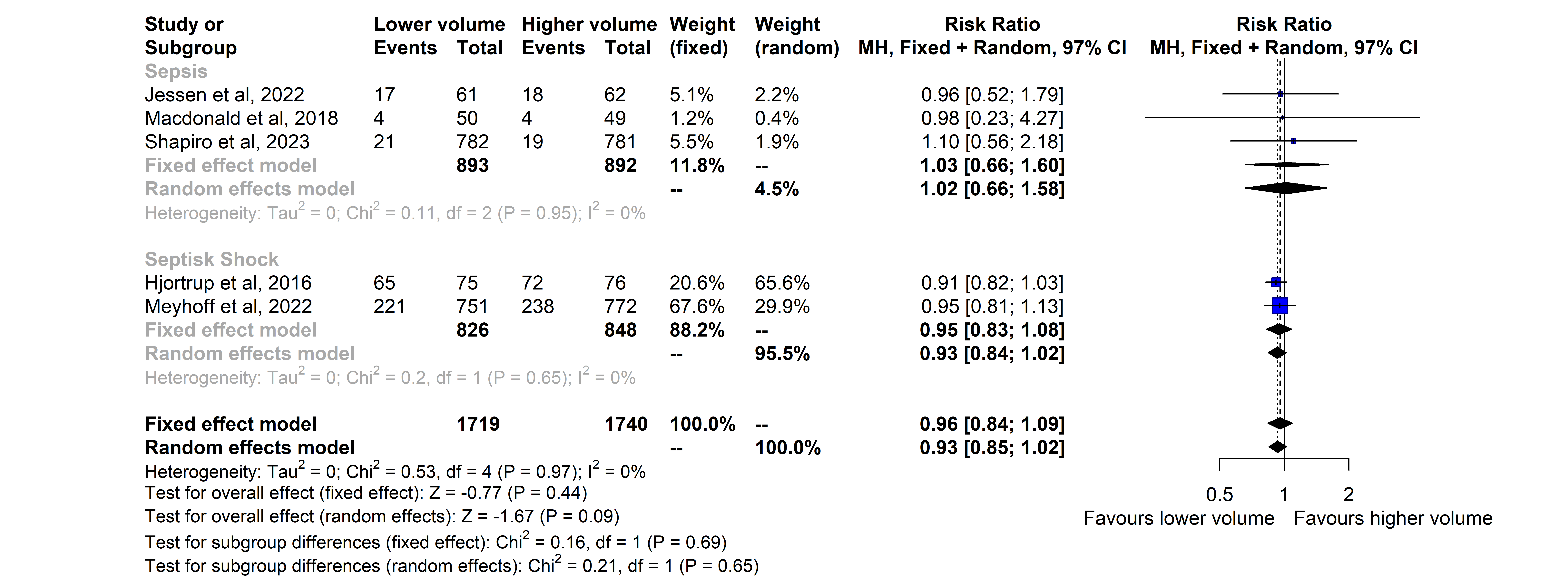


### All-cause mortality: Early vs later resuscitation phase of sepsis


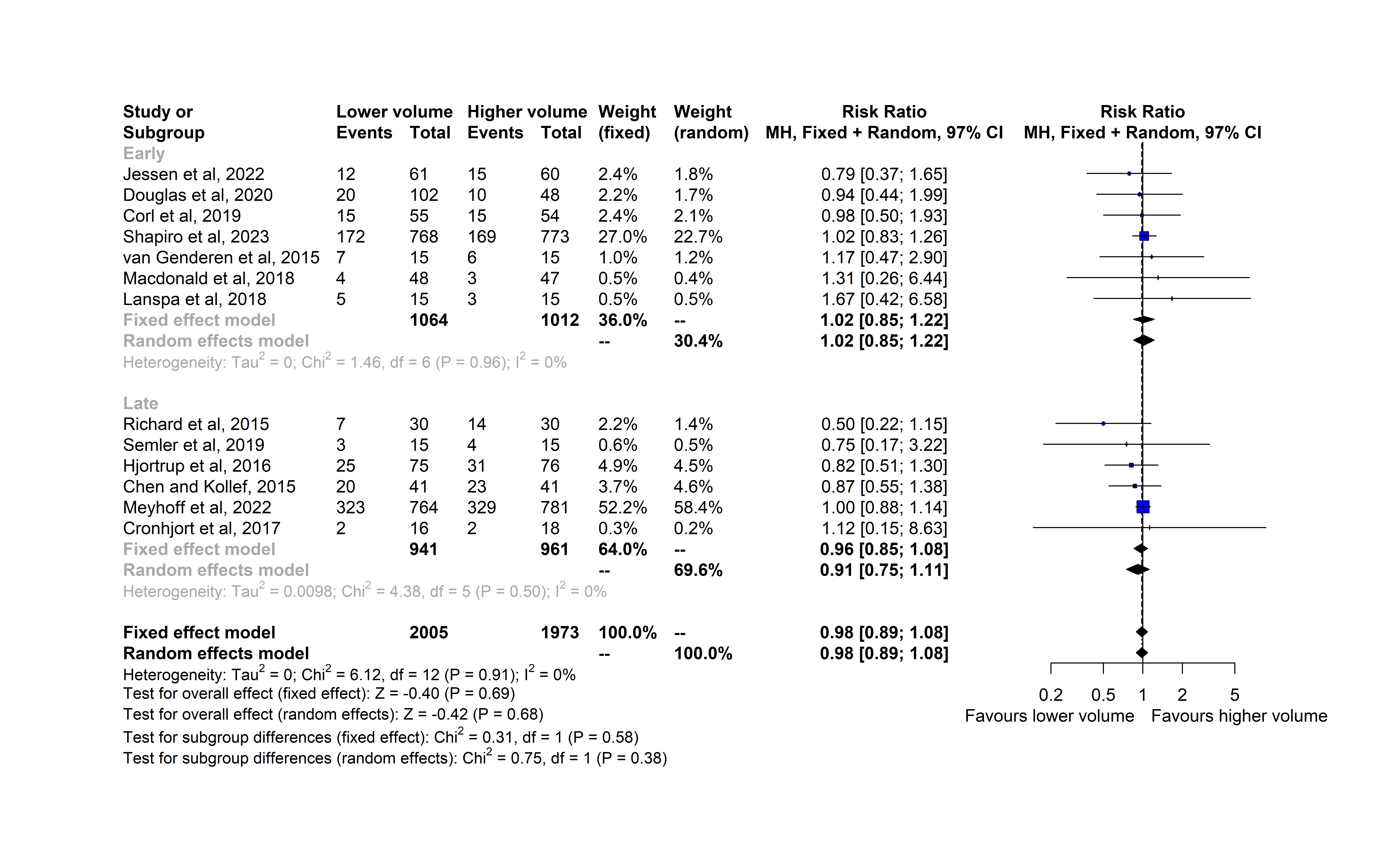


### Serious adverse events: Fluid-only interventions vs a complex hemodynamic protocol


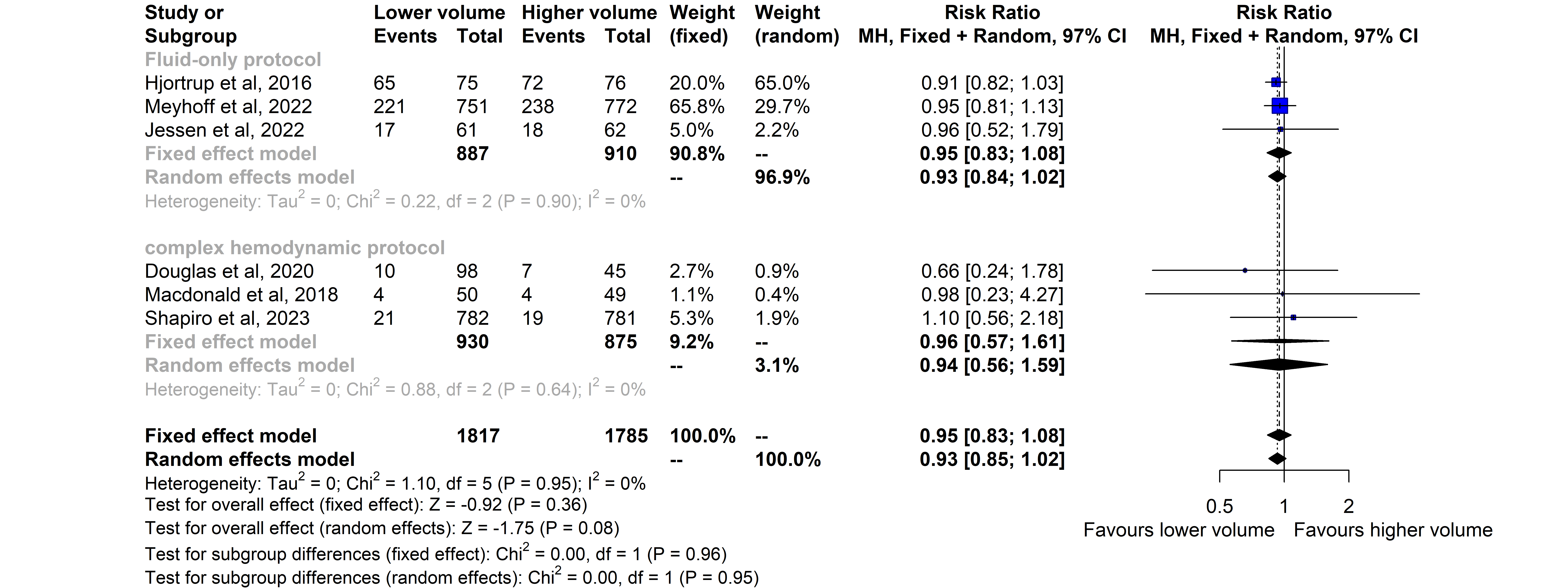


### Serious adverse events: Early vs later resuscitation phase of sepsis


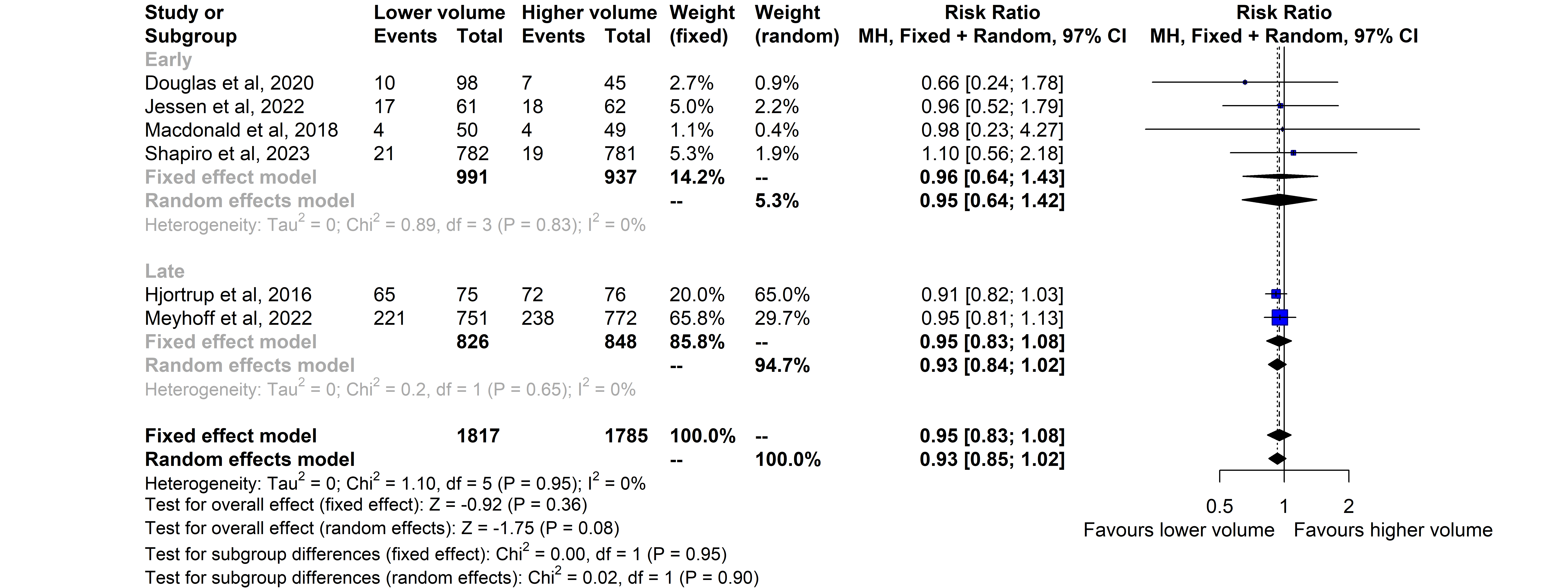


### 9.2.2 Secondary outcomes

### 9.2.2 a) Subgroup analyses of duration of mechanical ventilation

### Duration of mechanical ventilation: Overall low vs some concern or high risk of bias


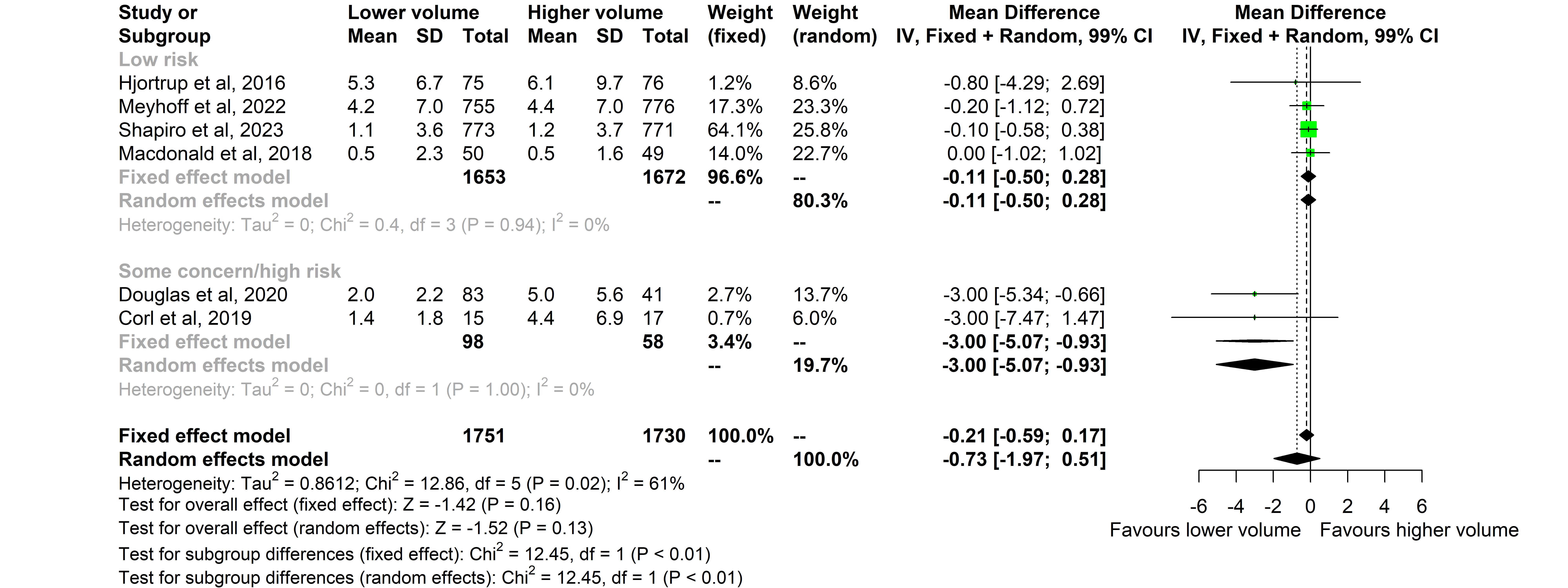


### Duration of mechanical ventilation: Patients with sepsis vs septic shock


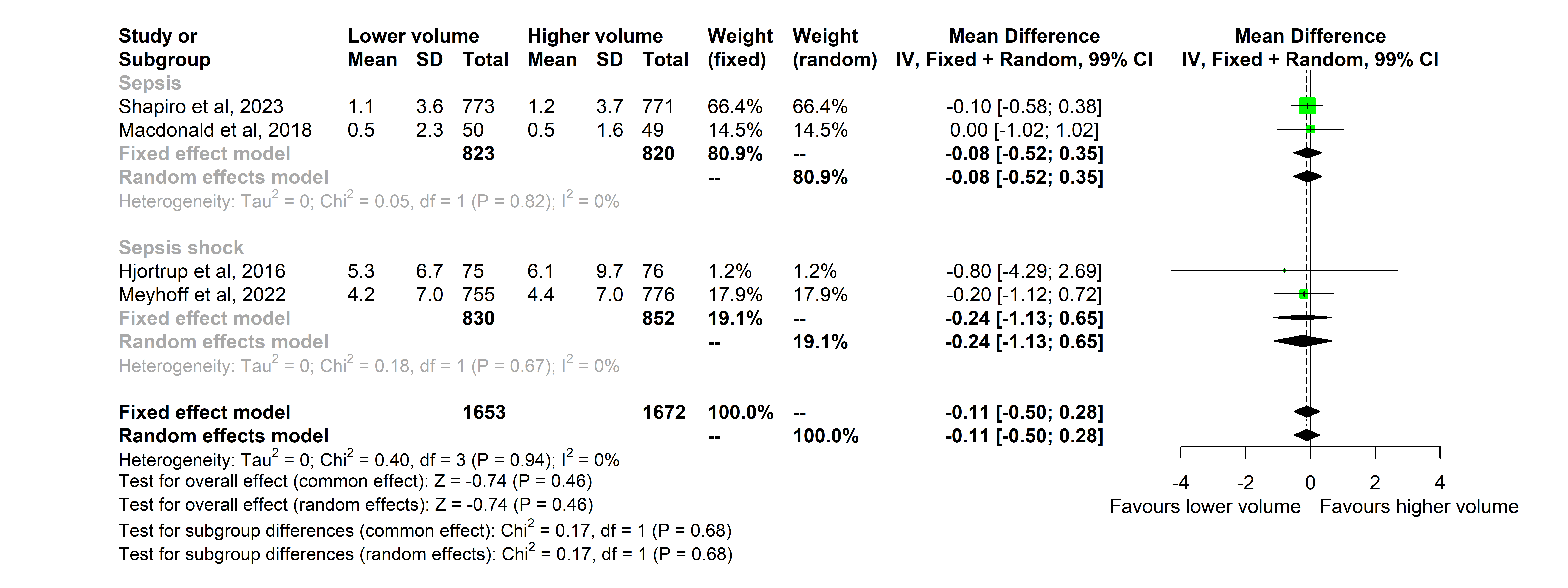


### Duration of mechanical ventilation: Fluid-only interventions vs a complex hemodynamic protocol


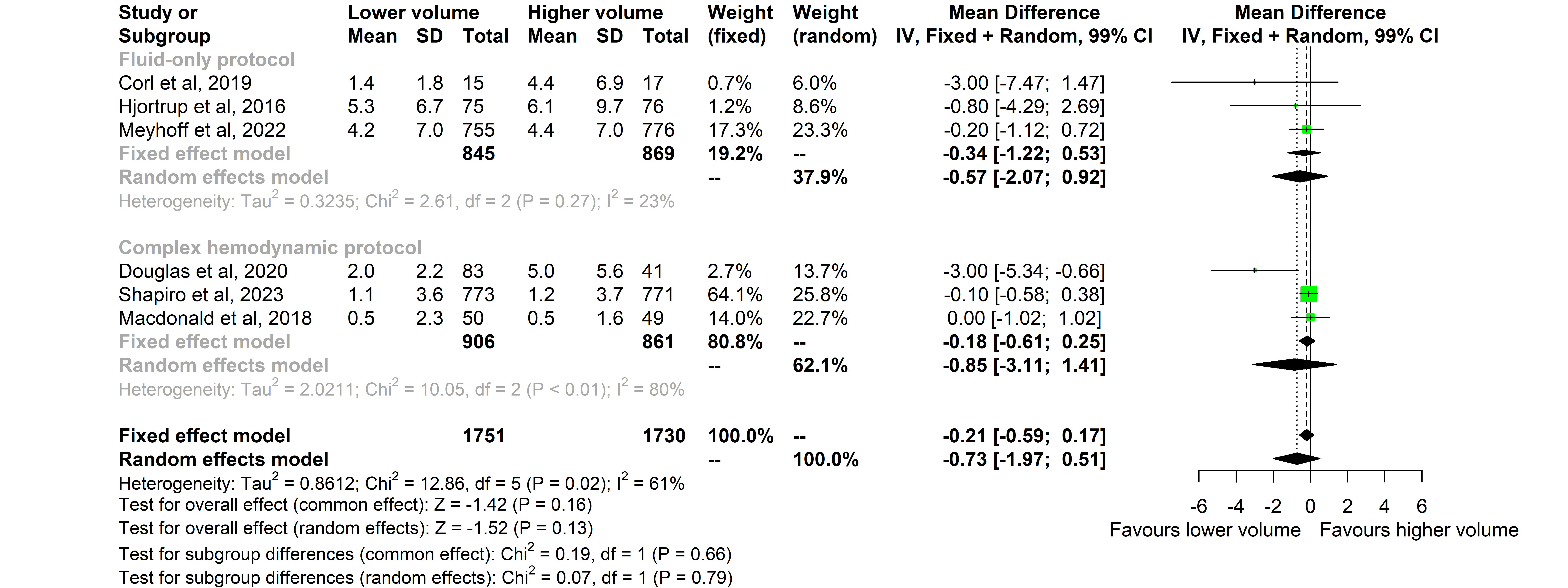


### Duration of mechanical ventilation: Early vs later resuscitation phase of sepsis


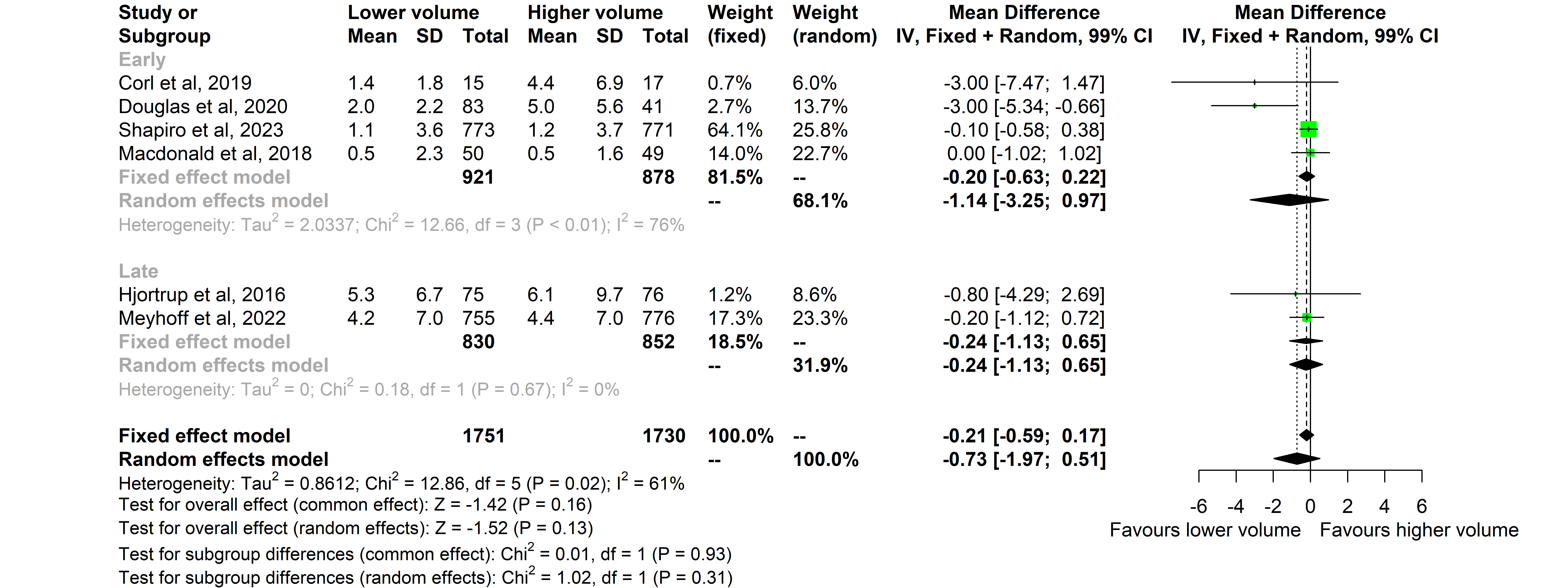


### 9.2.2 b) Subgroup analyses of ventilator-free days

### Ventilator-free days: Overall low vs some concern or high risk of bias


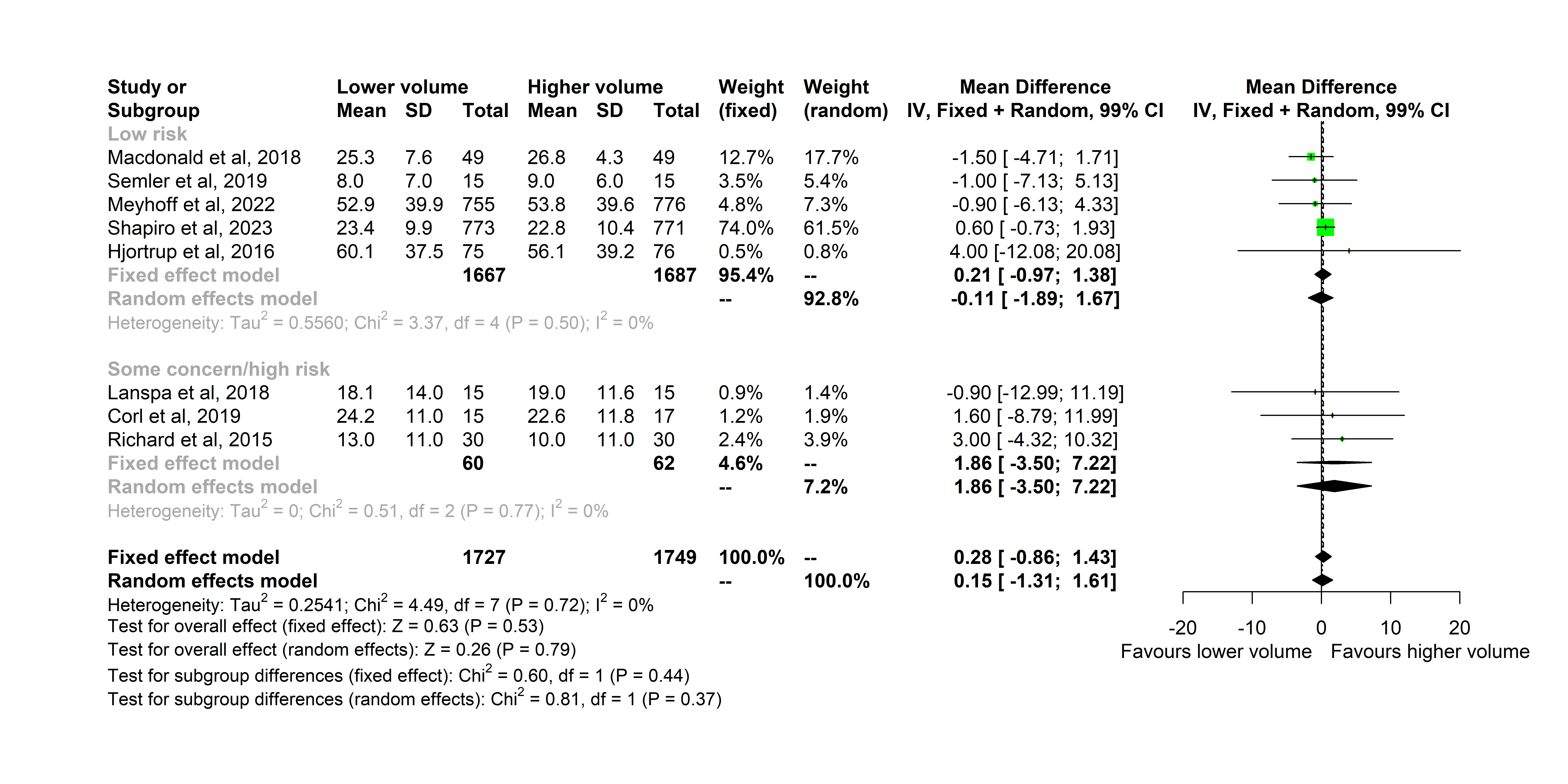


### Ventilator-free days: Successful vs unsuccessful separation in fluid volumes


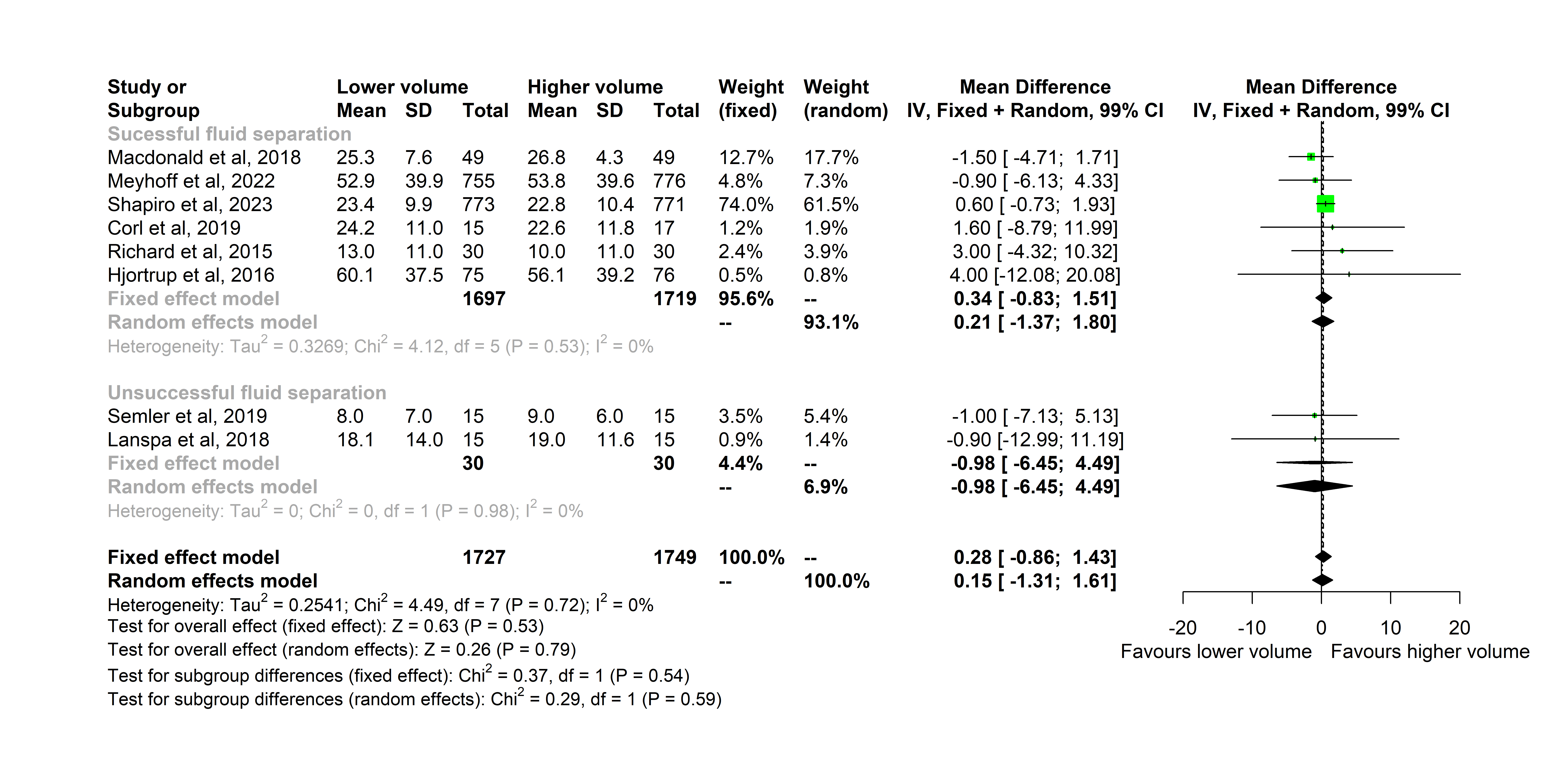


### Ventilator-free days: Patients with sepsis vs septic shock


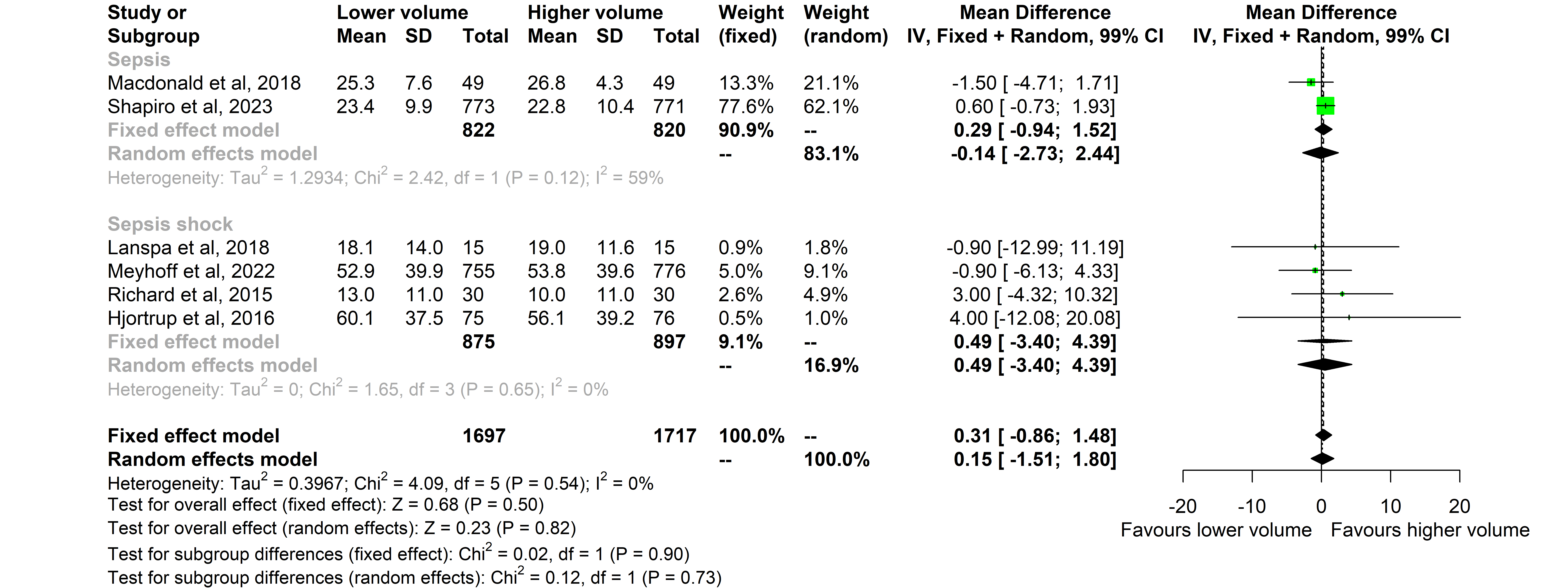


### Ventilator-free days: Fluid-only interventions vs a complex hemodynamic protocol


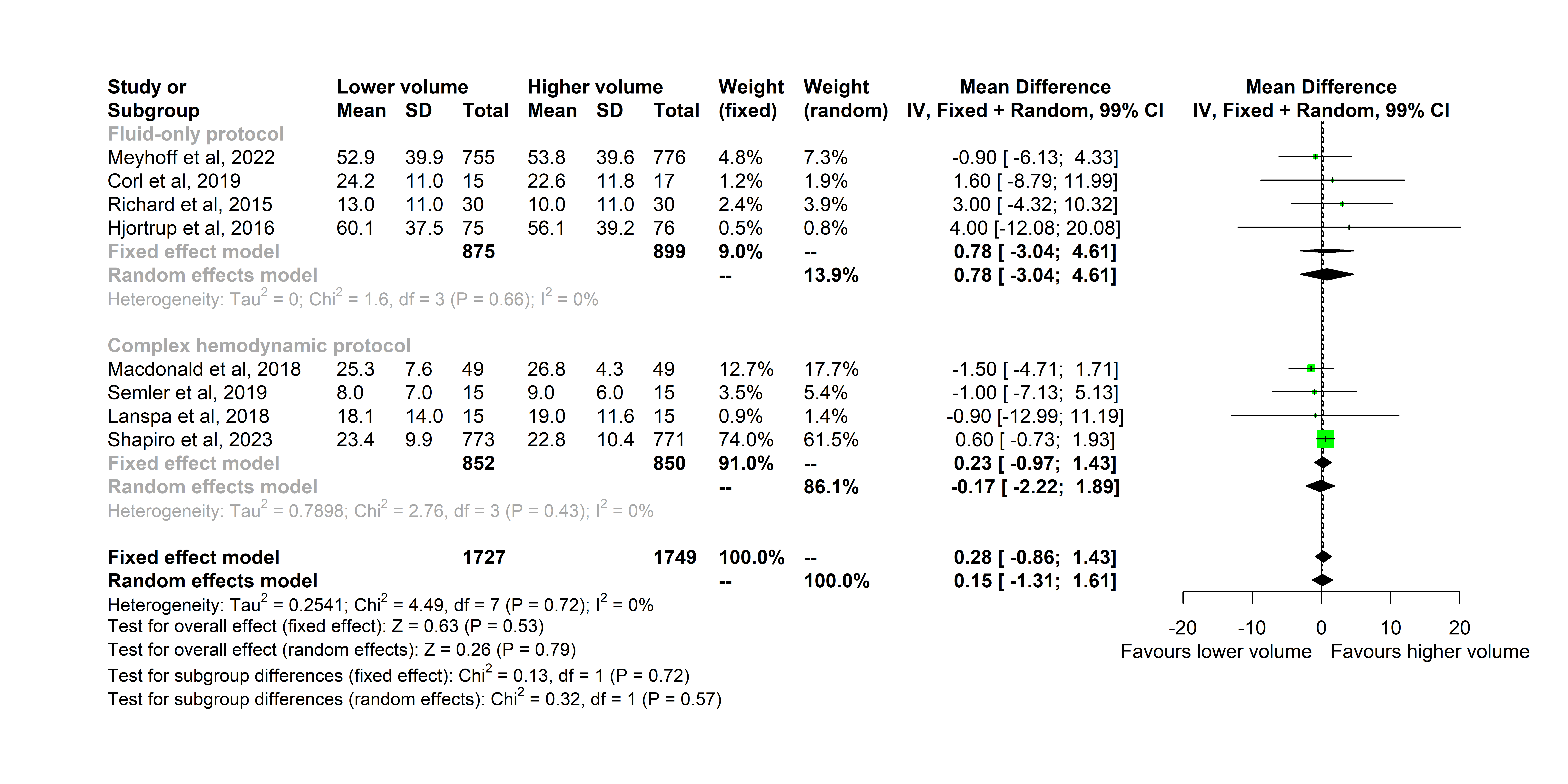


### Ventilator-free days: Early vs later resuscitation phase of sepsis


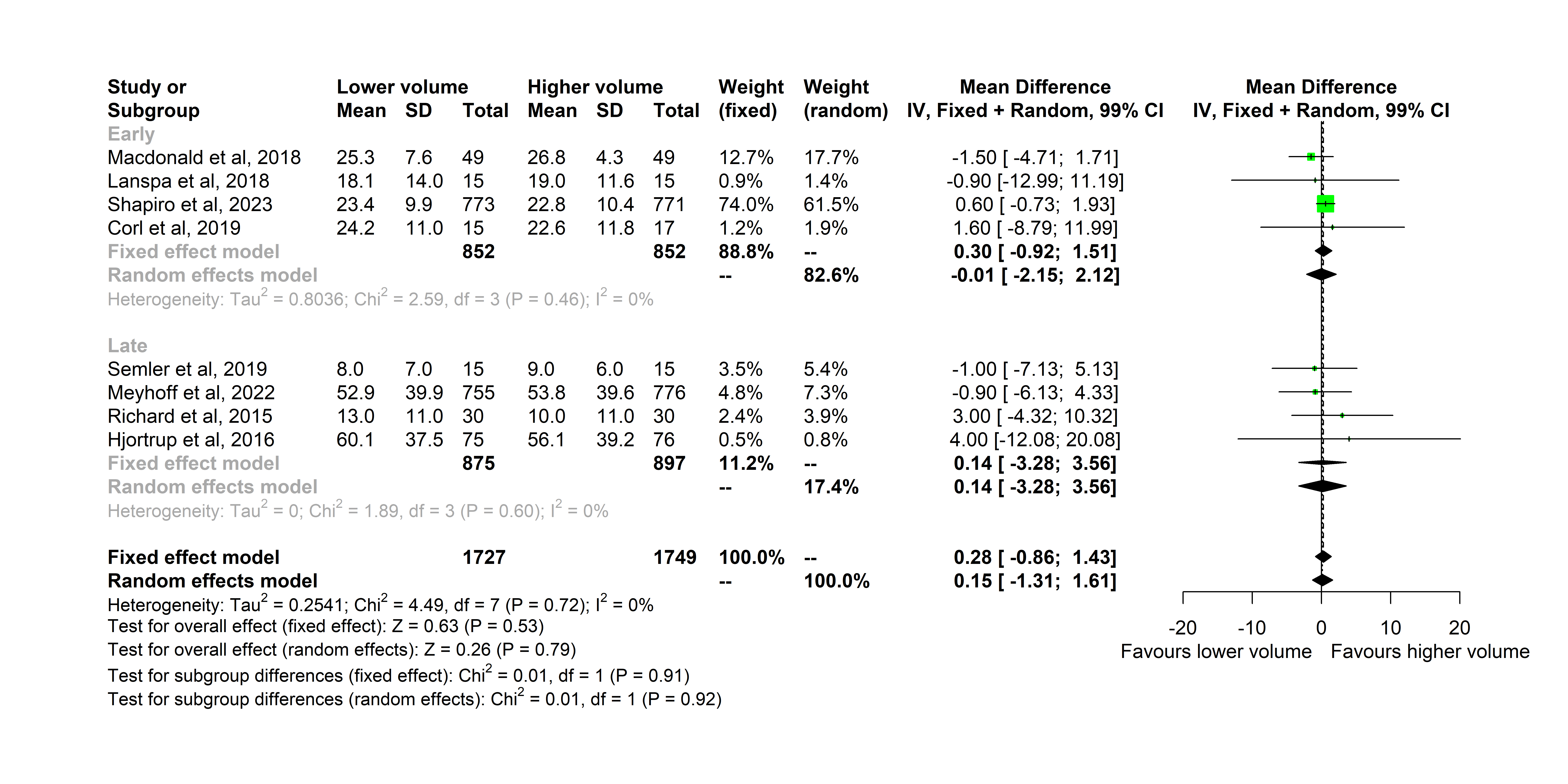


### 9.2.2 c) Subgroup analyses of duration of vasopressor or inotropes

### Duration of vasopressor or inotropes: Overall low vs some concern or high risk of bias


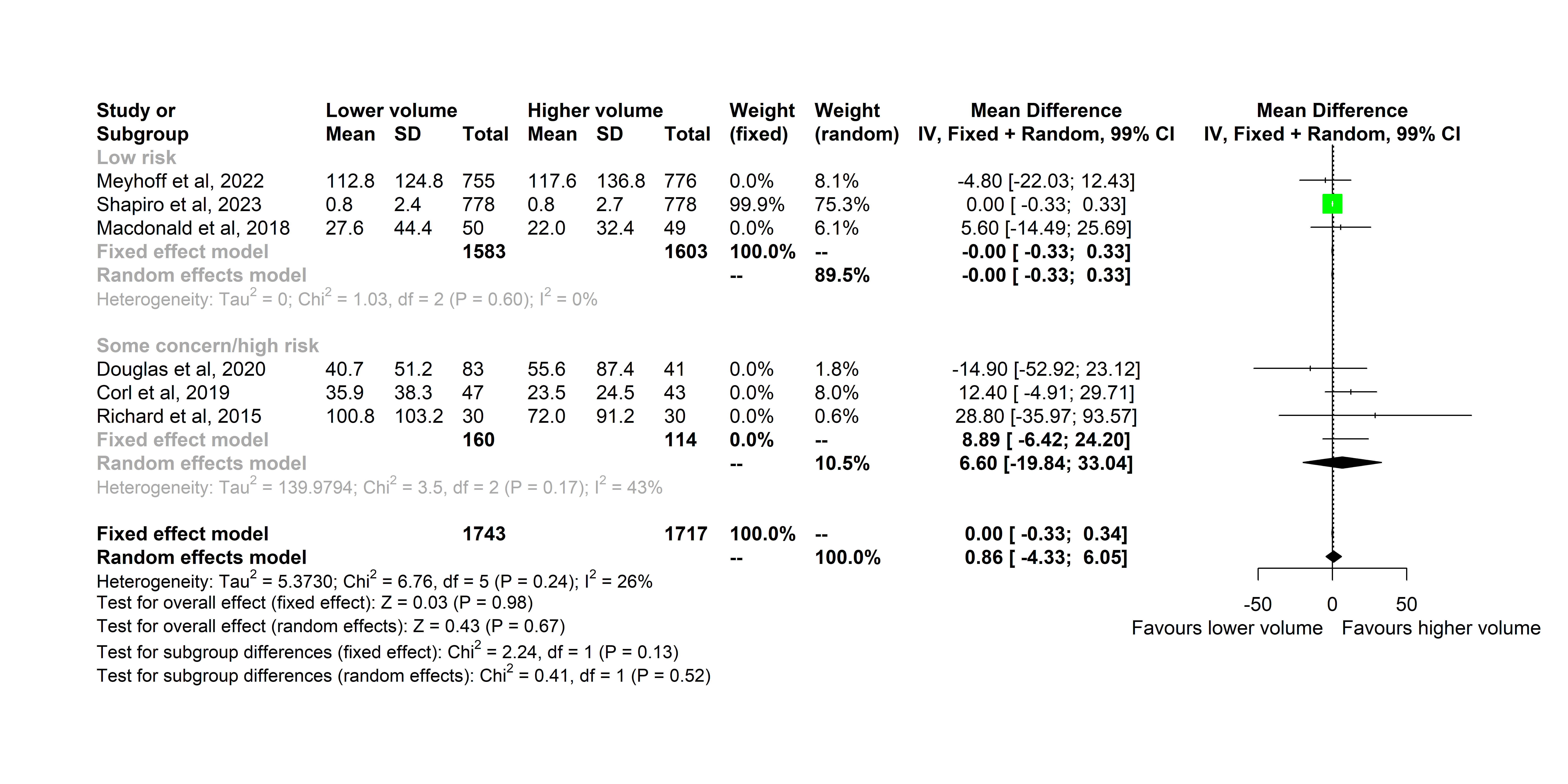


### Duration of vasopressor or inotropes: Patients with sepsis vs septic shock


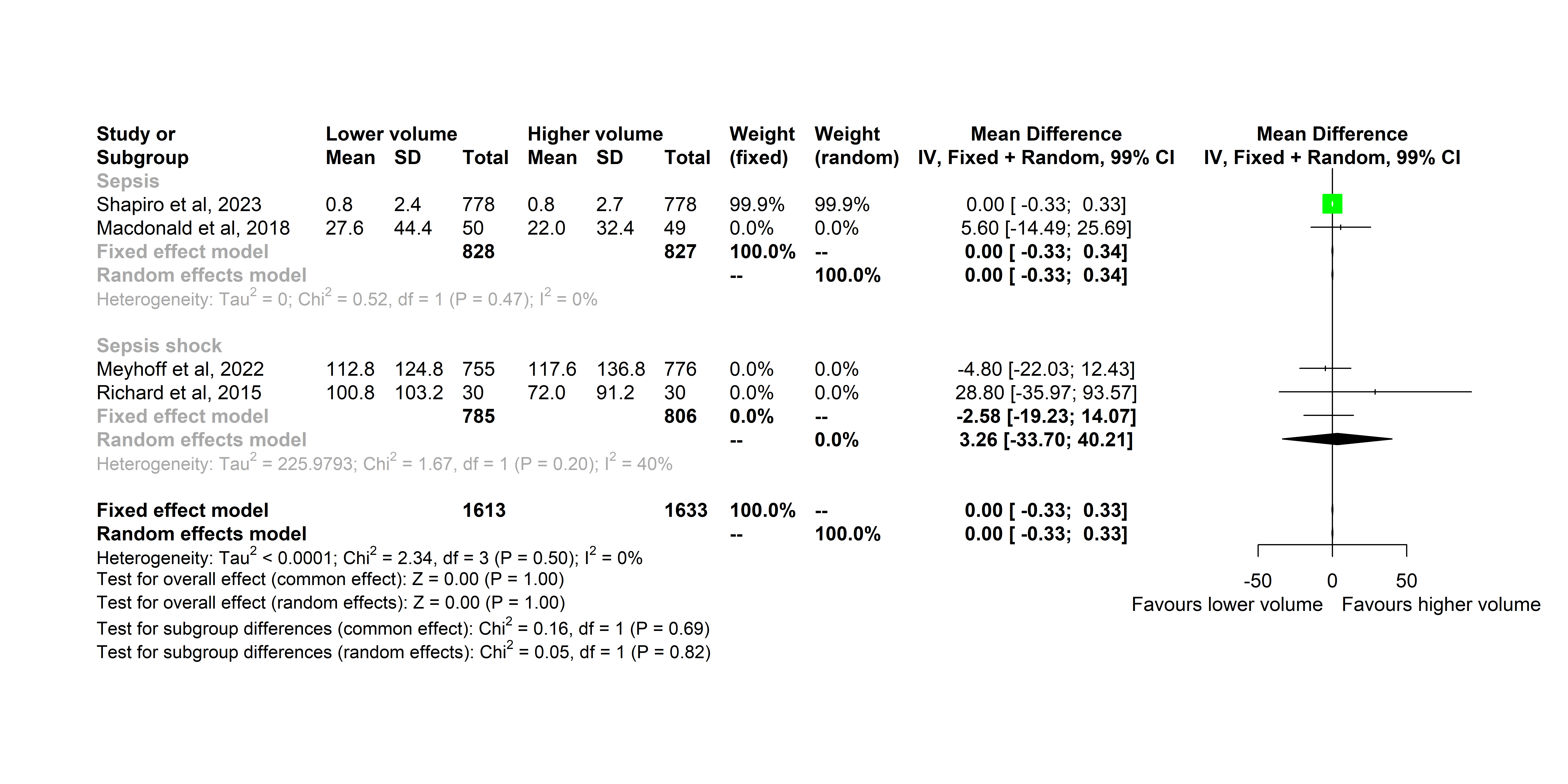


### Duration of vasopressor or inotropes: Fluid-only interventions vs a complex hemodynamic protocol


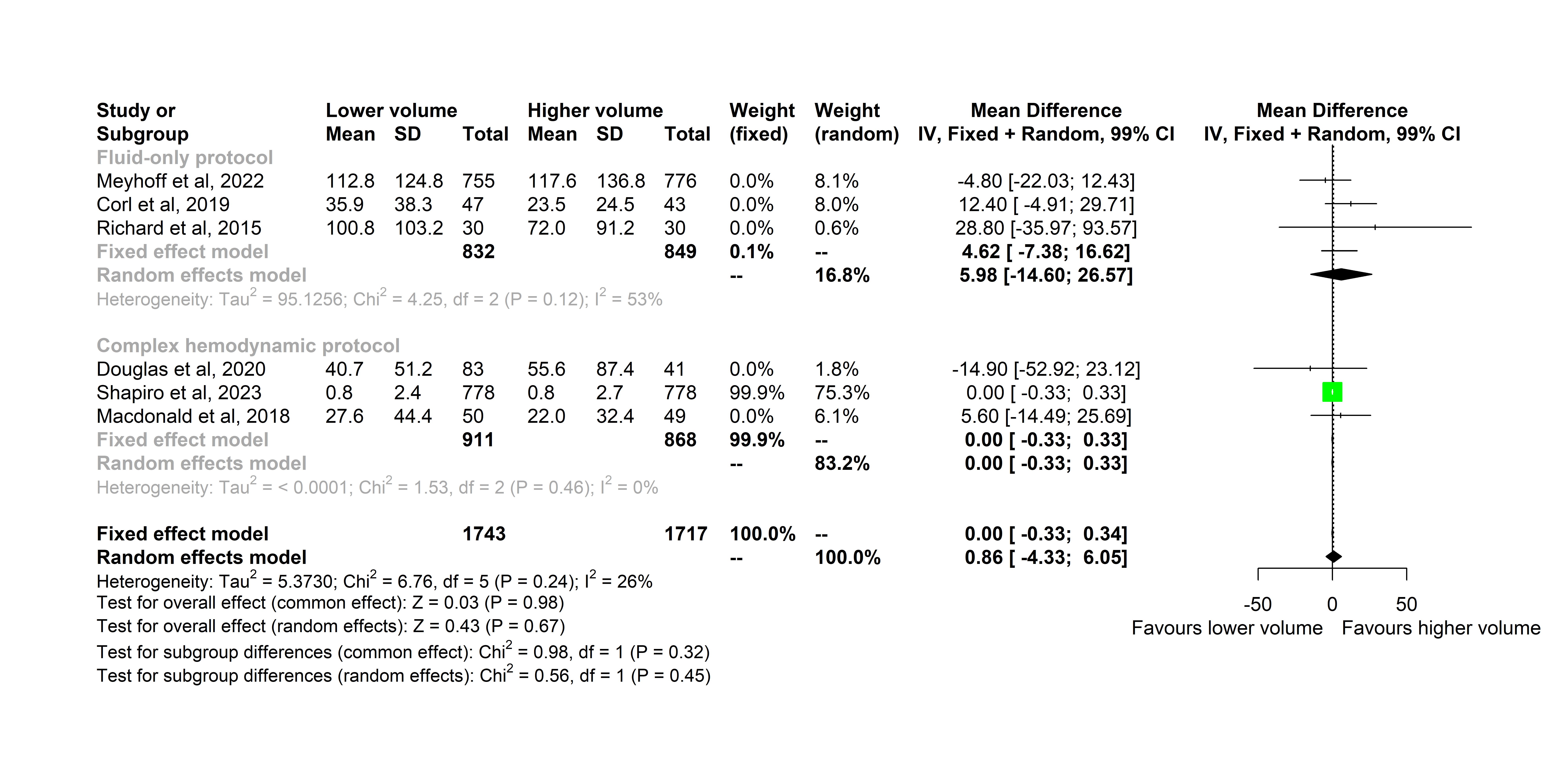


### Duration of vasopressor or inotropes: Early vs later resuscitation phase


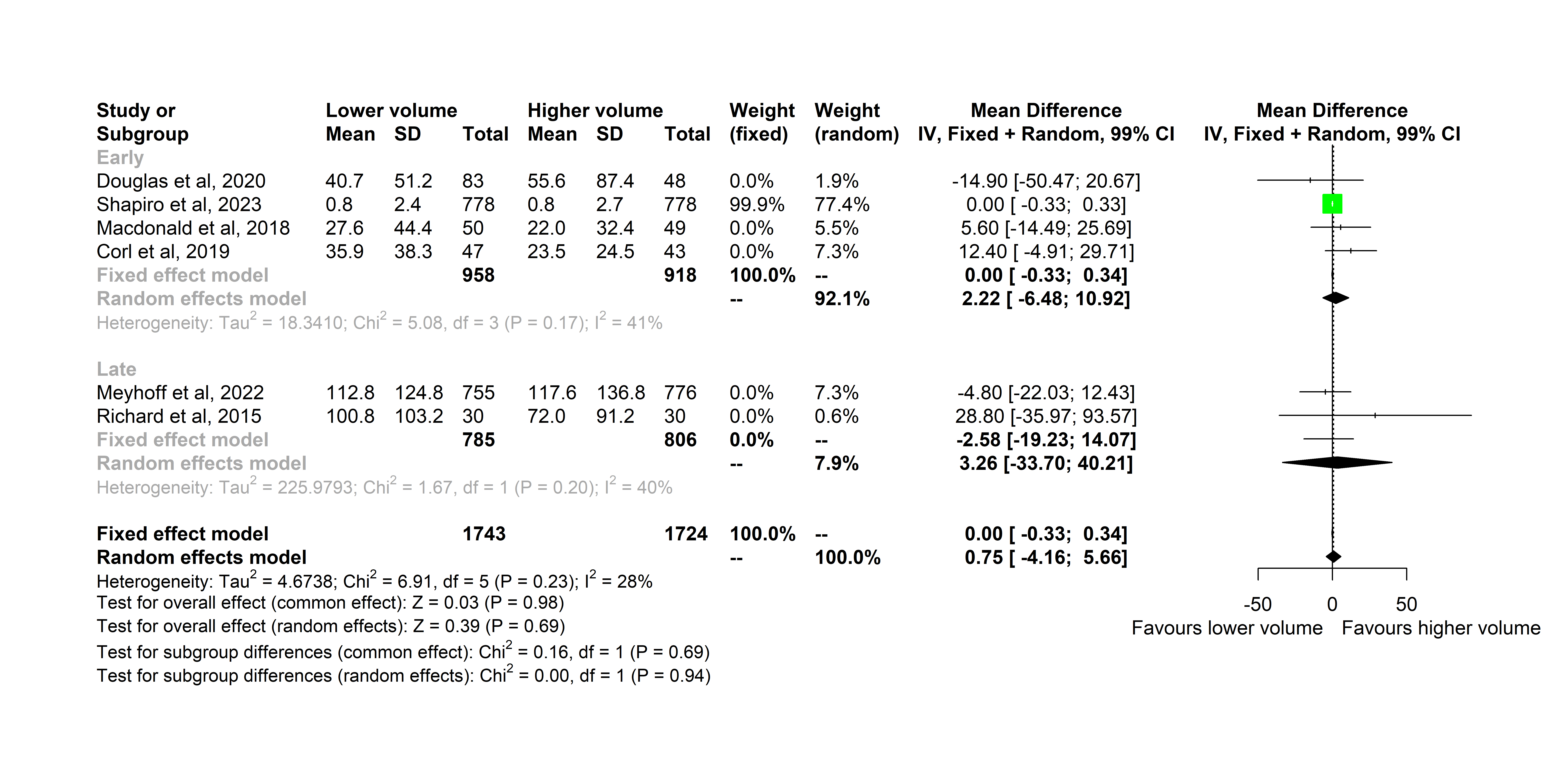


### 9.3.2 d) Subgroup analyses of vasopressor-free days

### Vasopressor-free days: Overall low vs some concern or high risk of bias


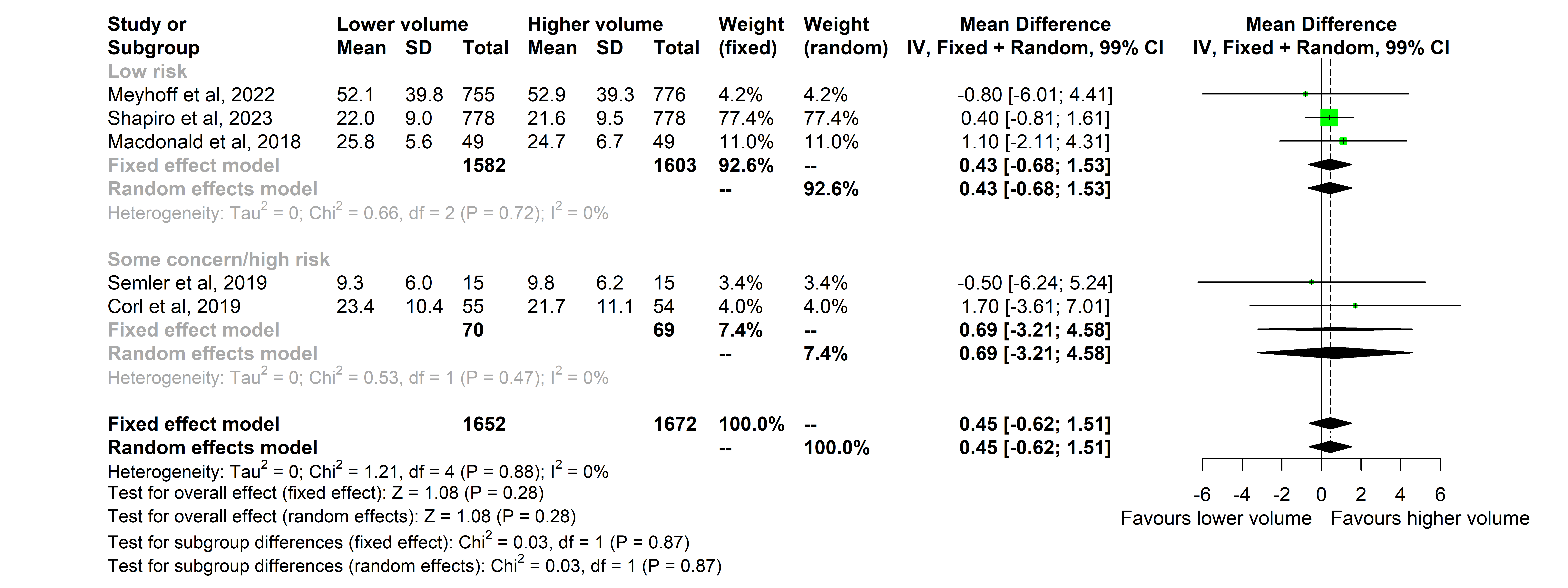


### Vasopressor-free days: Successful vs unsuccessful separation in fluid volumes


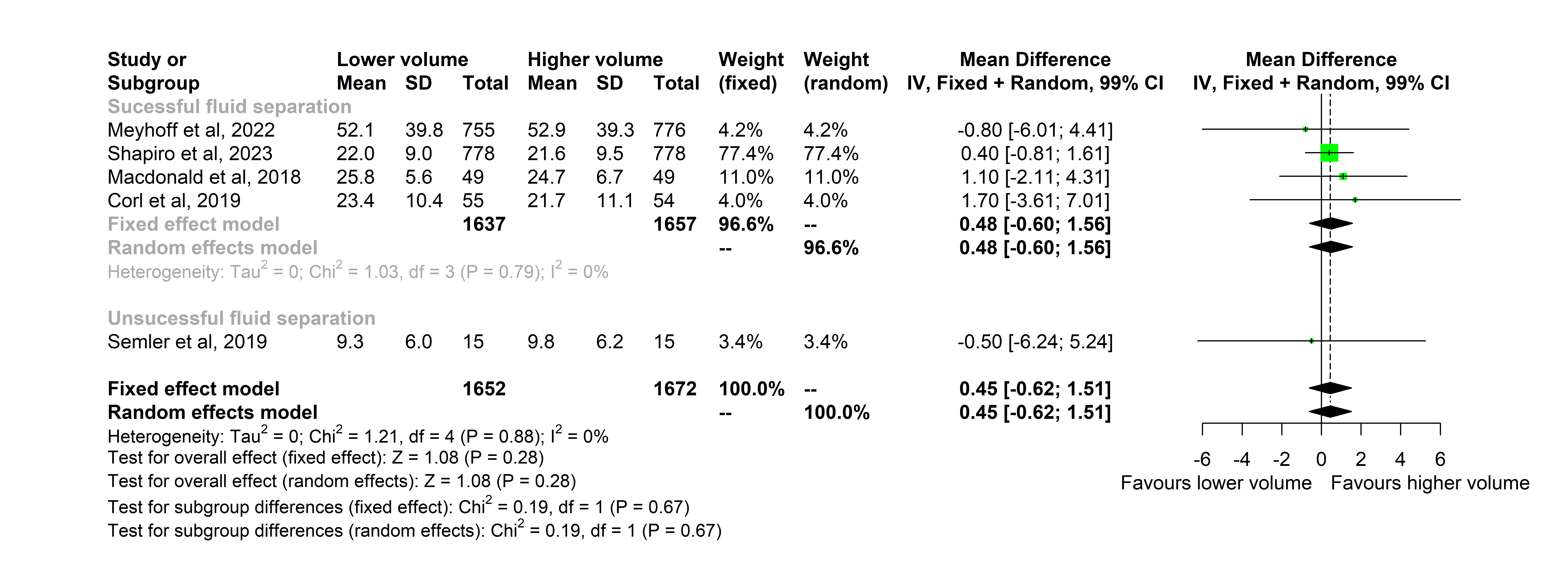


### Vasopressor-free days: Patients with sepsis vs septic shock


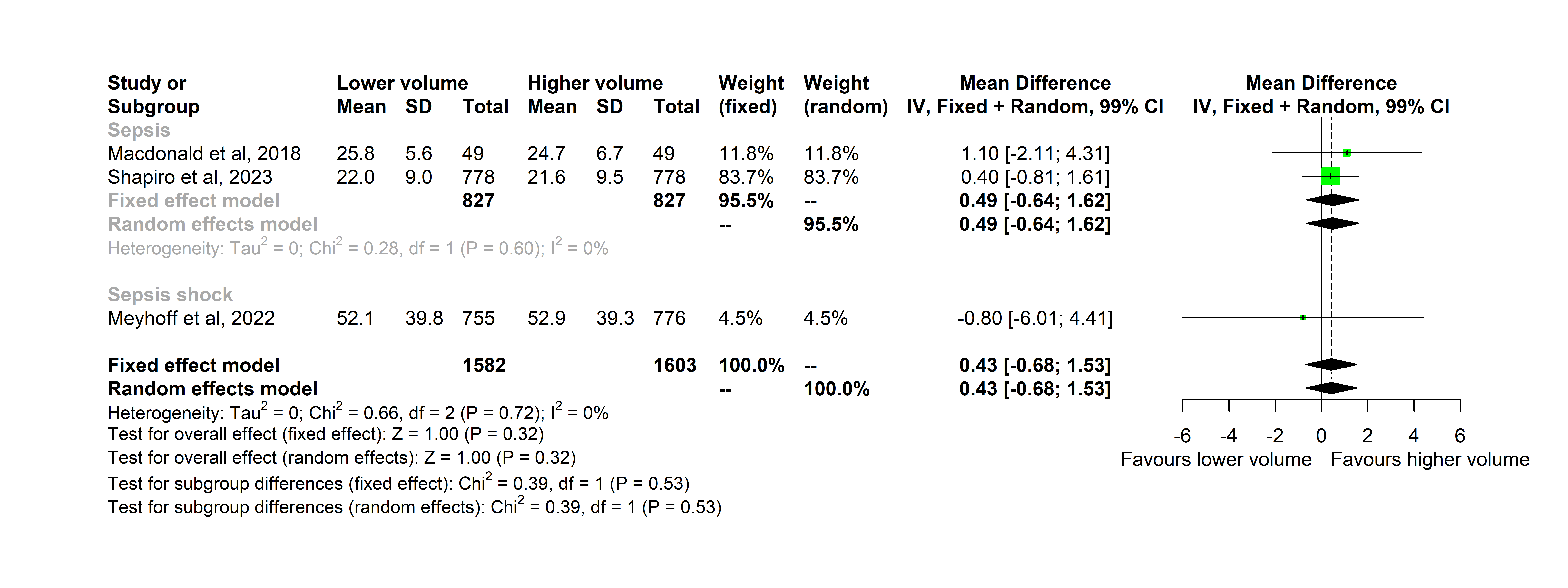


### Vasopressor-free days: Fluid-only interventions vs a complex hemodynamic protocol


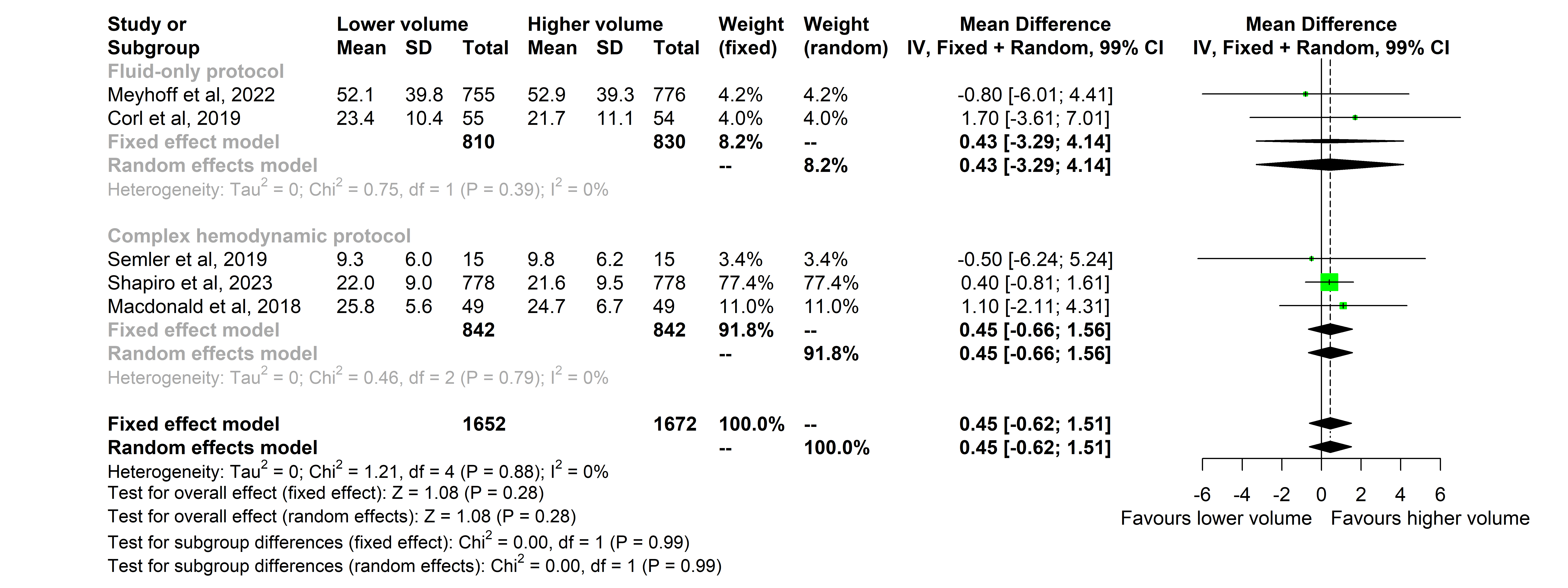


### Vasopressor-free days: Early vs later resuscitation phase of sepsis


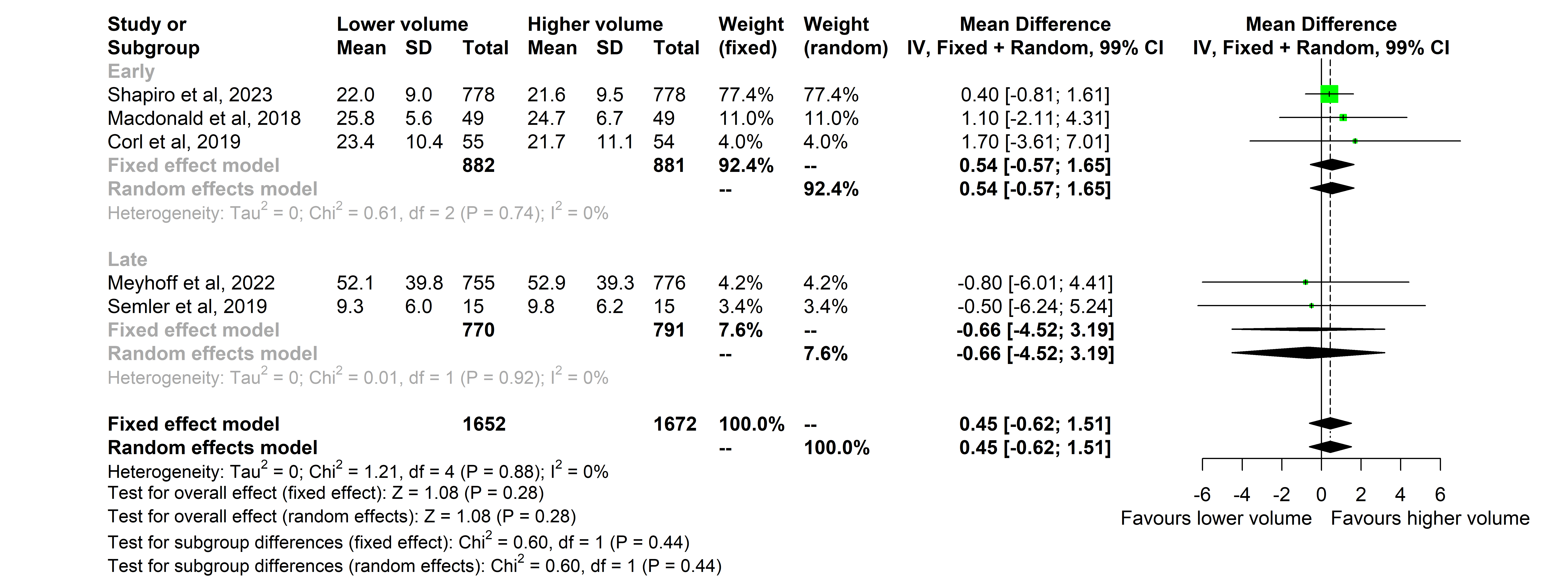


### 9.2.2 e) Subgroup analyses of the use of renal replacement therapy

### Use of renal replacement therapy: Overall low vs some concern or high risk of bias


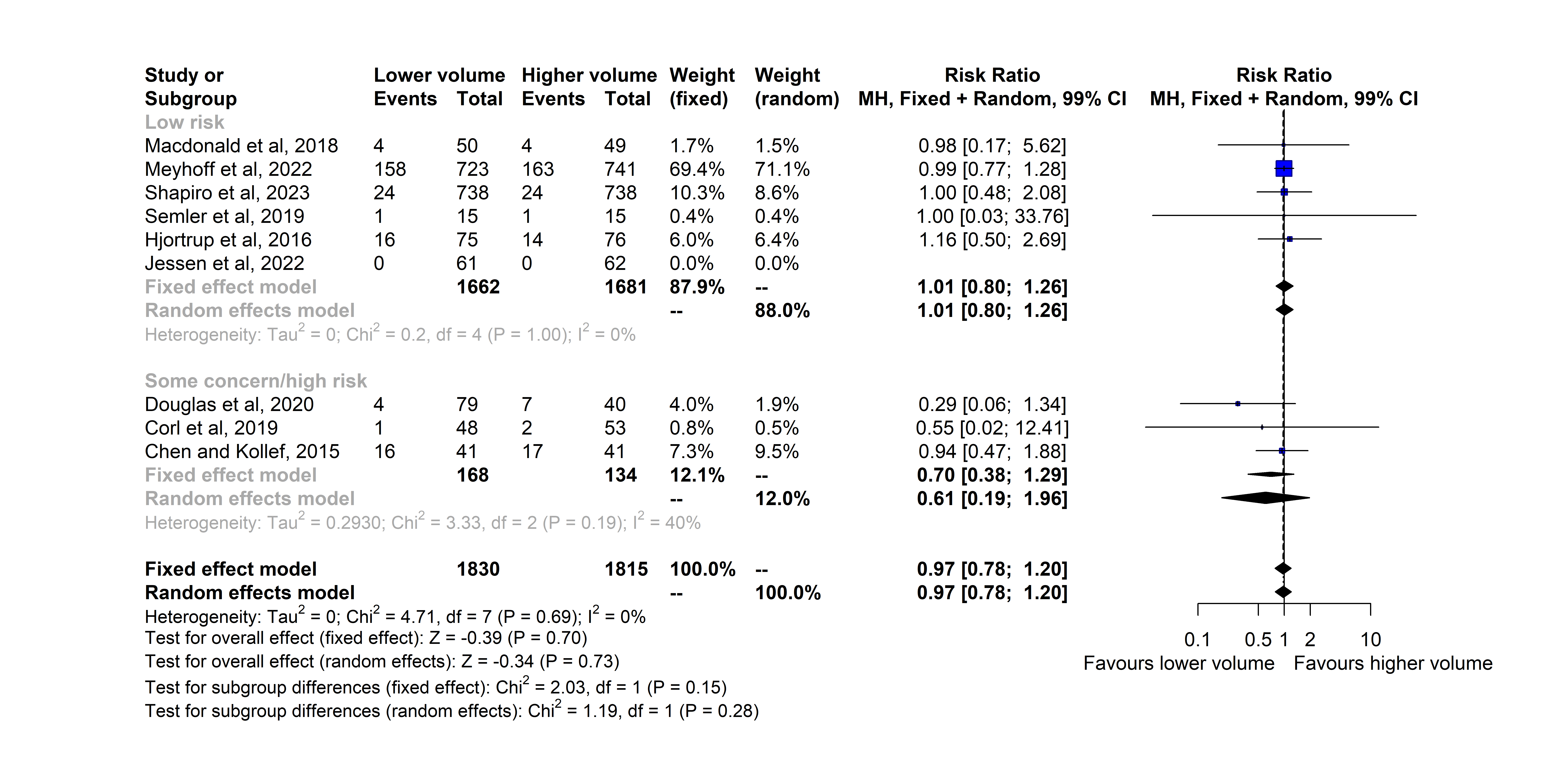


### Use of renal replacement therapy: Successful vs unsuccessful separation in fluid volumes


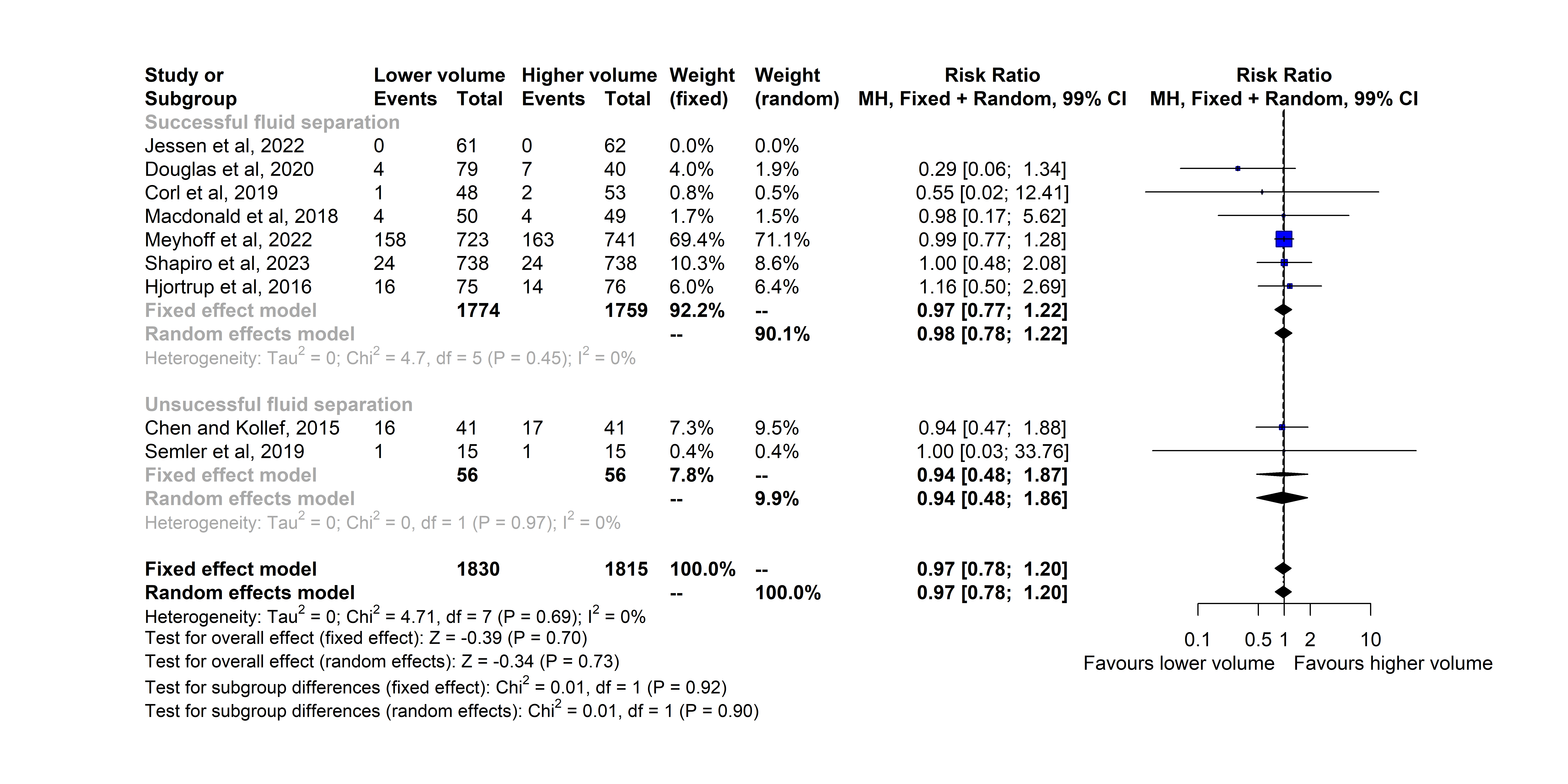


### Use of renal replacement therapy: Patients with sepsis vs septic shock


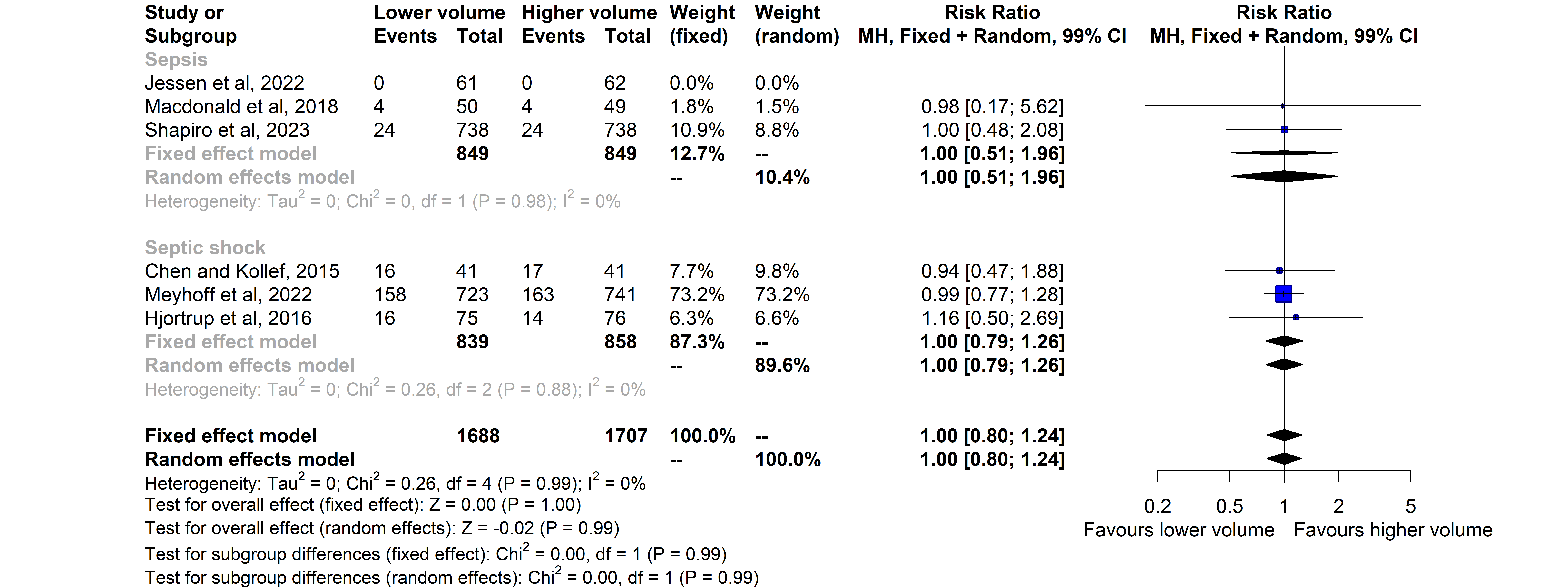


### Use of renal replacement therapy: Fluid-only interventions vs a complex hemodynamic protocol


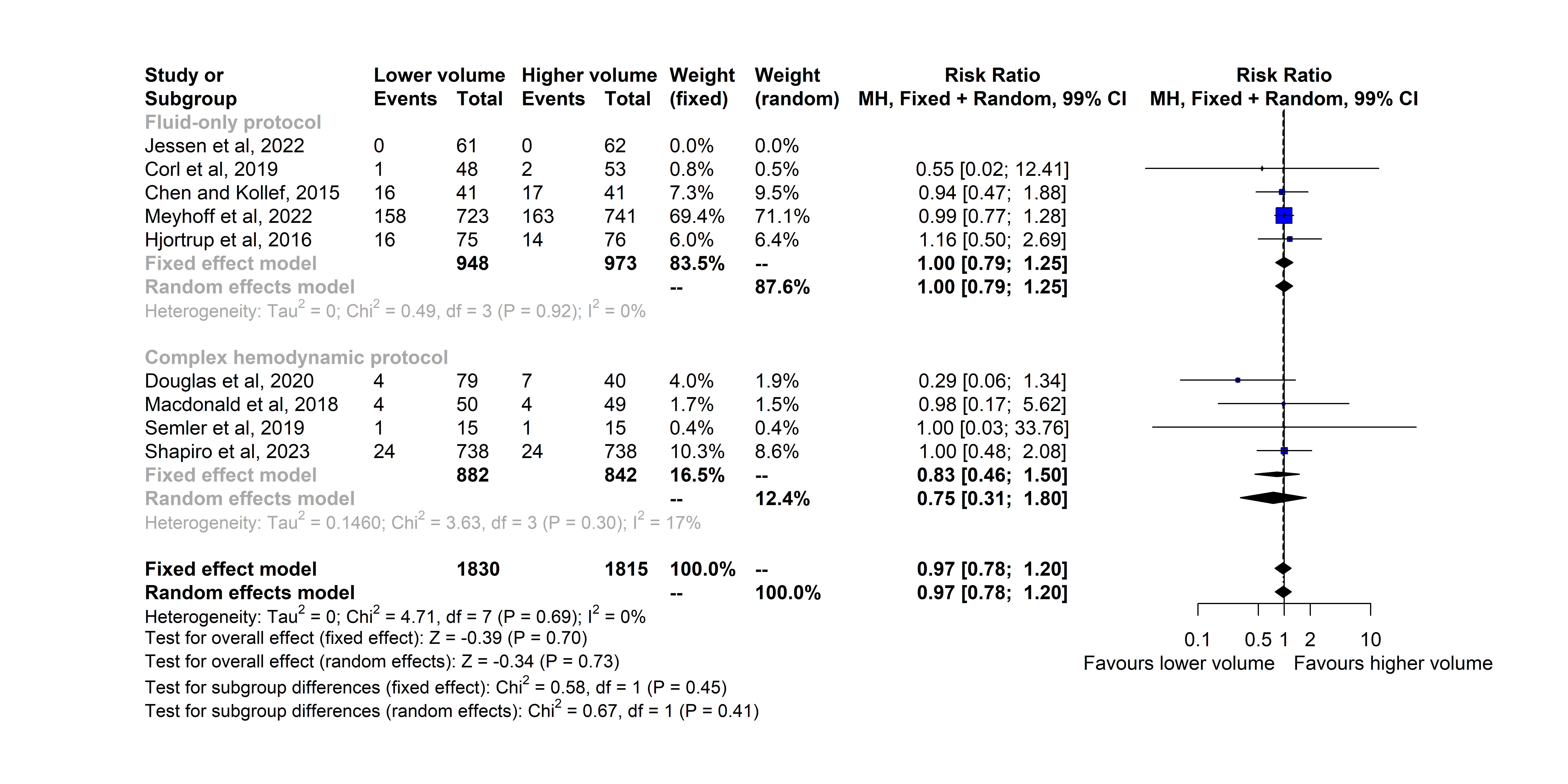


### Use of renal replacement therapy: Early vs later resuscitation phase of sepsis


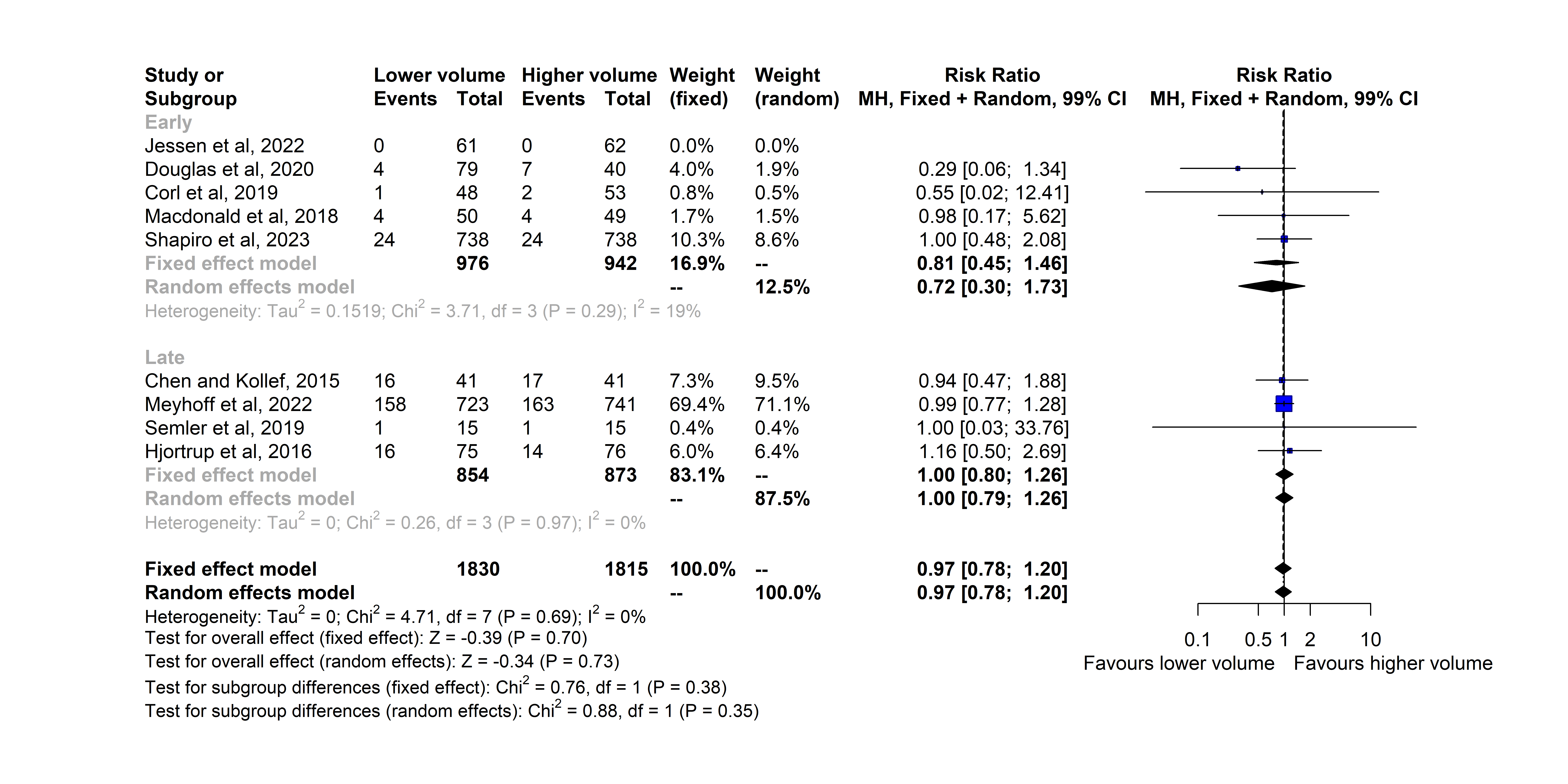


### 9.2.2 f) Subgroup analyses of the duration of renal replacement therapy (all trials low risk of bias trials)

### Duration of renal replacement therapy: Patients with sepsis vs septic shock


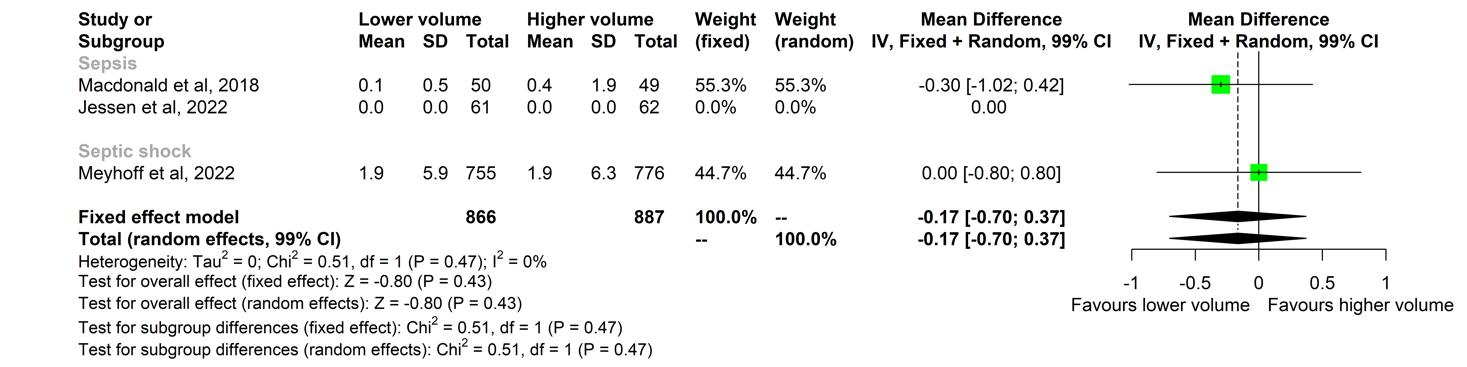


### Duration of renal replacement therapy: Fluid-only interventions vs a complex hemodynamic protocol


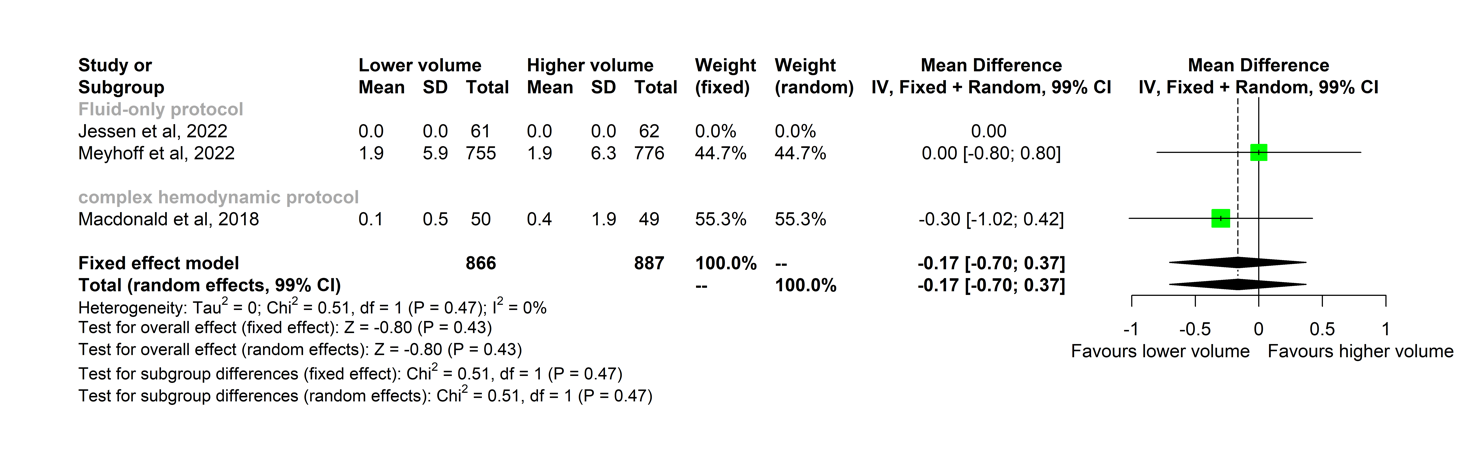


### Duration of renal replacement therapy: Early vs later resuscitation phase of sepsis


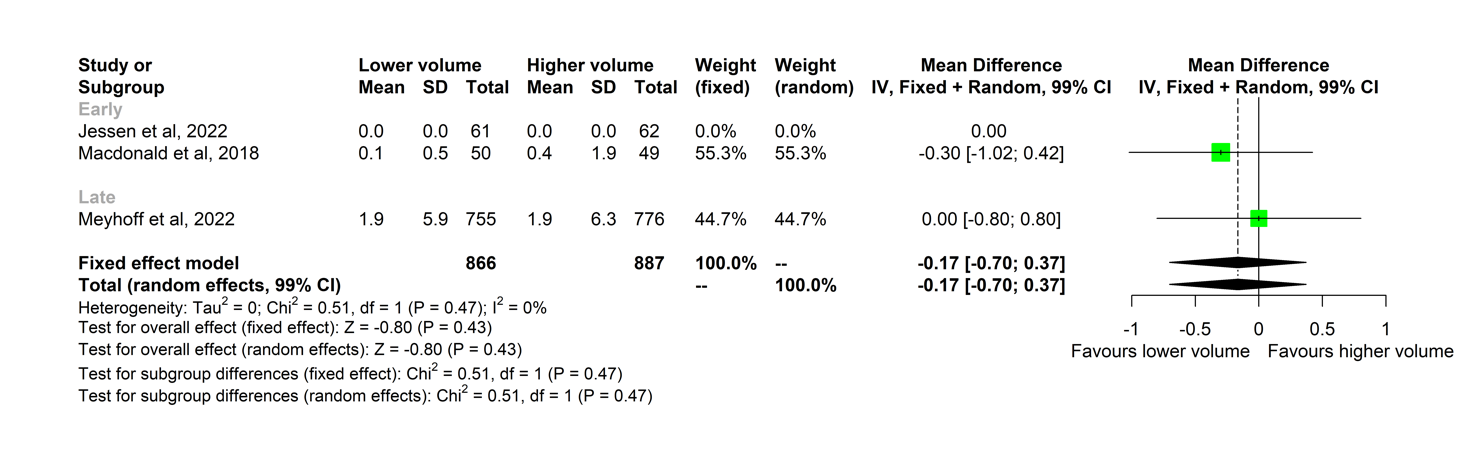


### 9.2.2 g) Subgroup analyses of renal replacement therapy free days (all trials low risk of bias trials)

### Renal replacement therapy free days: Successful vs unsuccessful separation in fluid volumes


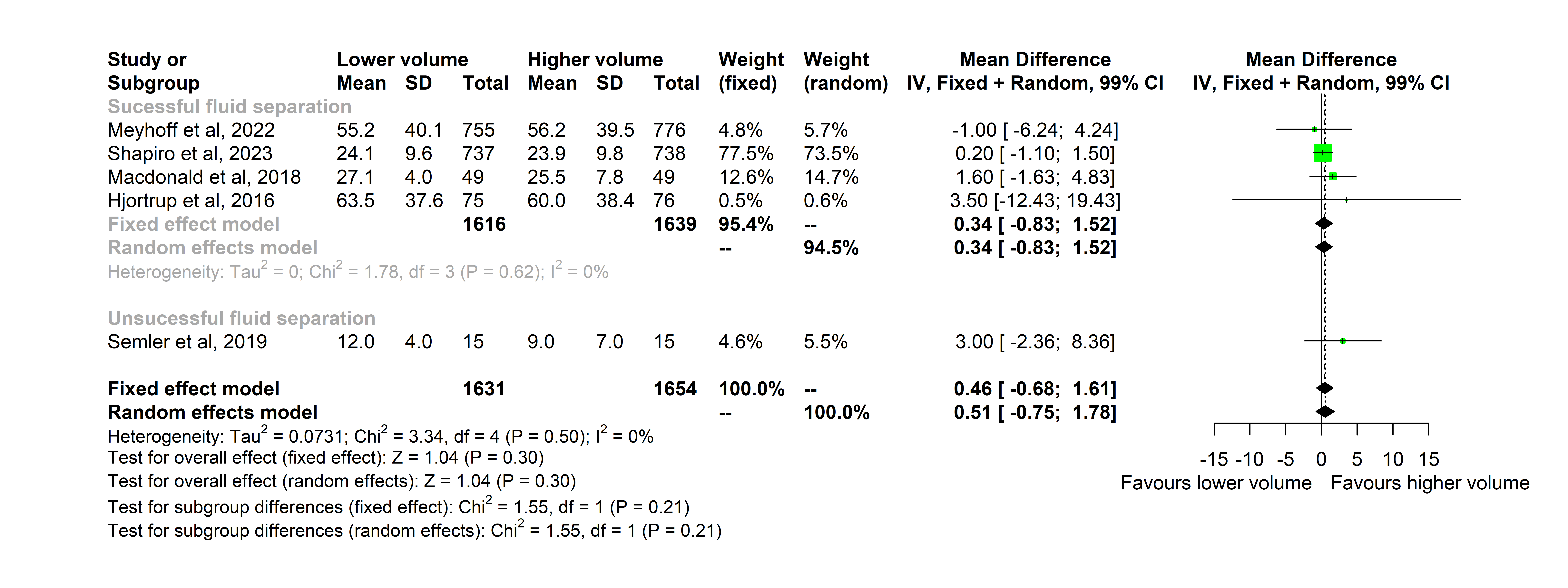


### Renal replacement therapy free days: Patients with sepsis vs septic shock


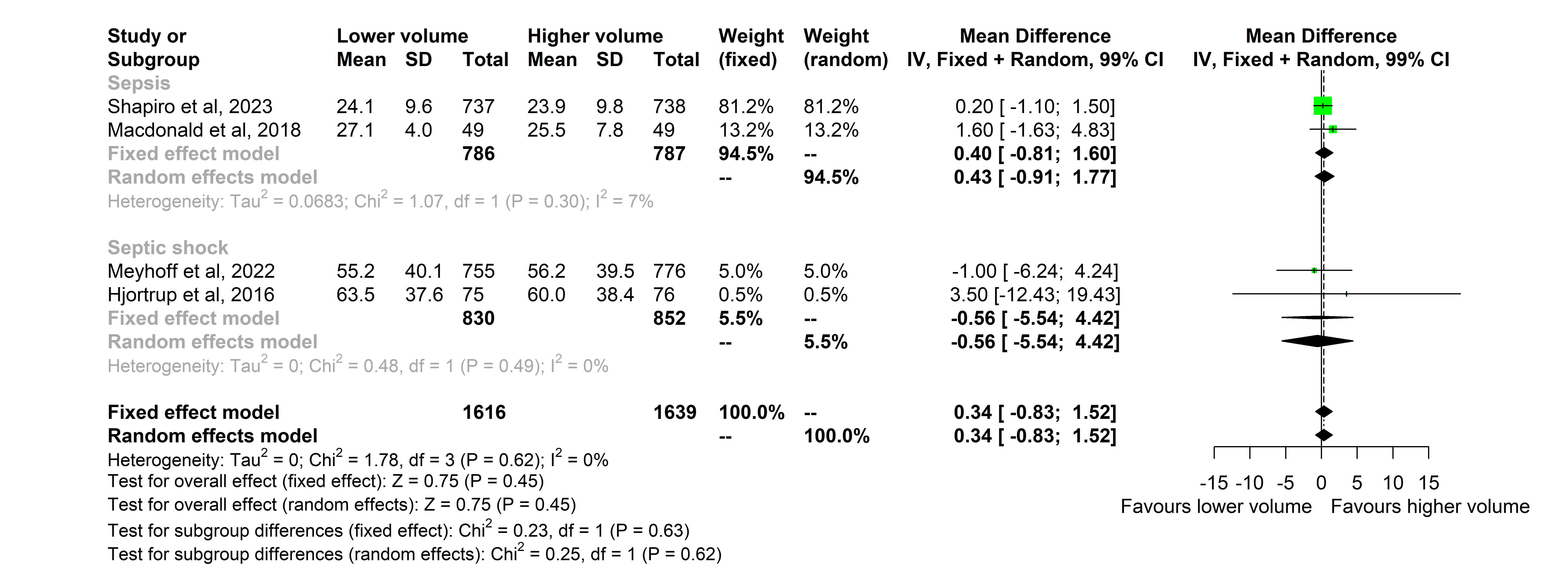


### Renal replacement therapy free days: Fluid-only interventions vs a complex hemodynamic protocol


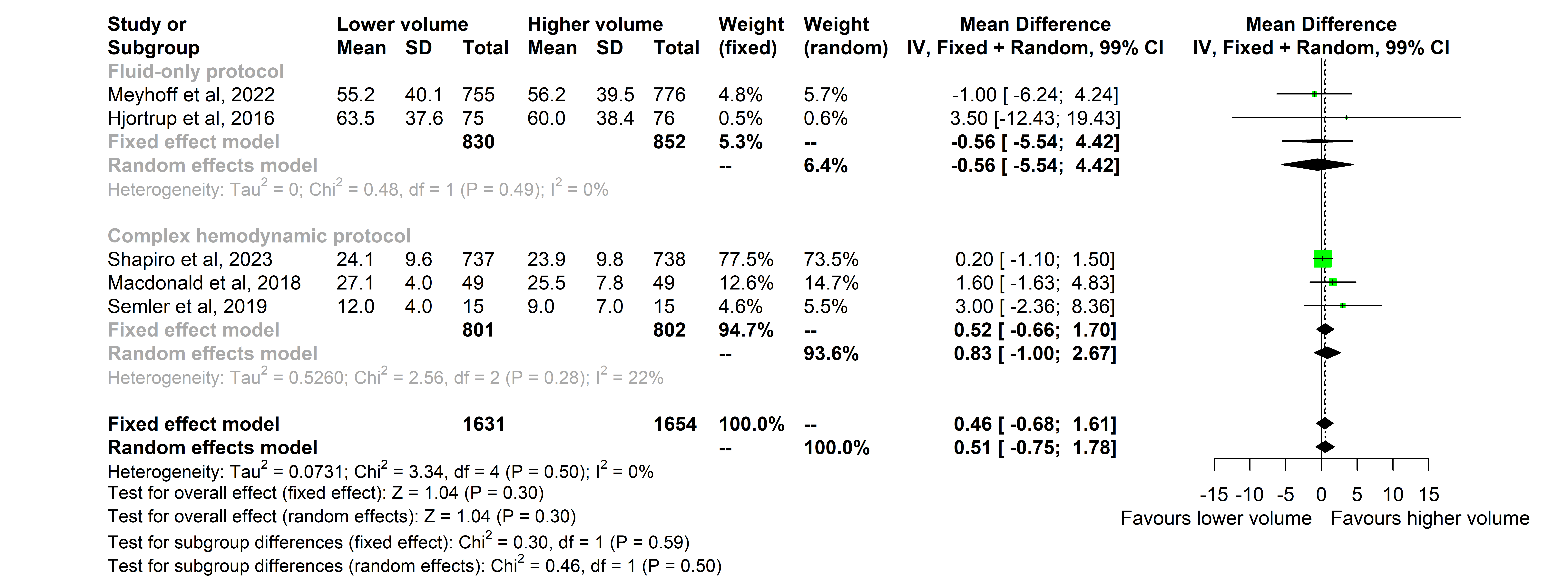


### Renal replacement therapy free days: Early vs later resuscitation phase of sepsis


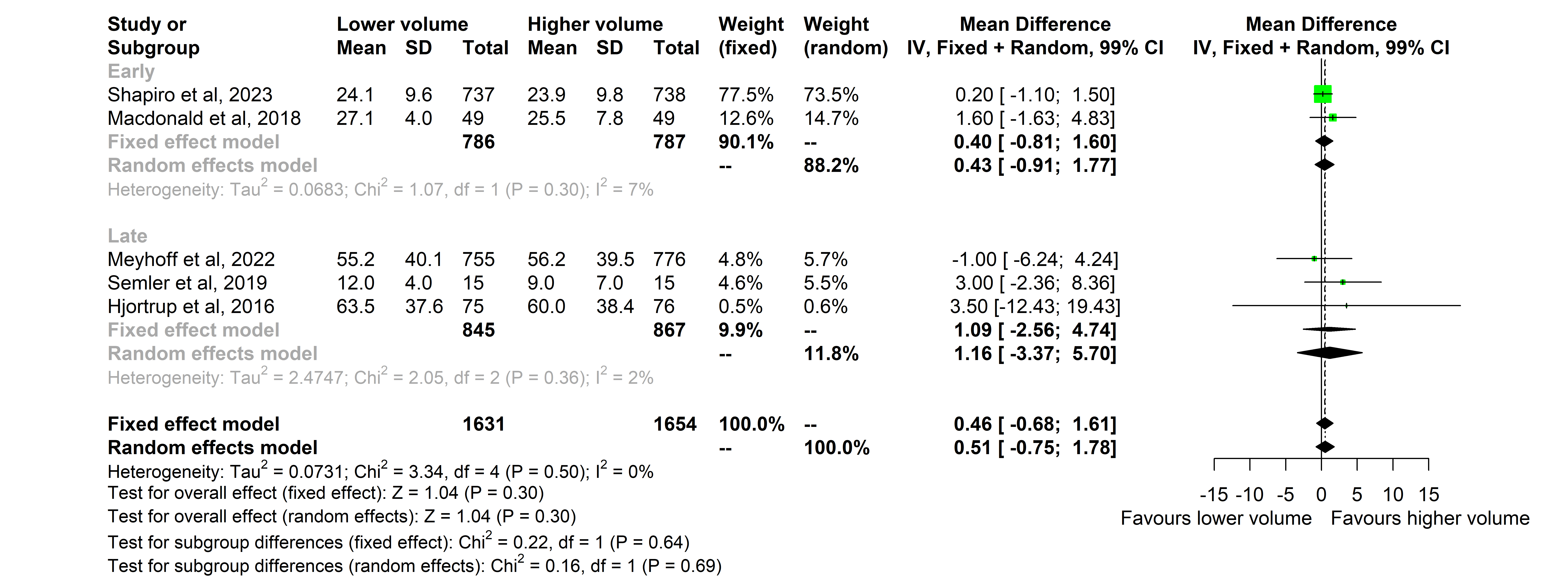


### 9.2.2 h) Subgroup analyses of incidence of acute kidney injury

### Incidence of acute kidney injury: Overall low vs some concern or high risk of bias


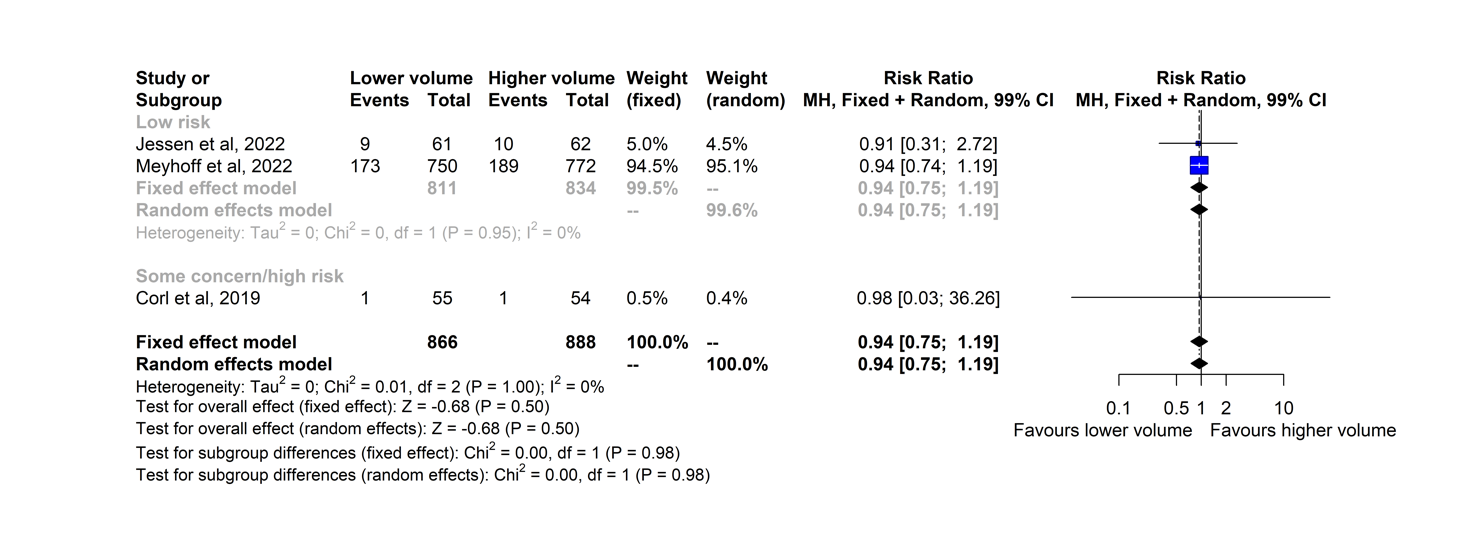


### Incidence of acute kidney injury: Patients with sepsis vs septic shock


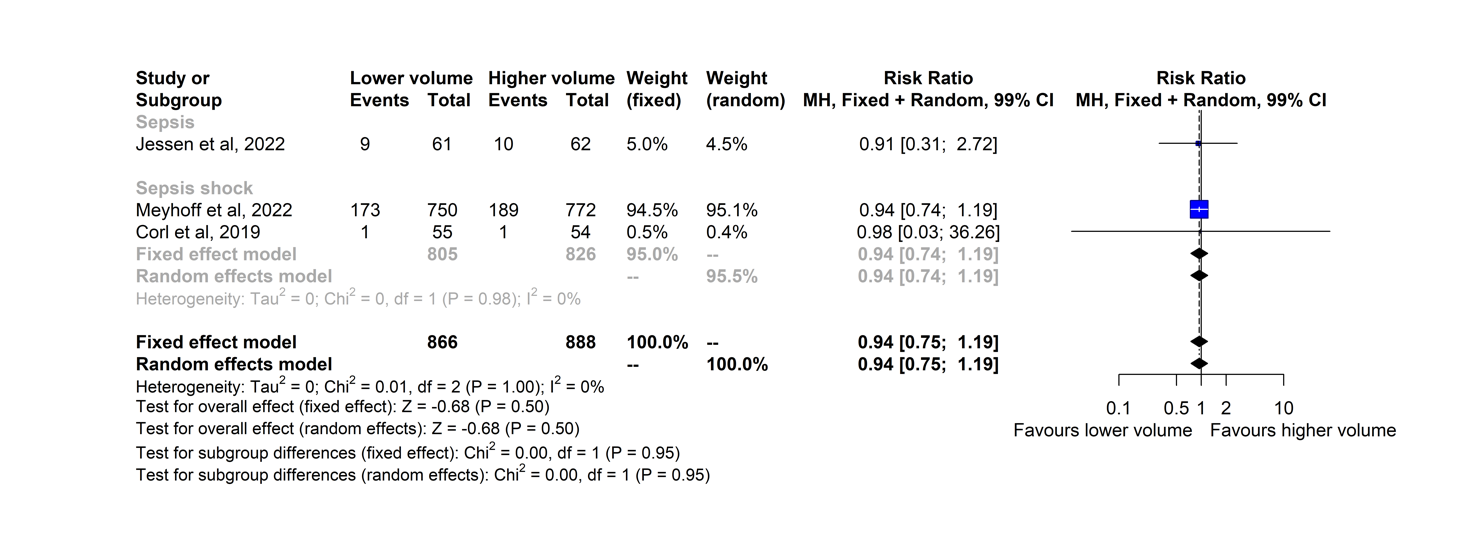


### Incidence of acute kidney injury: early vs later resuscitation phase of sepsis


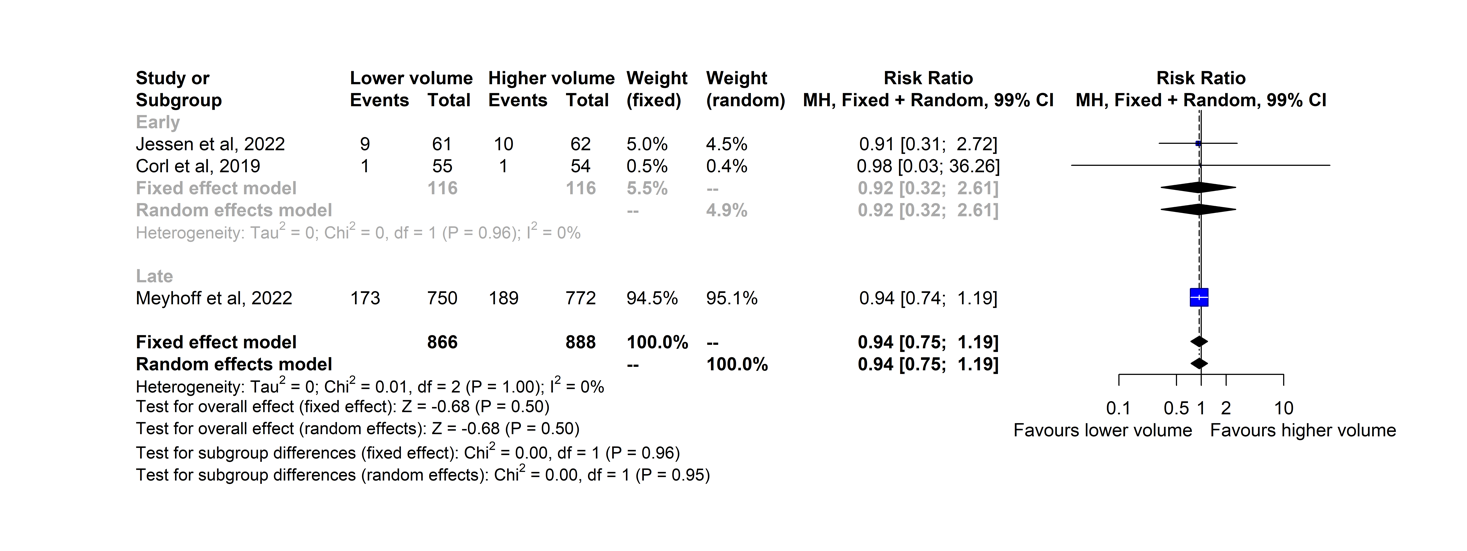


### 9.2.3 Exploratory outcomes

### 9.2.3 a) Subgroup analyses of the use of blood products

### Use of blood products: Overall low vs some concern or high risk of bias


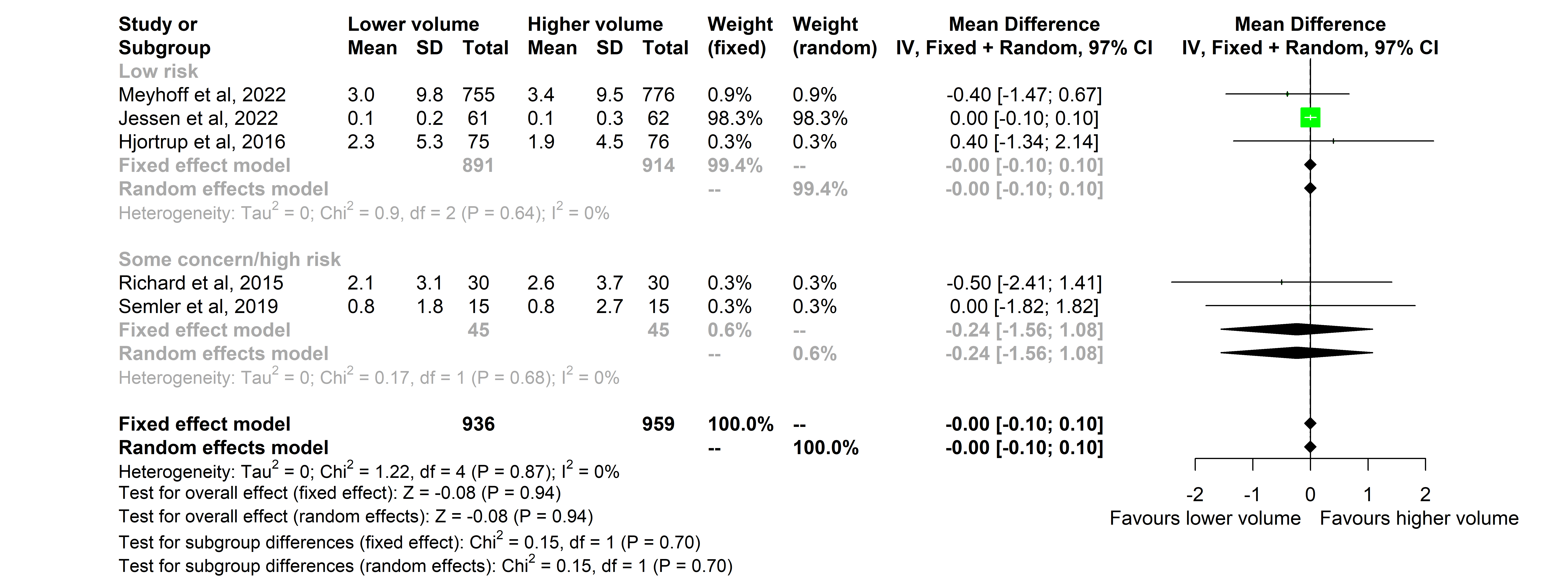


### Use of blood products: Successful vs unsuccessful separation in fluid volumes


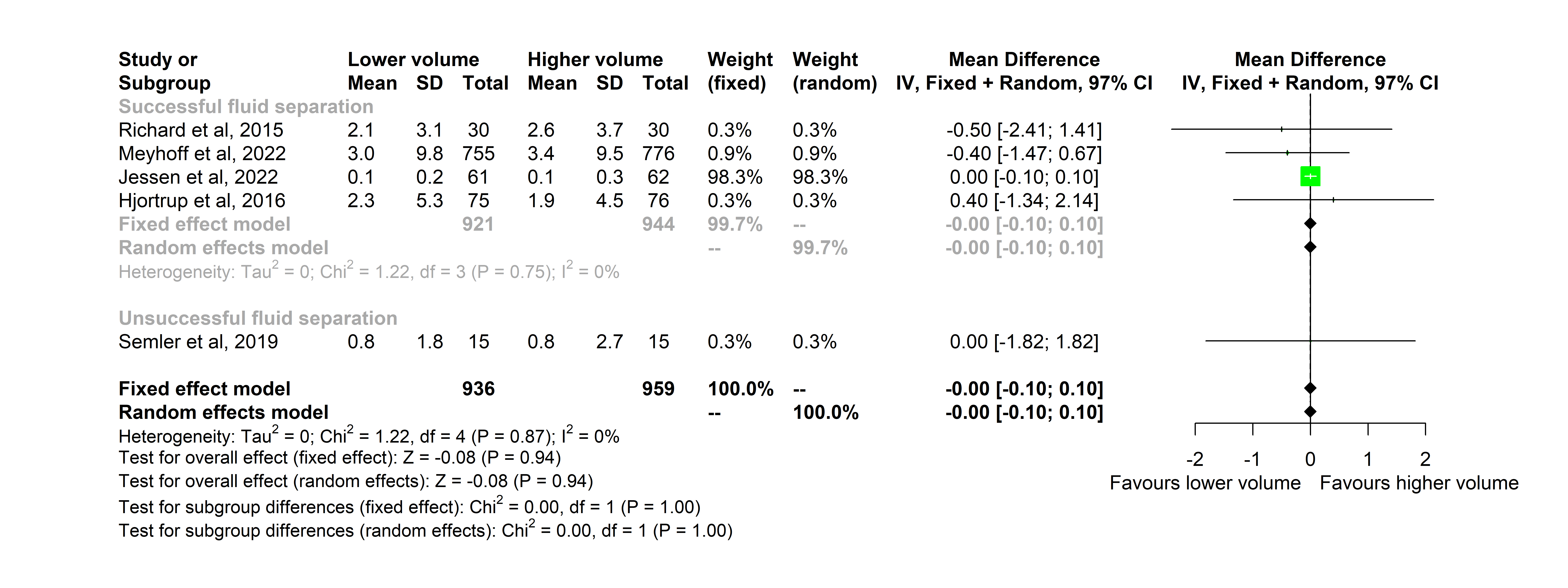


### Use of blood products: Patients with sepsis vs septic shock


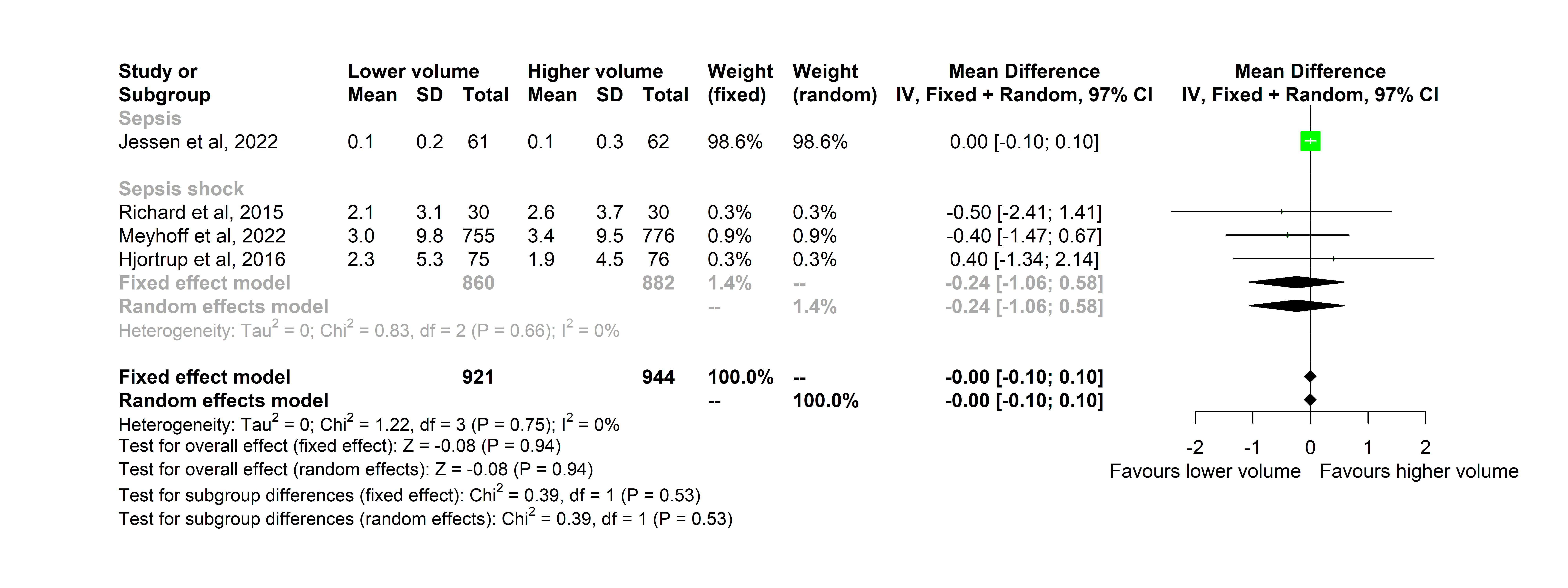


### Use of blood products: Fluid-only interventions vs a complex hemodynamic protocol


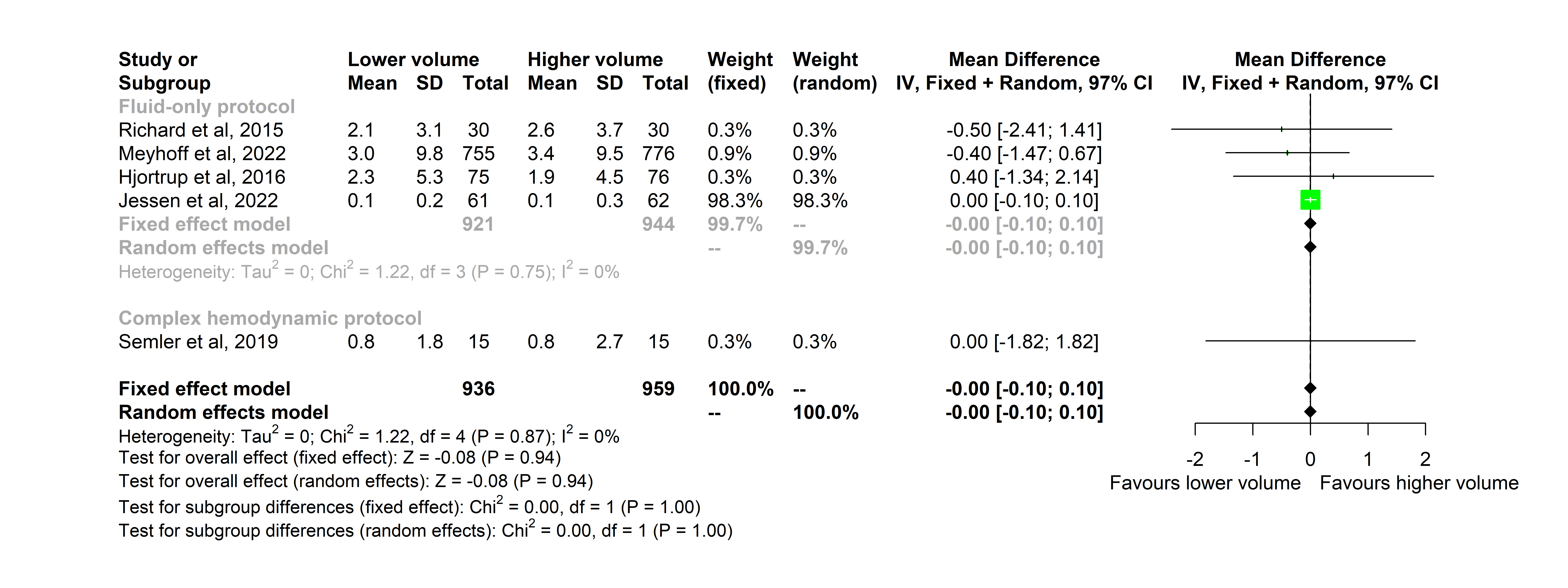


### Use of blood products: Early vs later resuscitation phase of sepsis


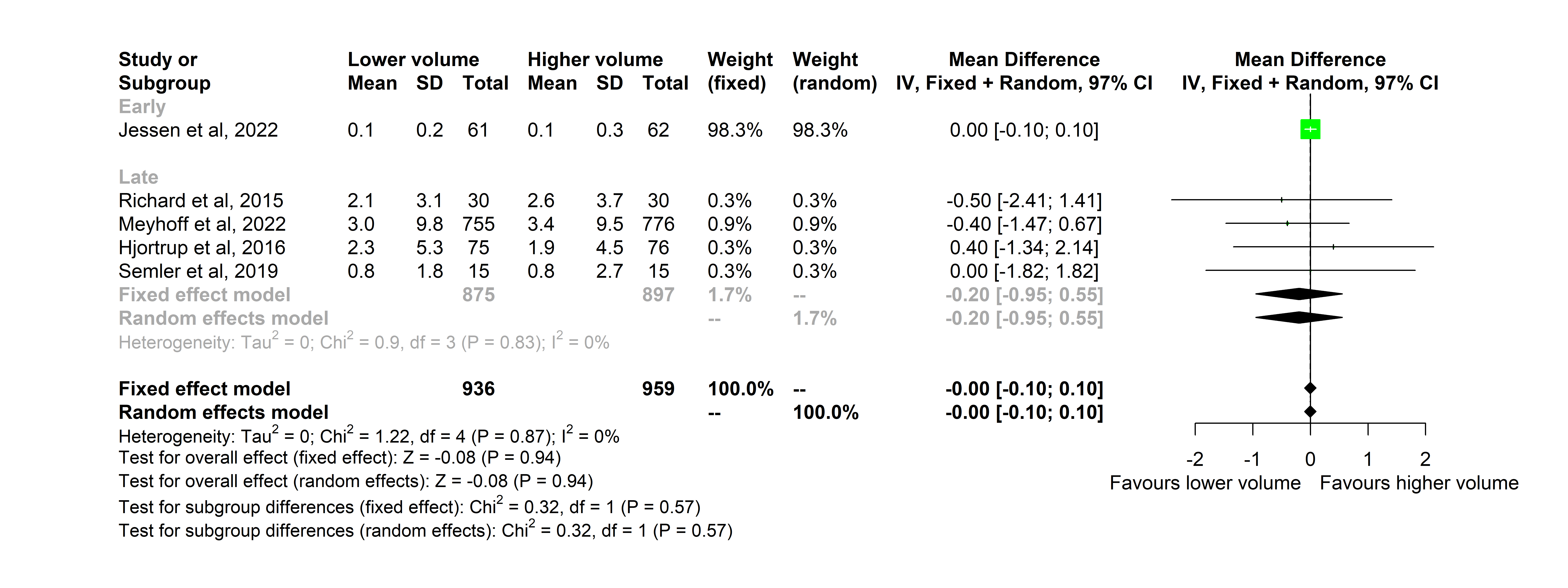


### 9.2.3 b) Subgroup analyses of ICU length of stay

### ICU length of stay: Overall low vs some concern or high risk of bias


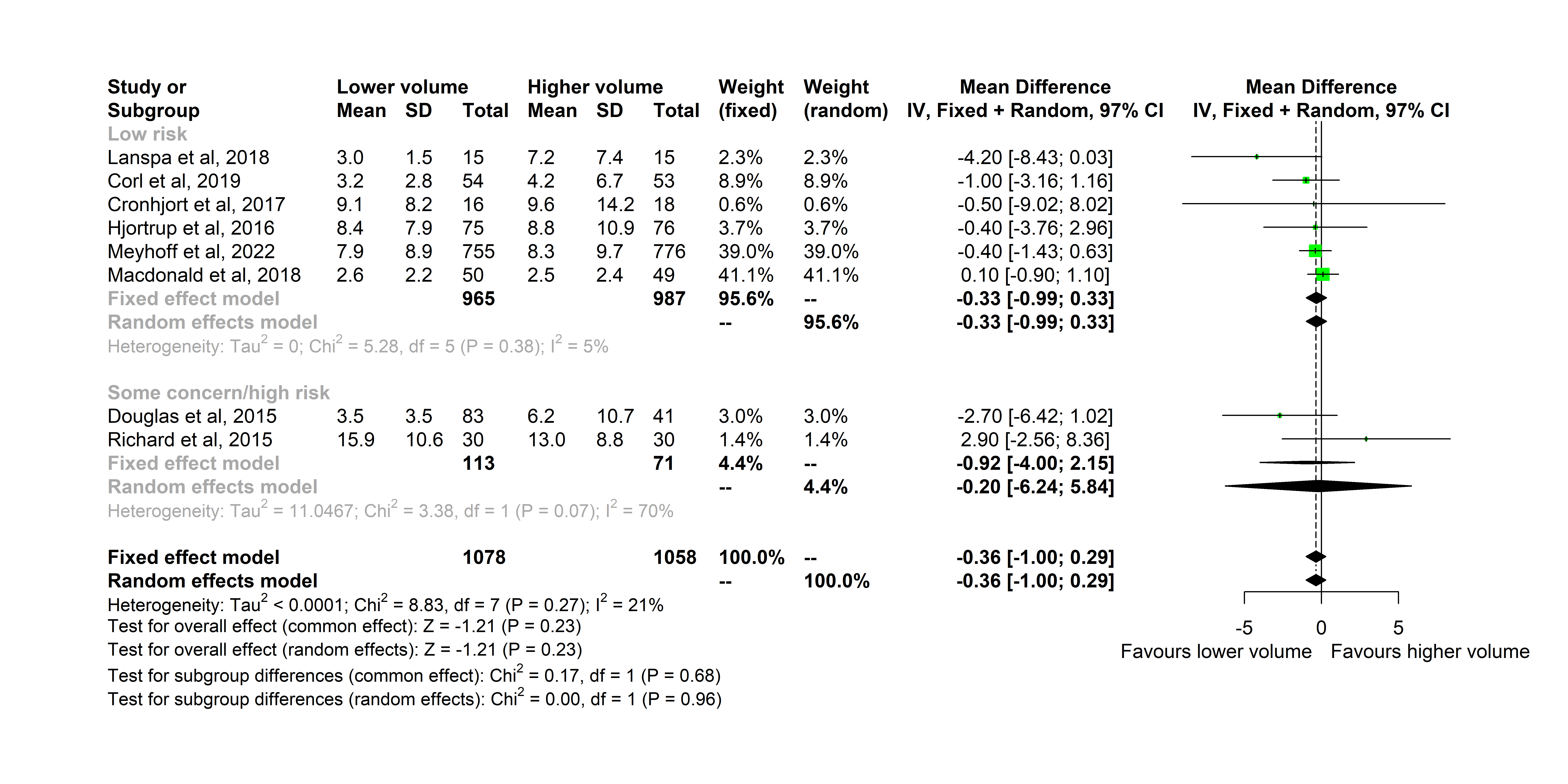


### ICU length of stay: Successful vs unsuccessful separation in fluid volumes


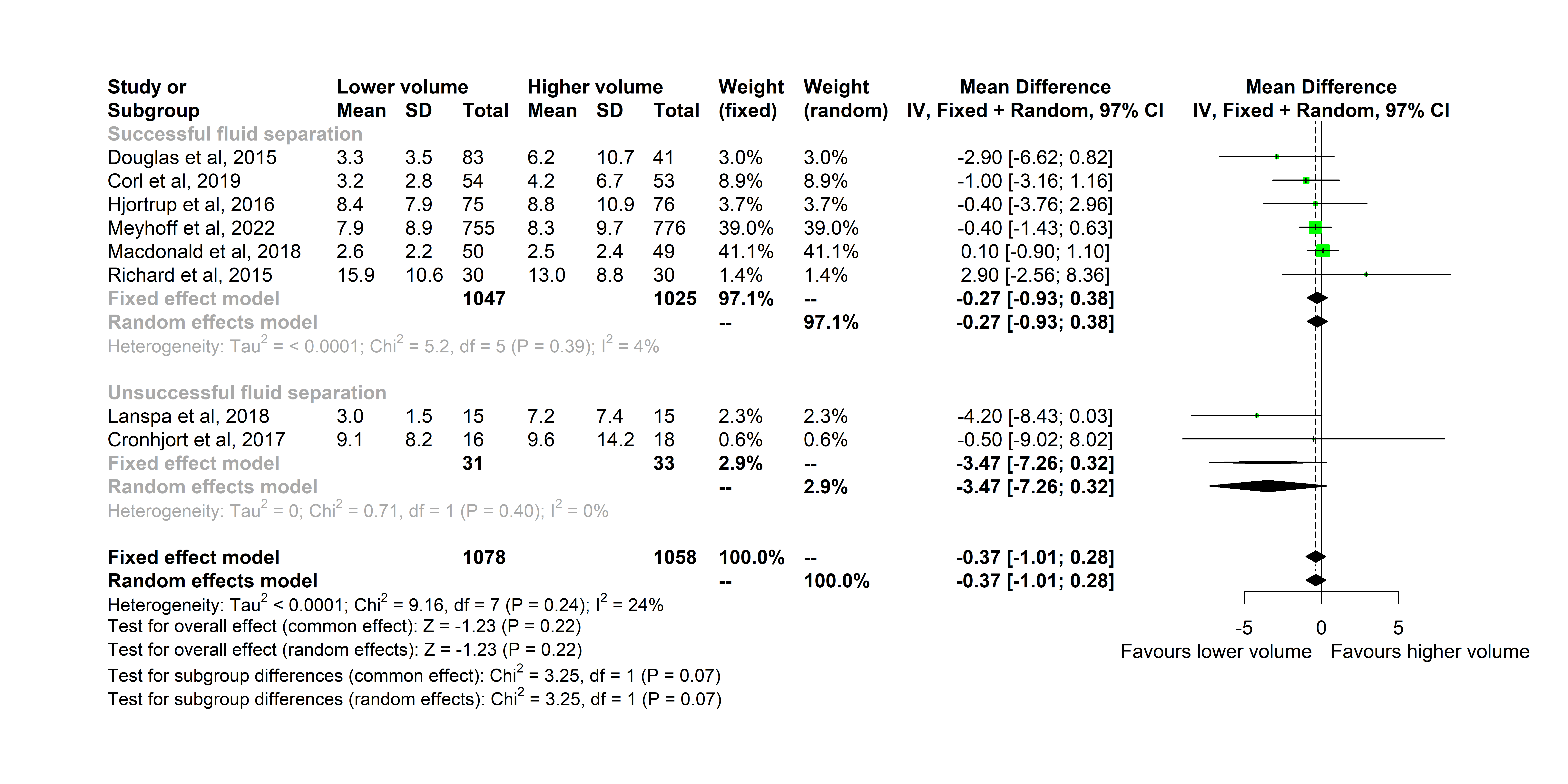


### ICU length of stay: Patients with sepsis vs septic shock


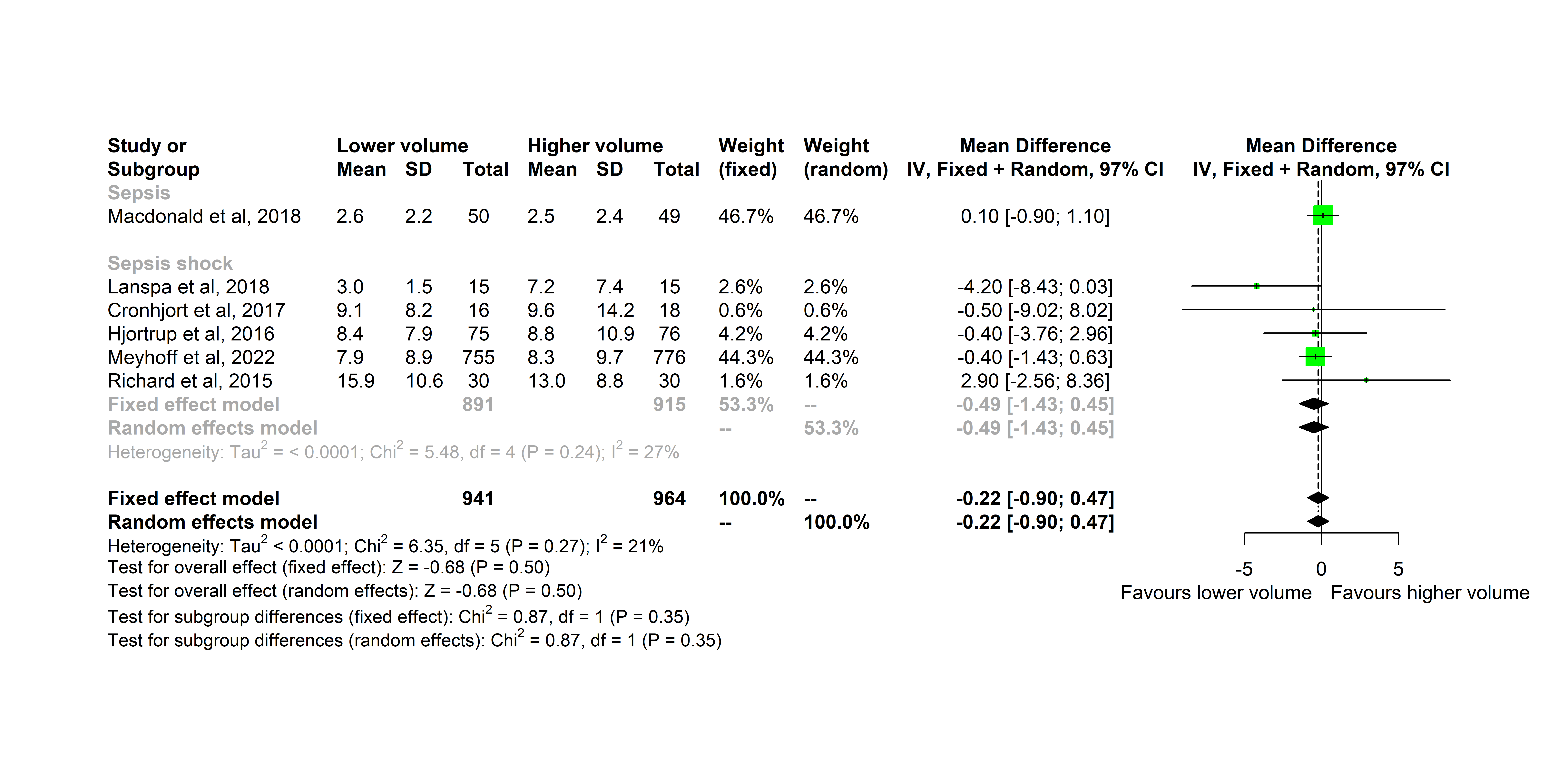


### ICU length of stay: Fluid-only interventions vs a complex hemodynamic protocol


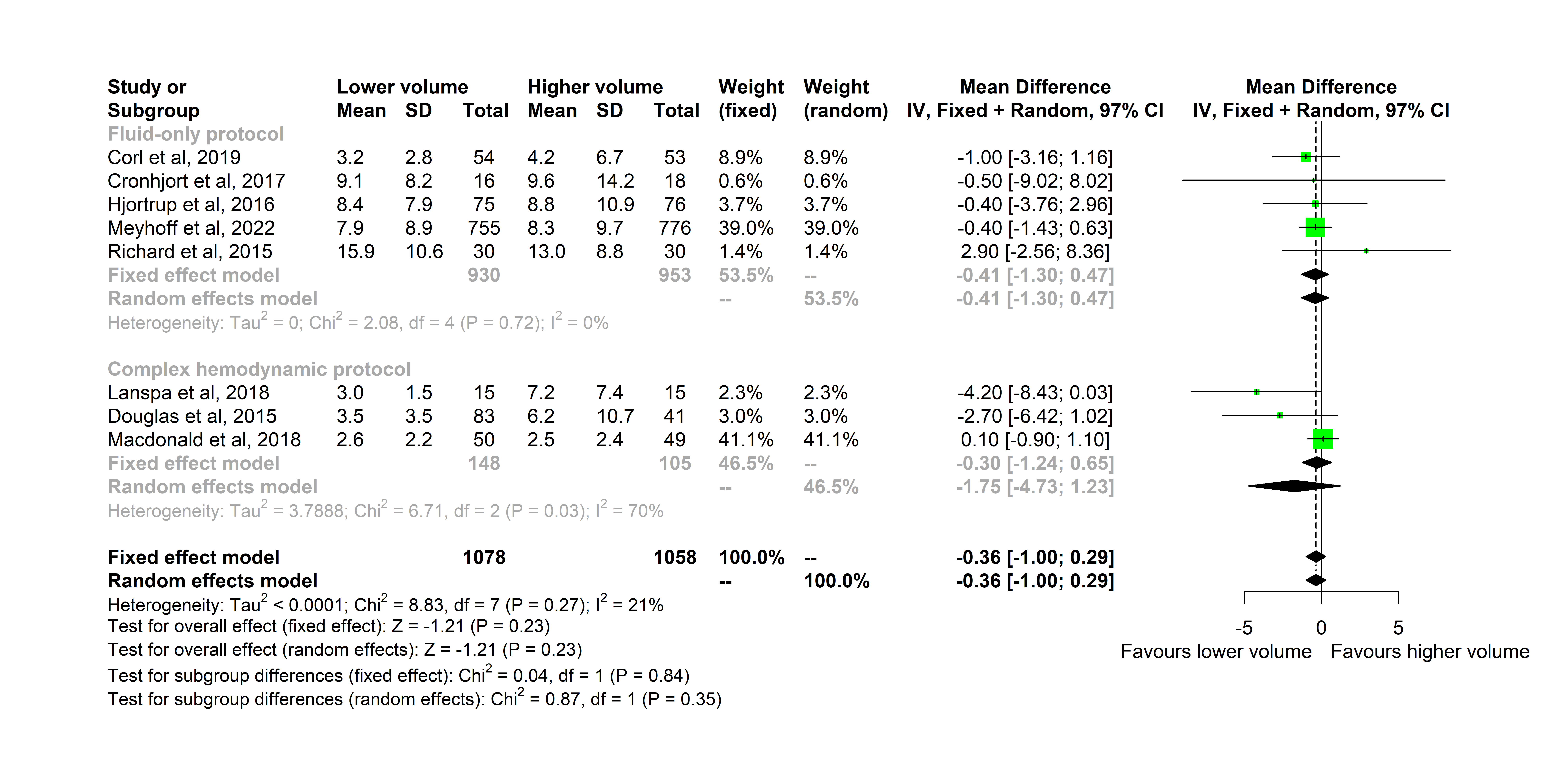


### ICU length of stay: Early vs later resuscitation phase of sepsis


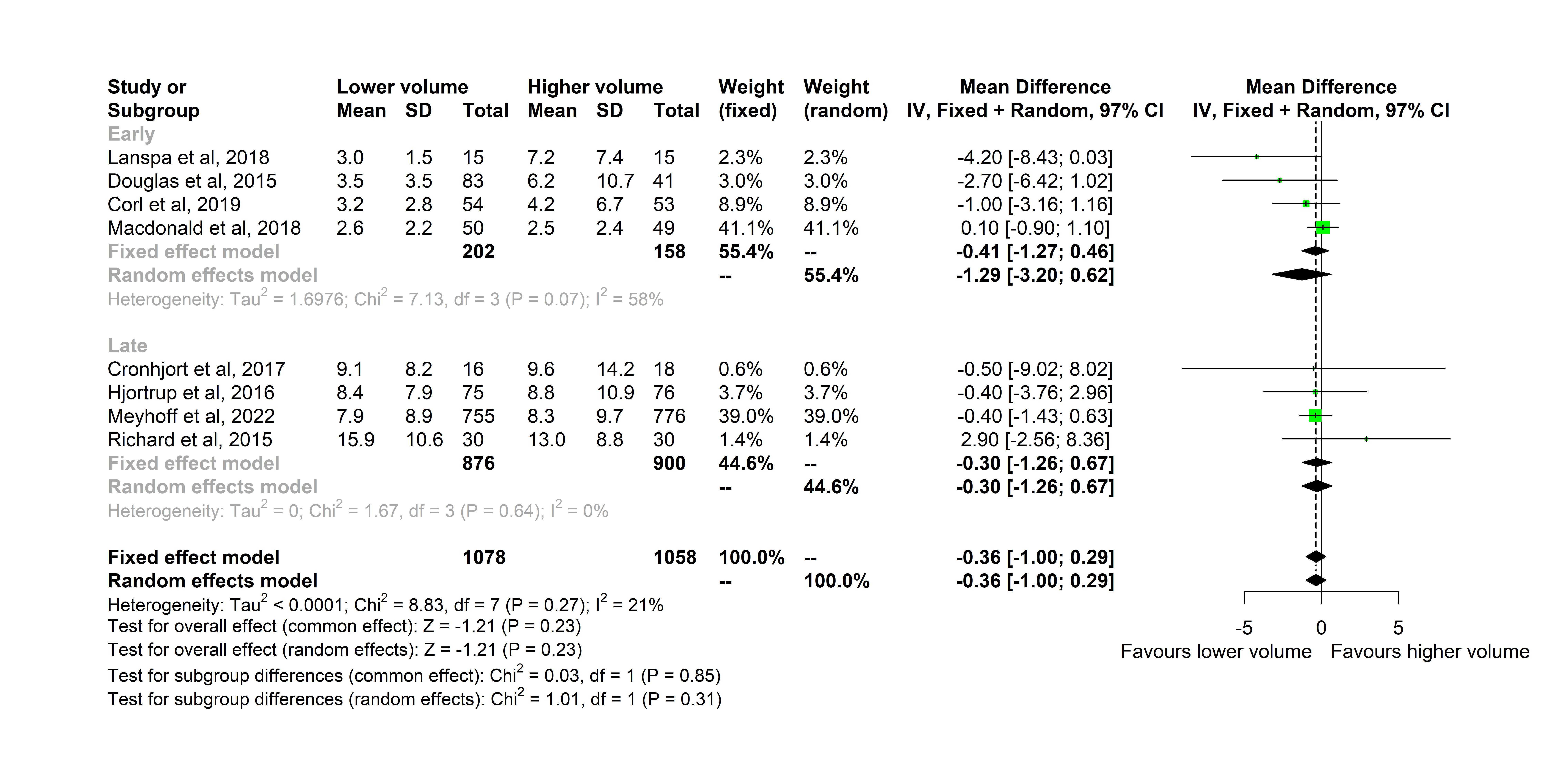


### 9.2.3 c) Subgroup analyses of hospital length of stay

### Hospital length of stay: Overall low vs some concern or high risk of bias

### Hospital length of stay: Patients with sepsis vs septic shock

### Hospital length of stay: Fluid-only interventions vs a complex hemodynamic protocol

### Hospital length of stay: Early vs later resuscitation phase of sepsis

# 10. e-Table 5 Fluid Data in the Included Trials

| **Author** | **Volume of resuscitation fluid/ml** | **Total fluid input/ml** | **Use of diuretics/ mg** | **Fluid removal by RRT/ml** | **Fluid balance over study period/ml** |
| --- | --- | --- | --- | --- | --- |
| Chen and Kollef^4^  *Low  High | Day 5  6244 (5106-8497)  8690 (4211-13197) | Day 5  9164  11350 | Used in both groups, encouraged as part of the intervention in lower fluid volume group | *NR*  *NR* | Day 5  2641 (1837 - 5075)  3616 (-1513 - 9746) |
| Cronhjort et al.^5^  Low  High | Day 3 or ICU discharge  2103 (1283-2645)  2408 (954-5045) | Day 3 or ICU discharge  10646 (7851-12092)  10526 (6158-12902) | *NR*  *NR* | *NR*  *NR* | Day 3 or ICU discharge  1566 ± 3725^a^  2669 ± 2675 |
| Hjortrup et al.^6^  Low  High | During ICU stay  500 (0-3250)  2200 (1000-4750) | During ICU stay  18291 (5518-17035)  16970 (7163-29889) | *NR*  *NR* | *NR*  *NR* | During ICU stay  1923 (-1964 – 5415)  2014 (-168 – 4678) |
| Richard et al. ^7^  Low  High | Daily  446 (295-1105)  986 (654-1624) | Daily  3610 (2982-4560)  4096 (3770-4677) | In case of fluid overload but not recommended first 48h of study | *NR*  *NR* | Daily  888 (153-2816)  1749 (146-2788) |
| Van Genderen et al.^8^  Low  High | *NR*  *NR* | 72h  7565 (982)^b^  10028 (941) | *NR*  *NR* | *NR*  *NR* | *NR*  *NR* |
| Macdonald et al.^9^  Low  High | 6h  550 (0-1150)  1535 (1000-2200) | 6h  968 (625-1458)  1715 (1017-2500) | *NR*  *NR* | *NR*  *NR* | *NR*  *NR* |
| Lanspa et al.^10^  Low  High | 6h study period  0 (0-2000)  1000 (0-2000) | 24h  6000 (4700-8500)  6400 (4700-9600) | *NR*  *NR* | *NR*  *NR* | *NR*  *NR* |
| Semler et al.^11^  Low  High | Day 14  300 ± 560 ^a^  733 ± 1083 | Day 14  8450 ± 10103 ^a^  7049 ± 6459 | Day 14  Furosemide  133 ± 361 ^a^  33 ± 68 | *NR*  *NR* | Day 14  -2195 ± 4313^a^  764 ± 4352 |
| Corl et al.^12^  Low  High | Prior to randomisation + 72h study period  4140 ±1660 ^a^  4963 ± 2362 | 72h study period  6213 ± 2207 ^a^  8027± 4120 ^a^ | *NR*  *NR* | *NR*  *NR* | *NR*  *NR* |
| Douglas et al.^13^  Low  High | 72 h post enrollment  3567 (1795-5350)  3231 (1787-6990) | NR  NR | Mean differences reported in time interval, however, not significant within first 72 h | 72 h post enrollment*  165  287 | 72 h post enrollment (+pre-enrollment fluids)  3418 (1219-6000)  3398 (1670-5781) |
| Jessen et al.^14^  Low  High | 24h  0 (0-600)  1000 (80-2000) | 24h  792 (400-1400)  1625 (1200-2650) | NR  NR | *7 days*  *0*  *0* | *NR*  *NR* |
| Meyhoff et al.^15^  Low  High | During ICU stay  1798 (500-4366)  3811 (1861-6762) | During ICU stay  10,433 (5024 – 25567)  12,747 (6453 – 28100) | NR  NR | During ICU stay  *2229* ± 8084^a,d^  *2233* ± 7208 | During ICU stay  *1645 (-461 to 4423)*  *2368 (368 to 5517)* |
| Shapiro et al.^16^  Low  High | 24h  500 (0-1500)  2750 (2000-3615) | 24h  1267 (555 – 2279)  3400 (2500 - 4495) | NR  NR | NR  NR | NR  NR |

Data reported as medians (IQR) unless otherwise stated. All study fluids are reported at longest time period.

*Low: Lower fluid volume, High: Higher fluid volume, NR: Not Reported, RRT: renal replacement therapy, ICU: intensive care unit, mg: miligram, ml: mililiter, kg: kilogram, h: hours

^a^ Data reported are mean ±standard deviation

^b^Data reported are mean (standard error)

^c^Not reported, but 72-hour fluid balance with and without dialysis volumes available, thus, we calculated and reported the difference

# 11. e-Table 6 Overview of Risk of Bias (RoB2) adjudications for all outcomes

**Table 2Sa Information on available source data**

| Trials | Original article | Supplements | Protocol/ Statistical analysis plan | Online registries |
| --- | --- | --- | --- | --- |
| Van Genderen et al, 2015 | X | x |  | x |
| Chen and Kollef, 2015 | X |  |  | x |
| Richard et al, 2015 | X | x |  | x |
| Lanspa et al, 2018 | X | x |  | x |
| Hjortrup et al, 2016 | X | x | x | x |
| Cronhjort et al, 2017 | X |  |  | x |
| Macdonald et al, 2018 | X | x | x | X |
| Corl et al, 2019 | X | x |  | x |
| Semler et al, 2019 | X | x |  | X |
| Jessen et al, 2022 | X | x | x | x |
| Meyhoff et al, 2022 | x | x | x | x |
| Shapiro et al, 2023 | x | x | x | x |

## e-Table 6.1 RoB2 adjudications for all-cause mortality

|  | **Risk of bias domain (assessment for the effect of assignment to intervention)** | | | | | **Overall**  **risk of bias** |
| --- | --- | --- | --- | --- | --- | --- |
| **Outcome and study** | 1. Randomisation process | 2. Deviations from intended interventions | 3. Missing outcome data | 4. Measurement of the outcome | 5. Selection of the reported result |  |
| **All-cause mortality** | | | | | | |
| Van Genderen et al, 2015 | Low | Some concerns | High | Low | Some concerns | High |
|  | Deviations from intended interventions: Protocol deviations not reported.  Missing outcome data: No information on missing outcome data.  Measurement of the outcome: Unclear whether outcome assessors were blinded.  Selection of the reported result: No published protocol but registered at clinicaltrials.gov prior to inclusion. However, mortality was not a pre-planned outcome. | | | | | |
| Chen and Kollef, 2015 | Some concerns | Low | Low | Low | Some concerns | Some concerns |
|  | Randomisation process: Insufficient information to permit judgement about allocation concealment.  Selection of the reported result: No published protocol but registered at clinicaltrials.gov after completion of the trial. However, unclear whether this was prior to unblinding. | | | | | |
| Richard et al, 2015 | Low | Low | Low | Low | Some concerns | Some concerns |
|  | Selection of the reported result: No published protocol but registered at clinicaltrials.gov after completion of the trial. However, unclear whether this was prior to unblinding. | | | | | |
| Lanspa et al, 2018 | Low | Low | Low | Low | Some concerns | Some concerns |
|  | Selection of the reported result: No published protocol, but registered at clinicaltrials.gov after initiation of the trial, but before data analysis. Mortality was reported up to day 28 but was pre-planned up to day 7. | | | | | |
| Douglas et al, 2020 | Low | Some concerns | Low | Low | Low | Some concerns |
|  | Deviations from intended interventions: No information on protocol deviations. | | | | | |
| Hjortrup et al, 2016 | Low | Low | Low | Low | Low | Low |
| Cronhjort et al, 2017 | Low | Low | Low | Low | Low | Low |
| Macdonald et al, 2018 | Low | Low | Low | Low | Low | Low |
| Corl et al, 2019 | Low | Low | Low | Low | Low | Low |
| Semler et al, 2019 | Low | Low | Low | Low | Low | Low |
| Jessen et al, 2022 | Low | Low | Low | Low | Low | Low |
| Meyhoff et al, 2022 | Low | Low | Low | Low | Low | Low |
| Shapiro et al, 2023 | Low | Low | Low | Low | Low | Low |

## e-Table 6.2 RoB2 adjudications for serious adverse events

|  | **Risk of bias domain (assessment for the effect of assignment to intervention)** | | | | | **Overall**  **risk of bias** |
| --- | --- | --- | --- | --- | --- | --- |
| **Outcome and study** | 1. Randomisation process | 2. Deviations from intended interventions | 3. Missing outcome data | 4. Measurement of the outcome | 5. Selection of the reported result |  |
| **Serious adverse events** | | | | | | |
| Hjortrup et al, 2016 | Low | Low | Low | Some concerns | Low | Some concerns |
|  | Measurement of the outcome: Outcome assessors were aware of the randomisation group, which could have influenced the outcome assessment. | | | | | |
| Macdonald et al, 2018 | Low | Low | Low | Low | Some concerns | Some concerns |
|  | Selection of the reported result: SAEs and AEs defined in the protocol, but not reported accordingly | | | | | |
| Douglas et al, 2020 | Low | Some concerns | Low | Some concerns | Low | Some concerns |
|  | Deviations from intended interventions: No information on protocol deviations.  Measurement of the outcome: Outcome assessors were aware of the randomisation group, which could have influenced the outcome assessment. | | | | | |
| Jessen et al, 2022 | Low | Low | Low | Some concerns | Low | Some concerns |
|  | Measurement of the outcome: Outcome assessors were aware of the randomisation group, which could have influenced the outcome assessment. | | | | | |
| Meyhoff et al, 2022 | Low | Low | Low | Some concerns | Low | Some concerns |
|  | Measurement of the outcome: Outcome assessors were aware of the randomisation group, which could have influenced the outcome assessment. | | | | | |
| Shapiro et al, 2023 | Low | Low | Low | Some concerns | Low | Some concerns |
|  | Measurement of the outcome: Outcome assessors were aware of the randomisation group, which could have influenced the outcome assessment. | | | | | |

## e-Table 6.3 RoB2 adjudications for duration of mechanical ventilation

|  | **Risk of bias domain (assessment for the effect of assignment to intervention)** | | | | | | **Overall**  **risk of bias** |
| --- | --- | --- | --- | --- | --- | --- | --- |
| **Outcome and study** | 1. Randomisation process | 2. Deviations from intended interventions | | 3. Missing outcome data | 4. Measurement of the outcome | 5. Selection of the reported result |  |
| **Duration of mechanical ventilation (MV)** | | | | | | | |
| Corl et al, 2019 | Low | Low | | Low | Low | Some concerns | Some concerns |
|  | Selection of the reported result: No published protocol, but registered at clinicaltrials.gov after initiation of the trial, but before data analysis. Duration of mechanical ventilation was reported up to day 30 but was pre-planned up to day 60. | | | | | | |
| Douglas et al, 2020 | Low | | Some concerns | Low | Low | Low | Some concerns |
|  | Deviations from intended interventions: No information on protocol deviations. | | | | | | |
| Hjortrup et al, 2016 | Low | | Low | Low | Low | Low | Low |
| Macdonald et al, 2018 | Low | | Low | Low | Low | Low | Low |
| Meyhoff et al, 2022 | Low | | Low | Low | Low | Low | Low |
| Shapiro et al, 2023 | Low | | Low | Low | Low | Low | Low |

## e-Table 6.4 RoB2 adjudications for ventilator-free days

|  | **Risk of bias domain (assessment for the effect of assignment to intervention)** | | | | | | **Overall**  **risk of bias** |
| --- | --- | --- | --- | --- | --- | --- | --- |
| **Outcome and study** | 1. Randomisation process | 2. Deviations from intended interventions | | 3. Missing outcome data | 4. Measurement of the outcome | 5. Selection of the reported result |  |
| **Ventilator-free days** | | | | | | | |
| Richard et al, 2015 | Low | | Low | Low | Low | Some concerns | Some concerns |
|  | Selection of the reported result: No published protocol but registered at clinicaltrials.gov after completion of the trial. However, unclear whether this was prior to unblinding. | | | | | | |
| Lanspa et al, 2018 | Low | | Low | Low | Low | Some concerns | Some concerns |
|  | Selection of the reported result: No published protocol, but registered at clinicaltrials.gov after initiation of the trial, but before data analysis. Ventilator-free days was reported up to day 28 but was pre-planned up to day 7. | | | | | | |
| Corl et al, 2019 | Low | | Low | Low | Low | Some concerns | Some concerns |
|  | Selection of the reported result: No published protocol but registered at clinicaltrials.gov after initiation of the trial. However, ventilator-free days was not a pre-planned outcome. | | | | | | |
| Hjortrup et al, 2016 | Low | | Low | Low | Low | Low | Low |
| Macdonald et al, 2018 | Low | | Low | Low | Low | Low | Low |
| Semler et al, 2019 | Low | | Low | Low | Low | Low | Low |
| Meyhoff et al, 2022 | Low | | Low | Low | Low | Low | Low |
| Shapiro et al, 2023 | Low | | Low | Low | Low | Low | Low |

## e-Table 6.5 RoB2 adjudications for duration of vasopressor or inotropes

|  | **Risk of bias domain (assessment for the effect of assignment to intervention)** | | | | | | **Overall**  **risk of bias** |
| --- | --- | --- | --- | --- | --- | --- | --- |
| **Outcome and study** | 1. Randomisation process | 2. Deviations from intended interventions | | 3. Missing outcome data | 4. Measurement of the outcome | 5. Selection of the reported result |  |
| **Duration of vasopressor or inotropes, hours** | | | | | | | |
| Richard et al, 2015 | Low | | Low | Low | Low | Some concerns | Some concerns |
|  | Selection of the reported result: No published protocol but registered at clinicaltrials.gov after completion of the trial. However, unclear whether this was prior to unblinding. Duration of vasopressor or inotropes was not a pre-planned outcome. | | | | | | |
| Corl et al, 2019 | Low | | Low | Low | Low | Some concerns | Some concerns |
|  | Selection of the reported result: No published protocol but registered at clinicaltrials.gov after initiation of the trial. However, duration of vasopressor or inotropes was reported up to day 30 but was pre-planned up to day 60. | | | | | | |
| Douglas et al, 2020 | Low | | Some concerns | Low | Low | Low | Some concerns |
|  | Deviations from intended interventions: No information on protocol deviations. | | | | | | |
| Macdonald et al, 2018 | Low | | Low | Low | Low | Low | Low |
| Meyhoff et al, 2022 | Low | | Low | Low | Low | Low | Low |
| Shapiro et al, 2023 | Low | | Low | Low | Low | Low | Low |

## e-Table 6.6 RoB2 adjudications for vasopressor-free days

|  | **Risk of bias domain (assessment for the effect of assignment to intervention)** | | | | | | **Overall**  **risk of bias** |
| --- | --- | --- | --- | --- | --- | --- | --- |
| **Outcome and study** | 1. Randomisation process | 2. Deviations from intended interventions | | 3. Missing outcome data | 4. Measurement of the outcome | 5. Selection of the reported result |  |
| **Vasopressor-free days** | | | | | | | |
| Corl et al, 2019 | Low | | Low | Low | Low | Some concerns | Some concerns |
|  | Selection of the reported result: No published protocol but registered at clinicaltrials.gov after initiation of the trial. However, vasopressor-free days was not a pre-planned outcome. | | | | | | |
| Semler et al, 2019 | Low | | Low | Low | Low | Some concerns | Some concerns |
|  | Selection of the reported result: No published protocol but registered at clinicaltrials.gov before initiation of the trial. However, vasopressor-free days was not a pre-planned outcome. | | | | | | |
| Macdonald et al, 2018 | Low | | Low | Low | Low | Low | Low |
| Meyhoff et al, 2022 | Low | | Low | Low | Low | Low | Low |
| Shapiro et al, 2023 | Low | | Low | Low | Low | Low | Low |

## e-Table 6.7 RoB2 adjudications for use of renal replacement therapy (RRT)

|  | **Risk of bias domain (assessment for the effect of assignment to intervention)** | | | | | **Overall**  **risk of bias** |
| --- | --- | --- | --- | --- | --- | --- |
| **Outcome and study** | 1. Randomisation process | 2. Deviations from intended interventions | 3. Missing outcome data | 4. Measurement of the outcome | 5. Selection of the reported result |  |
| **Use of RRT** | | | | | | |
| Chen and Kollef 2015 | Some concerns | Low | Low | Low | Some concerns | Some concerns |
|  | Randomisation process: Insufficient information to permit judgement about allocation concealment.  Selection of the reported result: No published protocol but registered at clinicaltrials.gov after completion of the trial. However, unclear whether this was prior to unblinding. Use of RRT was not a pre-planned outcome. | | | | | |
| Corl et al, 2019 | Low | Low | Low | Low | Some concerns | Some concerns |
|  | Selection of the reported result: No published protocol but registered at clinicaltrials.gov after initiation of the trial. The preplanned time frame was up to 60 days, however it is unclear if it is reported up to day 30 or 60. | | | | | |
| Douglas et al, 2020 | Low | Some concerns | Low | Low | Low | Some concerns |
|  | Deviations from intended interventions: No information on protocol deviations. | | | | | |
| Hjortrup et al, 2016 | Low | Low | Low | Low | Low | Low |
| Macdonald et al, 2018 | Low | Low | Low | Low | Low | Low |
| Semler et al, 2019 | Low | Low | Low | Low | Low | Low |
| Jessen et al, 2022 | Low | Low | Low | Low | Low | Low |
| Meyhoff et al, 2022 | Low | Low | Low | Low | Low | Low |
| Shapiro et al, 2023 | Low | Low | Low | Low | Low | Low |

## e-Table 6.8 RoB2 adjudications for duration of RRT

|  | **Risk of bias domain (assessment for the effect of assignment to intervention)** | | | | | **Overall**  **risk of bias** |
| --- | --- | --- | --- | --- | --- | --- |
| **Outcome and study** | 1. Randomisation process | 2. Deviations from intended interventions | 3. Missing outcome data | 4. Measurement of the outcome | 5. Selection of the reported result |  |
| **Duration of RRT** | | | | | | |
| Macdonald et al, 2018 | Low | Low | Low | Low | Low | Low |
| Jessen et al, 2022 | Low | Low | Low | Low | Low | Low |
| Meyhoff et al, 2022 | Low | Low | Low | Low | Low | Low |

## e-Table 6.9 RoB2 adjudications for renal replacement therapy-free days

|  | **Risk of bias domain (assessment for the effect of assignment to intervention)** | | | | | **Overall**  **risk of bias** |
| --- | --- | --- | --- | --- | --- | --- |
| **Outcome and study** | 1. Randomisation process | 2. Deviations from intended interventions | 3. Missing outcome data | 4. Measurement of the outcome | 5. Selection of the reported result |  |
| **RRT-free days** | | | | | | |
| Hjortrup et al, 2016 | Low | Low | Low | Low | Low | Low |
| Macdonald et al, 2018 | Low | Low | Low | Low | Low | Low |
| Semler et al, 2019 | Low | Low | Low | Low | Low | Low |
| Meyhoff et al, 2022 | Low | Low | Low | Low | Low | Low |
| Shapiro et al, 2023 | Low | Low | Low | Low | Low | Low |

## e-Table 6.10 RoB2 adjudications for incidence of acute kidney injury

|  | **Risk of bias domain (assessment for the effect of assignment to intervention)** | | | | | **Overall**  **risk of bias** |
| --- | --- | --- | --- | --- | --- | --- |
| **Outcome and study** | 1. Randomisation process | 2. Deviations from intended interventions | 3. Missing outcome data | 4. Measurement of the outcome | 5. Selection of the reported result |  |
| **Incidence of acute kidney injury** | | | | | | |
| Corl et al, 2019 | Low | Low | Low | Low | Some concerns | Some concerns |
|  | Selection of the reported result: No published protocol but registered at clinicaltrials.gov after initiation of the trial. However, incidence of AKI was not a pre-planned outcome. | | | | | |
| Jessen et al, 2022 | Low | Low | Low | Low | Low | Low |
| Meyhoff et al, 2022 | Low | Low | Low | Low | Low | Low |

## e-Table 6.11 RoB2 adjudications for blood products

|  | **Risk of bias domain (assessment for the effect of assignment to intervention)** | | | | | | **Overall**  **risk of bias** |
| --- | --- | --- | --- | --- | --- | --- | --- |
| **Outcome and study** | 1. Randomisation process | 2. Deviations from intended interventions | | 3. Missing outcome data | 4. Measurement of the outcome | 5. Selection of the reported result |  |
| **Use of blood products** | | | | | | | |
| Richard et al, 2015 | Low | | Low | Low | Low | Some concerns | Some concerns |
|  | Selection of the reported result: No published protocol but registered at clinicaltrials.gov after completion of the trial. However, unclear whether this was prior to unblinding. The use of blood products was not a pre-planned outcome. | | | | | | |
| Semler et al, 2019 | Low | | Low | Low | Low | Some concerns | Some concerns |
|  | Selection of the reported result: No published protocol but registered at clinicaltrials.gov before initiation of the trial. However, the use of blood products was not a pre-planned outcome. | | | | | | |
| Jessen et al, 2022 | Low | | Low | Low | Low | Low | Low |
| Hjortrup et al, 2016 | Low | | Low | Low | Low | Low | Low |
| Meyhoff et al, 2022 | Low | | Low | Low | Low | Low | Low |

## e-Table 6.12 RoB2 adjudications for length of ICU stay

|  | **Risk of bias domain (assessment for the effect of assignment to intervention)** | | | | | | **Overall**  **risk of bias** |
| --- | --- | --- | --- | --- | --- | --- | --- |
| **Outcome and study** | 1. Randomisation process | 2. Deviations from intended interventions | | 3. Missing outcome data | 4. Measurement of the outcome | 5. Selection of the reported result |  |
| **ICU length of stay** | | | | | | | |
| Richard et al, 2015 | Low | | Low | Low | Low | Some concerns | Some concerns |
|  | Selection of the reported result: No published protocol but registered at clinicaltrials.gov after completion of the trial. However, unclear whether this was prior to unblinding. ICU length of stay was not a pre-planned outcome. | | | | | | |
| Douglas et al, 2020 | Low | | Some concerns | Low | Low | Low | Some concerns |
|  | Deviations from intended interventions: No information on protocol deviations. | | | | | | |
| Hjortrup et al, 2016 | Low | | Low | Low | Low | Low | Low |
| Cronhjort et al, 2017 | Low | | Low | Low | Low | Low | Low |
| Macdonald et al, 2018 | Low | | Low | Low | Low | Low | Low |
| Lanspa et al, 2018 | Low | | Low | Low | Low | Low | Low |
| Corl et al, 2019 | Low | | Low | Low | Low | Low | Low |
| Meyhoff et al, 2022 | Low | | Low | Low | Low | Low | Low |

## e-Table 6.13 RoB2 adjudications for length of hospital stay

|  | **Risk of bias domain (assessment for the effect of assignment to intervention)** | | | | | | **Overall**  **risk of bias** |
| --- | --- | --- | --- | --- | --- | --- | --- |
| **Outcome and study** | 1. Randomisation process | 2. Deviations from intended interventions | | 3. Missing outcome data | 4. Measurement of the outcome | 5. Selection of the reported result |  |
| **Length of hospital stay** | | | | | | | |
| Corl et al, 2019 | Low | | Low | Low | Low | Some concerns | Some concerns |
|  | Selection of the reported result: No published protocol but registered at clinicaltrials.gov after initiation of the trial. However, hospital length of stay was not a pre-planned outcome | | | | | | |
| Douglas et al, 2020 | Low | | Some concerns | Low | Low | Some concerns | Some concerns |
|  | Deviations from intended interventions: No information on protocol deviations.  Selection of the reported result: Hospital length of stay was not a pre-planned outcome. | | | | | | |
| Macdonald et al, 2018 | Low | | Low | Low | Low | Low | Low |
| Jessen et al, 2022 | Low | | Low | Low | Low | Low | Low |
| Meyhoff et al, 2022 | Low | | Low | Low | Low | Low | Low |

# **12.** **e-Table 7 Overview of all primary meta-analyses**

|  | Random effects model | Fixed effect model | Heterogeneity |
| --- | --- | --- | --- |
| **Primary outcomes^a^  RR with 97%CI RR with 97%CI** | | | |
| All-cause mortality  *Low RoB trials only*^5,6,9,11,12,14–16^  *All trials*^4–16^ | 0.99 (0.90, 1.10) P=0.89  0.98 (0.89, 1.08) P=0.68 | 0.99 (0.89, 1.10) P=0.89  0.98 (0.89, 1.08) P=0.69 | I^2^=0% Tau^2^=0  I^2^=0% Tau^2^=0 |
| Serious Adverse Events  *Only if defined in the trial* ^6,9,13–16^  *SAE incl. mortality*^4–16^  *SAE cumulated*^4–16^ | 0.93 (0.85, 1.02) P=0.08  0.96 (0.89, 1.03) P=0.22  1.00 (0.97, 1.02) P=0.80 | 0.95 (0.83, 1.07) P=0.34  0.99 (0.90, 1.08) P=0.73  0.99 (0.93, 1.05) P=0.67 | I^2^=0% Tau^2^=0  I^2^=0% Tau^2^=0  I^2^=0% Tau^2^=0 |
| HRQoL | NR | NR | NR |
| **Secondary outcomes^a^ MD or RR with 99%CI MD or RR with 99%CI** | | |  |
| Duration of MV (days)  *Low RoB trials only*^6,9,15,16^  *All trials*^6,9,12,13,15,16^ | -0.11 (-0.50, 0.28) P=0.46  -0.73 (-1.97, 0.51) P=0.13 | -0.11 (-0.50, 0.28) P=0.46  -0.21 (-0.59, 0.17) P=0.16 | I^2^=0% Tau^2^=0  I^2^=61%, Tau^2^=0.86 |
| Ventilator-free days  *Low RoB trials only*^6,9,11,15,16^  *All trials*^6,7,9–12,15,16^ | -0.11 (-1.89, 1.67) P=0.87  0.15 (-1.31, 1.61) P=0.79 | -0.21 (-0.97, 1.38) P=0.65  -0.28 (-0.86, 1.43) P=0.53 | I^2^=0% Tau^2^=0.56  I^2^=0% Tau^2^=0.25 |
| Duration of vasopressor or inotropes (hours)  *Low RoB trials only*^9,15,16^  *All trials*^7,9,12,13,15,16^ | 0.00 (-0.33, 0.33) P=1.00  0.86 (-4.33, 6.05) P=0.52 | 0.00 (-0.33, 0.33) P=1.00  0.00 (-0.33, 0.34) P=0.13 | I^2^=0% Tau^2^=0  I^2^=26% Tau^2^=5.37 |
| Vasopressor-free days  *Low RoB trials only*^9,15,16^  *All trials*^9,11,12,15,16^ | 0.43 (-0.68, 1.53) P=0.32  0.45 (-0.62, 1.51) P=0.28 | 0.43 (-0.68, 1.53) P=0.32  0.45 (-0.62, 1.51) P=0.28 | I^2^=0% Tau^2^=0  I^2^=0% Tau^2^=0 |
| Use of RRT  *Low RoB trials only*^6,9,11,15,16^  *All trials*^4,6,9,11–16^ | 1.01 (0.80, 1.26) P=0.95  0.97 (0.78, 1.20) P=0.73 | 1.01 (0.80, 1.26) P=0.95  0.97 (0.78, 1.20) P=0.70 | I^2^=0% Tau^2^=0  I^2^=0% Tau^2^=0 |
| Duration of RRT (days)^b^  *All trials (all low RoB)* ^9,14,15^ | -0.17 (-0.70. 0.37) P=0.43 | -0.17 (-0.70. 0.37) P=0.43 | I^2^=0% Tau^2^=0 |
| RRT free days  *All trials (all low RoB)*^6,9,11,15,16^ | 0.51 (-0.75,1.78) P=0.30 | 0.46 (-0.68, 1.61) P=0.30 | I^2^=0% Tau^2^=0.07 |
| Incidence of AKI  *Low RoB trials only*^14,15^  *All trials*^12,14,15^ | 0.94 (0.75, 1.19) P=0.50  0.94 (0.75, 1.19) P=0.50 | 0.94 (0.75, 1.19) P=0.50  0.94 (0.75, 1.19) P=0.50 | I^2^=0% Tau^2^=0  I^2^=0% Tau^2^=0 |
| **Exploratory outcomes^a^ MD with 97%CI MD with 97%CI** | | |  |
| Use of blood products (units)  *Low RoB trials only*^6,14,15^  *All trials*^6,7,11,15^ | 0.00 (-0.10, 0.10) P=1.00  0.00 (-0.10, 0.10) P=0.98 | 0.00 (-0.10, 0.10) P=1.00  0.00 (-0.10, 0.10) P=0.98 | I^2^=0% Tau^2^=0  I^2^=0% Tau^2^=0 |
| ICU length of stay (days)  *Low RoB trials only*^5,6,9,10,12,15^  *All trials*^6,10–13,15^ | -0.33 (-0.99, 0.33) P=0.27  -0.36 (-1.00, 0.29) P=0.23 | -0.33 (-0.99, 0.33) P=0.27  0.36 (-1.00, 0.29) P=0.23 | I^2^=5% Tau^2^=0  I^2^=21%, Tau^2^=0 |
| Hospital length of stay (days)  *Low RoB trials only*^13–15^  *All trials*^9,12–15^ | 0.78 (-0.68, 2.24) P=0.25  0.47 (-0.80, 1.75) P=0.42 | 0.78 (-0.68, 2.24) P=0.25  0.47 (-0.80, 1.75) P=0.42 | I^2^=0% Tau^2^=0  I^2^=0% Tau^2^=0 |

RoB: Risk of Bias, CI: confidence interval, RR: relative risk, MD: mean difference

^a^ Confidence intervals corresponding to predefined alpha, thus 97% CI for primary and exploratory outcomes and 99% CI for secondary outcomes

^b^ One zero-event trial ignored^14^

# 13. e-Table 8 Overview of all subgroup analyses

| Outcome measure | Subgroups | N | MD/RR (97% or 99% CI) for lower vs higher volumes  Random effects model/Fixed effect model | | Test of interaction  (P-value) |  |  |  |  |
| --- | --- | --- | --- | --- | --- | --- | --- | --- | --- |
| PRIMARY OUTCOMES | | | | | |  |  |  |  |
| All-cause mortality | | | | |  |  |  |  |  |
| 8 trials^5,6,9,11,12,14–16^ | Low risk | 3626 | 0.99 (0.89, 1.10) | 0.99 (0.90, 1.10) | REM P=0.41 |  |  |  |  |
| 5 trials^4,7,8,10,13^ | Some concerns/high risk | 352 | 0.87 (0.63, 1.20) | 0.87 (0.63, 1.20) | FEM P=0.40 |  |  |  |  |
| 8 trials^6,7,9,12–16^ | Successful protocol | 3772 | 0.98 (0.89, 1.09) | 0.98 (0.89, 1.09)  0.98 (0.67, 1.43) | REM P=0.88 |  |  |  |  |
| 5 trials^4,5,8,10,11^ | Unsuccessful protocol | 206 | 0.96 (0.66, 1.39) |  | FEM P=0.99 |  |  |  |  |
| 3 trials^9,14,16^ | Sepsis | 1757 | 1.01 (0.83, 1.23) | 1.01 (0.83, 1.23)  0.97 (0.86, 1.09) | REM P=0.70 |  |  |  |  |
| 7 trials^4–8,10,15^ | Septic shock | 1932 | 0.97 (0.85, 1.10) |  | FEM P=0.72 |  |  |  |  |
| 8 trials^4–8,12,14,15^ | Fluid-only protocol | 2132 | 0.95 (0.83, 1.09) | 0.96 (0.86, 1.08)  1.03 (0.85, 1.25) | REM P=0.48 |  |  |  |  |
| 5 trials^9–11,13,16^ | Complex hemodynamic protocol | 1846 | 1.03 (0.85, 1.25) |  | FEM P=0.52 |  |  |  |  |
| 7 trials^8–10,12–14,16^ | Early | 2076 | 1.02 (0.85, 1.22) | 1.02 (0.85, 1.22)  0.96 (0.85, 1.08) | REM P=0.38 |  |  |  |  |
| 6 trials^4–7,11,15^ | Late | 1902 | 0.91 (0.75, 1.11) |  | FEM P=0.58 |  |  |  |  |
| 3 trials^9,14,15^ | Sepsis-3 definition | 1761 | 1.00 (0.88, 1.13) | 1.00 (0.88, 1.13) | REM P=0.64 |  |  |  |  |
| 10 trials^4-8,10–13,16^ | Other sepsis definitions | 2217 | 0.96 (0.82, 1.12) | 0.96 (0.82, 1.13) | FEM P=0.71 |  |  |  |  |
| Serious adverse events | | | | |  |  |  |  | Serious adverse events (model: FEM) |
| 3 trials^9,14,16^ | Sepsis | 1785 | 1.02 (0.66, 1.58) | 1.03 (0.66, 1.60)  0.95 (0.83, 1.08) | REM P=0.65 |  |  |  |  |
| 2 trials^6,15^ | Septic shock | 1674 | 0.93 (0.84, 1.02) |  | FEM P=0.69 |  |  |  |  |
| 3 trials^6,14,15^ | Fluid-only protocol | 1797 | 0.93 (0.84, 1.02) | 0.95 (0.83, 1.08)  0.96 (0.57, 1.61) | REM P=0.95 |  |  |  |  |
| 3 trials^9,13,16^ | Complex hemodynamic protocol | 1805 | 0.94 (0.56, 1.59) |  | FEM P=0.96 |  |  |  |  |
| 4 trials^9,13,14,16^ | Early | 1928 | 0.95 (0.64, 1.42) | 0.96 (0.64, 1.43)  0.95 (0.83, 1.08) | REM P=0.90 |  |  |  |  |
| 2 trials^6,15^ | Late | 1674 | 0.93 (0.84, 1.02) |  | FEM P=0.95 |  |  |  |  |
| SECONDARY OUTCOMES | | | | | |  |  |  |  |
| Duration of mechanical ventilation (days) | | | | | |  |  |  |  |
| 4 trials^6,9,15,16^ | Low risk | 3325 | -0.11 (-0.50, 0.28) | -0.11 (-0.50, 0.28) | REM P<0.01 |  |  |  |  |
| 2 trials^12,13^ | Some concerns/high risk | 156 | -3.00 (-5.07,-0.93) | -3.00 (-5.07,-0.93) | FEM P<0.01 |  |  |  |  |
| 2 trial^9,16^ | Sepsis | 1643 | 0.08 (-0.52, 0.35) | 0.08 (-0.52, 0.35) | REM P=0.68  FEM P=0.68 |  |  |  |  |
| 2 trials^6,15^ | Septic shock | 1682 | -0.24 (-1.13, 0.65) | -0.24 (-1.13, 0.65) |  |  |  |  |  |
| 3 trials^6,12,15^ | Fluid-only protocol | 1714 | -0.57 (-2.07,0.92) | -0.34 (-1.22, 0.53) | REM P=0.79  FEM P=0.66 |  |  |  |  |
| 3 trials^9,13,16^ | Complex hemodynamic protocol | 1767 | -0.85 (-3.11, 1.41) | -0.18 (-0.61, 0.25) |  |  |  |  |  |
| 4 trials^9,12,13,16^ | Early | 1799 | -1.14 (-3.25, 0.97) | -0.20 (-0.63, 0.22) | REM P=0.31  FEM P=0.93 |  |  |  |  |
| 2 trials^6,15^ | Late | 1682 | -0.24 (-1.13, 0.65) | -0.24 (-1.13, 0.65) |  |  |  |  |  |
| Ventilator-free days | | | | | |  |  |  |  |
| 5 trials^6,9,11,15,16^ | Low risk | 3354 | -0.11 (-1.89, 1.67) | -0.21 (-0.97, 1.38) | REM P=0.37 |  |  |  |  |
| 3 trials^7,10,12^ | Some concerns/high risk | 122 | 1.86 (-3.50, 7.22) | 1.86 (-3.50, 7.22) | FEM P=0.44 |  |  |  |  |
| 6 trials^6,7,9,12,15,16^ | Successful protocol | 3416 | 0.21 (-1.37, 1.80) | 0.34 (-0.83, 1.51) | REM P=0.59  FEM P=0.54 |  |  |  |  |
| 2 trials^10,11^ | Unsuccessful protocol | 60 | -0.98 (-6.45, 4.49) | -0.98 (-6.45, 4.49) |  |  |  |  |  |
| 2 trial^9,16^ | Sepsis | 1702 | -0.14 (-2.73, 2.44) | 0.29 (-0.94, 1.52) | REM P=0.73  FEM P=0.90 |  |  |  |  |
| 4 trials^6,7,10,15^ | Septic shock | 1772 | 0.49 (-3.40, 4.39) | 0.49 (-3.40, 4.39) |  |  |  |  |  |
| 4 trials^6,7,12,15^ | Fluid-only protocol | 1774 | 0.78 (-3.04, 4.61) | 0.78 (-3.04, 4.61) | REM P=0.24  FEM P=0.24 |  |  |  |  |
| 4 trials^9–11,16^ | Complex hemodynamic protocol | 1702 | -0.17 (-2.22, 1.89) | 0.23 (-0.97, 1.43) |  |  |  |  |  |
| 4 trials^9,10,12,16^ | Early | 1704 | -0.01 (-2.15, 2.12) | 0.30 (-0.92, 1.51) | REM P=0.92  FEM P=0.91 |  |  |  |  |
| 4 trials^6,7,11,15^ | Late | 1772 | 0.14 (-3.28, 3.56) | 0.14 (-3.28, 3.56) |  |  |  |  |  |
| Duration of vasopressor or inotropes (hours) | | | | | |  |  |  |  |
| 3 trials^9,15,16^ | Low risk | 3186 | 0.00 (-0.33, 0.33) | 0.00 (-0.33, 0.33) | REM P=0.52 |  |  |  |  |
| 3 trials^7,12,13^ | Some concerns/high risk | 274 | 6.60 (-19.84,33.04) | 8.89 (-6.42, 24.20) | FEM P=0.13 |  |  |  |  |
| 2 trial^9,16^ | Sepsis | 1655 | 0.00 (-0.33, 0.34) | 0.00 (-0.33, 0.34) | REM P=0.89  FEM P=0.69 |  |  |  |  |
| 2 trials^7,15^ | Septic shock | 1591 | 3.26 (-33.70, 40.21) | -2.58 (-19.23, 14.07) |  |  |  |  |  |
| 3 trials^7,12,15^ | Fluid-only protocol | 1681 | 5.98 (-14.60, 26.57) | 4.62 (-7.38, 16.62) | REM P=0.45  FEM P=0.32 |  |  |  |  |
| 3 trials^9,13,16^ | Complex hemodynamic protocol | 1779 | 0.00 (-0.33, 0.33) | 0.00 (-0.33, 0.33) |  |  |  |  |  |
| 4 trials^9,12,13,16^ | Early | 1876 | 2.22 (-6.48, 10.92) | 0.00 (-0.33, 0.34) | REM P=0.94  FEM P=0.69 |  |  |  |  |
| 2 trials^7,15^ | Late | 1591 | 3.26 (-33.70, 40.21) | -2.58 (-19.23, 14.07) |  |  |  |  |  |
| Vasopressor-free days | | | | | |  |  |  |  |
| 3 ^9,15,16^ | Low risk | 3185 | 0.43 (-0.68, 1.53) | 0.43 (-0.68, 1.53) | REM P=0.87 |  |  |  |  |
| 2 trials^11,12^ | Some concerns/high risk | 139 | 0.69 (-3.21, 4.58) | 0.69 (-3.21, 4.58) | FEM P=0.87 |  |  |  |  |
| 4 trials^9,12,15,16^ | Successful protocol | 3294 | 0.48 (-0.60, 1.56) | 0.48 (-0.60, 1.56) | REM P=0.67  FEM P=0.67 |  |  |  |  |
| 1 trial^11^ | Unsuccessful protocol | 30 | -0.50 (-6.24, 5.24) | -0.50 (-6.24, 5.24) |  |  |  |  |  |
| 2 trial^9,16^ | Sepsis | 1654 | 0.49 (-0.64, 1.62) | 0.49 (-0.64, 1.62) | REM P=0.53  FEM P=0.53 |  |  |  |  |
| 1 trial^15^ | Septic shock | 1531 | -0.80 (-6.01, 4.41) | -0.80 (-6.01, 4.41) |  |  |  |  |  |
| 2 trials^12,15^ | Fluid-only protocol | 1640 | 0.43 (-3.29, 4.14) | 0.43 (-3.29, 4.14) | REM P=0.99  FEM P=0.99 |  |  |  |  |
| 3 trials^9,11,16^ | Complex hemodynamic protocol | 1684 | 0.45 (-0.66, 1.56) | 0.45 (-0.66, 1.56) |  |  |  |  |  |
| 3 trials^9,12,16^ | Early | 1763 | 0.54 (-0.57, 1.65) | 0.54 (-0.57, 1.65) | REM P=0.44  FEM P=0.44 |  |  |  |  |
| 2 trials^11,15^ | Late | 1561 | -0.66 (-4.52, 3.19) | -0.66 (-4.52, 3.19) |  |  |  |  |  |
| Use of renal replacement therapy | | | | | |  |  |  |  |
| 6 trials^6,9,11,14–16^ | Low risk | 3343 | 1.01 (0.80, 1.26) | 1.01 (0.80, 1.26) | REM P=0.28 |  |  |  |  |
| 3 trials^4,12,13^ | Some concerns/high risk | 302 | 0.61 (0.19, 1.96) | 0.70 (0.38, 1.29) | FEM P=0.15 |  |  |  |  |
| 7 trials^6,9,12–16^ | Successful protocol | 3533 | 0.98 (0.78, 1.22) | 0.97 (0.77, 1.22) | REM P=0.90  FEM P=0.92 |  |  |  |  |
| 2 trials^4,11^ | Unsuccessful protocol | 112 | 0.94 (0.48, 1.86) | 0.94 (0.48, 1.87) |  |  |  |  |  |
| 3 trials^9,14,16^ ** | Sepsis | 1698 | 1.00 (0.51, 1.96) | 1.00 (0.51, 1.96) | REM P=0.99  FEM P=0.99 |  |  |  |  |
| 3 trials^4,6,15^ | Septic shock | 1697 | 1.00 (0.79, 1.26) | 1.00 (0.79, 1.26) |  |  |  |  |  |
| 5 trials^4,6,12,14,15^ | Fluid-only protocol | 1921 | 1.00 (0.79, 1.25) | 1.00 (0.79, 1.25) | REM P=0.41  FEM P=0.45 |  |  |  |  |
| 4 trials^9,11,13,16^ | Complex hemodynamic protocol | 1724 | 0.75 (0.31, 1.80) | 0.83 (0.46, 1.50) |  |  |  |  |  |
| 5 trials^9,12–14,16^ | Early | 1918 | 0.72 (0.30, 1.73) | 0.81 (0.45, 1.46) | REM P=0.35  FEM P=0.38 |  |  |  |  |
| 4 trials^4,6,11,15^ | Late | 1727 | 1.00 (0.79, 1.26) | 1.00 (0.80, 1.26) |  |  |  |  |  |
| Duration of renal replacement therapy (days) | | | | | |  |  |  |  |
| 3 trials^9,14,15^ ^**^ | Low risk (all trials) | 1630 | -0.17 (-0.70, 0.37) | -0.17 (-0.70, 0.37) | FEM P=0.43 |  |  |  |  |
| 2 trials^9,14^ | Sepsis | 222 | -0.30 (-1.02, 0.42) | -0.30 (-1.02, 0.42) | REM P=0.47  FEM P=0.47 |  |  |  |  |
| 1 trial^15^ | Septic shock | 1531 | 0.00 (-0.80, 0.80) | 0.00 (-0.80, 0.80) |  |  |  |  |  |
| 2 trials^14,15^ | Fluid-only protocol | 1654 | 0.00 (-0.80, 0.80) | 0.00 (-0.80, 0.80) | REM P=0.47  FEM P=0.47 |  |  |  |  |
| 1 trial^9^ | Complex hemodynamic protocol | 99 | -0.30 (-1.02, 0.42) | -0.30 (-1.02, 0.42) |  |  |  |  |  |
| 2 trials^9,14^ | Early | 222 | -0.30 (-1.02, 0.42) | -0.30 (-1.02, 0.42) | REM P=0.47  FEM P=0.47 |  |  |  |  |
| 1 trial^15^ | Late | 1531 | 0.00 (-0.80, 0.80) | 0.00 (-0.80, 0.80) |  |  |  |  |  |
| Renal replacement therapy-free days | | | | | |  |  |  |  |
| 5 trials^6,9,11,15,16^ | Low risk (all trials) | 3285 | 0.51 (-0.75, 1.78) | 0.46 (-0.68, 1.61) | FEM P=0.30 |  |  |  |  |
| 4 trials ^6,9,15,16^ | Successful protocol | 3255 | 0.34 (-0.83, 1.52) | 0.34 (-0.83, 1.52) | REM P=0.21  FEM P=0.21 |  |  |  |  |
| 1 trial^11^ | Unsuccessful protocol | 30 | 3.00 (-2.36, 8.36) | 3.00 (-2.36, 8.36) |  |  |  |  |  |
| 2 trial^9,16^ | Sepsis | 1573 | 0.43 (-0.91, 1.77) | 0.40 (-0.81, 1.60) | REM P=0.62  FEM P=0.63 |  |  |  |  |
| 2 trials^6,15^ | Septic shock | 1682 | -0.56 (-5.54, 4.42) | -0.56 (-5.54, 4.42) |  |  |  |  |  |
| 2 trials^6,15^ | Fluid-only protocol | 1682 | -0.56 (-5.54, 4.42) | -0.56 (-5.54, 4.42) | REM P=0.50  FEM P=0.59 |  |  |  |  |
| 3 trials^9,11,16^ | Complex hemodynamic protocol | 1603 | 0.83 (-1.00, 2.67) | 0.52 (-0.66, 1.70) |  |  |  |  |  |
| 2 trial^9,16^ | Early | 1573 | 0.43 (-0.91, 1.77) | 0.40 (-0.81, 1.60) | REM P=0.69  FEM P=0.64 |  |  |  |  |
| 3 trials^6,11,15^ | Late | 1712 | 1.16 (-3.37, 5.70) | 1.09 (-2.56, 4.74) |  |  |  |  |  |
| Incidence of acute kidney injury | | | | | |  |  |  |  |
| 2 trials^14,15^ | Low risk | 1645 | 0.94 (0.75, 1.19) | 0.94 (0.75, 1.19) | REM P=0.98 |  |  |  |  |
| 1 trial^12^ | Some concerns/high risk | 109 | 0.98 (0.03, 36.26) | 0.98 (0.03, 36.26) | FEM P=0.98 |  |  |  |  |
| 1 trial^14^ | Sepsis | 123 | 0.91 (0.31, 2.72) | 0.91 (0.31, 2.72) | REM P=0.95  FEM P=0.95 |  |  |  |  |
| 2 trials^12,15^ | Septic shock | 1631 | 0.94 (0.74, 1.19) | 0.94 (0.74, 1.19) |  |  |  |  |  |
| 2 trials^12,14^ | Early | 232 | 0.92 (0.32, 2.61) | 0.92 (0.32, 2.61) | REM P=0.96  FEM P=0.96 |  |  |  |  |
| 1 trial^15^ | Late | 1522 | 0.94 (0.74, 1.19) | 0.94 (0.74, 1.19) |  |  |  |  |  |
| EXPLORATORY OUTCOMES | | | | | |  |  |  |  |
| Use of blood products (units) | | | | | |  |  |  |  |
| 3 trials^6,1514^ | Low risk | 1805 | 0.00 (-0.10, 0.10) | 0.00 (-0.10, 0.10) | REM P=0.70 |  |  |  |  |
| 2 trials^7,11^ | Some concerns/high risk | 90 | -0.24 (-1.60, 1.12) | -0.24 (-1.60, 1.12) | FEM P=0.70 |  |  |  |  |
| 4 trials^6,7,14,15^ | Successful protocol | 1865 | 0.00 (-0.10, 0.10) | 0.00 (-0.10, 0.10) | REM P=1.00  FEM P=1.00 |  |  |  |  |
| 1 trial^11^ | Unsuccessful protocol | 30 | 0.00 (-1.82, 1.82) | 0.00 (-1.82, 1.82) |  |  |  |  |  |
| 1 trial^14^ | Sepsis | 123 | 0.00 (-0.10, 0.10) | 0.00 (-0.10, 0.10) | REM P=0.86  FEM P=0.86 |  |  |  |  |
| 3 trials^6,7,15^ | Septic shock | 1742 | -0.05 (-0.71, 0.60) | -0.05 (-0.71, 0.60) |  |  |  |  |  |
| 4 trials^6,7,14,15^ | Fluid-only protocol | 1865 | 0.00 (-0.10, 0.10) | 0.00 (-0.10, 0.10) | REM P=1.00  FEM P=1.00 |  |  |  |  |
| 1 trial ^11^ | Complex hemodynamic protocol | 30 | 0.00 (-1.82, 1.82) | 0.00 (-1.82, 1.82) |  |  |  |  |  |
| 1 trial^14^ | Early | 123 | 0.00 (-0.10, 0.10) | 0.00 (-0.10, 0.10) | REM P=0.87  FEM P=0.87 |  |  |  |  |
| 4 trials^6,7,11,15^ | Late | 1772 | -0.05 (-0.66, 0.57) | -0.05 (-0.66, 0.57) |  |  |  |  |  |
| ICU length of stay (days) | | | | | |  |  |  |  |
| 6 trials^5,6,9,12,14,15^ | Low risk | 1952 | -0.33 (-1.01, 0.35) | -0.33 (-1.01, 0.35) | REM P=0.96 |  |  |  |  |
| 2 trials^7,13^ | Some concerns/high risk | 184 | -0.20 (-6.44, 6.04) | -0.92 (-4.10, 2.25) | FEM P=0.68 |  |  |  |  |
| 6 trials^6,7,9,12,13,15^ | Successful protocol | 2057 | -0.27 (-0.93, 0.38) | -0.27 (-0.93, 0.38) | REM P=0.07  FEM P=0.07 |  |  |  |  |
| 2 trials^5,10^ | Unsuccessful protocol | 64 | -3.47 (-7.26, 0.32) | -3.47 (-7.26, 0.32) |  |  |  |  |  |
| 1 trial^9^ | Sepsis | 99 | 0.10 (-0.90, 1.10) | 0.10 (-0.90, 1.10) | REM P=0.35 |  |  |  |  |
| 5 trials^5–7,10,15^ | Septic shock | 1806 | -0.49 (-1.43, 0.45) | -0.49 (-1.43, 0.45) |  |  |  |  |  |
| 5 trials^5–7,12,15^ | Fluid-only protocol | 1883 | -0.41 (-1.30, 0.47) | -0.41 (-1.30, 0.47) | FEM P=0.35 |  |  |  |  |
| 3 trials^9,10,13^ | Complex hemodynamic protocol | 238 | -1.75 (-4.73, 1.23) | -0.30 (-1.24, 0.65) |  |  |  |  |  |
| 4 trials^9,10,12,13^ | Early | 345 | -1.29 (-3.20, 0.62) | -0.41 (-1.27, 0.46) | REM P=0.35 |  |  |  |  |
| 4 trials^5–7,15^ | Late | 1776 | -0.30 (-1.26, 0.67) | -0.30 (-1.26, 0.67) |  |  |  |  |  |
| Hospital length of stay (days) | | | | | |  |  |  |  |
| 3 trials^13–15^ | Low risk | 1754 | 0.78(-0.68, 2.24) | 0.78(-0.68, 2.24) | REM P=0.35 |  |  |  |  |
| 2 trials^9,12^ | Some concerns/high risk | 259 | -0.53 (-3.17, 2.11) | -0.53 (-3.17, 2.11) | FEM P=0.35 |  |  |  |  |
| 2 trials^9,14^ | Sepsis | 223 | 1.18 (-0.72, 3.08) | 1.18 (-0.72, 3.08) | REM P=0.47  FEM P=0.47 |  |  |  |  |
| 1 trial^15^ | Septic shock | 1531 | 0.20 (-2.87, 2.47) | 0.20 (-2.87, 2.47) |  |  |  |  |  |
| 3 trials^12,14,15^ | Fluid-only protocol | 1763 | 0.67 (-0.76, 2.10) | 0.67 (-0.76, 2.10) | REM P=0.51  FEM P=0.51 |  |  |  |  |
| 2 trials^9,13^ | Complex hemodynamic protocol | 250 | -0.31 (-3.15, 2.53) | -0.31 (-3.15, 2.53) |  |  |  |  |  |
| 4 trials^9,12–14^ | Early | 509 | 0.60 (-0.80, 1.99) | 0.60 (-0.80, 1.99) | REM P=0.75  FEM P=0.75 |  |  |  |  |
| 1 trial^15^ | Late | 1531 | 0.20 (-1.85, 2.25) | 0.20 (-1.85, 2.25) |  |  |  |  |  |

CI: Confidence interval, FEM: Fixed effect model, ICU: Intensiv care unit, MD: mean difference, REM: random effects model, RR: relative risk

*97% CI for primary and exploratory outcomes and 99% for secondary outcomes

** One zero-event trial not included^14^

# 14. e-Table 9 Sensitivity analyses for missing data across all outcomes

| **Outcome measure** | **No. of trials with missing data**  **(no. patients)** | | **RR (97% CI or 99% CI)** | **Heterogeneity** |
| --- | --- | --- | --- | --- |
| **Primary Outcomes 97% CI** | | | | |
| Mortality | 4 trials^9,14–16^ (32) | Best-worst scenario | 0.96 (0.88, 1.05) P=0.36 | I^2^=0% Tau^2^=0 |
|  |  | Worst-best scenario | 1.01 (0.92, 1.10) P=0.84 | I^2^=0% Tau^2^=0 |
| Serious adverse events | 2 trials^13,15^ (25) | Best-worst scenario | 0.91 (0.81, 1.02) P=0.10 | I^2^=0% Tau^2^=0 |
|  |  | Worst-best scenario | 0.98 (0.87, 1.10) P=0.69 | I^2^=0% Tau^2^=0 |
| **Secondary Outcomes 99% CI** | | | | |
| Duration of mechanical ventilation | 3 trials^13,15,16^ (63) | Best-worst scenario | -0.45 (-0.83, 0.06) P<0.01 | I^2^=84% Tau^2^=3.71 |
|  |  | Worst-best scenario | -0.02 (-0.37, 0.41) P=0.88 | I^2^=0% Tau^2^=0 |
| Ventilator-free days | 3 trials^9,15,16^ (38) | Best-worst scenario | 1.03 (-0.13, 2.19) P=0.02 | I^2^=0% Tau^2^=0 |
|  |  | Worst-best scenario | 0.04 (-1.12, 1.20) P=0.93 | I^2^=0% Tau^2^=0 |
| Duration of vasopressor or inotropes | 3 trials^13,15,16^ (51) | Best-worst scenario | 0.00 (-0.33, 0.33) P=0.99 | I^2^=77% Tau^2^=468.13 |
|  |  | Worst-best scenario | 0.01 (-0.32, 0.34) P=0.94 | I^2^=43% Tau^2^=25.64 |
| Vasopressor-free days | 4 trials^12,13,15,16^ (30) | Best-worst scenario | 0.77 (-0.31, 1.84) P=0.07 | I^2^=0% Tau^2^=0 |
|  |  | Worst-best scenario | 0.14 (-0.93, 1.22) P=0.73 | I^2^=0% Tau^2^=0 |
|  | 3 trials^13,15,16^ (64) | Best-worst scenario | 0.76 (0.41, 1.41) P=0.25 | I^2^=52% Tau^2^=0.24 |
| Use of renal replacement therapy |  | Worst-best scenario | 1.11 (0.90, 1.36) P=0.20 | I^2^=0 Tau^2^=0 |
| Duration of renal replacement therapy | 1 trial^15^ (18) | Best-worst scenario | -0.29 (-0.83, 0.25) P=0.17 | I^2^=0% Tau^2^=0 |
|  |  | Worst-best scenario | -0.03 (-0.77, 0.72) P=0.93 | I^2^=47% Tau^2^=0.08 |
| Renal replacement therapy -free days | 3 trials^9,15,16^ (34) | Best-worst scenario | 0.89 (-0.27, 2.04) P=0.05 | I^2^=0 Tau^2^=0 |
|  |  | Worst-best scenario | 0.05 (-1.11, 1.20) P=0.92 | I^2^=31% Tau^2^=0.78 |
| Incidence of acut kidney injury | 1 trial^15^ (18) | Best-worst scenario | 0.93 (0.74, 1.18) P=0.45 | I^2^=0% Tau^2^=0 |
|  |  | Worst-best scenario | 1.03 (0.82, 1.29) P=0.77 | I^2^=0% Tau^2^=0 |
| **Exploratory Outcomes 97% CI** | | | | |
| Use of blood products | 1 trial^15^ (18) | Best-worst scenario | 0.00 (-0.10, 0.09) P=0.91 | I^2^=0% Tau^2^=0 |
|  |  | Worst-best scenario | 0.00 (-0.10, 0.10) P=0.95 | I^2^=0% Tau^2^=0 |
| ICU length of stay | 2 trials^13,15^ (44) | Best-worst scenario | -0.67 (-1.32. 0.02) P=0.03 | I^2^=68% Tau^2^=4.50 |
|  |  | Worst-best scenario | 0.00 (-0.67. 0.66) P=0.99 | I^2^=0% Tau^2^=0 |
| Hospital length of stay | 1 trial^15^ (18) | Best-worst scenario | 0.17 (-1.11. 1.45) P=0.77 | I^2^=0% Tau^2^=0.07 |
|  |  | Worst-best scenario | 0.77 (-0.51. 2.05) P=0.19 | I^2^=0% Tau^2^=0 |

ICU: Intensive care unit, CI: Confidence interval, No.: Number, RR: Relative risk

# 15. e-Table 10 Full GRADE Evaluation of the Certainty of Evidence

| **Certainty assessment** | | | | | | | **№ of patients** | | **Effect** | | **Certainty** | **Importance** |
| --- | --- | --- | --- | --- | --- | --- | --- | --- | --- | --- | --- | --- |
| **№ of studies** | **Study design** | **Risk of bias** | **Inconsistency** | **Indirectness** | **Imprecision** | **Other considerations** | **Lower fluid volumes** | **Higher fluid volumes** | **Relative (97% or 99% CI)** | **Absolute (97% CI or 99% CI)** |  |  |
| All-cause mortality (low RoB trials only ) | | | | | | | | | | | | |
| 8 | randomized trials | not serious | not serious | not serious | serious^a^ | none | 556/1802 (30.9%) | 568/1824 (31.1%) | **RR 0.99** (0.89 to 1.10) | **3 fewer per 1.000** (from 34 fewer to 31 more) | ⨁⨁⨁◯ Moderate | CRITICAL |
| All-cause mortality | | | | | | | | | | | | |
| 13 | randomized trials | not serious | not serious | not serious | serious^b^ | none | 615/2005 (30.7%) | 624/1973 (31.6%) | **RR 0.98** (0.89 to 1.08) | **6 fewer per 1.000** (from 35 fewer to 25 more) | ⨁⨁⨁◯ Moderate | CRITICAL |
| SAEs (highest proportion) | | | | | | | | | | | | |
| 6 | randomized trials | serious^c^ | not serious | not serious | serious^d^ | none | 338/1817 (18.6%) | 358/1783 (20.1%) | **RR 0.95** (0.83 to 1.07) | **10 fewer per 1.000** (from 34 fewer to 14 more) | ⨁⨁◯◯ Low | CRITICAL |
| SAEs (highest proportion incl. mortality) | | | | | | | | | | | | |
| 13 | randomized trials | serious^e^ | not serious | serious^f^ | not serious^g^ | none | 681/2021 (33.7%) | 688/1985 (34.7%) | **RR 0.99** (0.90 to 1.08) | **3 fewer per 1.000** (from 35 fewer to 28 more) | ⨁⨁◯◯ Low | CRITICAL |
| SAEs (cumulated) | | | | | | | | | | | | |
| 13 | randomized trials | serious^e^ | not serious | serious^f,h^ | not serious^g^ | none | 1024/2021 (50.7%) | 1035/1985 (52.1%) | **RR 1.00** (0.97 to 1.02) | **0 fewer per 1.000** (from 16 fewer to 10 more) | ⨁⨁◯◯ Low | CRITICAL |
| Health-related quality of life | | | | | | | | | | | | |
| 0 | randomized trials |  |  |  |  |  | 0/0 | 0/0 | not estimable |  | - | CRITICAL |
| Duration (days) of MV (only low RoB trials) | | | | | | | | | | | | |
| 4 | randomized trials | not serious | not serious | not serious | not serious^i^ | none | 1653 | 1672 | - | MD 0.11 days lower (0.5 lower to 0.28 higher) | ⨁⨁⨁⨁ High | IMPORTANT |
| Duration (days) of MV | | | | | | | | | | | | |
| 6 | randomized trials | serious^j^ | not serious^k^ | not serious | serious^l^ | none | 1751 | 1730 | - | MD 0.21 days lower (0.59 lower to 0.17 higher) | ⨁⨁◯◯ Low | IMPORTANT |
| Ventilator-free days (low RoBs trials only) | | | | | | | | | | | | |
| 5 | randomized trials | not serious | not serious | not serious | serious^m^ | none | 1667 | 1687 | - | MD 0.11 days lower (1.89 lower to 1.67 higher) | ⨁⨁⨁◯ Moderate | IMPORTANT |
| Ventilator-free days | | | | | | | | | | | | |
| 8 | randomised trials | not serious | not serious | not serious | serious^n^ | none | 1727 | 1749 | - | MD 0.15 days higher (1.31 lower to 1.61 higher) | ⨁⨁⨁◯ Moderate | IMPORTANT |
| Duration (hours) of vasopressor or inotropes (low RoBs trials only) | | | | | | | | | | | | |
| 3 | randomized trials | not serious | not serious | not serious | not serious^o^ | none | 1583 | 1603 | - | MD 0 hours  (0.33 lower to 0.33 higher) | ⨁⨁⨁⨁ High | IMPORTANT |
| Duration (hours) of vasopressor or inotropes | | | | | | | | | | | | |
| 6 | randomized trials | not serious | not serious | not serious | not serious^p^ | none | 1743 | 1717 | - | MD 0 hours  (0.33 lower to 0.34 higher) | ⨁⨁⨁⨁ High | IMPORTANT |
| Vasopressor-free days (low RoB trials only) | | | | | | | | | | | | |
| 3 | randomized trials | not serious | not serious | not serious | serious^q^ | none | 1582 | 1603 | - | MD 0.43 days higher (0.68 lower to 1.53 higher) | ⨁⨁⨁◯ Moderate | IMPORTANT |
| Vasopressor-free days | | | | | | | | | | | | |
| 5 | randomized trials | not serious | not serious | not serious | serious^r^ | none | 1652 | 1672 | - | MD 0.45 days higher (0.62 lower to 1.51 higher) | ⨁⨁⨁◯ Moderate | IMPORTANT |
| Use of RRT(low RoB trials only) | | | | | | | | | | | | |
| 6^s^ | randomized trials | not serious | not serious | not serious | serious^t^ | none | 203/1662 (12.2%) | 206/1681 (12.3%) | **RR 1.01** (0.80 to 1.26) | **1 more per 1.000** (from 25 fewer to 32 more) | ⨁⨁⨁◯ Moderate | IMPORTANT |
| Use of RRT | | | | | | | | | | | | |
| 9^s^ | randomized trials | not serious | not serious | not serious | serious^u^ | none | 224/1830 (12.2%) | 232/1815 (12.8%) | **RR 0.97** (0.78 to 1.20) | **4 fewer per 1.000** (from 28 fewer to 26 more) | ⨁⨁⨁◯ Moderate | IMPORTANT |
| Duration (days) of RRT (all trials are low RoB trials) | | | | | | | | | | | | |
| 3^v^ | randomized trials | not serious | not serious | not serious | not serious^w^ | none | 866 | 887 | - | MD 0.17 days lower (0.7 lower to 0.37 higher) | ⨁⨁⨁⨁ High | IMPORTANT |
| RRT-free days (all trials are low RoB trials) | | | | | | | | | | | | |
| 5 | randomized trials | not serious | not serious | not serious | serious^x^ | none | 1631 | 1654 | - | MD 0.46 days higher (0.68 lower to 1.61 higher) | ⨁⨁⨁◯ Moderate | IMPORTANT |
| Incidence of AKI (low RoB trials only) | | | | | | | | | | | | |
| 2 | randomized trials | not serious | not serious | not serious | serious^y^ | none | 182/811 (22.4%) | 199/834 (23.9%) | **RR 0.94** (0.75 to 1.19) | **14 fewer per 1.000** (from 60 fewer to 45 more) | ⨁⨁⨁◯ Moderate | IMPORTANT |
| Incidence of AKI | | | | | | | | | | | | |
| 3 | randomized trials | not serious | not serious | not serious | serious^z^ | none | 183/866 (21.1%) | 200/888 (22.5%) | **RR 0.94** (0.75 to 1.19) | **14 fewer per 1.000** (from 56 fewer to 43 more) | ⨁⨁⨁◯ Moderate | IMPORTANT |
| Use of blood products - units (low RoB trials only) | | | | | | | | | | | | |
| 3 | randomized trials | not serious | not serious | not serious | not serious^aa^ | none | 891 | 914 | - | MD 0 units  (0.1 lower to 0.1 higher) | ⨁⨁⨁⨁ High | IMPORTANT |
| Use of blood products (units) | | | | | | | | | | | | |
| 5 | randomized trials | not serious | not serious | not serious | not serious^ab^ | none | 936 | 959 | - | MD 0 units  (0.1 lower to 0.1 higher) | ⨁⨁⨁⨁ High | IMPORTANT |
| ICU length of stay - days (low RoB trials only) | | | | | | | | | | | | |
| 6 | randomized trials | not serious | not serious | not serious | not serious^ac^ | none | 965 | 982 | - | MD 0.33 days lower (0.99 lower to 0.33 higher) | ⨁⨁⨁⨁ High | IMPORTANT |
| ICU length of stay (days) | | | | | | | | | | | | |
| 8 | randomized trials | not serious | not serious | not serious | serious^ad^ | none | 1078 | 1058 | - | MD 0.36 days lower (1 lower to 0.29 higher) | ⨁⨁⨁◯ Moderate | IMPORTANT |
| Hospital length of stay - days (low RoB trials only) | | | | | | | | | | | | |
| 3 | randomized trials | not serious | not serious | not serious | serious^ae^ | none | 866 | 888 | - | MD 0.78 days higher (0.73 lower to 2.28 higher) | ⨁⨁⨁◯ Moderate | IMPORTANT |
| Hospital length of stay (days) | | | | | | | | | | | | |
| 5 | randomized trials | not serious | not serious | not serious | serious^af^ | none | 1023 | 990 | - | MD 0.47 days higher (0.85 lower to 1.79 higher) | ⨁⨁⨁◯ Moderate | IMPORTANT |

No: number, CI: confidence interval, RoB: Risk of Bias, SAEs: serious adverse events, RR: risk ratio, RRR: Relative risk reduction, MD: mean difference, ICU: Intensive care unit

Explanations

a. TSA highlighted that 83% of RIS was reached. The area of futility to detect a predefined RRR of 15% was reached, however, the CI overlaps no effect and we cannot exclude important benefit or harm. Adjusted CI 0.89 to 1.11 (from 34 fewer to 34 more).

b. TSA highlighted that 93% of RIS was reached. The area of futility to detect a predefined RRR of 15% was crossed, however the CI overlaps no effect and we cannot exclude important benefit or harm. Adjusted CI 0.89 to 1.08 (from 35 fewer to 25 more).

c. Risk of bias were adjudicated as some concerns for all six trials based on measurement of the outcome, deviations from intended intervention or selection of the reported result.

d. TSA highlighted that 46% of RIS was reached. Adjusted CI 0.78 to 1.15 (from 44 fewer to 30 more).

e. Risk of bias were adjudicated as some concerns for all SAEs (high proportion) and as some concerns or high risk for five of the trials on all-cause mortality.

f. SAE was not defined or reported in all trials and the definitions varied across the trials. We prespecified to employ mortality as an SAE according to the ICH-GCP categorisation. Hence, we consider evidence on effect of treatment on SAE as indirect.

g. TSA highlighted that RIS was reached thus the TSA-adjusted CI is identical to the unadjusted CI. The boundary for futility was crossed, hence a relative risk reduction of 15% is unlikely. However, the CI overlaps no effect and we cannot exclude important benefit or harm.

h. All of the reported SAEs (including mortality) are cumulated, which might overestimate the treatment effect.

i. TSA highlighted that the boundary for futility crossed and RIS was reached, thus the adjusted CI is identical to the unadjusted. Thus, we can exclude a predefined mean difference of 1 day.

j. Indication of substantial statistical heterogeneity as suggested by I^2^=66% explained by differences in subgroup effect in trials with low vs high/some concerns risk of bias.

k. I^2^ 66% (D^2^=93%) explained by risk of bias, and therefore downgraded in the risk of bias domain.

l. TSA highlighted that 22% of RIS was reached. Adjusted CI -5.29 to 3.66.

m. TSA highlighted that 34% of RIS was reached. Adjusted CI -1.86 to 2.27

n. TSA highlighted that 35% of RIS was reached. Adjusted CI -1.66 to 2.23.

o. TSA highlighted that the boundary for futility crossed and RIS was reached, thus the adjusted CI is identical to the unadjusted. Therefore, we can exclude a predefined mean difference of 24 hours.

p. TSA highlighted that RIS of 217 was reached based on a predefined MD of 24 hours. Thus, we can exclude a predefined mean difference of 24 hours.

q. TSA highlighted that 38% of RIS was reached. Adjusted CI -1.41 to 2.27.

r. TSA highlighted that 41% of RIS was reached. Adjusted CI -1.25 to 2.14.

s. Jessen et al. had zero-events and was not included in meta-analysis or TSA.

t. TSA highlighted that 19% of RIS was reached. Adjusted CI 0.49 to 2.04 (from 62 fewer to 127 more).

u. TSA highlighted that 22% of RIS was reached. Adjusted CI 0.50 to 1.89 (from 64 fewer to 114 more).

v. Jessen et al. had zero-events and was not included in meta-analysis or TSA. The remaining three trials are low RoB trials.

w. TSA highlighted that the boundary for futility crossed and RIS was reached, thus the adjusted CI is identical to the unadjusted. Thus, we can exclude a predefined mean difference of 1 day.

x. TSA highlighted that 35% of RIS was reached. Adjusted CI -1.50 to 2.43.

y. TSA highlighted that 22% of RIS was reached. Adjusted CI 0.46 to 1.93.

z. TSA highlighted that 21% of RIS was reached. Adjusted CI 0.46 to 1.89.

aa. TSA highlighted that RIS was reached and the boundary for futility crossed based on a predefined MD of one unit of blood product.

ab. TSA highlighted that RIS was reached based on a predefined MD of one unit of blood product.

ac. TSA highlighted 87% of RIS was reached and the boundary for futility crossed based on a predefined MD of 1 day. Adjusted CI -1.07 to 0.40.

ad. TSA highlighted that 91% of RIS was reached. Adjusted CI -1.05 to 0.34

ae. TSA highlighted that 18% of RIS was reached. Adjusted CI -4.60 to 6.15.

af. TSA highlighted that 23% of RIS was reached. Adjusted CI -2.52 to 3.46

# 16. References

1. Meyhoff TS, Møller MH, Hjortrup PB, Cronhjort M, Perner A, Wetterslev J. Lower vs Higher Fluid Volumes During Initial Management of Sepsis: A Systematic Review With Meta-Analysis and Trial Sequential Analysis. *Chest*. 2020;157(6):1478-1496.

2. Meyhoff TS, Møller MH, Hjortrup PB, Cronhjort M, Perner A, Wetterslev J. Lower vs. higher fluid volumes in sepsis—protocol for a systematic review with meta-analysis. *Acta Anaesthesiol Scand*. 2017;61(8):942-951.

3. Page MJ, McKenzie JE, Bossuyt PM, et al. The PRISMA 2020 statement: An updated guideline for reporting systematic reviews. *BMJ*. 2021;372.

4. Chen C, Kollef MH. Targeted fluid minimization following initial resuscitation in septic shock a pilot study. *Chest*. 2015;148(6):1462-1469.

5. Cronhjort M, Bergman M, Joelsson-Alm E, et al. Fluid Responsiveness Assessment Using Passive Leg Raising Test to Reduce Fluid Administration and Weight Gain in Patients with Septic Shock. *J Anesth Perioper Med*. 2017;4(January).

6. Hjortrup PB, Haase N, Bundgaard H, et al. Restricting volumes of resuscitation fluid in adults with septic shock after initial management: the CLASSIC randomised, parallel-group, multicentre feasibility trial. *Intensive Care Med*. 2016;42(11):1695-1705.

7. Richard JC, Bayle F, Bourdin G, et al. Preload dependence indices to titrate volume expansion during septic shock: A randomized controlled trial. *Crit Care*. 2015;19(1):1-13.

8. van Genderen M.E.; Engels, N.; van der Valk, R.J.P.; Lima, A.; Klijn, E.; Bakker, J.; van Bommel J. Early Peripheral Perfusion–guided Fluid Therapy in Patients with Septic Shock. *Am J Respir Crit Care Med*. 2015;191(4):477-480.

9. Macdonald SPJ, Keijzers G, Taylor DMD, et al. Restricted fluid resuscitation in suspected sepsis associated hypotension (REFRESH): a pilot randomised controlled trial. *Intensive Care Med*. 2018;44(12):2070-2078.

10. Lanspa MJ, Burk RE, Wilson EL, Hirshberg EL, Grissom CK, Brown SM. Echocardiogram-guided resuscitation versus early goal-directed therapy in the treatment of septic shock: A randomized, controlled, feasibility trial. *J Intensive Care*. 2018;6(1).

11. Semler, M.; Janz, D.R. ; Casey, J.D. ; Self, W.H. ; Rice TW. Conservative Fluid Management after Sepsis Resuscitation: A Pilot Randomized Trial. *J Intensive Care Med*. 2020;35(12):1374-1382.

12. Corl KA, Prodromou M, Merchant RC, et al. The Restrictive IV Fluid Trial in Severe Sepsis and Septic Shock (RIFTS): A Randomized Pilot Study. *Crit Care Med*. 2019;47(7):951-959.

13. Douglas IS, Alapat PM, Corl KA, et al. Fluid Response Evaluation in Sepsis Hypotension and Shock: A Randomized Clinical Trial. *Chest*. 2020;158(4):1431-1445.

14. Jessen MK, Andersen LW, Thomsen MH, et al. Restrictive Fluids Versus Standard Care in Adults with Sepsis in the Emergency Department ( REFACED ) – a Multicenter, Randomized Feasibility Trial . *Acad Emerg Med*. Published online 2022.

15. Meyhoff TS, Hjortrup PB, Wetterslev J, et al. Restriction of Intravenous Fluid in ICU Patients with Septic Shock. *N Engl J Med*. 2022;386(26):2459-2470.

16. The National Heart, Lung, and Blood Institute Prevention and Early Treatment of Acute Lung Injury Clinical Trials Network; Shapiro NI, Douglas IS BR et al. Early Restrictive or Liberal Fluid Management for Sepsis-Induced Hypotension. *N Engl J Med*. Published online 2023:1-12.

17. Jakobsen JC, Wetterslev J, Winkel P, Lange T, Gluud C. Thresholds for statistical and clinical significance in systematic reviews with meta-analytic methods. *BMC Med Res Methodol*. 2014;14(1):1-13.

18. Jakobsen JC, Gluud C, Winkel P, Lange T, Wetterslev J. The thresholds for statistical and clinical significance - A five-step procedure for evaluation of intervention effects in randomised clinical trials. *BMC Med Res Methodol*. 2014;14(1):1-12.

19. Hammond NE, Zampieri FG, Di Tanna GL, et al. Balanced Crystalloids versus Saline in Critically Ill Adults — A Systematic Review with Meta-Analysis. *NEJM Evid*. 2022;1(2):1-12.

20. Turner RM, Jackson D, Wei Y, Thompson SG, Higgins JPT. Predictive distributions for between-study heterogeneity and simple methods for their application in Bayesian meta-analysis. *Stat Med*. 2015;34(6):984-998.

21. Turner RM, Davey J, Clarke MJ, Thompson SG, Higgins JP. Predicting the extent of heterogeneity in meta-analysis, using empirical data from the Cochrane Database of Systematic Reviews. *Int J Epidemiol*. 2012;41(3):818-827.

22. Granholm A, Munch MW, Myatra SN, et al. Dexamethasone 12 mg versus 6 mg for patients with COVID-19 and severe hypoxaemia: a pre-planned, secondary Bayesian analysis of the COVID STEROID 2 trial. *Intensive Care Med*. 2022;48(1):45-55.

23. Gabry J, Simpson D, Vehtari A, Betancourt M, Gelman A. Visualization in Bayesian workflow. *J R Stat Soc Ser A Stat Soc*. 2019;182(2):389-402.

24. Vehtari A, Gelman A, Gabry J. Practical Bayesian model evaluation using leave-one-out cross-validation and WAIC. *Stat Comput*. 2017;27(5):1413-1432.
